# Causality of genetically determined metabolites on susceptibility to prevalent urological cancers: a two-sample Mendelian randomization study and meta-analysis

**DOI:** 10.3389/fgene.2024.1398165

**Published:** 2024-07-01

**Authors:** Xianyu Dai, Hongjie Wang, Rong Zhong, Jiajun Li, Yuchuan Hou

**Affiliations:** ^1^ Urology Department, First Hospital of Jilin University, Changchun, China; ^2^ Department of Hepatic Surgery, The First Affiliated Hospital of Harbin Medical University, Harbin, China

**Keywords:** urological cancers, metabolomics, Mendelian randomization, genetically determined metabolites, genome-wide association studies, meta-analysis, causality, FinnGen

## Abstract

**Background:**

Prevalent urological cancers, including kidney, prostate, bladder, and testicular cancers, contribute significantly to global cancer incidence and mortality. Metabolomics, focusing on small-molecule intermediates, has emerged as a tool to understand cancer etiology. Given the knowledge gap in this field, we employ a two-sample Mendelian randomization (MR) analysis to investigate the causal relationships between genetically determined metabolites (GDMs) and the susceptibility to four common urological cancers.

**Methods:**

The study employs genome-wide association studies (GWAS) data from European populations, featuring the most extensive case count available for both blood metabolites and four prevalent urological cancers. Preliminary and secondary MR analyses were separately conducted, employing inverse variance weighted (IVW) as the primary method. Multiple statistical analyses, including the MR-Steiger test, Cochran’s Q test, leave-one-out analysis, MR-Egger intercept analysis, and MR-PRESSO analysis, were executed to ensure robustness. Additionally, a meta-analysis was carried out to consolidate findings. The weighted median (WM) method was utilized for a relatively lenient correction (P_WM_ < 0.05).

**Results:**

After rigorous genetic variation filtering, 645 out of 1,400 metabolites were included in both preliminary and secondary MR analyses. Preliminary MR analysis identified 96 potential causal associations between 94 distinct metabolites and four urological cancers. Secondary analysis based on Finnish outcome data revealed 93 potential causal associations. Cross-database meta-analysis identified 68 blood metabolites associated with four urological cancers. Notably, 31 metabolites remained significant after using WM for correction, with additional 37 suggestive causal relationships. Reverse MR analysis revealed a significant causal association between genetically predicted prostate cancer and elevated 4-hydroxychlorothalonil levels (IVW, combined OR: 1.039, 95% CI 1.014–1.064, *p* = 0.002; WM, combined OR: 1.052, 95% CI 1.010–1.095, *p* = 0.014).

**Conclusion:**

This comprehensive MR study provides insights into the causal relationships between blood metabolites and urological cancers, revealing potential biomarkers and therapeutic targets, thereby addressing gaps in understanding and laying the foundation for targeted interventions in urological cancer research and treatment.

## 1 Introduction

The prevalent cancers affecting the urinary system, encompassing kidney, prostate, bladder, and testicular cancers, present a formidable challenge in the realms of cancer epidemiology and clinical management. These malignancies significantly contribute to the global incidence and mortality rates of cancer, constituting a substantial component of the worldwide health burden (Sung et al., 2021). A focused research endeavor is imperative for comprehending the intricate etiology of urinary system cancers and devising effective prevention and treatment strategies. For instance, prostate cancer (PC) ranks as the second most prevalent cancer in global males, demonstrating noteworthy geographical variations in incidence and mortality rates (Rawla, 2019). These disparities underscore distinctions in genetic susceptibility, diagnostic practices, lifestyle factors, and healthcare access (Rawla, 2019). Bladder cancer (BC), the tenth most globally diagnosed cancer, exhibits a male predilection (Sung et al., 2021) and is markedly influenced by factors such as smoking (Cumberbatch et al., 2016; van Osch et al., 2016), occupational carcinogen exposure (Rota et al., 2014), and environmental exposure (el-Mawla et al., 2001). Kidney cancer (KC), the eleventh most common cancer in global males (Sung et al., 2021), often manifests asymptomatically, leading to later-stage diagnosis and a higher proportion of metastasis (Motzer et al., 1996). Despite being relatively rare, testicular carcinoma (TC) is the most prevalent cancer in young males of European descent (Sung et al., 2021) and has witnessed an increase in incidence in Western countries (Van Hemelrijck et al., 2013). While treatment modalities have made strides, including surgery, radiation therapy, chemotherapy, targeted therapy, and immunotherapy, the prognosis for metastatic urinary system cancers remains pessimistic (Stecca et al., 2021; Rahnea-Nita et al., 2023), underscoring the urgency of early detection and personalized treatment paradigms.

Metabolite research, focusing on small-molecule intermediates and products of metabolic processes, has garnered attention in cancer research (Gu et al., 2022). Metabolomics has been applied to identify biomarkers by revealing altered metabolic pathways and intermediate metabolites, offering in-depth insights into the occurrence and development of diseases (Lains et al., 2019). Numerous studies indicate that metabolites, as functional intermediates, aid in elucidating potential disease genetic biology mechanisms (Yang et al., 2020; Xiao et al., 2022) and may serve as biomarkers for personalized cancer treatment (Wishart, 2019). Genome-wide association studies (GWAS) of blood metabolites have identified numerous genetic loci associated with endogenous metabolite levels (Chen et al., 2023), paving the way for understanding the genetic basis of metabolic diversity and its significance in disease susceptibility.

Emerging evidence suggests that changes in specific metabolites are associated with the risk of urinary system cancers. For instance, dysregulation in lipid metabolism, including increased lipogenesis and β-oxidation, is linked to a heightened risk of PC (Deep and Schlaepfer, 2016; Zekovic et al., 2023). Additionally, metabolites like 4-hydroxynonenal and 2-hydroxybutyric acid have been significantly elevated in the plasma of PC patients (Perkovic et al., 2023). Abnormal amino acid metabolism may contribute to the progression of KC (Sciacovelli et al., 2022). Liu et al. utilized liquid chromatography-mass spectrometry to identify significant elevations of 12,13-DHOME and 9,10,13-TriHOME in BC patients (Liu et al., 2020). Furthermore, serum phosphocholine levels were notably increased in patients with TC (Cheng et al., 2016). Collectively, these findings highlight the potential of metabolomics profiling as biomarkers for cancer risk and progression. However, ethical considerations often limit the ability to validate the causal relationships between serum metabolite levels and cancer incidence. Therefore, there is an urgent need for innovative research methods to infer causality and advance our understanding of these associations.

Mendelian randomization (MR) analysis is a method that utilizes the random allocation of alleles at conception to assess causality in relationships (Smith and Ebrahim, 2003). It provides a unique opportunity to study the causal role of genetically determined metabolites (GDMs) in urinary system cancers. MR uses genetic variation, often single nucleotide polymorphisms (SNPs), as instrumental variables (IVs) for the exposure of interest (Burgess et al., 2015a), reducing common confounding and reverse causation seen in traditional epidemiological studies (Lu, 2009; Smit et al., 2014).

Recent MR studies have applied this method to investigate the causal impact of various risk factors on urological cancers (Chen et al., 2020; Xiong et al., 2022). By integrating GWAS data on GDMs with GWAS data on urinary system cancer susceptibility, MR analysis can provide robust evidence linking changes in metabolism to cancer risk.

The necessity of our study arises from the knowledge gap regarding the causality of metabolite-urinary system cancer associations and the potential for metabolic-targeted interventions. Utilizing GWAS data for blood metabolites and urological cancers, our two-sample MR study and subsequent meta-analysis aim to unravel the causal relationships between GDMs and the susceptibility of four common urinary system cancers. Our research not only aims to enhance our understanding of the metabolic pathways involved in urinary system cancer pathogenesis but also lays the groundwork for novel prevention and treatment strategies.

## 2 Materials and methods

### 2.1 Study design

**FIGURE 1:**
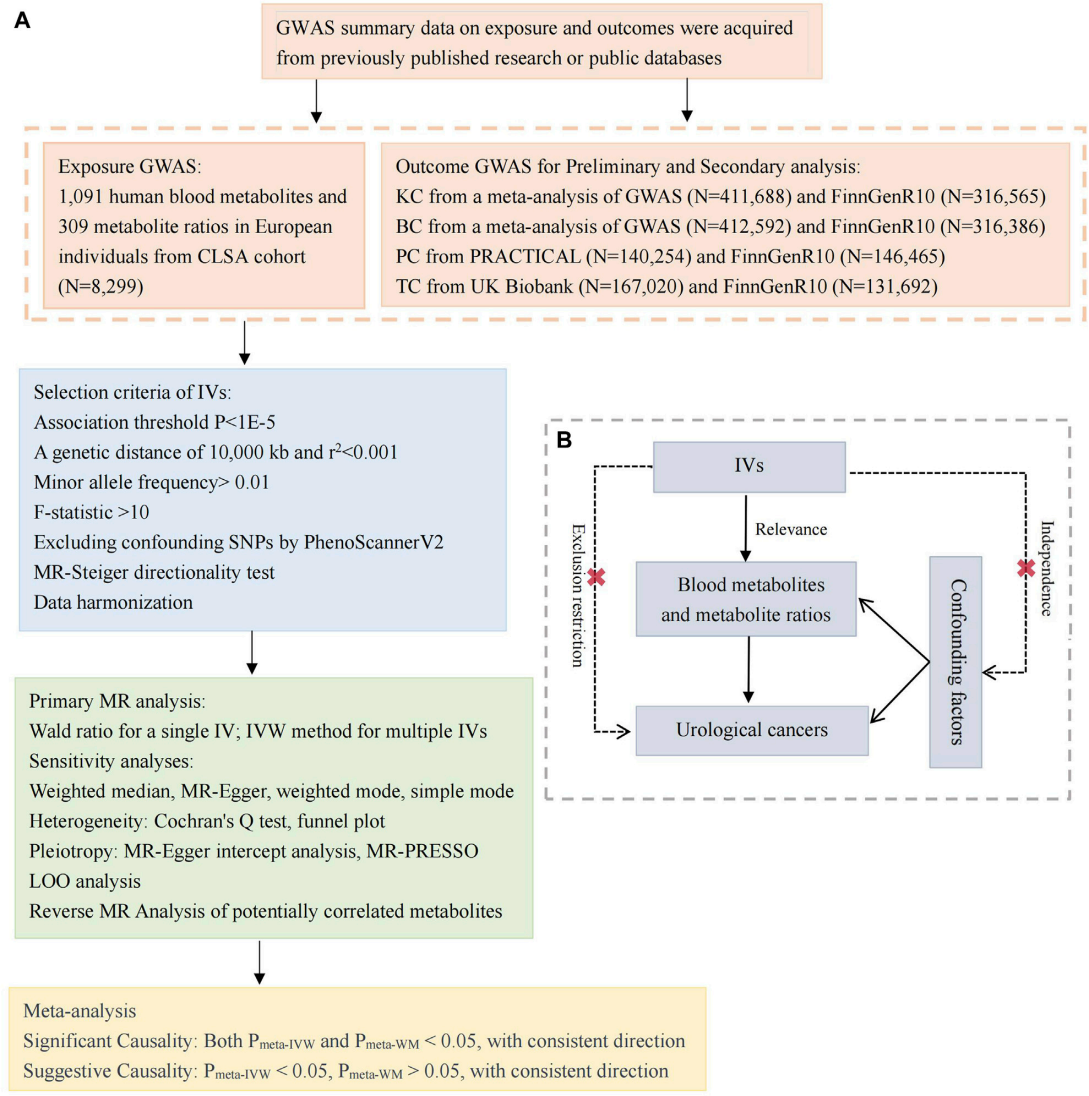
Study design and Mendelian randomization core assumption. **(A)** Data sources and study design of Mendelian randomization study. **(B)** Three core assumptions in the Mendelian randomization. GWAS, genome-wide association study; CLSA, Canadian Longitudinal Study of Aging; KC, kidney cancer; BC, bladder cancer, PC, prostate cancer; TC, testicular carcinoma; IVs, instrumental variables; SNPs, single nucleotide polymorphisms; MR, Mendelian randomization; LOO, Leave-one-out; IVW, inverse variance weighted; MR-PRESSO, MR pleiotropy residual sum and outlier; PRACTICAL, Prostate Cancer Association Group to Investigate Cancer-Associated Alterations in the Genome.

This study employs a two-sample MR analysis to investigate the causal relationship between 1,400 blood metabolites and the susceptibility to four common urological cancers. We acquired two sets of independent outcome GWAS data and subsequently conducted preliminary MR and secondary MR analyses on each dataset. This was followed by a meta-analysis to consolidate the findings. The foundational dataset for this study comprises publicly available data. All included studies obtained approval from their respective academic ethics committees, and each participant provided informed consent. As this study does not involve the use of raw data, ethical approval was not required. Figure 1A illustrates the overall framework of the study design. Utilizing genetic variations as IVs for MR analysis requires adherence to three crucial assumptions, as depicted in Figure 1B: 1) Relevance: Variables selected as genetic instruments must be closely associated with blood metabolites; 2) Independence: Genetic variations should be unrelated to confounding factors; 3) Exclusion restriction: Genetic variations should not influence outcomes through pathways other than affecting blood metabolites (Lawlor et al., 2008; Emdin et al., 2017).

### 2.2 GWAS data for human blood metabolites

Statistical data for 1,091 blood metabolites and 309 metabolite ratios were sourced from Chen’s study (Chen et al., 2023). Specifically, this study conducted GWAS and MR analyses on 8,299 European individuals from the Canadian Longitudinal Study on Aging (CLSA). Quantitative measurement of 1,458 plasma metabolites using the Metabolon HD4 platform underwent rigorous quality control, standardization, and screening. The study identified genetic variation data for 1,091 blood metabolites and 309 metabolite ratios. Additionally, 248 and 69 gene loci were discovered to be significantly associated with 690 metabolites and 143 metabolite ratios, respectively.

### 2.3 GWAS data for urological cancers

To ensure the accuracy and reliability of our MR study, we selected GWAS statistical data with the largest available case count for European populations. In the preliminary MR analysis, genetic variation data for KC and BC were derived from meta-analyses with sample sizes of 411,688 and 412,592, respectively. PC data originated from the Prostate Cancer Association Group to Investigate Cancer-Associated Alterations in the Genome (PRACTICAL) study (N = 140,254), and TC data came from the UK Biobank (N = 167,020). For the secondary MR analysis, outcome data were sourced from the FinnGen consortium (Kurki et al., 2023), with sample sizes for KC, BC, PC, and TC being 316,565, 316,386, 146,465, and 131,692, respectively (see Supplementary Table S1 for details), publicly accessible at the website: https://www.finngen.fi/fi.

### 2.4 Selection of IVs

To ensure the authenticity and accuracy of causal relationships, the study implemented quality control steps for IVs selection. Initially, a genome-wide significance threshold of 5 × 10^−8^ was used, resulting in a limited number of metabolite IVs. To capture more potential causal associations between blood metabolites and urological malignancies, the threshold was extended to 1 × 10^−5^, a common practice in current MR studies (Yu et al., 2021; Xiao et al., 2022; Yin et al., 2023). Subsequently, IVs with linkage disequilibrium (LD) (r^2^ > 0.001 or clustering distance within 10,000 kb) were excluded to ensure their independence. A minor allele frequency (MAF) threshold of 0.01 for relevant genetic variations was set. To prevent bias from strand direction or allele coding, palindrome and synonymous SNPs were excluded. Phenotypic information related to selected SNPs was queried using PhenoScannerV2 to eliminate SNPs associated with outcomes or other confounding factors (Staley et al., 2016; Kamat et al., 2019). F-statistics [F = R^2^ (N-2)/(1-R^2^)] for each SNP were calculated to identify potential weak IVs, with a threshold of F > 10 used to exclude weak IVs (Pierce et al., 2011). Here, N represents the sample size of the exposure variable, and R^2^ represents the proportion of exposure variable variance explained by IVs.

For reverse MR, to ensure an adequate number of exposure-related IVs, significance thresholds were set at 1E-5 for BC, 1E-6 for KC, 5E-8 for PC, 1E-5 for TC (UK Biobank), and 5E-8 for TC (FinnGen consortium). Other screening criteria remained consistent with those mentioned previously.

### 2.5 Statistical analysis

To assess the strength of IVs in explaining exposure versus outcome variations, MR-Steiger test was conducted to validate the causal relationships’ directionality (Hemani et al., 2017). In investigating the link between blood metabolites and urological cancers, we employed the Wald ratio for a single IV and a random-effects inverse-variance weighted (IVW) model for multiple IVs (Burgess et al., 2013; Burgess et al., 2015b), serving as our primary MR analysis methods. IVW, a widely used approach, combines Wald ratios for each SNP to generate a comprehensive estimate (Pierce and Burgess, 2013). Additionally, sensitivity analyses included weighted median (WM) (Bowden et al., 2016), MR-Egger (Bowden et al., 2015), weighted mode (Hartwig et al., 2017), and simple mode methods. WM, adept at combining data from multiple genetic variants into a single causal estimate, exhibits decreased type I error rates in limited samples, even when up to 50% of genetic variants are invalid (Bowden et al., 2016). Heterogeneity tests utilized Cochran’s Q test and funnel plots. Lack of significant heterogeneity was assumed with Q-test *p*-values exceeding 0.05 and symmetric funnel plots. To assess the impact of horizontal pleiotropy, an MR-Egger intercept analysis was conducted (Bowden et al., 2015). Significance of the intercept term (*p* < 0.05) would indicate potential pleiotropic effects of the selected IVs. The MR pleiotropy residual sum and outlier (MR-PRESSO) method (Verbanck et al., 2018) were employed to identify and correct for outlier SNPs impacting estimation results. To ensure study robustness, leave-one-out (LOO) analysis systematically evaluated the impact of each removed SNP on the overall causal relationship estimate (Flatby et al., 2023). In preliminary and secondary MR analyses, a potential causal relationship was considered when P_IVW_ was <0.05, and the other four sensitivity analysis effect directions were consistent. Subsequently, a meta-analysis of potential meaningful metabolites from two independent outcome datasets was conducted. A two-sided *p*-value <0.05 was considered statistically significant. If heterogeneity (I^2^) between two MR analyses was ≥50%, a random-effects model was used; otherwise, a fixed-effects model was employed to combine odds ratio (OR) values from the IVW and WM methods separately. To correct for multiple testing false-positive errors, a significant causal relationship was considered when both P_meta-IVW_ and P_meta-WM_ to be < 0.05, with consistent effect directions. A potential causal association was presumed if only P_meta-IVW_ <0.05 and the effect direction of WM was consistent. All analyses were performed using R 4.2.3 software with the Mendelian Randomization (0.9.0), TwoSample MR (0.5.6) (Hemani et al., 2018), and meta (6.5.0) packages.

## 3 Results

The results of this study are divided into several key sections. First, IVs were selected. Next, a preliminary analysis explored the potential causal relationships between these metabolites and four urological cancers. Subsequently, a secondary analysis using data from the FinnGen consortium was conducted. Finally, a cross-database meta-analysis confirmed significant causal associations. Each section is detailed below.

### 3.1 Selection of IVs

Following a series of IVs selection steps, a total of 645 metabolites were identified for subsequent MR estimation (755 metabolites were excluded due to the absence of available IVs) (Supplementary Table S2). Supplementary Table S3 lists the significant SNPs associated with each metabolite and their corresponding traits (PhenoScannerV2). Supplementary Table S4 lists the confounding SNPs excluded in this study. The number of IVs for each metabolite ranged from 6 to 42, with a minimum F-statistic value of 19.507, surpassing the threshold of 10, indicating the absence of weak instrument bias. All IVs passed the MR-Steiger filtering (TRUE). Detailed IVs information is provided in Supplementary Material S2. In the reverse MR analysis, the number of IVs for the four urological malignancies ranged from 1 to 109, with a minimum F-statistic value of 19.539. All IVs passed the MR-Steiger filtering (TRUE). Detailed IVs information of reverse MR is available in Supplementary Table S5.

### 3.2 Preliminary analysis

**FIGURE 2:**
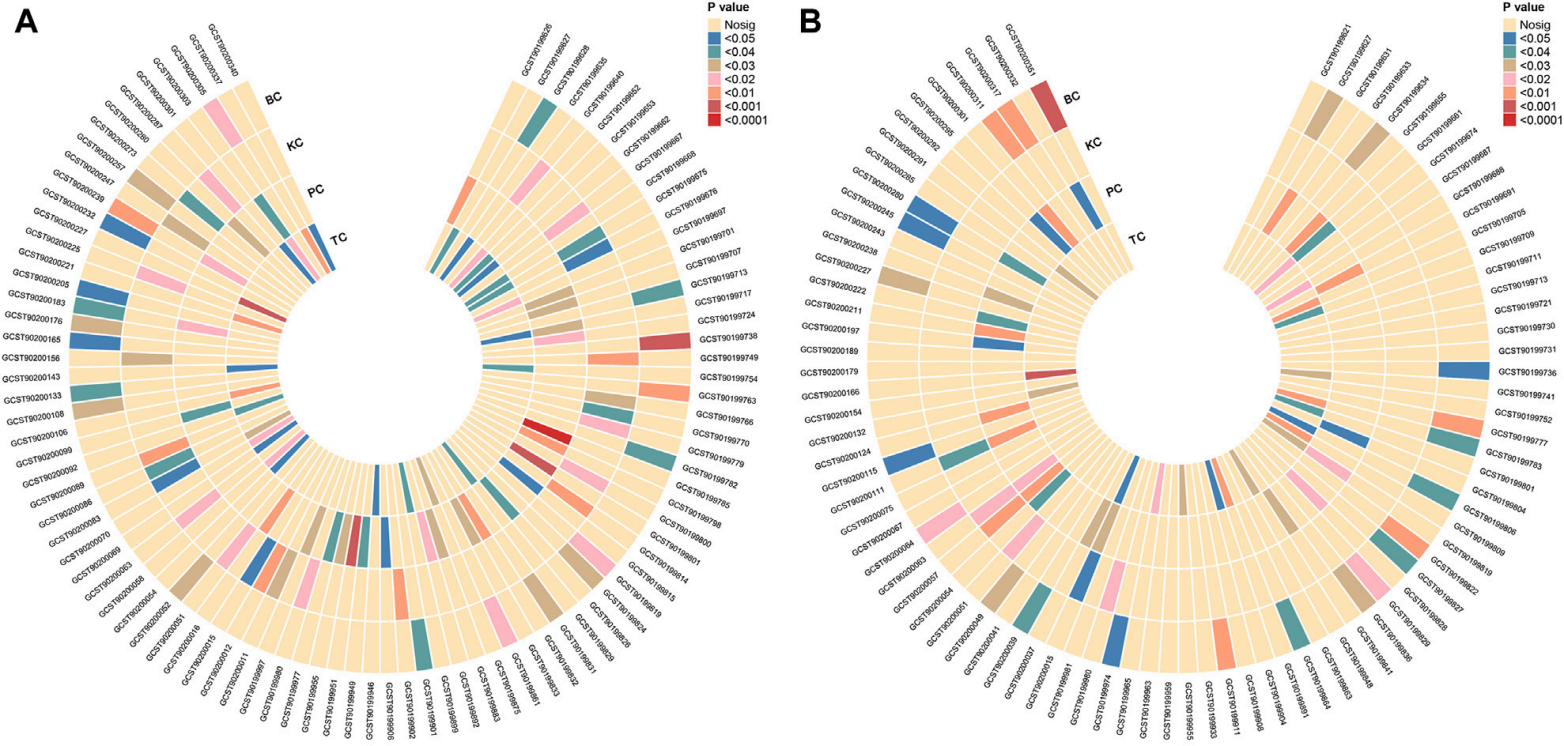
Mendelian randomization association of four urological cancers with blood metabolites. **(A)** Preliminary analysis reveals 96 potential causal associations, involving 94 distinct and known metabolites. **(B)** Secondary analysis, focused on Finnish outcomes, identifies 93 potential causal associations, linked to 86 unique and known metabolites. KC, kidney cancer; BC, bladder cancer; PC, prostate cancer; TC, testicular carcinoma.

Employing the IVW method, we identified 96 potential causal associations (P_IVW_<0.05), corresponding to 94 distinct and known metabolites (Figure 2A). Specifically, 9, 11, 11, and 17 GDMs displayed potential negative associations with the incidence risks of BC, KC, PC, and TC, respectively (Supplementary Table S6). Conversely, 11, 13, 15, and 9 GDMs exhibited potential positive associations with the incidence risks of BC, KC, PC, and TC, respectively (Supplementary Table S6).

Sensitivity analyses, including WM, MR-Egger, weighted mode, and simple mode, consistently supported the findings, maintaining a uniform direction of effect for the 96 causal associations identified by IVW (Supplementary Table S6). No evidence of potential horizontal pleiotropy was observed (all P_MR-Egger intercept_ > 0.05, Supplementary Table S7). Notably, the Cochran Q test indicated heterogeneity in the analysis of 1-(1-enyl-palmitoyl)-GPE (p-16:0) on PC (both P_MR-Egger.Q_ and P_IVW.Q_ <0.05). However, LOO analysis demonstrated robust results without high-influence SNPs, and MR-PRESSO did not identify any outliers.

Heterogeneity was also detected in the analysis of 1-methylxanthine on BC, N-acetylalliin on PC, and glycoursodeoxycholic acid sulfate on TC (both P_MR-Egger.Q_ and P_IVW.Q_ <0.05), and outliers were also identified through MR-PRESSO analysis (P_MR-PRESSO Global_ <0.05). After excluding outliers (Supplementary Table S8) for correction, we attained acceptable heterogeneity and global test *p*-values (Supplementary Table S9), with causal effects remaining unchanged (all P_IVW_ < 0.05). No heterogeneity or outliers were detected for the remaining causal associations (Supplementary Table S10). LOO analyses and funnel plots are provided in Supplementary Material S3. All preliminary MR analysis results are summarized in Supplementary Table S11.

In a reverse MR analysis focusing on the 96 identified causal associations, predominantly utilizing the IVW method, we discovered potential links. Specifically, higher genetic predictions of 4-hydroxychlorothalonil were associated with an increased risk of PC (OR: 1.040, 95% confidence interval (CI) 1.004–1.077, *p* = 0.030), while higher genetic predictions of 3-methyladipate were associated with a decreased risk of TC (OR: 2.01E-07, 95%CI 0.000–0.703, *p* = 0.045). Sensitivity analysis did not reveal evidence of heterogeneity or pleiotropy (Supplementary Tables S12, S13), and the reverse MR results are detailed in Supplementary Table S14.

### 3.3 Secondary analysis based on Finnish outcome

Using outcome GWAS data from the FinnGen consortium, we conducted a comprehensive secondary analysis. Employing the IVW method, we identified 93 potential causal associations corresponding to 86 unique and known metabolites (P_IVW_ < 0.05, Figure 2B). Specifically, 9, 16, 11, and 11 GDMs exhibited potential negative associations with the incidence risks of BC, KC, PC, and TC, respectively (Supplementary Table S15). Conversely, 11, 16, 12, and 7 GDMs displayed potential positive associations with the incidence risks of BC, KC, PC, and TC, respectively Supplementary Table S15).

Crucially, the following causal relationships aligned consistently with the preliminary analysis: an elevated genetic prediction of 1-palmitoyl-2-docosahexaenoyl-GPE (16:0/22:6) (OR: 1.116, 95%CI 1.002–1.242, *p* = 0.046), a decreased N-formylphenylalanine (OR: 0.846, 95%CI 0.740–0.967, *p* = 0.014), and a decreased 1-palmitoyl-2-dihomo-linolenoyl-GPC (16:0/20:3n3 or 6) (OR: 0.867, 95%CI 0.765–0.983, *p* = 0.026) were potentially associated with increased susceptibility to KC. Additionally, an elevated genetic prediction of 5-oxoproline (OR: 1.052, 95%CI 1.003–1.104, *p* = 0.039) was potentially associated with increased susceptibility to PC.

For the 93 causal associations identified by IVW, four sensitivity analyses consistently yielded concordant conclusions with a consistent direction of effect (Supplementary Table S15). No evidence of potential horizontal pleiotropy was observed (all P_MR-Egger intercept_ > 0.05, Supplementary Table S7). Notably, the Cochran Q test indicated heterogeneity in the analysis of glycocholenate sulfate on PC (both P_MR-Egger.Q_ and P_IVW.Q_ < 0.05) and adrenate (22:4n6) on TC (P_IVW.Q_ < 0.05). However, LOO analyses showed robust results without high-influence SNPs, and MR-PRESSO identified no outliers.

Heterogeneity was also detected in the analysis of sphingomyelin (d18:2/24:2) on PC, 3-hydroxypyridine glucuronide on PC, and 1-(1-enyl-palmitoyl)-2-oleoyl-GPC (P-16:0/18:1) on KC (both P_MR-Egger.Q_ and P_IVW.Q_ < 0.05), and outliers were also identified through MR-PRESSO analysis (P_MR-PRESSO Global_ < 0.05). After excluding outliers (Supplementary Table S8) for correction, we attained acceptable heterogeneity and global test *p*-value (Supplementary Table S9), with causal effects remaining unchanged (all P_IVW_ < 0.05). No heterogeneity or outliers were detected for the remaining causal associations (Supplementary Table S10). LOO analyses and funnel plots are provided in Supplementary Material S4. All secondary MR analysis results are summarized in Supplementary Table S11.

In a reverse MR analysis focusing on the 93 identified causal associations, primarily utilizing the IVW method, no evidence of reverse causal relationships was found (all P_IVW_ > 0.05). The complete reverse MR results are detailed in Supplementary Table S14.

### 3.4 Confirmation of the causal relationship by cross-database meta-analysis

**FIGURE 3:**
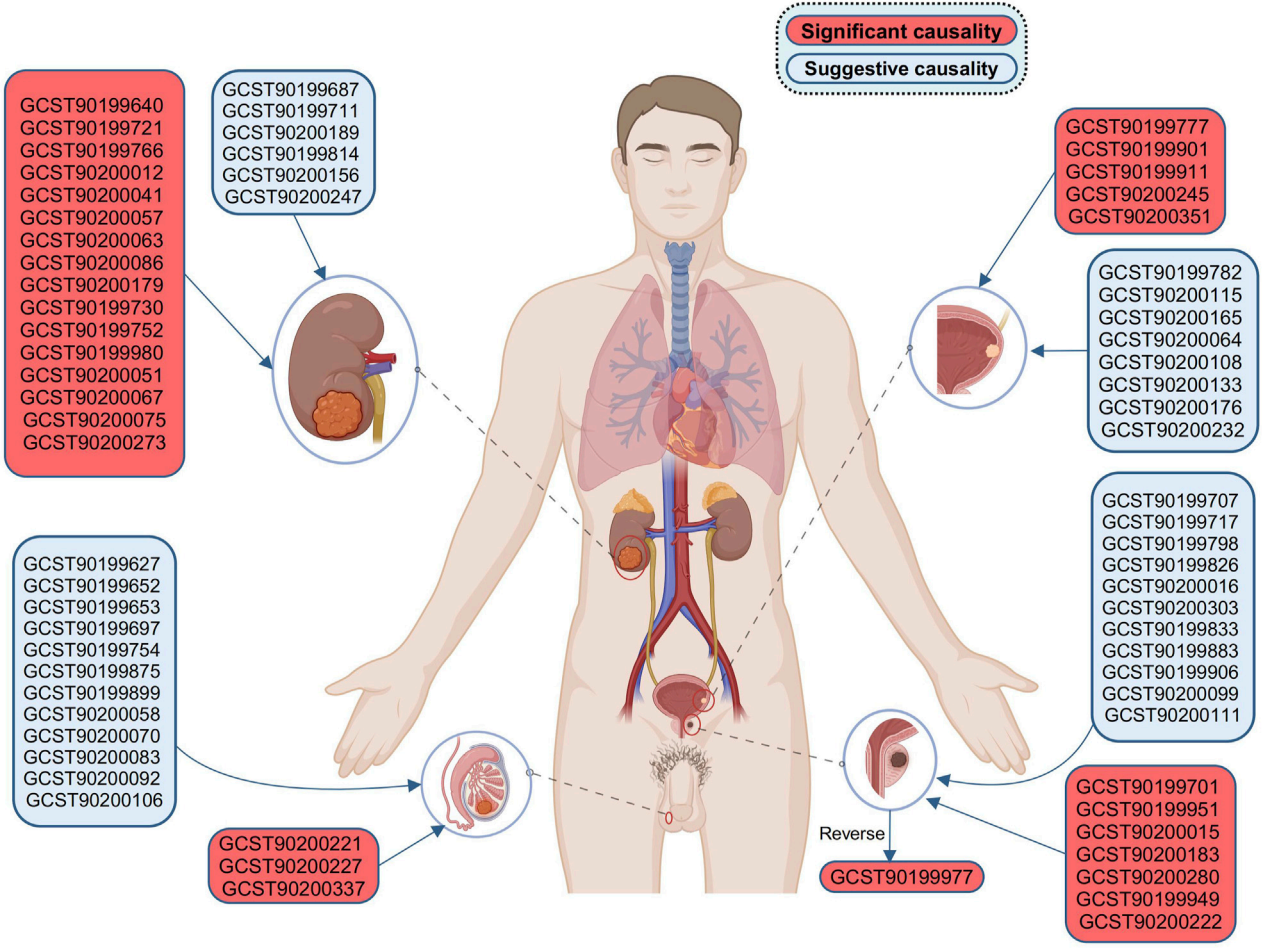
Bidirectional causal association of four urological cancers with blood metabolites. Results for KC and BC apply to all genders, while PC and TC are limited to males. BC, bladder cancer; KC, kidney cancer; PC, prostate cancer; TC, testicular carcinoma. Created with BioRender.com.

**FIGURE 4:**
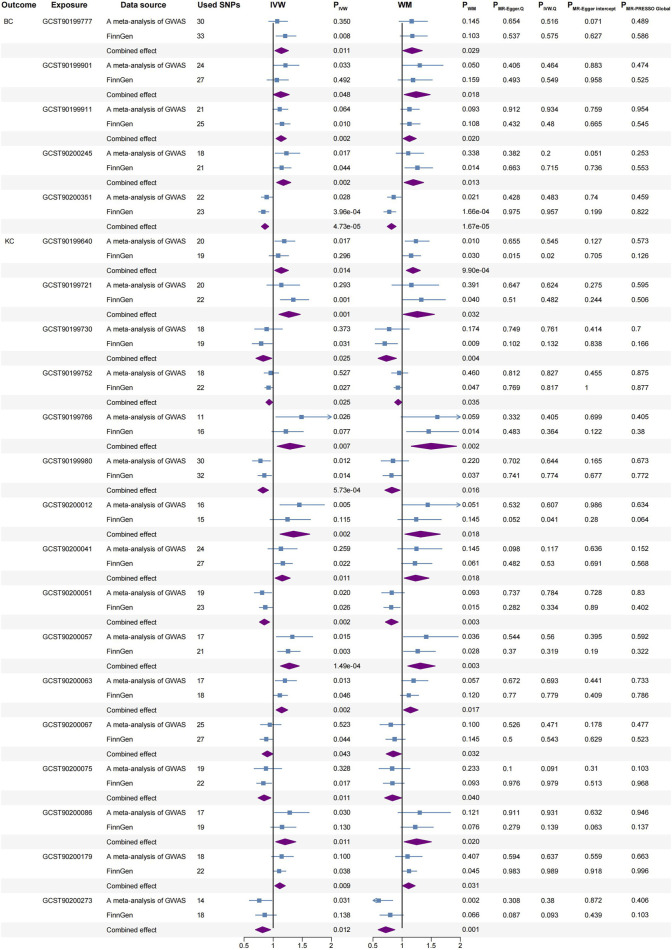
Significant causal associations between BC and KC with blood metabolites using the IVW and WM methods, along with sensitivity analyses. BC, bladder cancer; KC, kidney cancer; SNPs, single nucleotide polymorphisms; IVW, inverse variance weighted; WM, weighted median.

**FIGURE 5:**
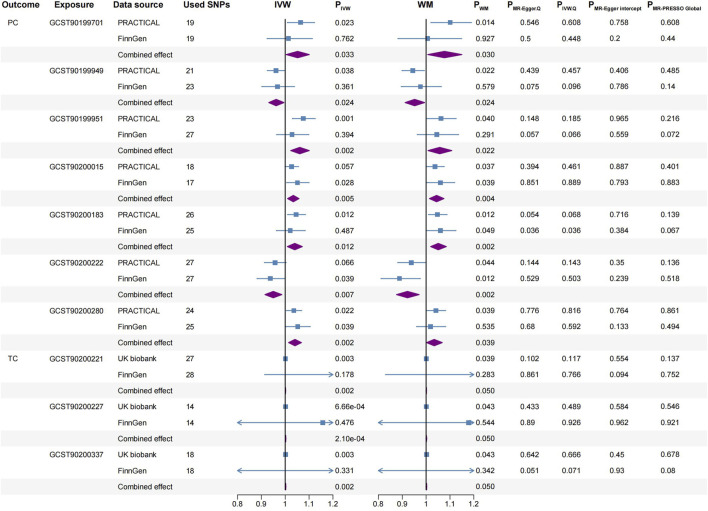
Significant causal associations between PC and TC with blood metabolites using the IVW and WM methods, along with sensitivity analyses. PC, prostate cancer; TC, testicular carcinoma; SNPs, single nucleotide polymorphisms; PRACTICAL, Prostate Cancer Association Group to Investigate Cancer-Associated Alterations in the Genome; IVW, inverse variance weighted; WM, weighted median.

Before the meta-analysis, we thoroughly screened for pleiotropy, excluding three causal associations (P_MR-Egger intercept_ < 0.05 in the other analysis, Supplementary Table S16). This step ensured the robustness of the meta-analysis results. The meta-analysis, based on the IVW method, revealed 68 causal associations of blood metabolites on four urological cancers (P_meta-IVW_ < 0.05, Supplementary Table S17). These associations corresponded to 68 specific blood metabolites (Figure 3). After applying the WM method for correction, we observed 31 blood metabolites significantly associated with four urological cancers (both P_meta-IVW_ and P_meta-WM_ < 0.05, Figures 4, 5). The remaining 37 associations are considered suggestive of causality (Figure 3). The detailed significant causal relationships are as follows:

BC: 1-palmitoleoyl-GPC (16:1) (IVW, combined OR: 1.141, 95%CI 1.031–1.262, *p* = 0.011; WM, combined OR: 1.170, 95%CI 1.016–1.347, *p* = 0.029), 1-methyl-5 imidazoleacetate (IVW, combined OR: 1.130, 95%CI 1.001–1.276, *p* = 0.048; WM, combined OR: 1.240, 95%CI 1.037–1.481, *p* = 0.018), Beta-citrylglutamate (IVW, combined OR: 1.135, 95%CI 1.049–1.227, *p* = 0.002; WM, combined OR: 1.125, 95%CI 1.019–1.243, *p* = 0.020) and Eicosenedioate (C20:1-DC) (IVW, combined OR: 1.175, 95%CI 1.060–1.303, *p* = 0.002; WM, combined OR: 1.191, 95%CI 1.037–1.368, *p* = 0.013) were associated with higher BC risk.

Conversely, N-acetylputrescine (IVW, combined OR: 0.859, 95%CI 0.798–0.924, *p* = 4.73E-05; WM, combined OR: 0.817, 95%CI 0.745–0.896, *p* = 1.67E-05) was correlated with a reduced risk of BC.

KC: 4-guanidinobutanoate (IVW, combined OR: 1.138, 95%CI 1.026–1.262, *p* = 0.014; WM, combined OR: 1.186, 95%CI 1.072–1.313, *p* = 0.001), Stearidonate (18:4n3) (IVW, combined OR: 1.269, 95%CI 1.099–1.465, *p* = 0.001; WM, combined OR: 1.259, 95%CI 1.020–1.554, *p* = 0.032), 3-(3-amino-3-carboxypropyl) uridine (IVW, combined OR: 1.287, 95%CI 1.070–1.548, *p* = 0.007; WM, combined OR: 1.496, 95%CI 1.157–1.934, *p* = 0.002), 3-hydroxyhexanoate (IVW, combined OR: 1.349, 95%CI 1.118–1.627, *p* = 0.002; WM, combined OR: 1.317, 95%CI 1.048–1.655, *p* = 0.018), 1-palmitoyl-2-stearoyl-GPC (16:0/18:0) (IVW, combined OR: 1.156, 95%CI 1.034–1.293, *p* = 0.011; WM, combined OR: 1.231, 95%CI 1.037–1.461, *p* = 0.018), 1-(1-enyl-palmitoyl)-2-oleoyl-GPC (P-16:0/18:1) (IVW, combined OR: 1.277, 95%CI 1.125–1.449, *p* = 1.49E-04; WM, combined OR: 1.311, 95%CI 1.097–1.566, *p* = 0.003), 1-palmitoyl-2-docosahexaenoyl-GPE (16:0/22:6) (IVW, combined OR: 1.146, 95%CI 1.050–1.250, *p* = 0.002; WM, combined OR: 1.143, 95%CI 1.024–1.275, *p* = 0.017), Arachidonoylcholine (IVW, combined OR: 1.204, 95%CI 1.043–1.389, *p* = 0.011; WM, combined OR: 1.248, 95%CI 1.036–1.503, *p* = 0.020) and Glyco-beta-muricholate (IVW, combined OR: 1.114, 95%CI 1.028–1.208, *p* = 0.009; WM, combined OR: 1.112, 95%CI 1.010–1.224, *p* = 0.031) were associated with higher KC risk.

Conversely, Carnitine C14 (IVW, combined OR: 0.829, 95%CI 0.704–0.977, *p* = 0.025; WM, combined OR: 0.728, 95%CI 0.587–0.902, *p* = 0.004), Epiandrosterone sulfate (IVW, combined OR: 0.932, 95%CI 0.876–0.991, *p* = 0.025; WM, combined OR: 0.932, 95%CI 0.872–0.995, *p* = 0.035), N-formylphenylalanine (IVW, combined OR: 0.825, 95%CI 0.739–0.920, *p* = 0.001; WM, combined OR: 0.824, 95%CI 0.704–0.964, *p* = 0.016), 1-palmitoyl-2-dihomo-linolenoyl-GPC (16:0/20:3n3 or 6) (IVW, combined OR: 0.848, 95%CI 0.765–0.939, *p* = 0.002; WM, combined OR: 0.815, 95%CI 0.712–0.933, *p* = 0.003), 1-stearoyl-2-linoleoyl-GPI (18:0/18:2) (IVW, combined OR: 0.899, 95%CI 0.810–0.997, *p* = 0.043; WM, combined OR: 0.847, 95%CI 0.727–0.986, *p* = 0.032), 1-(1-enyl-palmitoyl)-2-linoleoyl-GPE (p-16:0/18:2) (IVW, combined OR: 0.844, 95%CI 0.741–0.961, *p* = 0.011; WM, combined OR: 0.833, 95%CI 0.700–0.991, *p* = 0.040) and 2-methoxyhydroquinone sulfate (2) (IVW, combined OR: 0.817, 95%CI 0.697–0.956, *p* = 0.012; WM, combined OR: 0.718, 95%CI 0.589–0.875, *p* = 0.001) were correlated with a reduced risk of KC.

PC: Pyridoxate (IVW, combined OR: 1.052, 95%CI 1.004–1.101, *p* = 0.033; WM, combined OR: 1.077, 95%CI 1.007–1.150, *p* = 0.030), N-acetylalliin (IVW, combined OR: 1.061, 95%CI 1.023–1.101, *p* = 0.002; WM, combined OR: 1.057, 95%CI 1.008–1.108, *p* = 0.022), N-acetylkynurenine (2) (IVW, combined OR: 1.034, 95%CI 1.010–1.058, *p* = 0.005; WM, combined OR: 1.044, 95%CI 1.014–1.075, *p* = 0.004), N-acetyl-2-aminooctanoate (IVW, combined OR: 1.040, 95%CI 1.009–1.072, *p* = 0.012; WM, combined OR: 1.051, 95%CI 1.019–1.085, *p* = 0.002) and 5-oxoproline (IVW, combined OR: 1.041, 95%CI 1.014–1.068, *p* = 0.002; WM, combined OR: 1.034, 95%CI 1.002–1.068, *p* = 0.039) were associated with higher PC risk.

Conversely, 6-oxopiperidine-2-carboxylate (IVW, combined OR: 0.962, 95%CI 0.931–0.995, *p* = 0.024; WM, combined OR: 0.952, 95%CI 0.912–0.994, *p* = 0.024) and Dihydrocaffeate sulfate (2) (IVW, combined OR: 0.950, 95%CI 0.915–0.986, *p* = 0.007; WM, combined OR: 0.922, 95%CI 0.876–0.971, *p* = 0.002) were correlated with a reduced risk of PC.

TC: Glycoursodeoxycholic acid sulfate (1) (IVW, combined OR: 1.001, 95%CI 1.001–1.002, *p* = 0.002; WM, combined OR: 1.001, 95%CI 1.000–1.002, *p* = 0.0497), 3-ethylcatechol sulfate (2) (IVW, combined OR: 1.002, 95%CI 1.001–1.003, *p* = 2.10E-04; WM, combined OR: 1.001, 95%CI 1.000–1.003, *p* = 0.0497) and Taurochenodeoxycholate (IVW, combined OR: 1.002, 95%CI 1.001–1.003, *p* = 0.002; WM, combined OR: 1.002, 95%CI 1.000–1.003, *p* = 0.0496) were associated with higher TC risk.

For reverse MR analysis, we found a significant causal association between genetically predicted PC and the elevation of 4-hydroxychlorothalonil (IVW, combined OR: 1.039, 95% CI 1.014–1.064, *p* = 0.002; WM, combined OR: 1.052, 95% CI 1.010–1.095, *p* = 0.014, Figure 3).

## 4 Discussion

To our knowledge, this represents the first MR study to apply a combined genomics and metabolomics approach in assessing the causal relationships between GDMs and susceptibility to prevalent urological cancers, namely, KC, BC, PC, and TC. Utilizing GWAS data on metabolites from the CLSA cohort involving 8,299 individuals, we meticulously selected IVs for 645 metabolites, a more comprehensive scope than the previous 486 metabolites studied (Shin et al., 2014). The paucity of knowledge on the causal links between metabolomics and urological malignancies led us to leverage two sets of independent large-scale GWAS datasets with the largest available case numbers. To uncover meaningful and credible causal associations, preliminary and secondary MR analyses were conducted on these independent outcome GWAS data, followed by meta-analyses, further bolstering the robustness of our findings. Aware of the potential pitfalls associated with multiple testing, we employed the WM method for correction. This dual-threshold approach heightened the credibility of our results, identifying 31 metabolites exhibiting significant causal associations with four urological cancers and 37 metabolites showing suggestive causal associations with these cancers (Supplementary Table S17). These insights not only deepen our understanding of the pathogenic mechanisms underlying urological malignancies but also offer novel clues for future preventative and therapeutic strategies.

Notably, both primary and secondary MR analyses revealed that an increase in 1-palmitoyl-2-docosahexaenoyl-GPE (16:0/22:6) is associated with a heightened risk of KC, while decreased levels of N-formylphenylalanine and 1-palmitoyl-2-dihomo-linoleoyl-GPC correlate with increased KC risk. An elevation in 5-oxoproline is linked to an augmented PC risk.

For BC, our findings identified 1-palmitoyl-GPC, composed of palmitic acid, as a risk enhancer. Existing research hints at a potential association between palmitic acid and hypertension (Zheng et al., 1999; Kulkarni et al., 2013), with further evidence indicating a positive correlation between hypertension and BC (Kok et al., 2018). This suggests that hypertension might mediate the increased BC risk associated with 1-palmitoleoyl-GPC (16:1). Additionally, research shows that palmitic acid can activate various inflammatory mediators, such as NFκB and CCL2 (Wang et al., 2024), and induce inflammation through Toll-like receptors (TLR2 and TLR4), leading to the release of cytokines like IL-1β and IL-6 (Matsufuji et al., 2023). This heightened inflammatory response can promote cancer development. N-acetylputrescine (NAP), a derivative of putrescine, has been found to help reduce intracellular oxidative stress, enhance the ability of immune cells to recognize and destroy cancer cells, and regulate the activity of key enzymes such as ODC and SSAT to inhibit tumor cell proliferation (Liu et al., 2017; Li et al., 2020), thereby exerting anti-cancer effects. These findings are consistent with our study. However, there is a lack of research on the associations between cancer and other metabolites such as 1-methyl-5-imidazoleacetate, Beta-citrylglutamate, and Eicosenedioate (C20:1-DC). Additional investigation is needed to understand the mechanisms underlying these associations.

For KC, 1-palmitoyl-2-docosahexaenoyl-GPE (16:0/22:6) is a phospholipid that contains docosahexaenoic acid (DHA). While DHA has antioxidant properties (Jewell and Jackson, 2022), at high concentrations, it can also form harmful reactive oxygen species (ROS) through self-oxidation (Su et al., 2019). These ROS can cause DNA damage, thereby increasing cancer risk. Additionally, research by Shao et al. supports the link between elevated 1-palmitoyl-2-docosahexaenoyl-GPE (16:0/22:6) and metabolic dysfunction-associated fatty liver disease (MAFLD) (Shao et al., 2023). Consistently, Liu et al. (2022) reported an increased incidence of KC in MAFLD patients (OR:1.77, 95%CI 1.49–2.11, *p* < 0.001). These findings suggest that MAFLD may mediate the causal relationship between 1-palmitoyl-2-docosahexaenoyl-GPE (16:0/22:6) and KC, further supporting our conclusion. N-formylphenylalanine is a potent neutrophil chemoattractant with significant anti-inflammatory properties. By binding to specific receptors such as FPR1, N-formylphenylalanine can modulate immune cell activity and reduce inflammatory responses, helping to lower the risk of various cancers (Tennenberg et al., 1988; Snapkov et al., 2016). This is consistent with our research findings. 1-palmitoyl-2-dihomo-linoleoyl-GPC contains a Dihomo-γ-linolenic acid (DGLA) chain. Studies have shown that prostaglandins (such as PGE1) derived from the metabolism of DGLA exhibit anti-inflammatory effects, which may help reduce cancer risk by mitigating chronic inflammation (Das, 2006). Additionally, a study involving 1,111 Mexican Americans (Palmer et al., 2018) conducted a dynamic measure analysis and found an association between the improvement of insulin resistance (a hallmark of type 2 diabetes) and 1-palmitoyl-2-dihomo-linolenoyl-GPC (16:0/20:3n3 or 6). Simultaneously, a meta-analysis of nine cohort studies (Larsson and Wolk, 2011) found that patients with diabetes have a statistically significant increase in the likelihood of developing KC compared to those without diabetes (RR: 1.42, 95% CI 1.06–1.91). This suggests that a potential mechanism for the protective role of 1-palmitoyl-2-dihomo-linolenoyl-GPC (16:0/20:3n3 or 6) against KC could be the reduction of diabetes risk. Similarly, a study by Feofanova et al. (2020) implicates elevated 1-palmitoyl-2-stearoyl-GPC (16:0/18:0) in increasing type 2 diabetes risk (OR: 1.24, 95% CI = 1.10–1.40), shedding light on possible mechanisms by which certain metabolites might influence KC risk.

However, conflicting evidence exists, such as a study from the MetKid consortium (Guida et al., 2021), which in a crude model suggests an inverse relationship between 1-(1-enyl-palmitoyl)-2-oleoyl-GPC (P-16:0/18:1) levels and KC risk (OR: 0.83, 95% CI: 0.74–0.93). After adjusting for BMI, alcohol consumption, smoking, and hypertension, this inverse relationship lost its statistical significance (OR: 0.92, 95% CI: 0.79–1.07), contrary to our findings. Specifically, the study by Guida et al. is a nested case-control study within five prospective cohorts (EPIC, MCCS, NSHDS, Estonian BB, HUNT), involving 2,614 participants. These cohorts have participants with diverse genetic backgrounds and environmental factors. In contrast, our study has a larger sample size and is based on more uniform sample selection and rigorous genetic analysis methods, reducing heterogeneity between cohorts. Guida et al. used traditional observational methods, which may be subject to confounding bias and reverse causation. Our study, utilizing MR, employs genetic variants as IVs, minimizing these influences. Additionally, the metabolites measured in Guida et al.’s study lacked chemical standard confirmation, which could affect the reliability of their results. Our results, supported by two independent outcome MR analyses and meta-analysis (Figure 4), indicate 1-(1-enyl-palmitoyl)-2-oleoyl-GPC (P-16:0/18:1) as a risk factor for KC, warranting further research on its biological effects.

Turning to PC, our study linked Pyridoxate, a vitamin B6 metabolite (Costeira et al., 2023), to increased susceptibility of PC. A case-control study in 1997 (Key et al., 1997) suggested a protective role of higher vitamin B6 intake against PC. Yet, recent a meta-analysis does not support significant associations between dietary intake or plasma levels of vitamin B6 and PC (Mocellin et al., 2017). The biological effects of Pyridoxate remain inadequately studied in the context of academic research. A previous observational study that had identified an association between N-acetylkynurenine and PC (Huang et al., 2016). Building on this foundation, our current research substantiates the role of N-acetylkynurenine as a risk factor for PC. Furthermore, elevated levels of blood 5-oxoproline, also known as Pyroglutamic acid, have been found to correlate with pancreatic cancer (Wang et al., 2023a) and breast cancer (Park et al., 2019). Complementing these findings, an analysis conducted by Markin et al. (2020) revealed an increase in Pyroglutamic acid levels during the progression from prostatic intraepithelial neoplasia to PC. This observation aligns with the results of our study to some extent. However, research regarding the remaining metabolites causally associated with PC remains scant and warrants further investigation.

In the case of TC, although we identified three metabolites with significant causal relationships, their effect sizes were modest. The precise mechanisms through which glycoursodeoxycholic acid sulfate, 3-ethylcatechol sulfate, and taurochenodeoxycholate contribute to testicular cancer remain under investigation. It is hypothesized that their involvement in metabolic processes and cellular signaling pathways may create conditions conducive to tumorigenesis. Altered levels of bile acid conjugates, such as glycoursodeoxycholic acid sulfate and taurochenodeoxycholate, may disrupt homeostatic processes (Chiang, 2013). These disrupted homeostatic processes, in turn, regulate metabolism and cell death pathways, thereby influencing the tumor microenvironment and playing a crucial role in tumorigenesis and tumor progression (Yang et al., 2024). Simultaneously, metabolites like 3-ethylcatechol sulfate, related to catechol metabolism, could influence oxidative stress and hormonal imbalances (Chainy and Sahoo, 2020). While these associations require further validation through rigorous scientific research, they underscore the complexity of metabolic alterations in cancer development.

Moreover, our reverse MR analysis uncovered an intriguing association—PC may lead to elevated levels of 4-hydroxychlorothalonil in the blood. Cancer promotes the proliferation of cancer cells and adaptation to the tumor microenvironment through metabolic reprogramming and changes in metabolite levels (Wang et al., 2023b). Circulating metabolites have great potential as non-invasive cancer diagnostic markers. However, there is currently a lack of specific research on the association between PC and 4-hydroxychlorothalonil, and more studies are needed to verify this finding and explore its clinical application value.

The strength of our study lies in utilizing large-scale GWAS sample data from two independent sources for each outcome, corroborated by meta-analysis of positive findings from two rounds of MR analysis to assure the robustness and comprehensiveness of our results. However, several limitations should be acknowledged. Firstly, to analyze a broader range of metabolites, a significance threshold of *p* < 1 × 10^−5^ was applied to the metabolomic GWAS data, while ensuring robustness by excluding weak IVs (F-statistic >10 for each SNP). Secondly, the study predominantly included individuals of European descent, limiting generalizability to other ethnicities. Thirdly, while MR analysis revealed some metabolites with causal links to urological malignancies, the very low effect sizes limit their potential application as biomarkers, particularly concerning TC. Fourthly, the sample size differences for the four urological cancers from UK Biobank and FinnGen consortium may potentially impact the causal inference from the meta-analysis. Future research should aim to expand existing GWAS databases and conduct analyses on more balanced datasets to further validate and strengthen our findings. Fifthly, the scarcity of metabolites identified in relation to outcomes precluded performing metabolic pathway enrichment analyses to delve into regulatory mechanisms and biological processes. Sixthly, the reverse MR analysis focused on meaningfully discovered metabolites and did not comprehensively study all metabolites for potential reverse causal effects. Lastly, in this MR study, we conducted a preliminary exploratory analysis aimed at uncovering novel biological insights. We opted to use the WM method (P_WM_ < 0.05) for a relatively lenient correction rather than applying more stringent Bonferroni and false discovery rate (FDR) corrections. This decision was made in order to retain a greater number of potential findings. Future research should seek to validate our findings through randomized controlled trials and further investigate the specific roles these metabolites play in the etiology of urological malignancies.

## 5 Conclusion

This two-sample MR study revealed the significant role of blood metabolites in four urological malignancies (BC, KC, PC, and TC). The investigation identified 31 metabolites exhibiting significant causal associations with the four urological cancers and 37 metabolites showing suggestive causal relationships. Furthermore, a significant causal association was discovered between genetically predicted PC and elevated levels of 4-hydroxychlorothalonil. In summary, this study offers new insights into the metabolic features of urological cancers, providing potential biomarkers and molecular targets for early diagnosis and therapeutic research in cancer. These findings pave the way for future explorations in targeted interventions, fostering advancements in research and treatment development within the urological cancer field.

## Data Availability

The original contributions presented in the study are included in the article/Supplementary Material, further inquiries can be directed to the corresponding author.
