# Supplementary material for: Causality of genetically determined metabolites on susceptibility to prevalent urological cancers: a two-sample Mendelian randomization study and meta-analysis
Source: Front Genet. 2024 Jul 1;15:1398165. doi: 10.3389/fgene.2024.1398165 (PMC11246892; doi:10.3389/fgene.2024.1398165)
Supplement: Supplementary file 2 [file Table3.DOCX]

**Additional file 3: Leave-one-out analyses and funnel plots of potential causality in preliminary MR analysis**.

Bladder Cancer
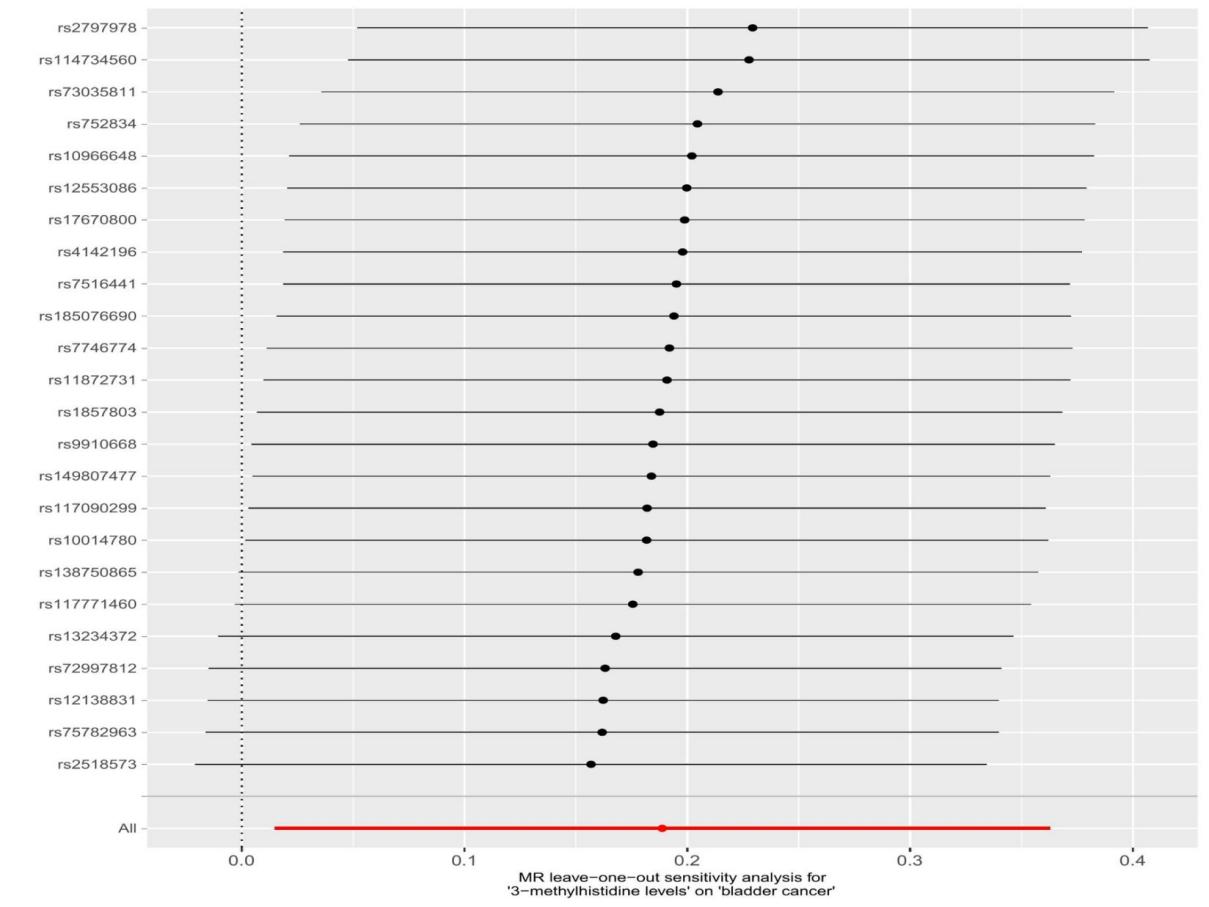


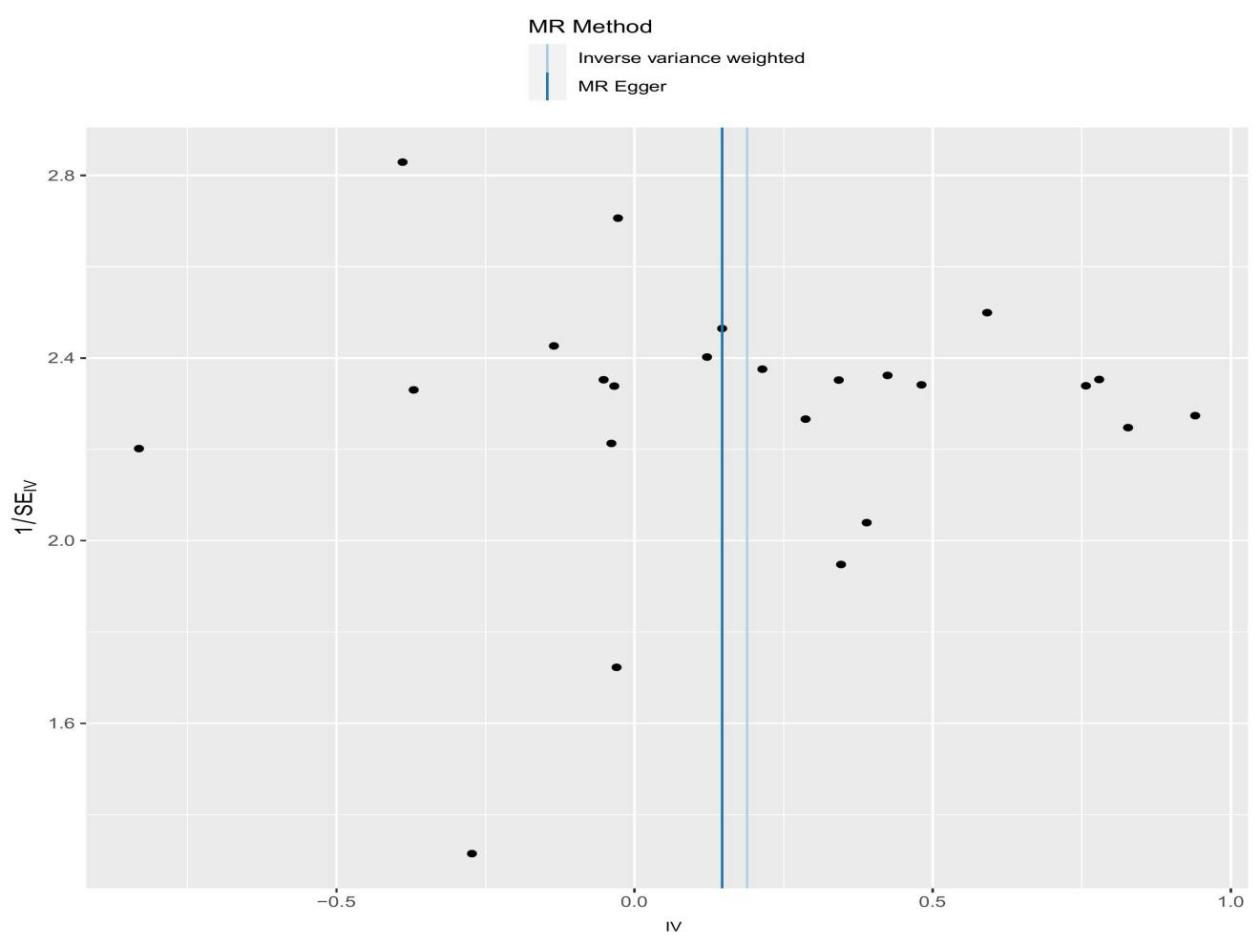


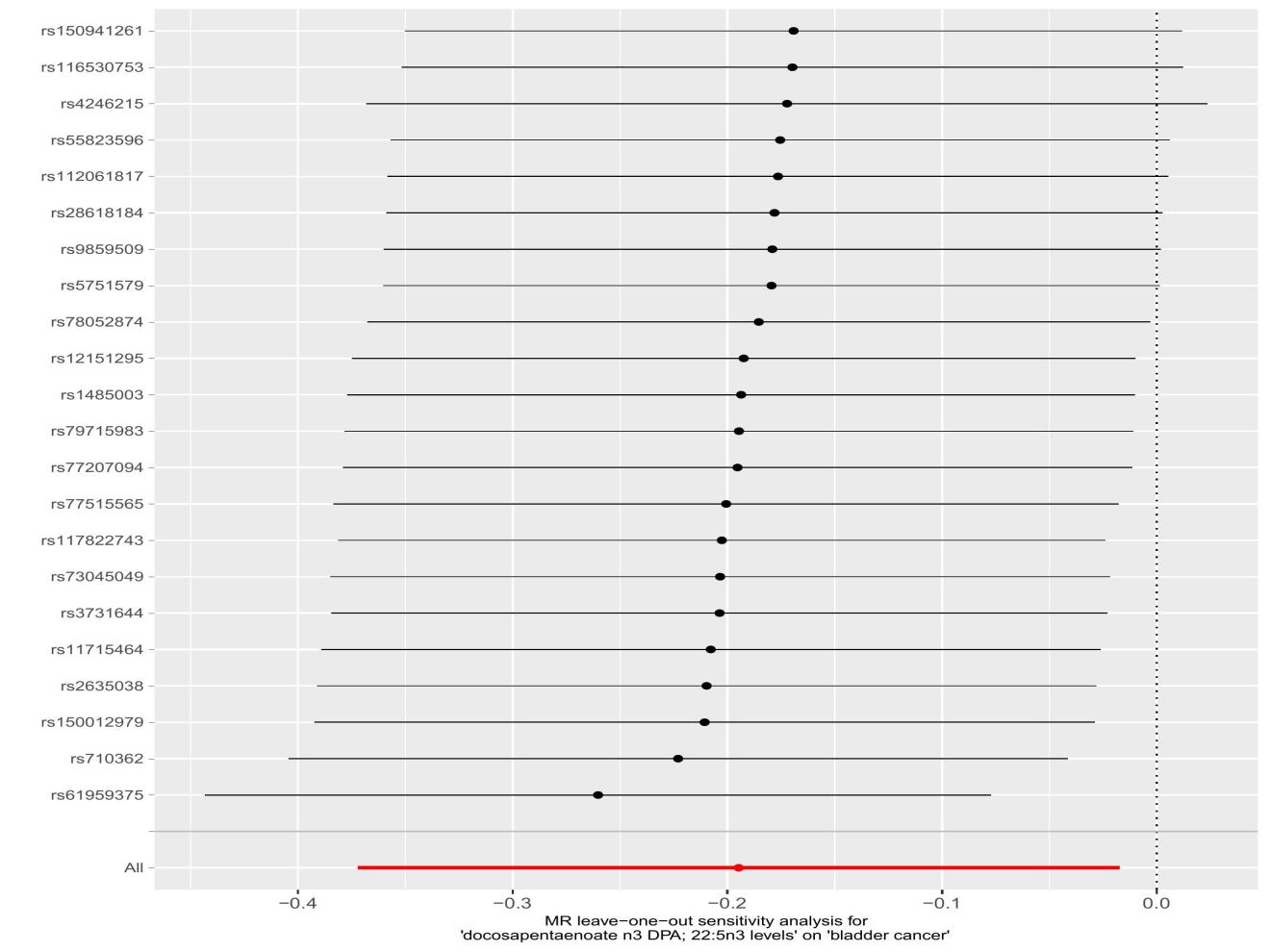


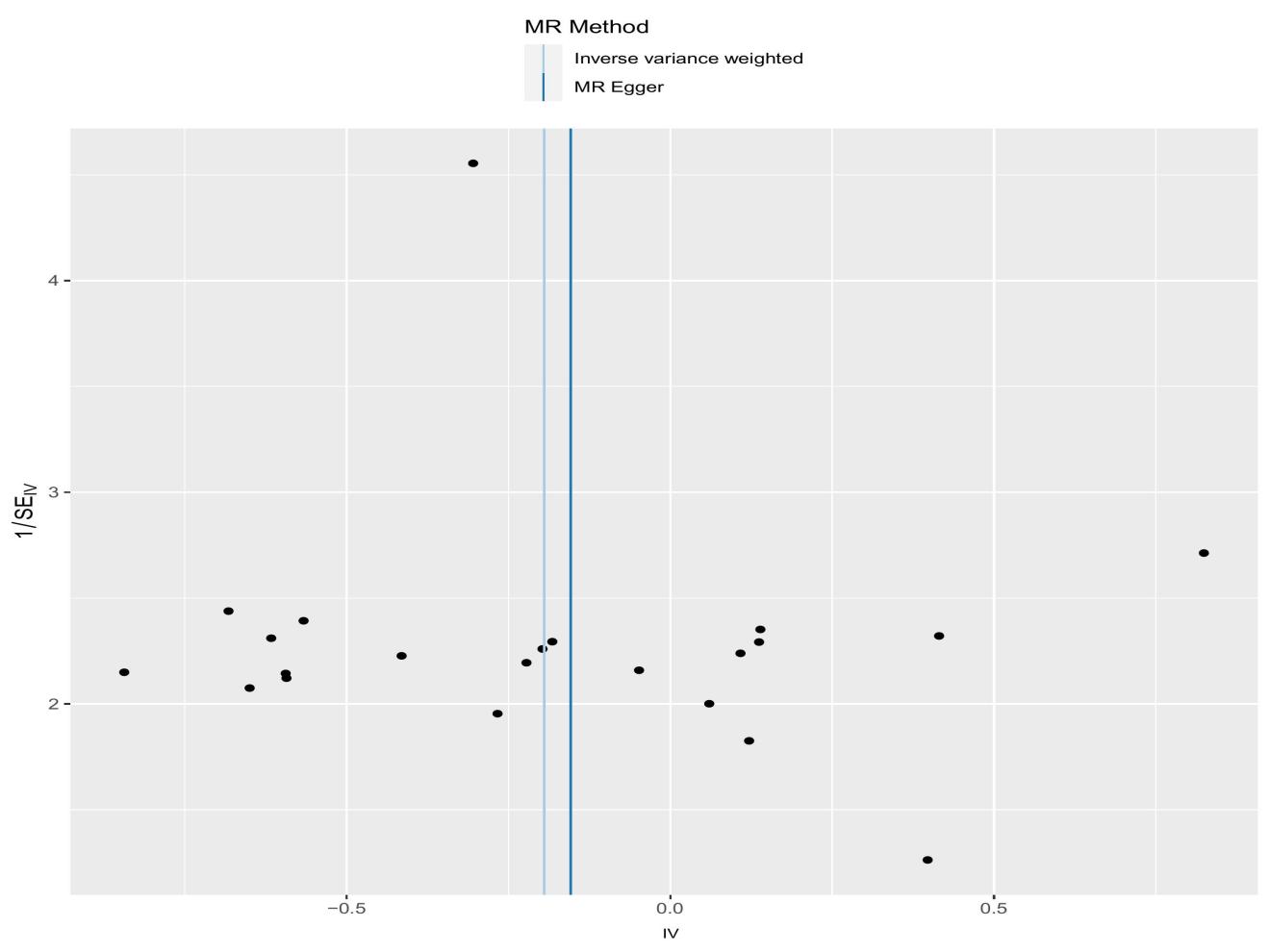


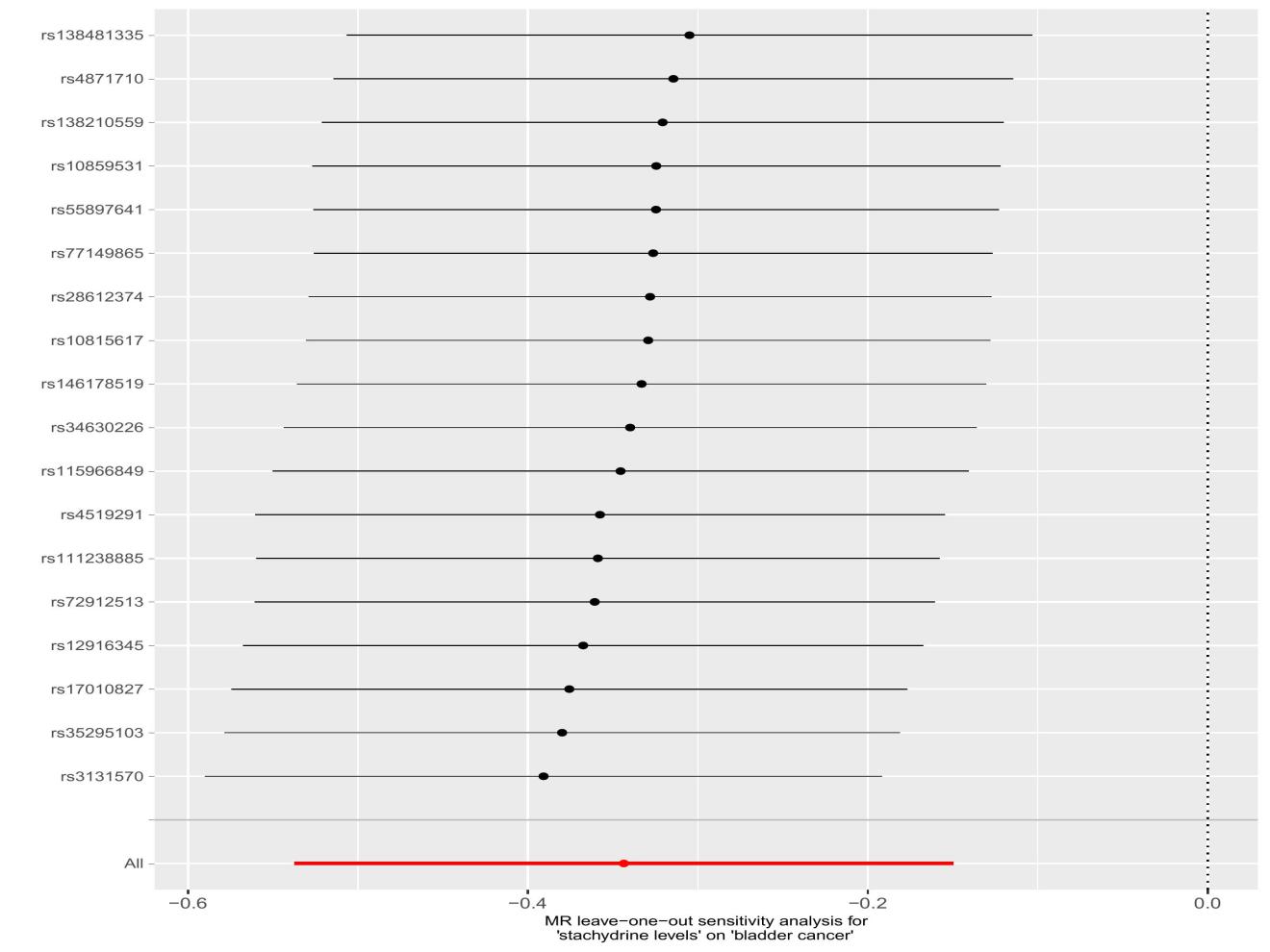


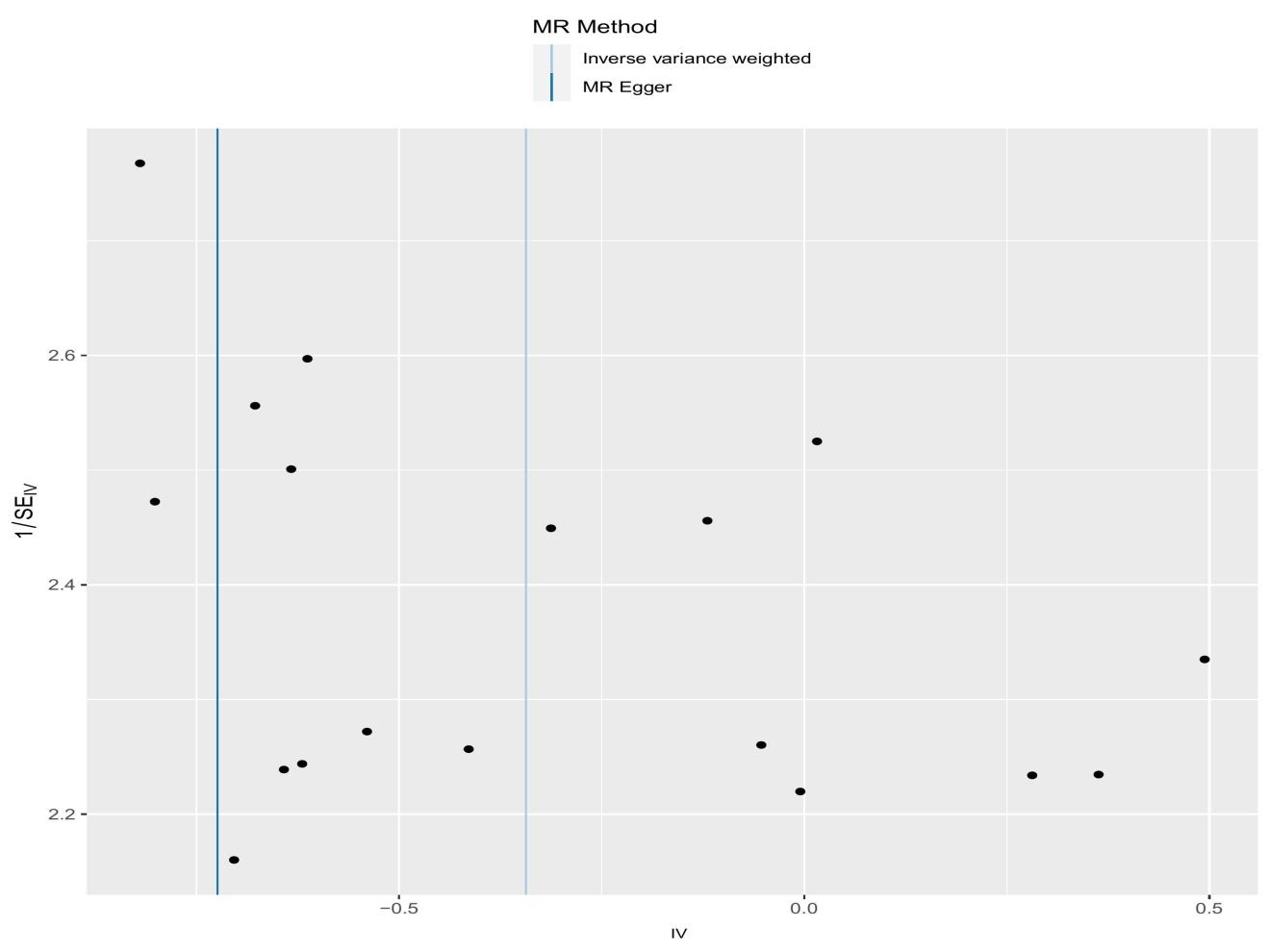


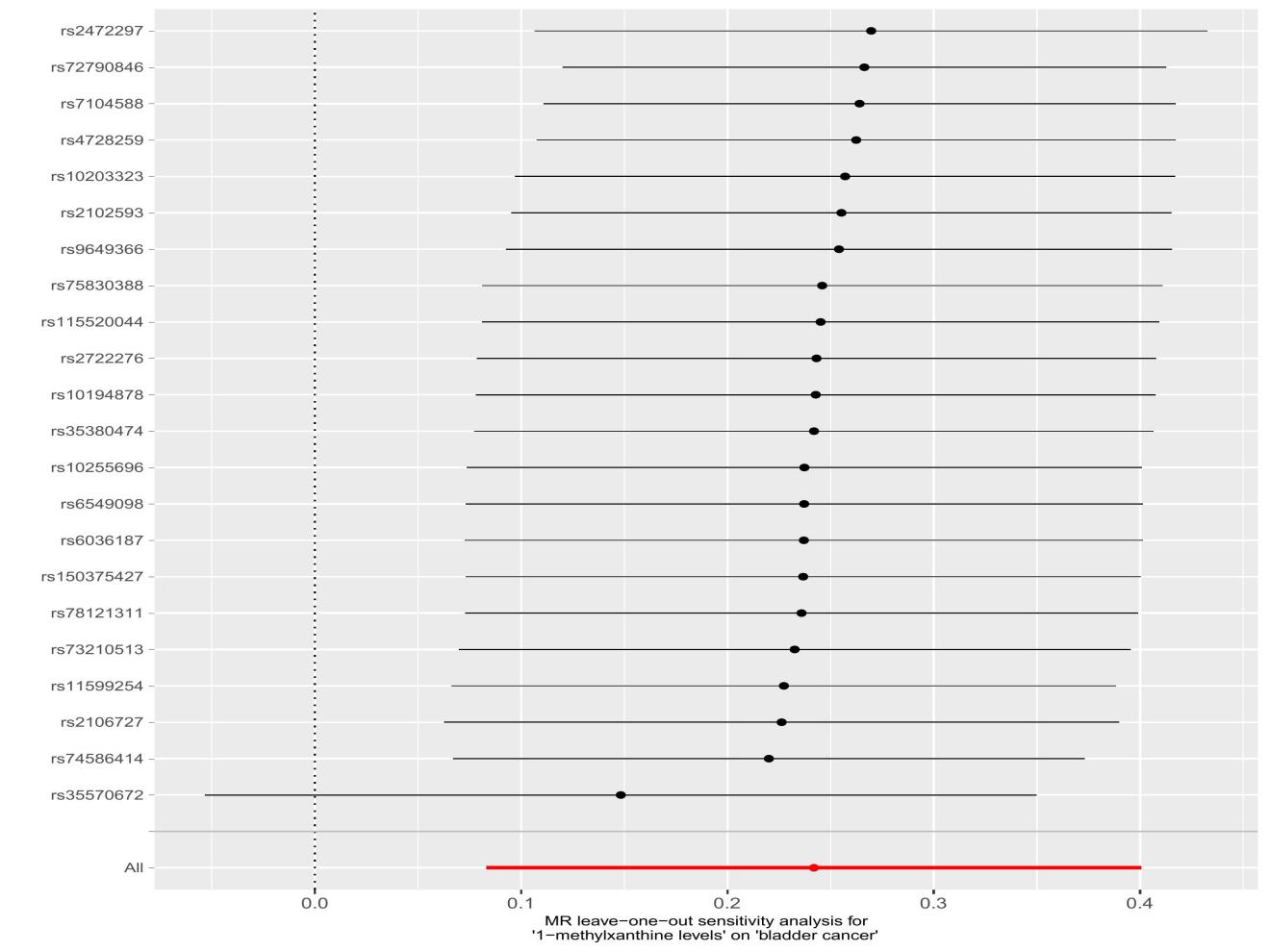


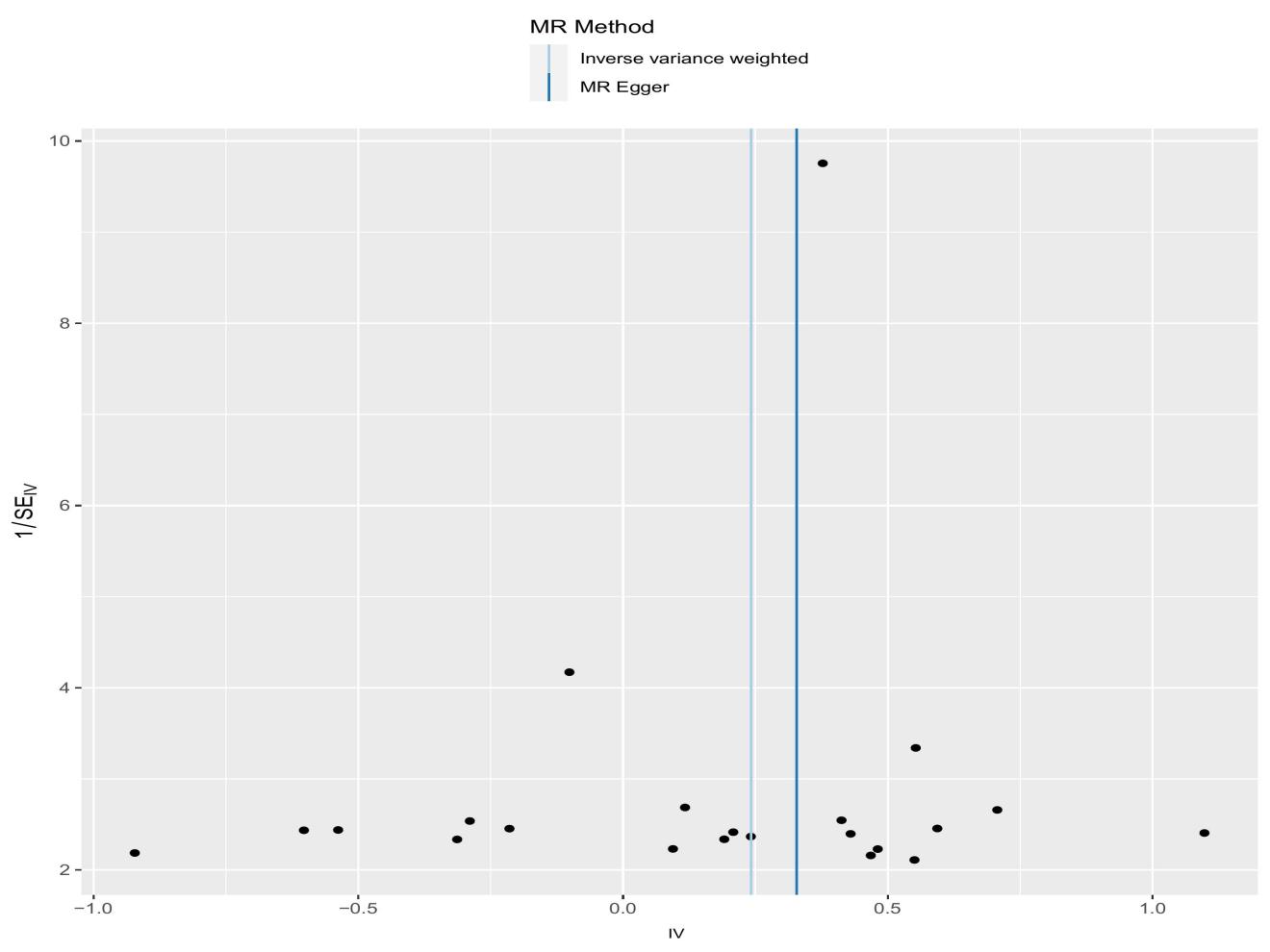


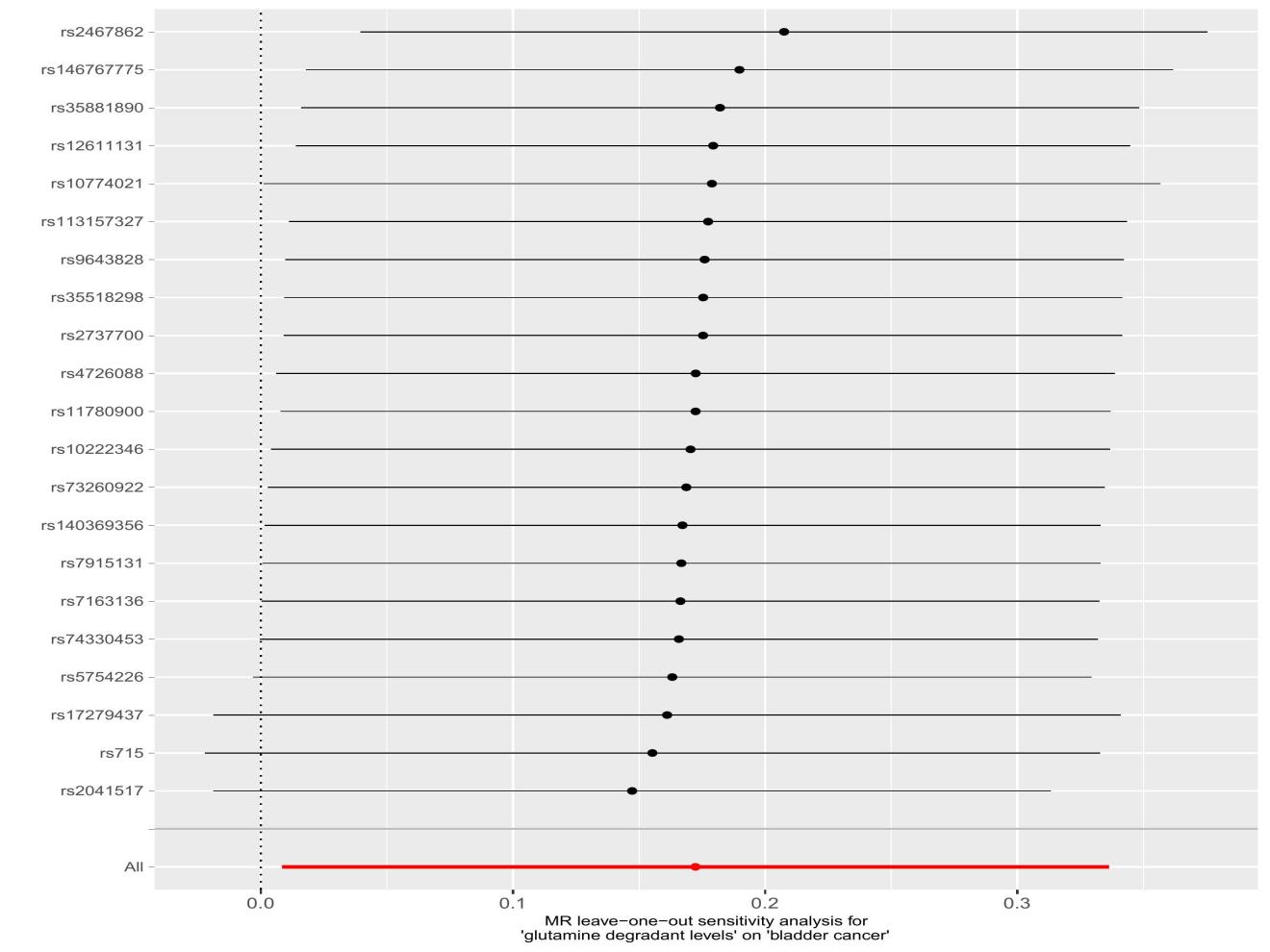


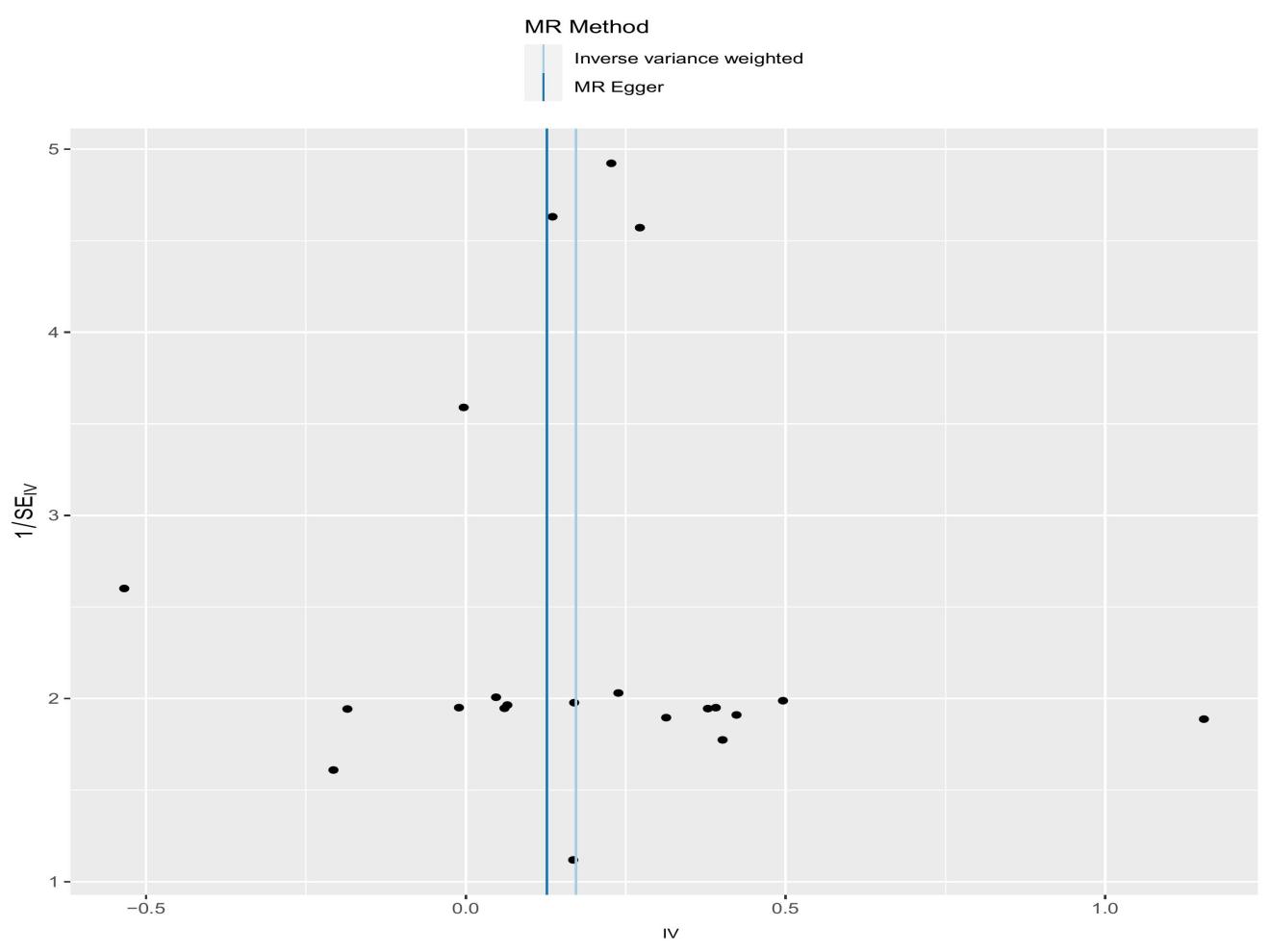


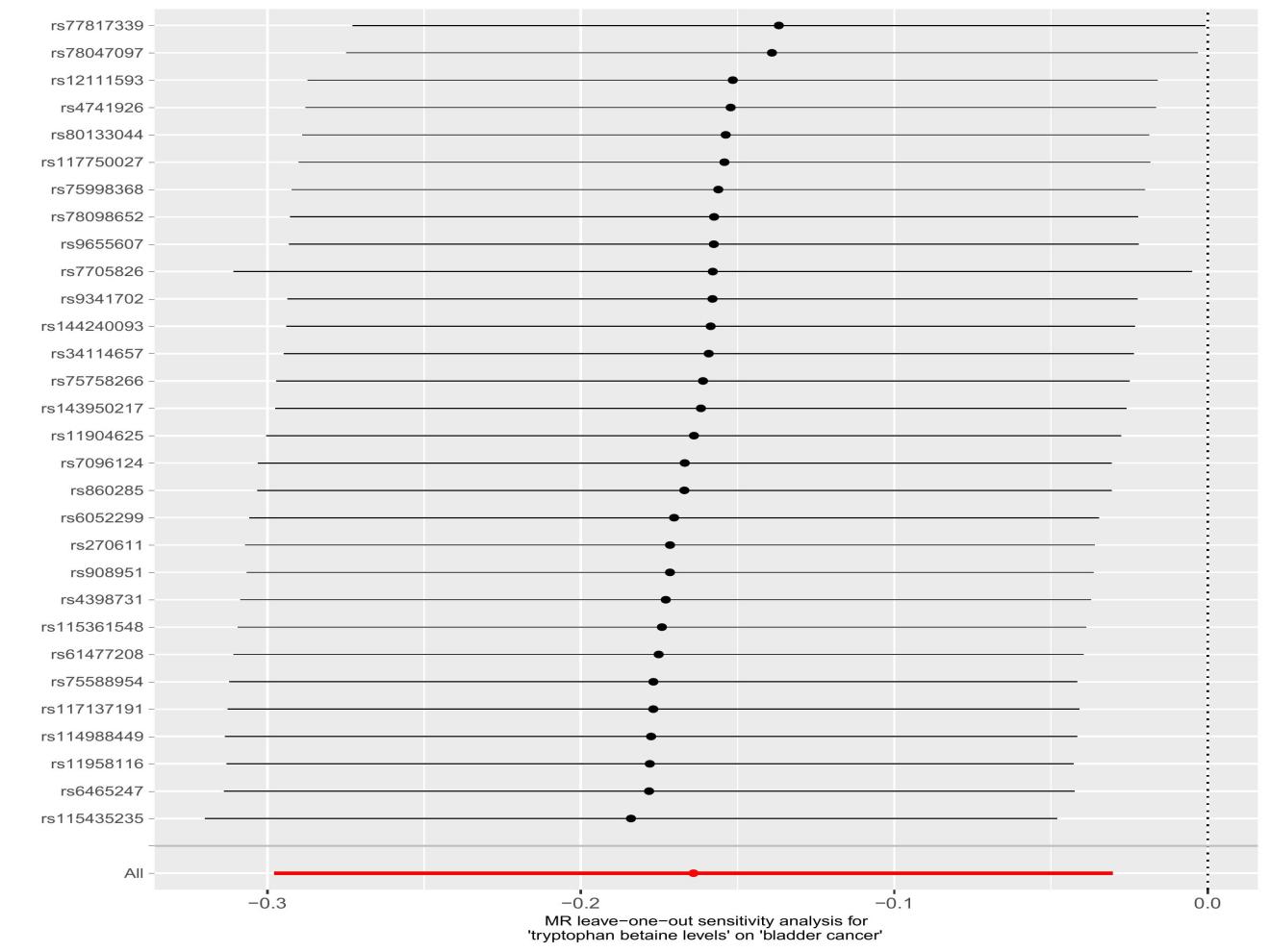


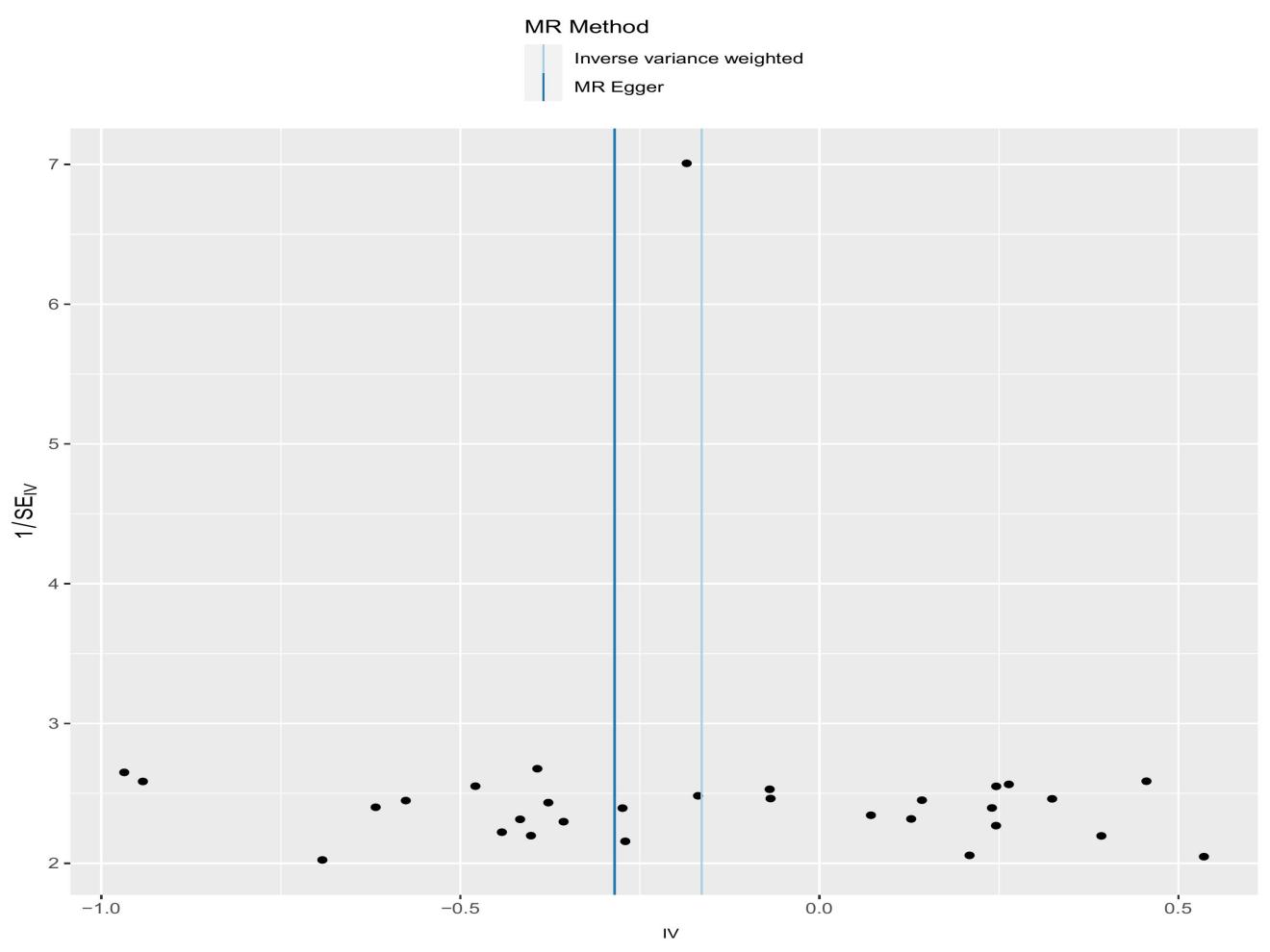


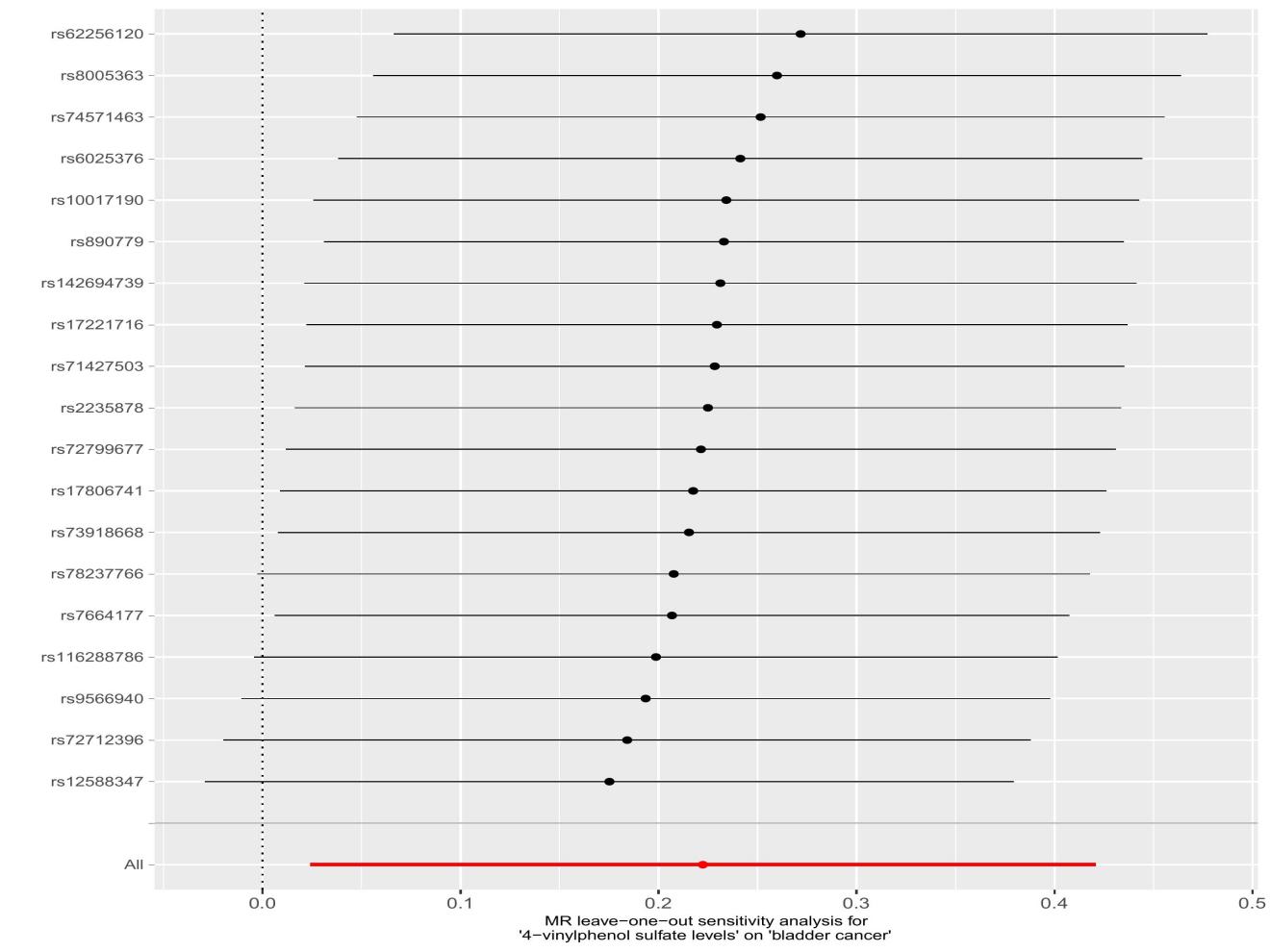


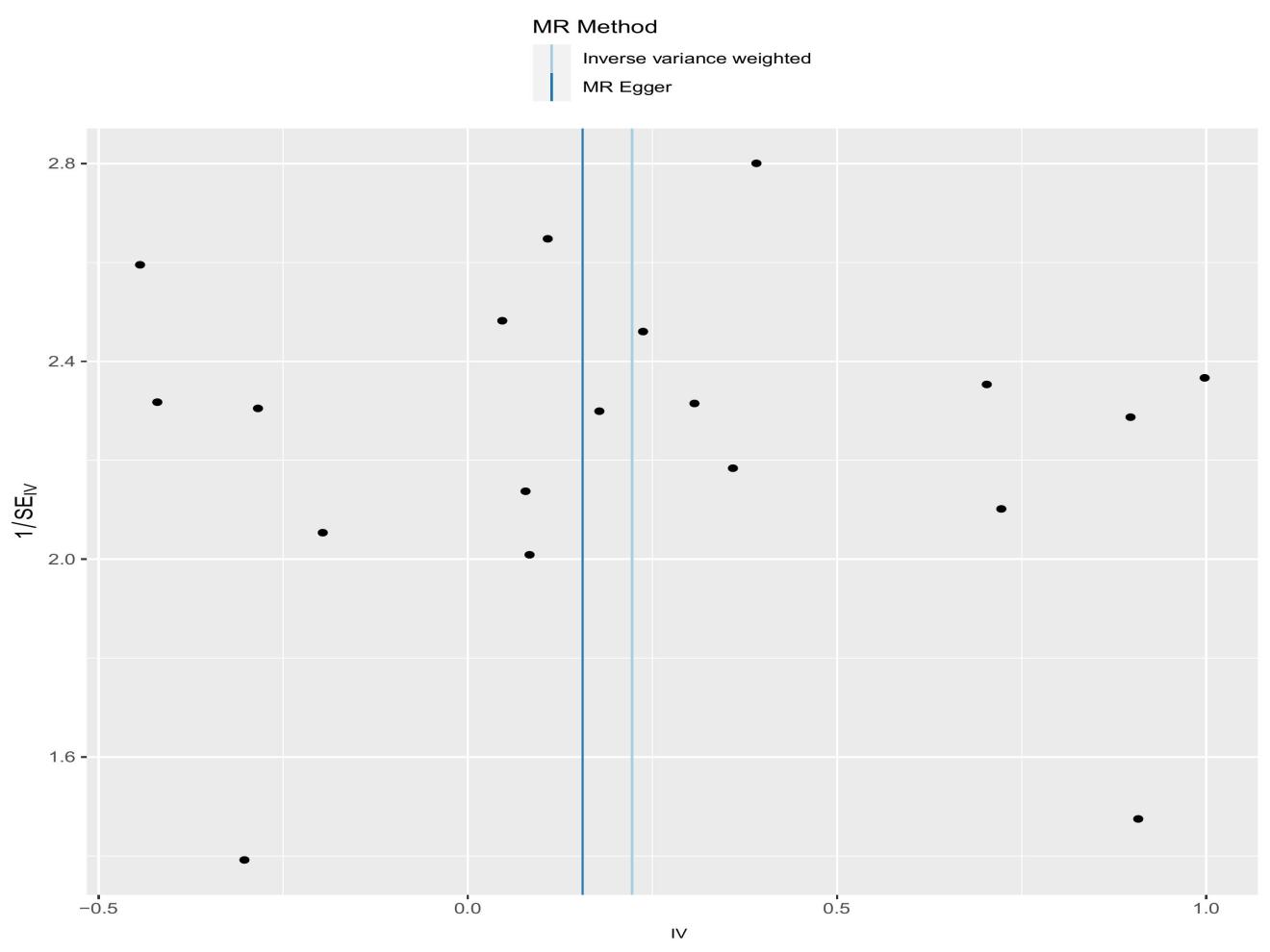


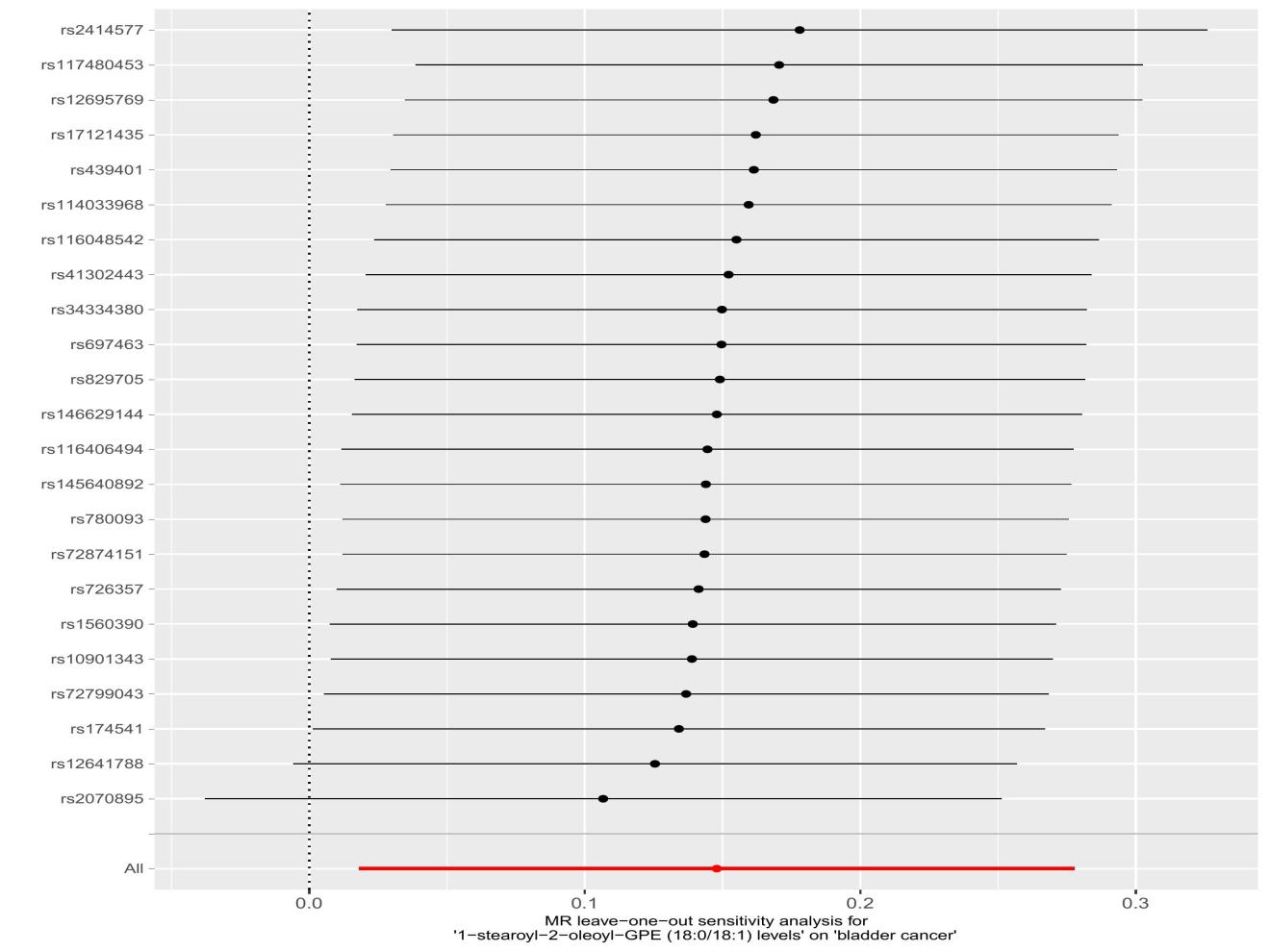


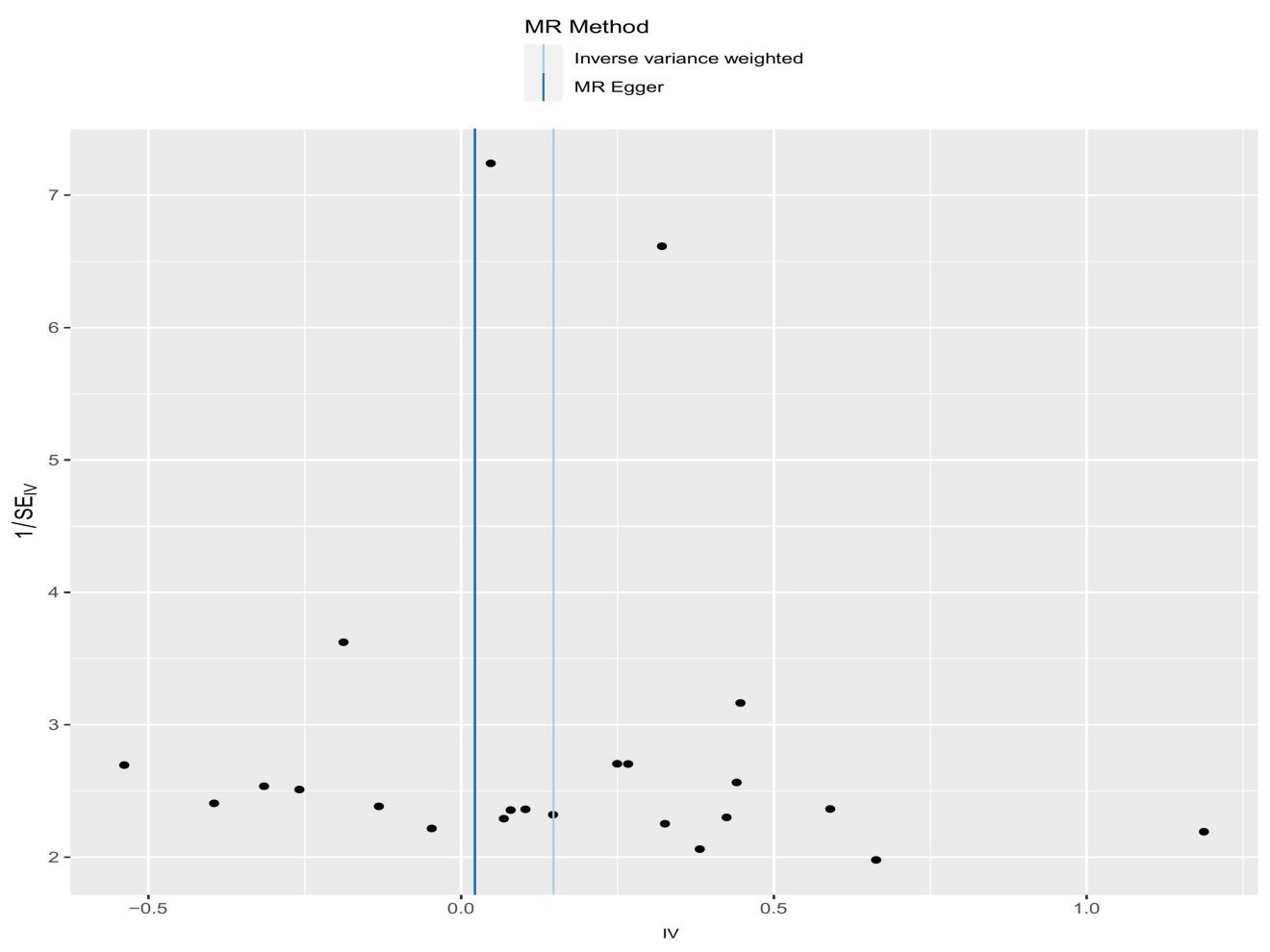


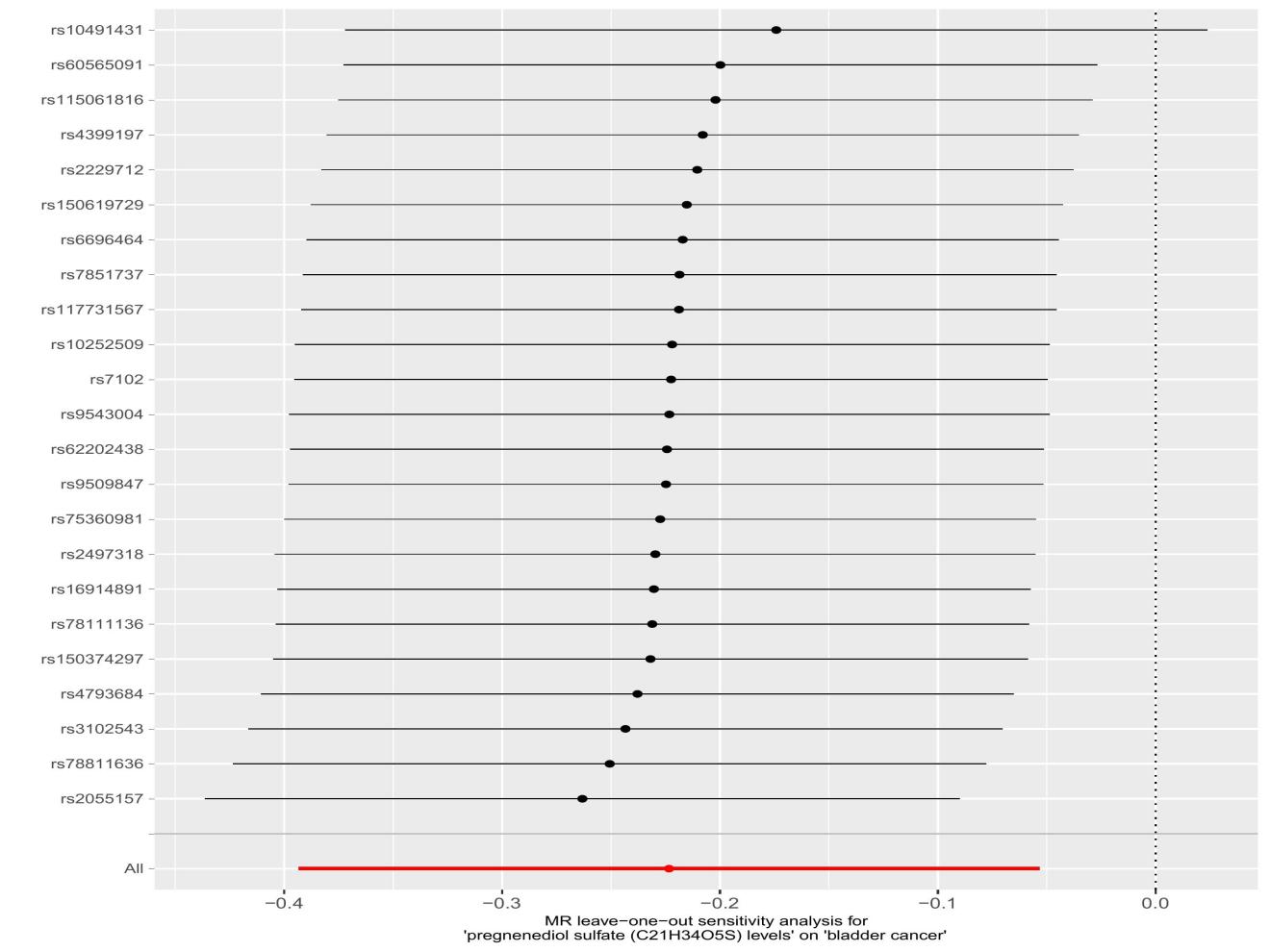


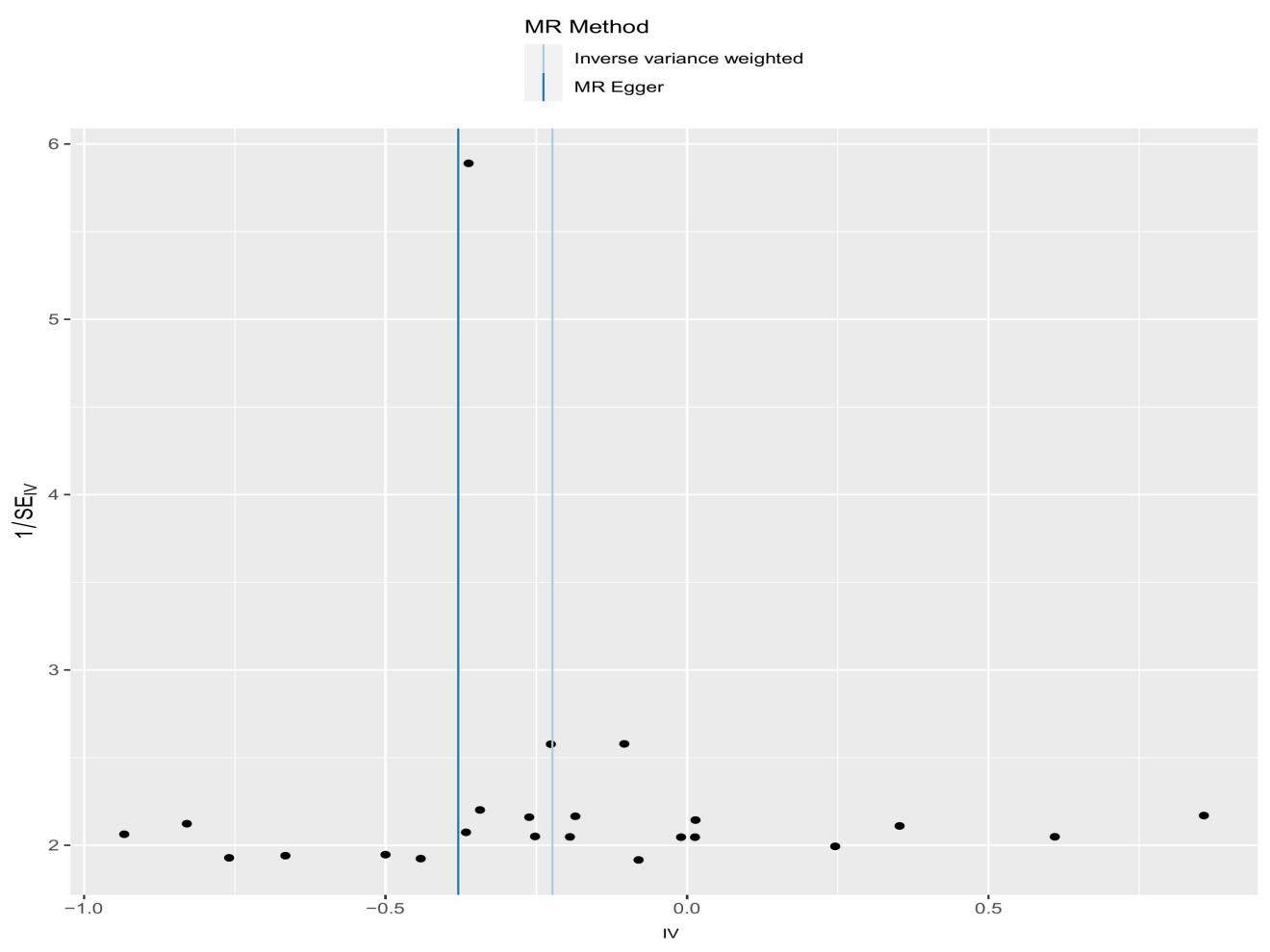


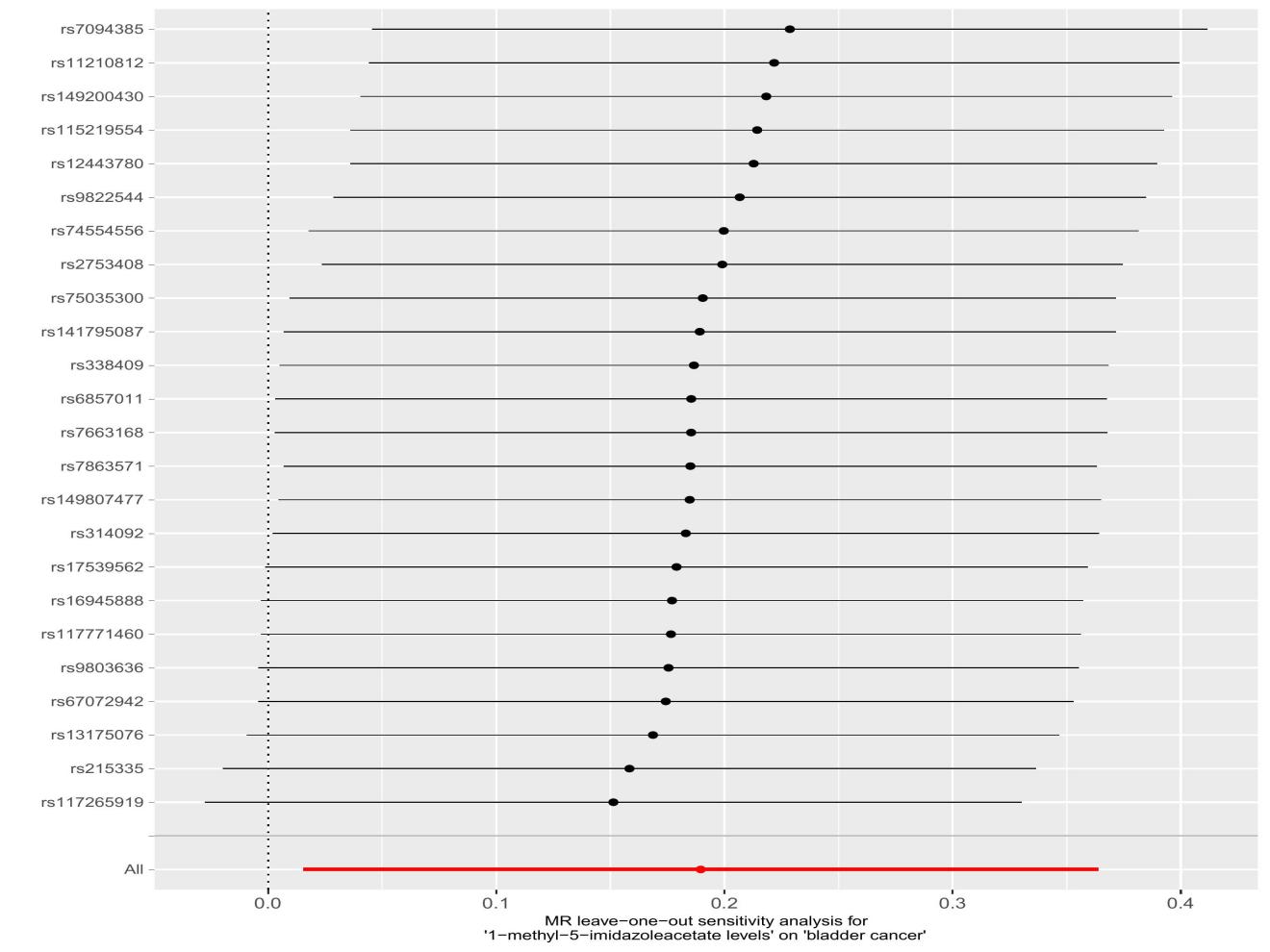


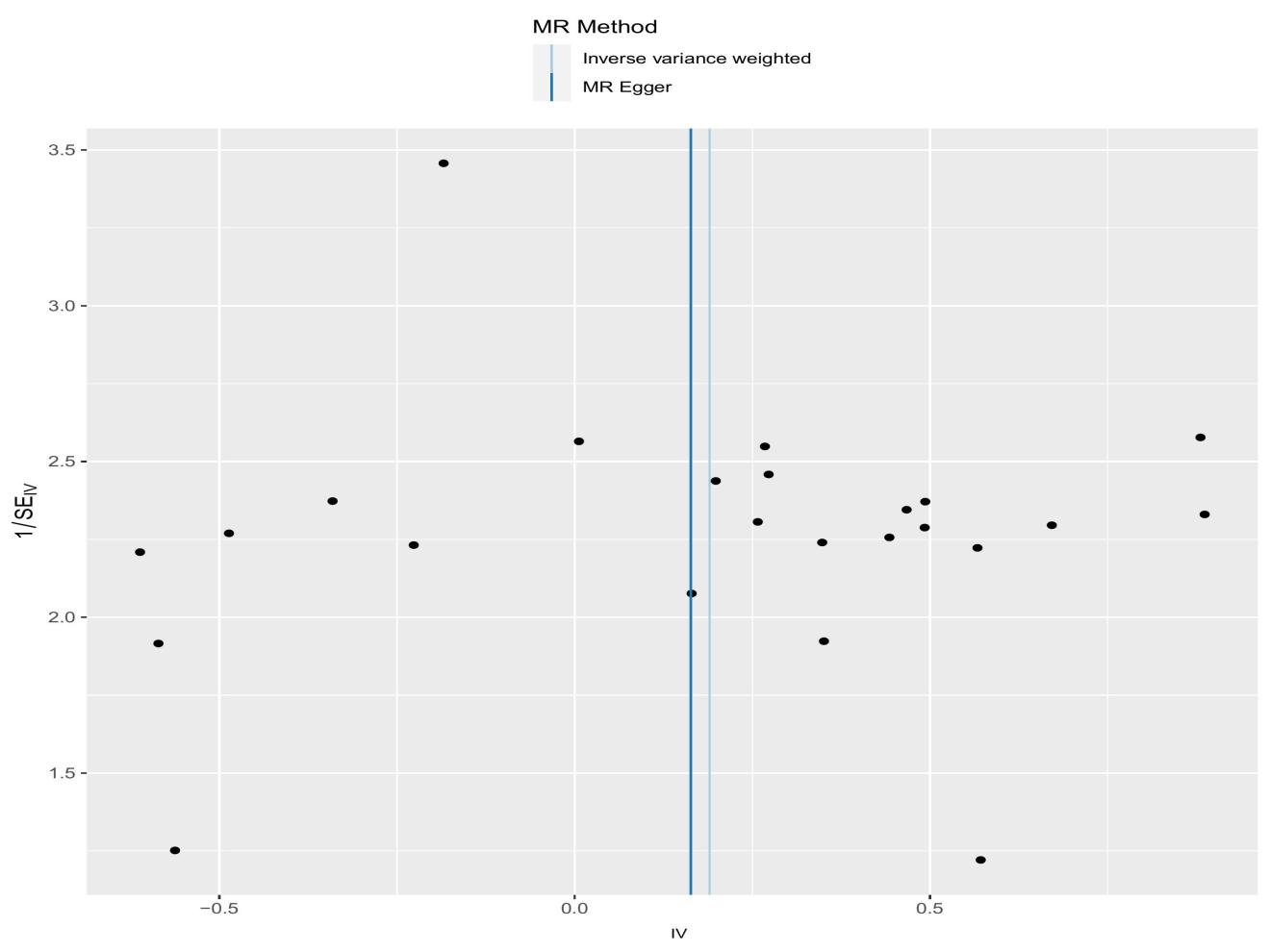


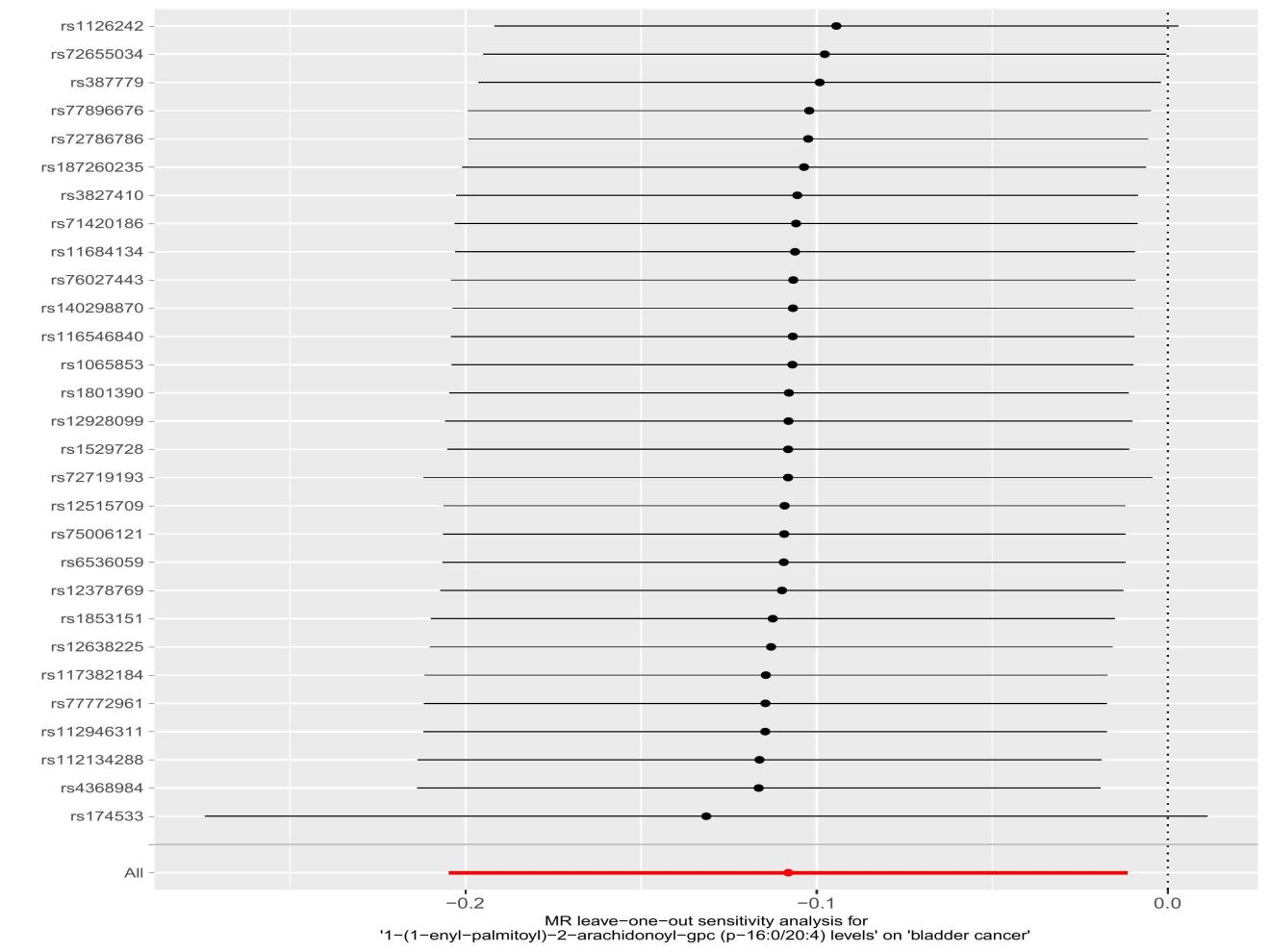


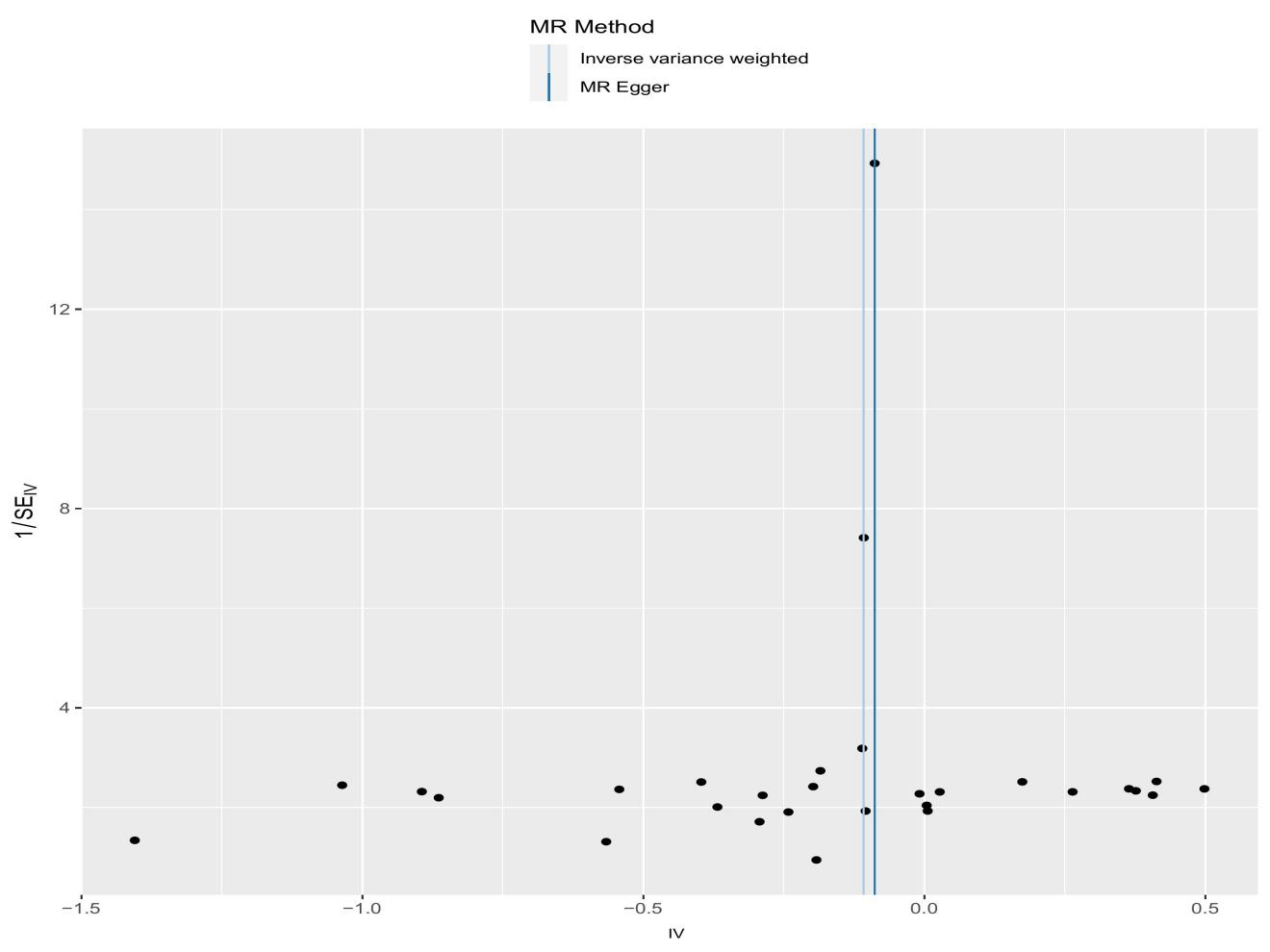


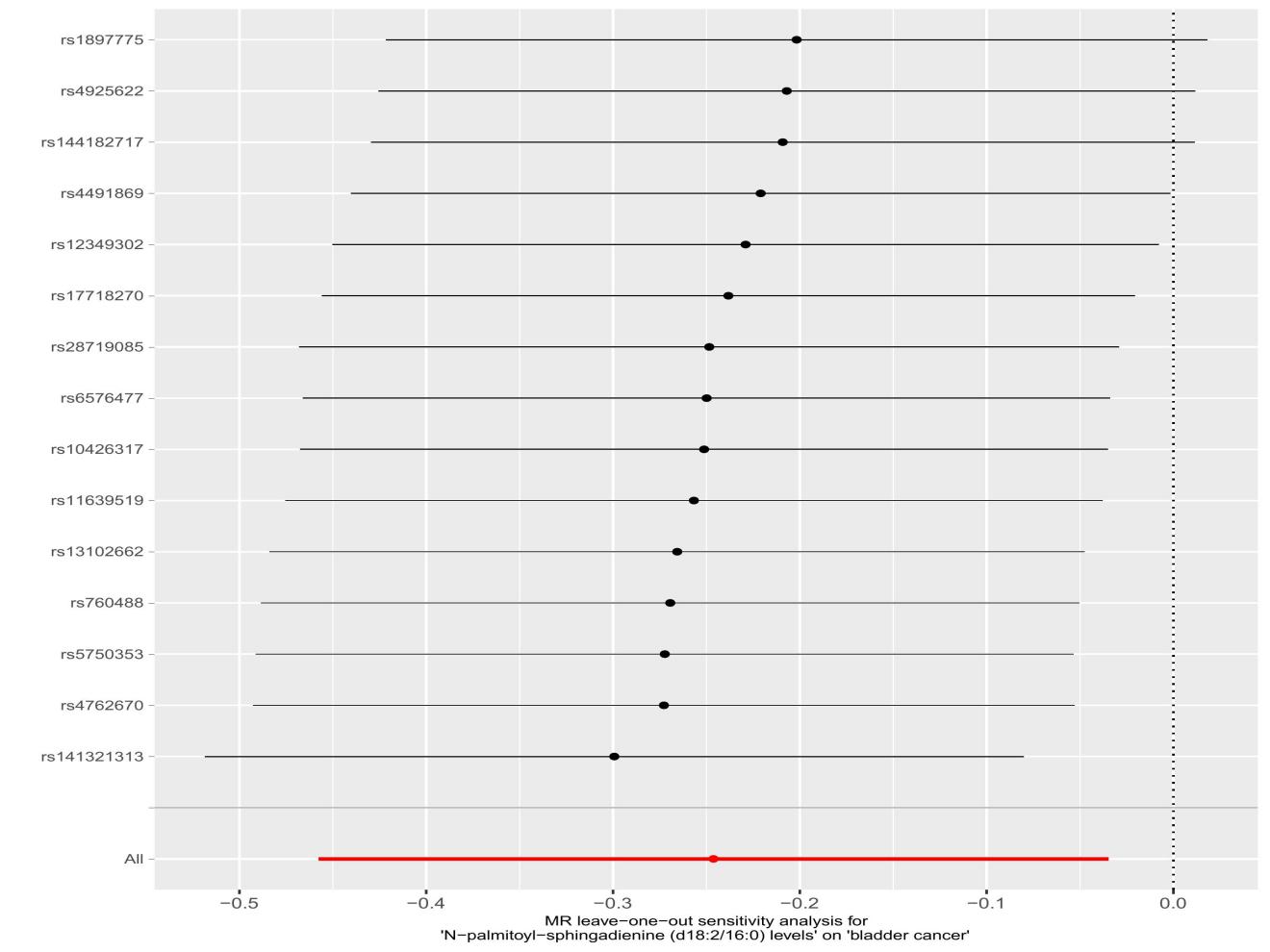


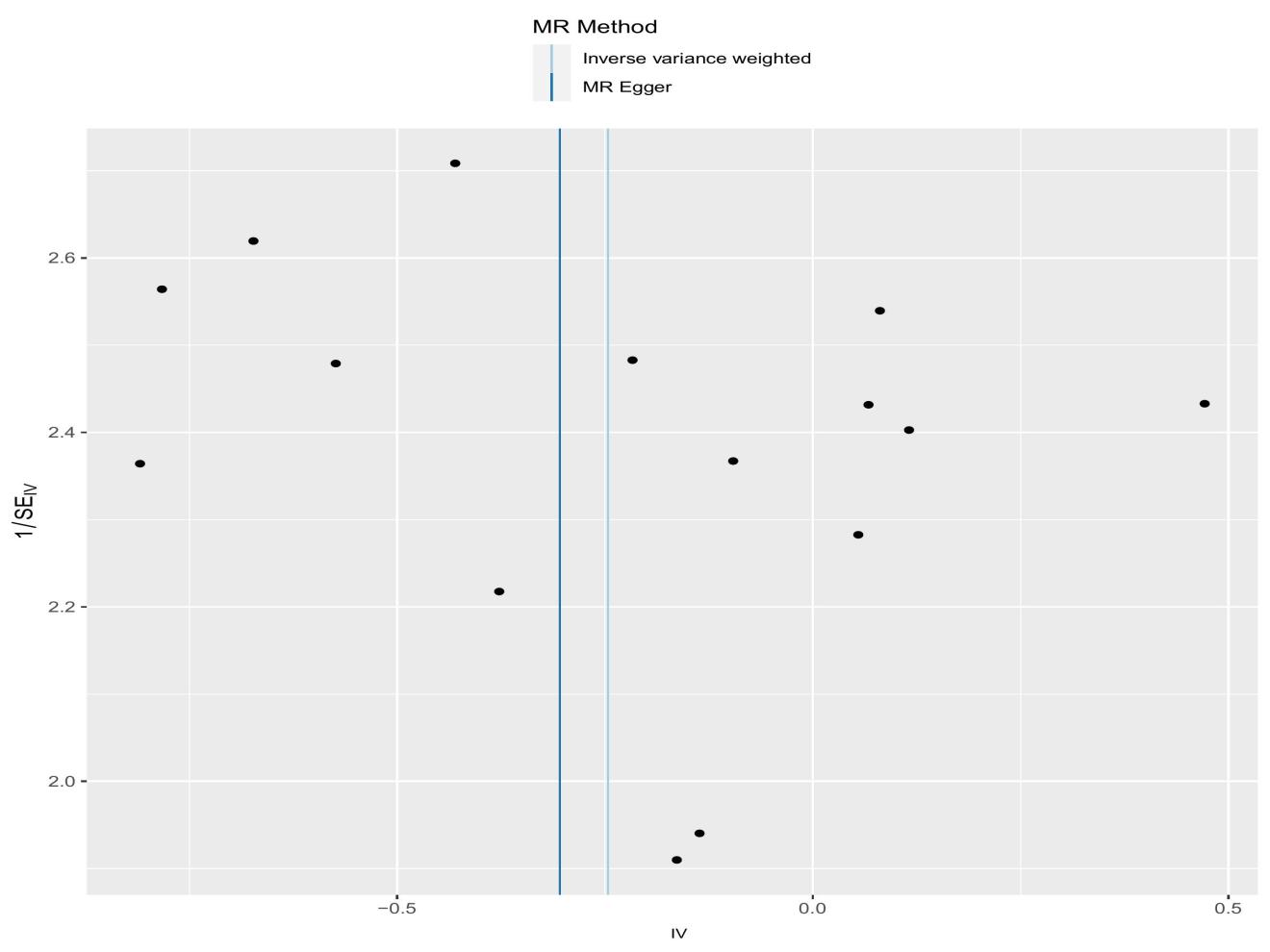


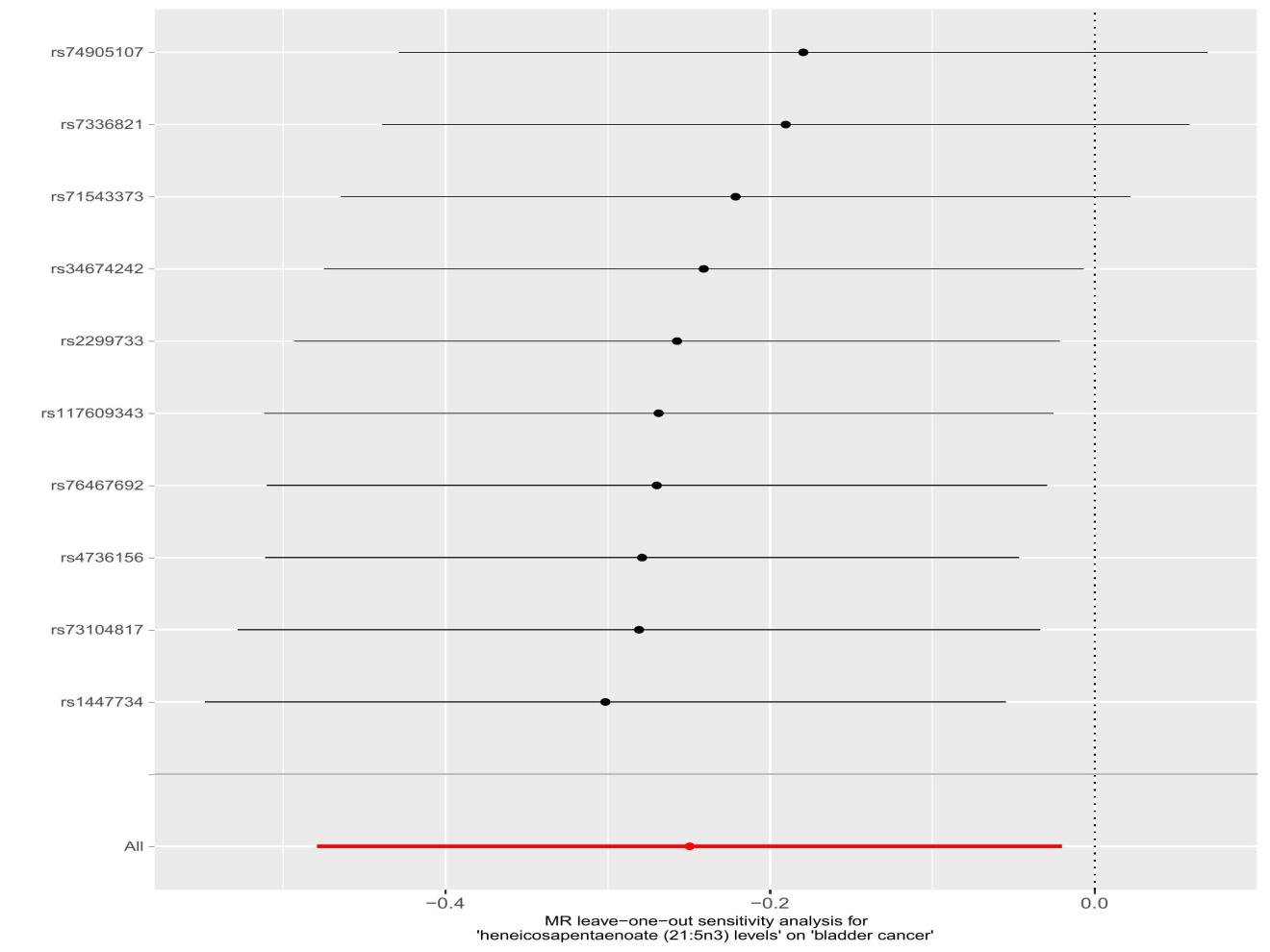


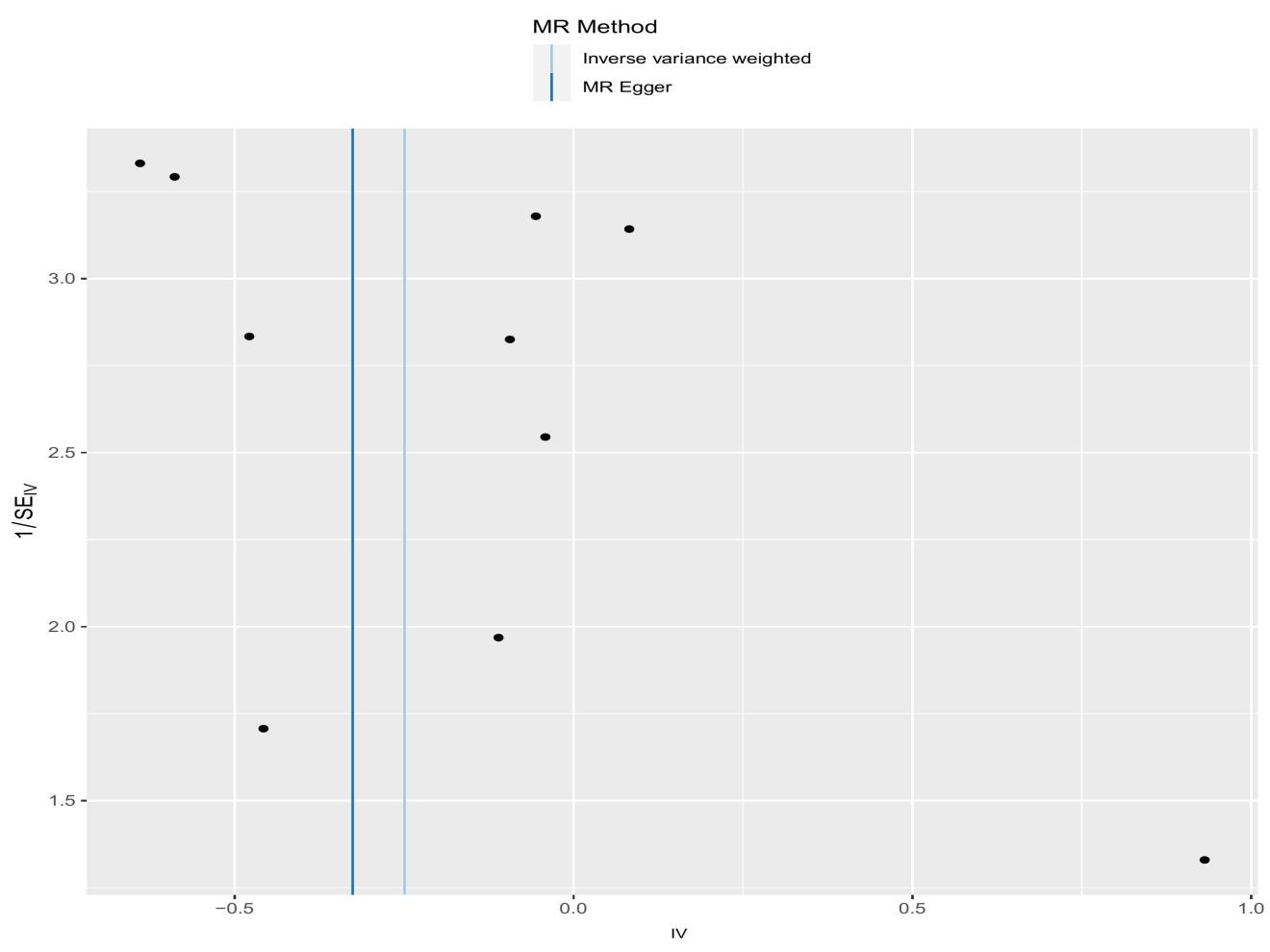


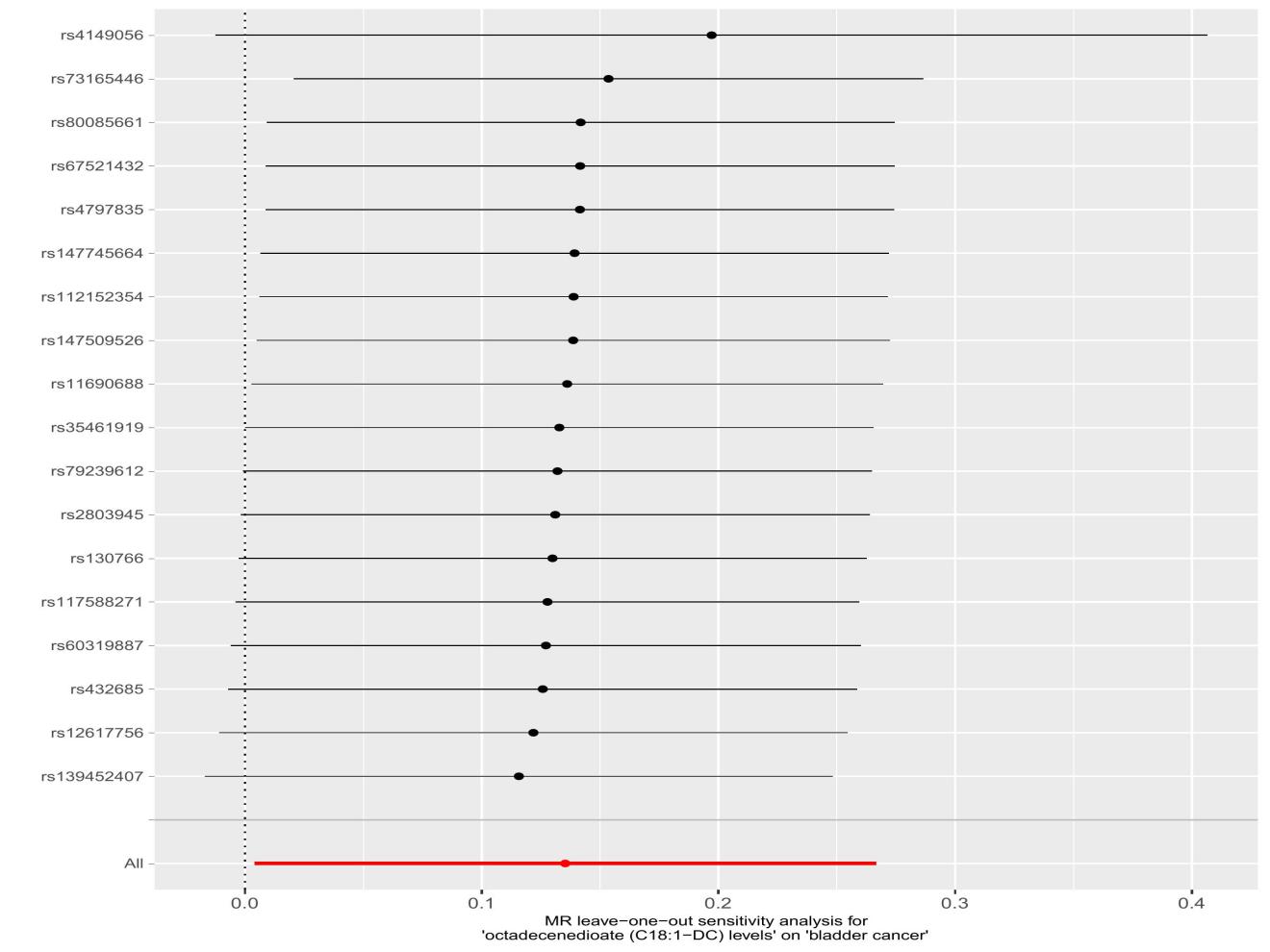


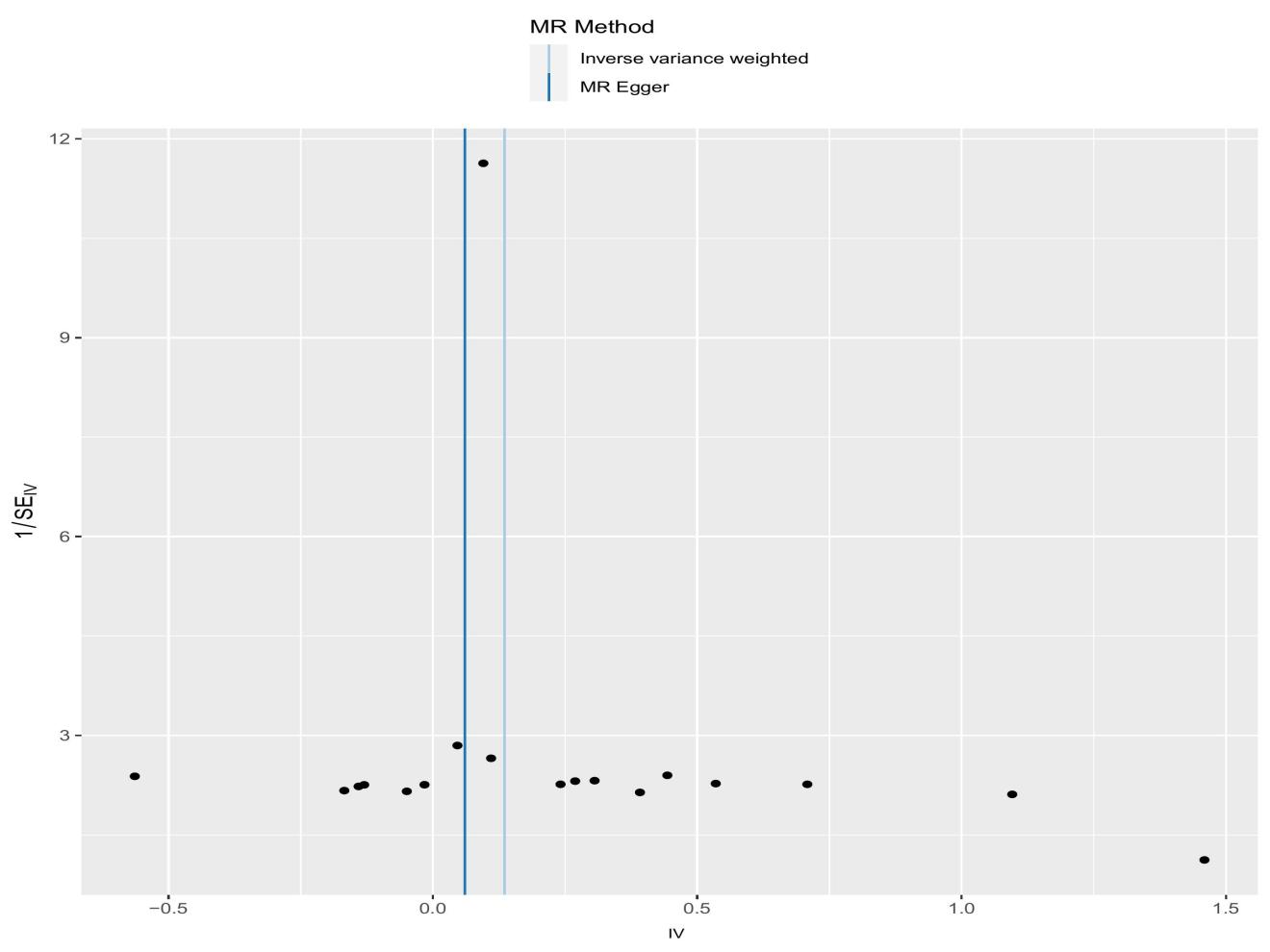


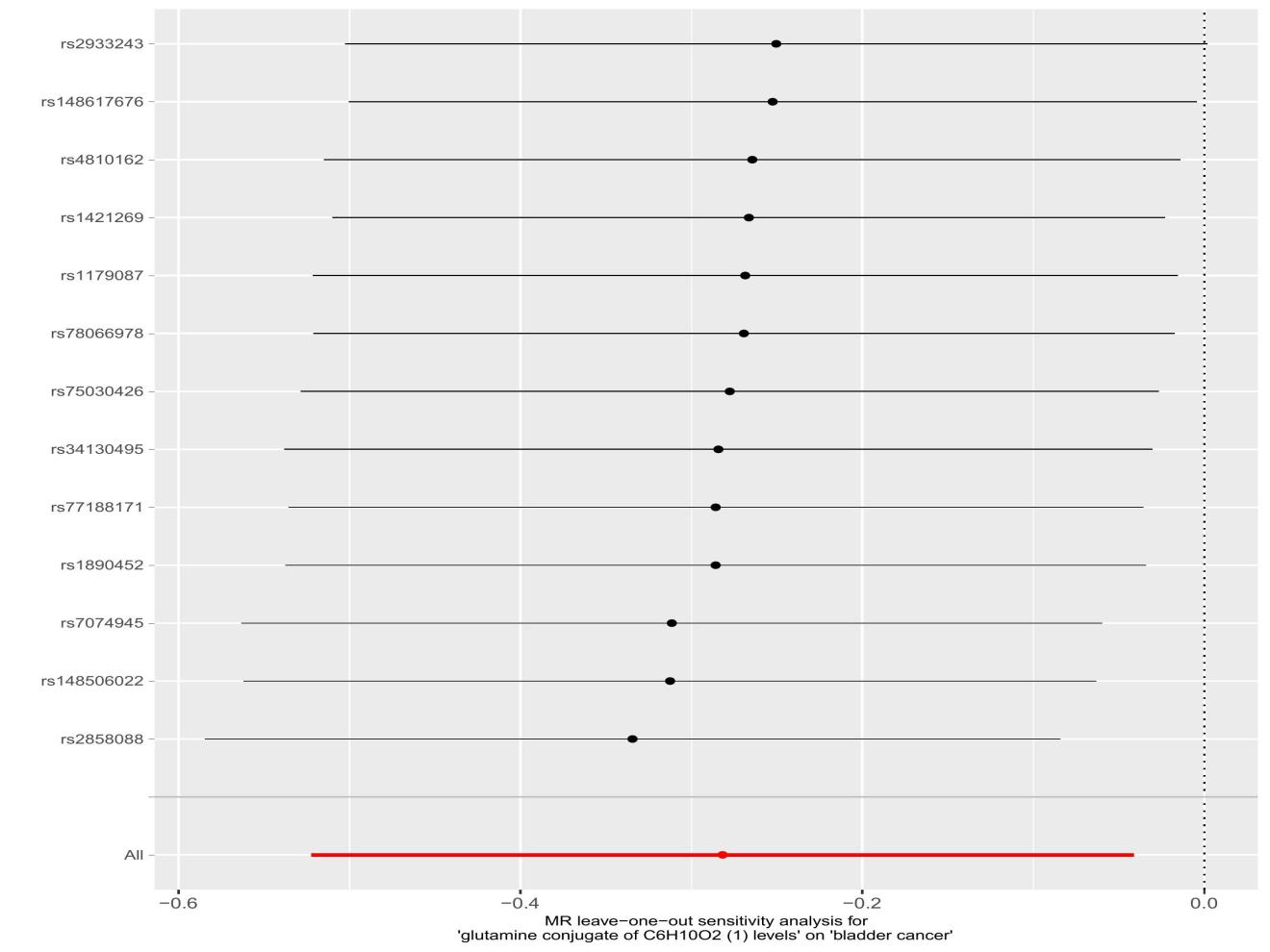


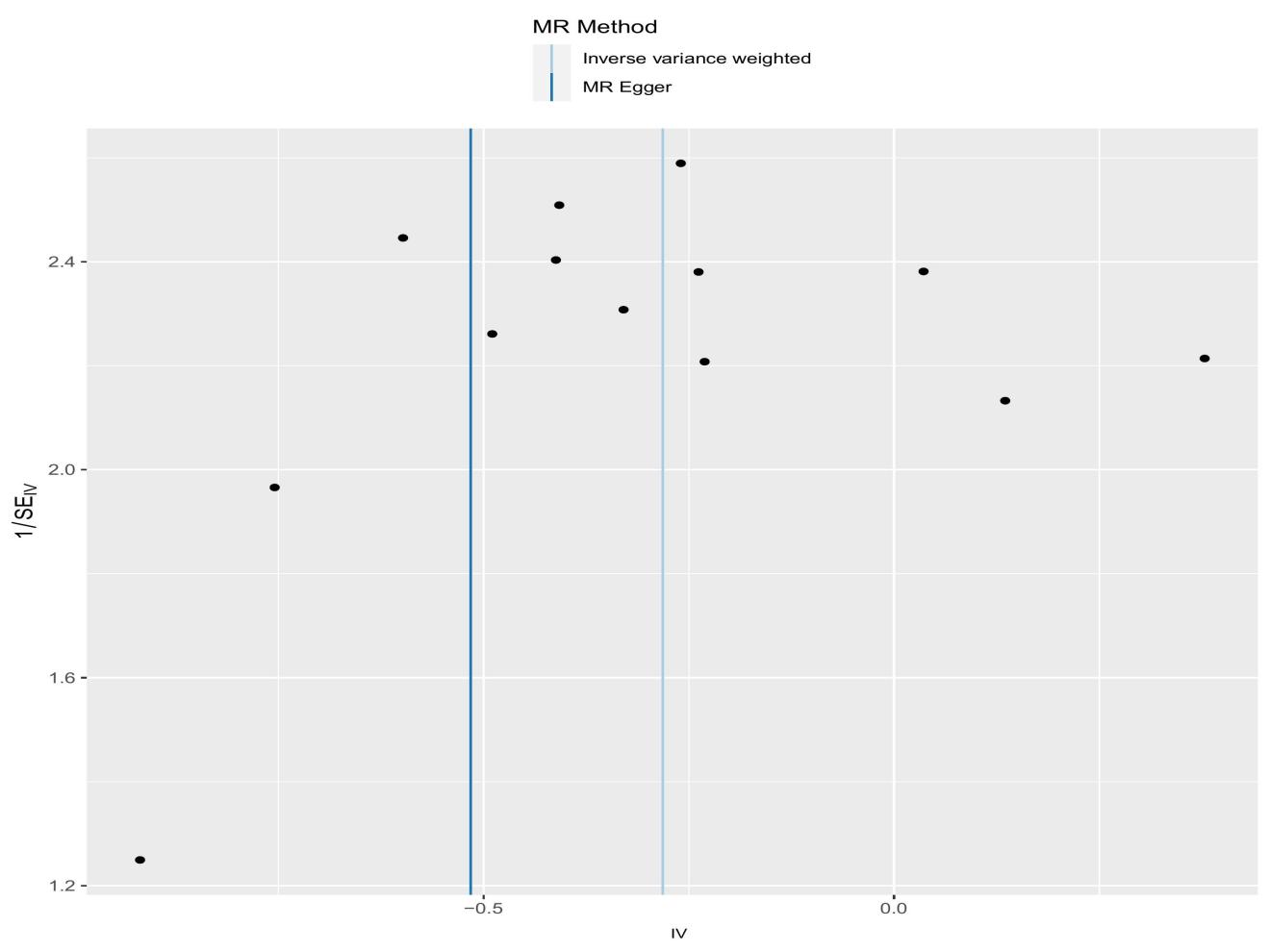


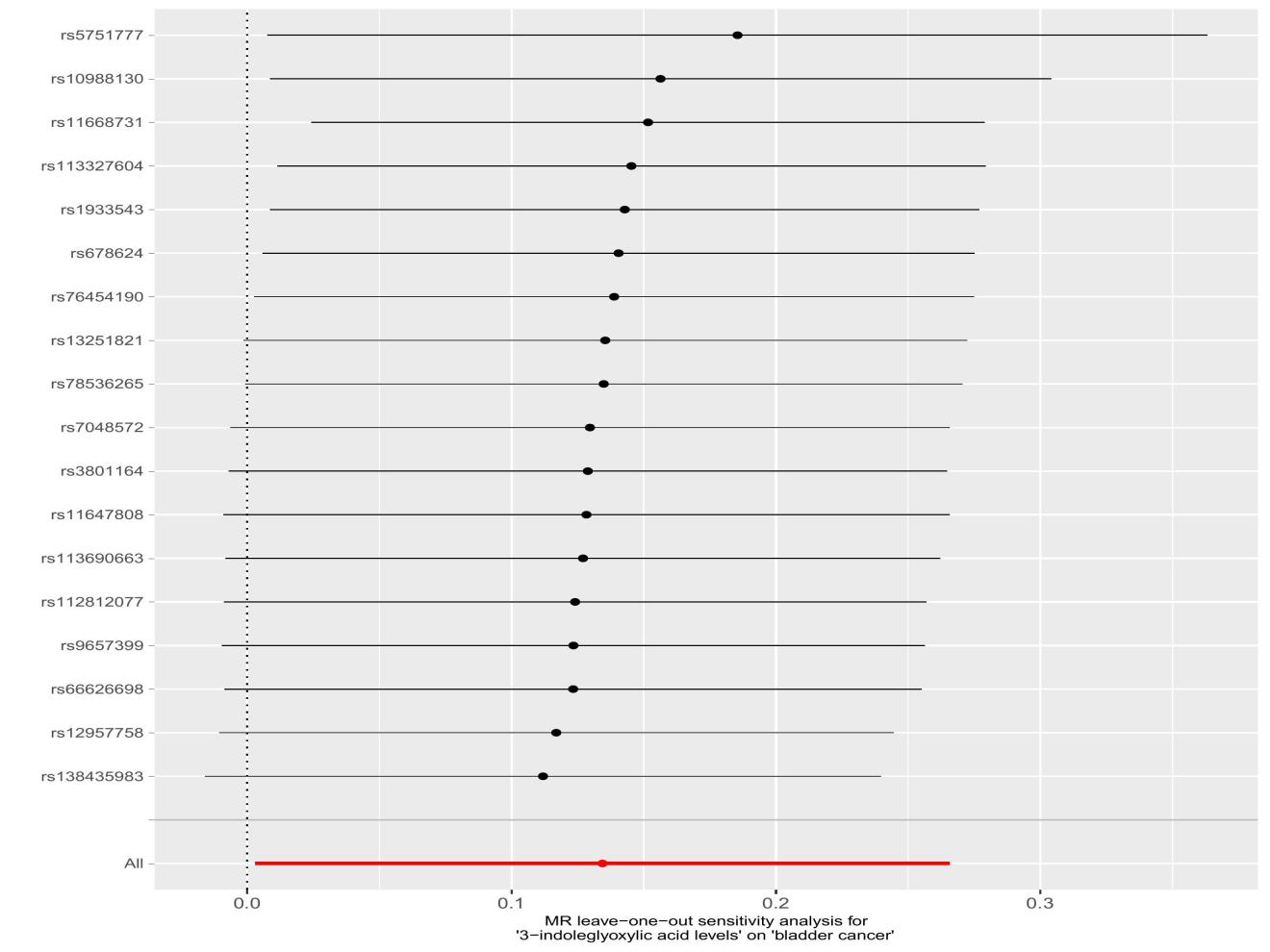


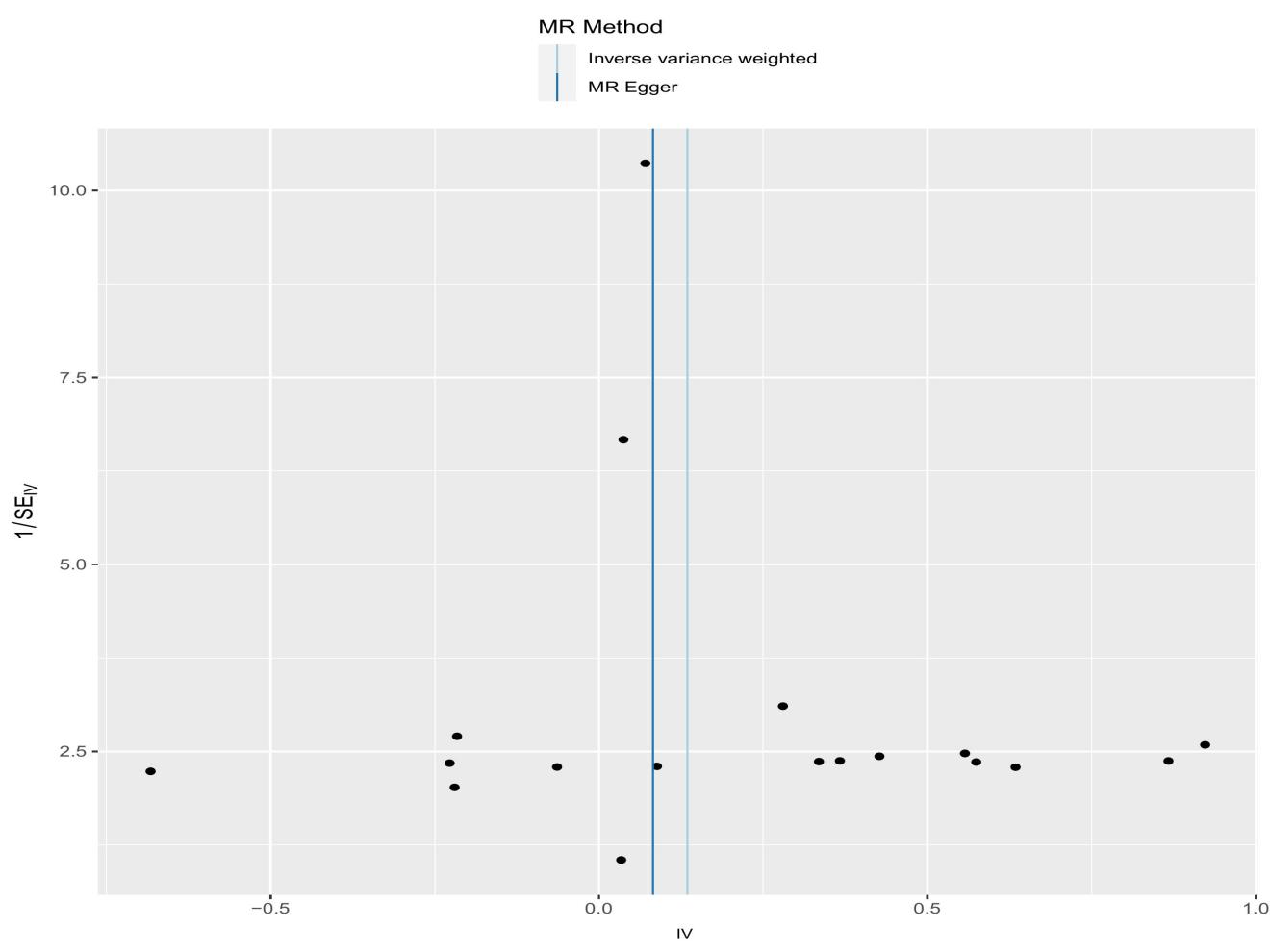


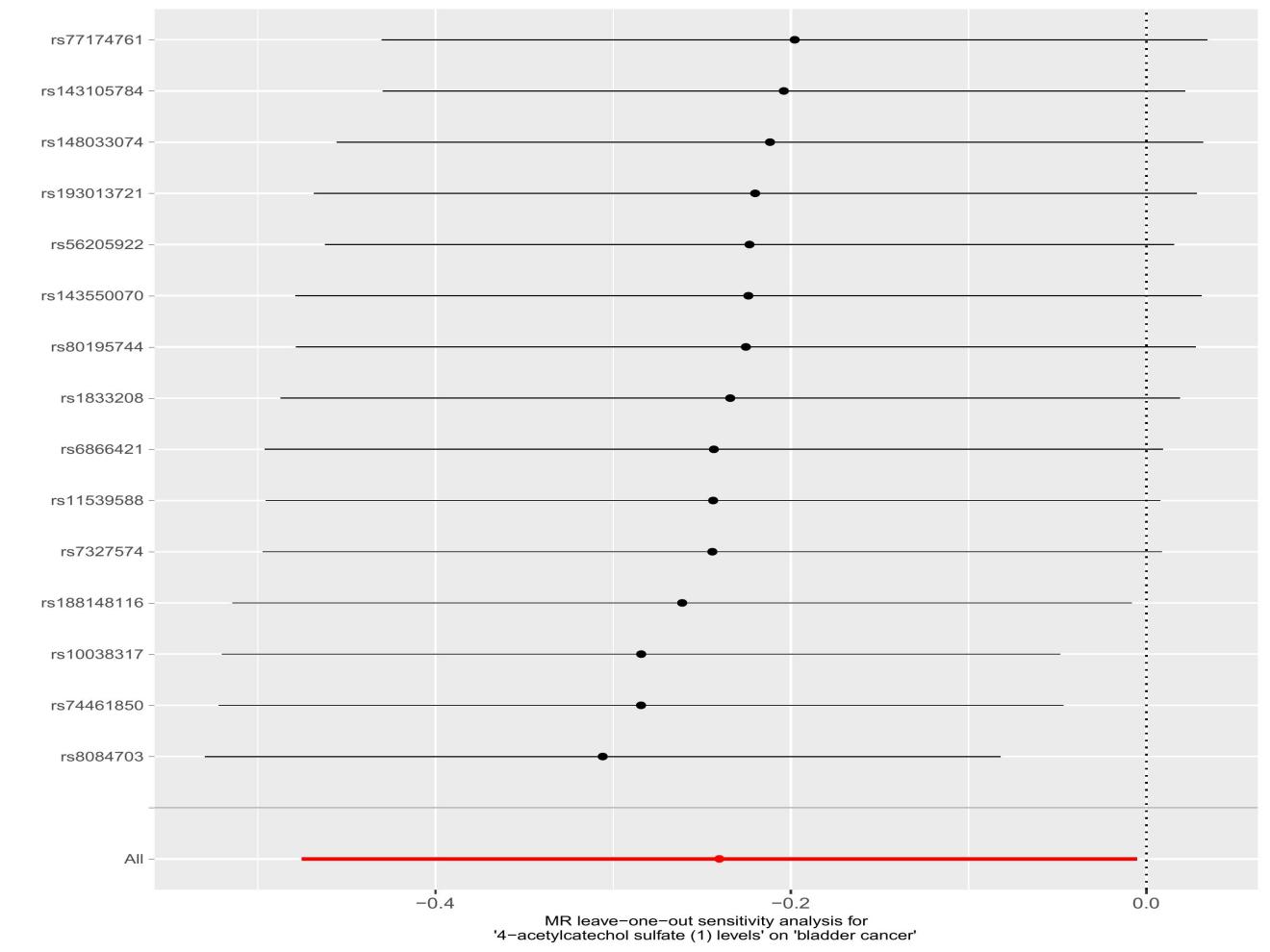


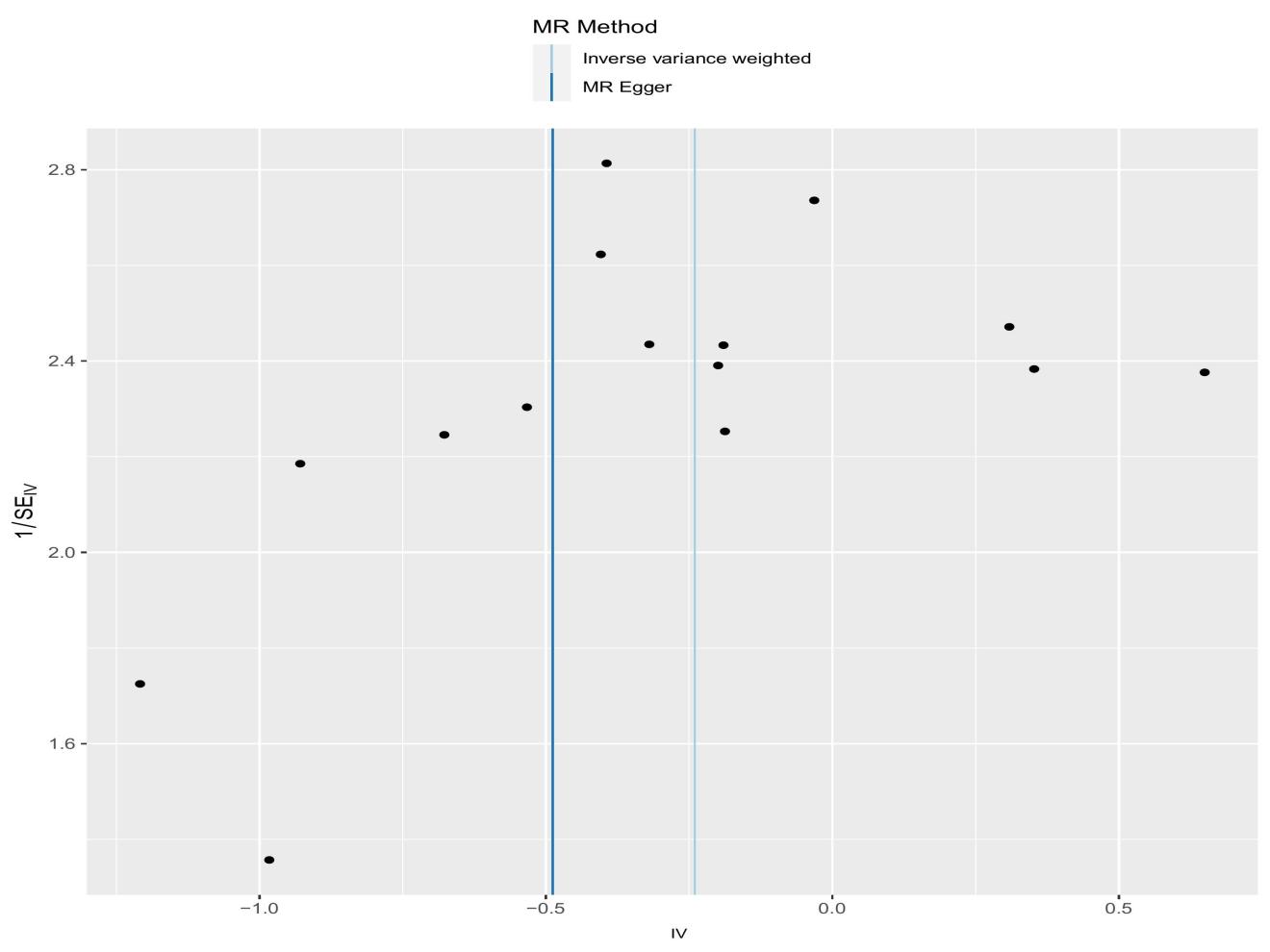


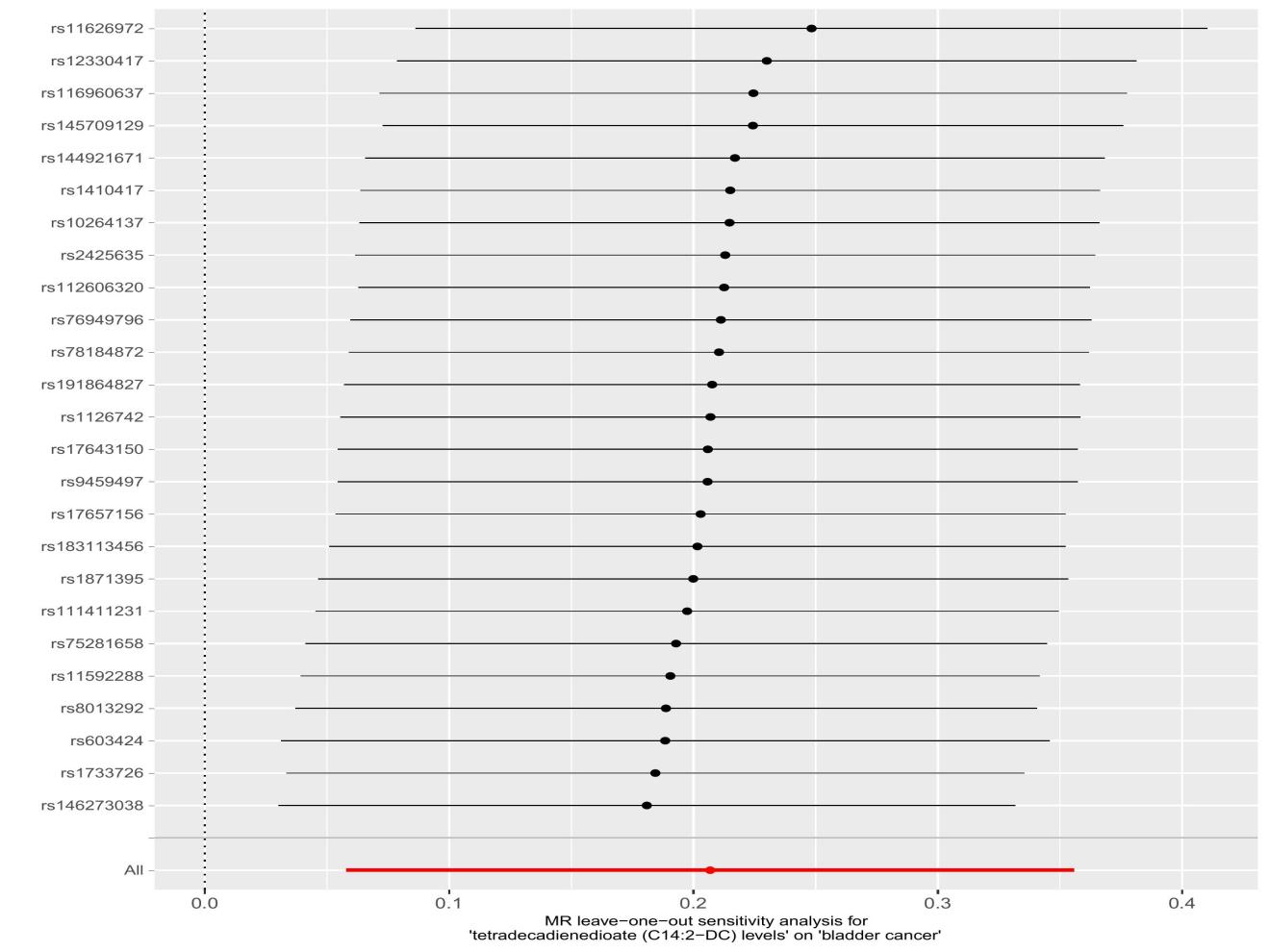


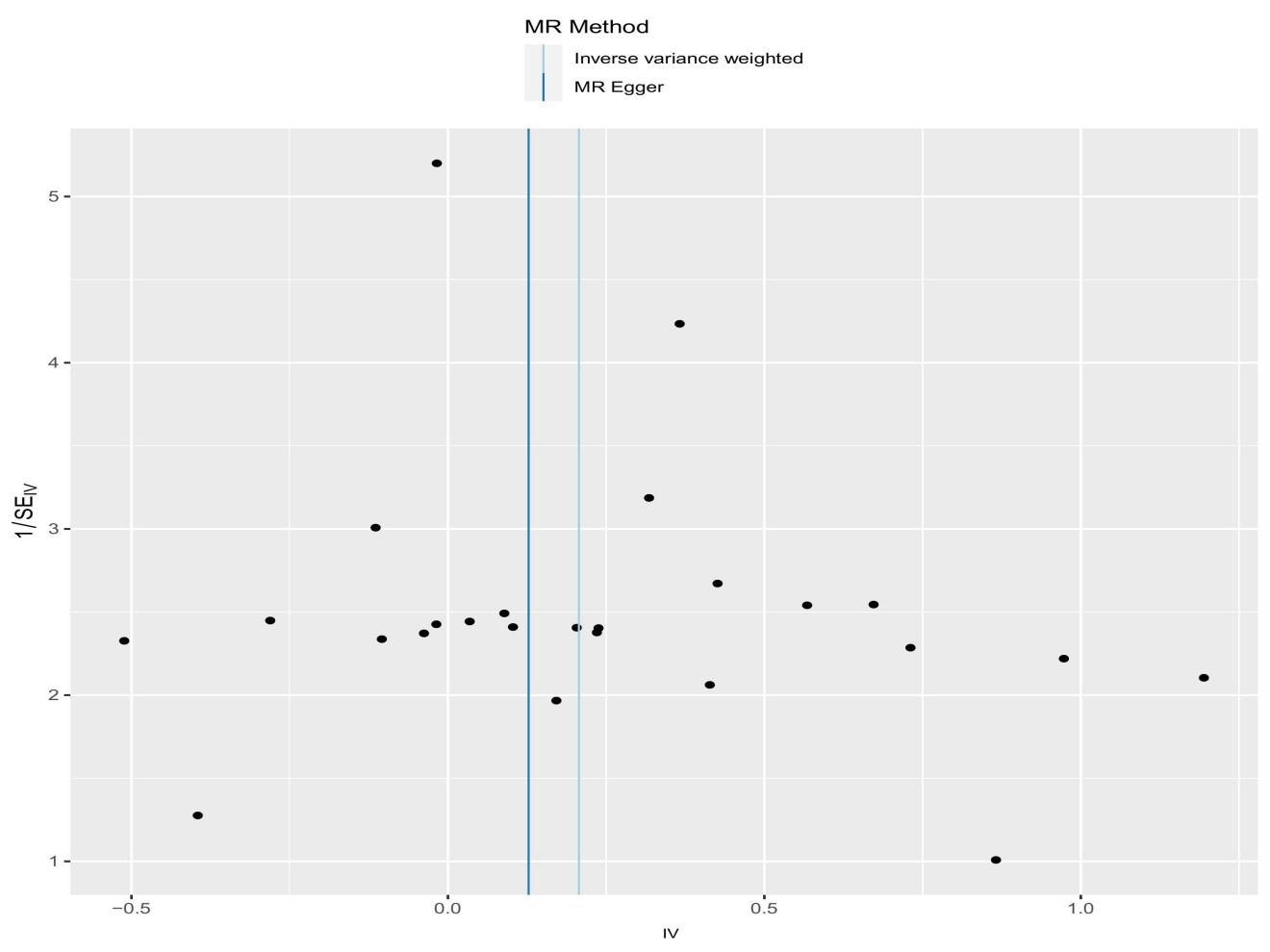


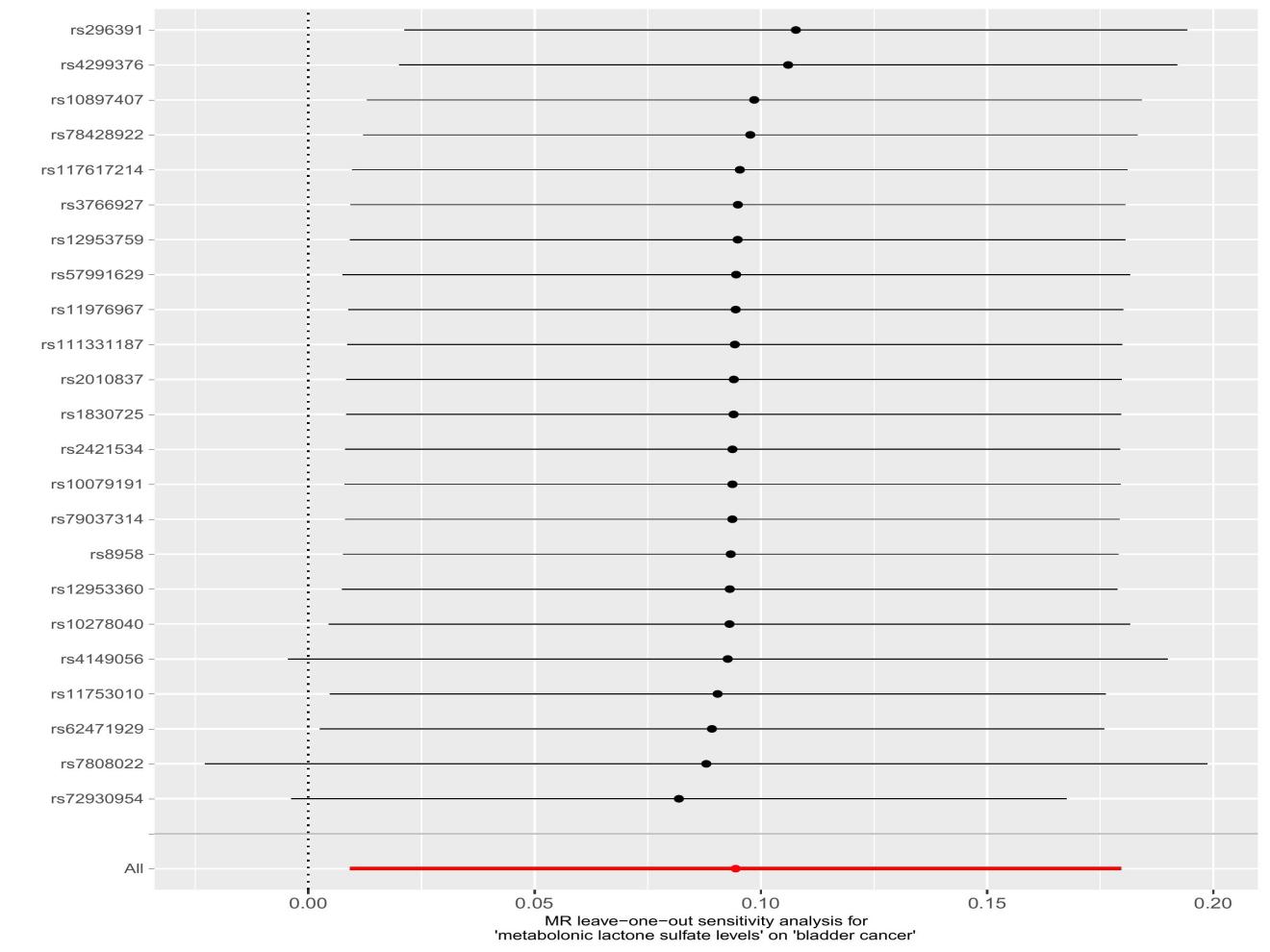


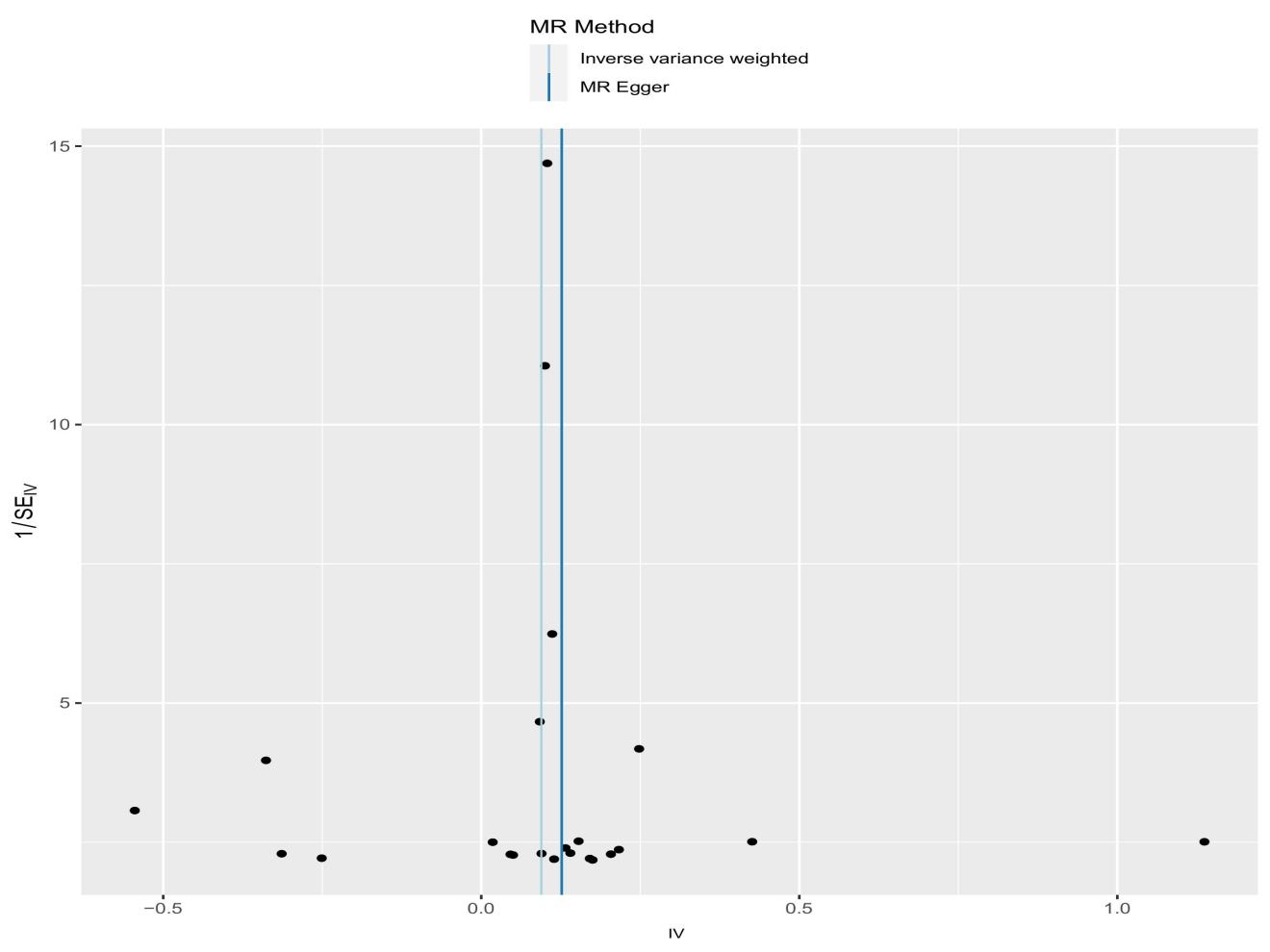


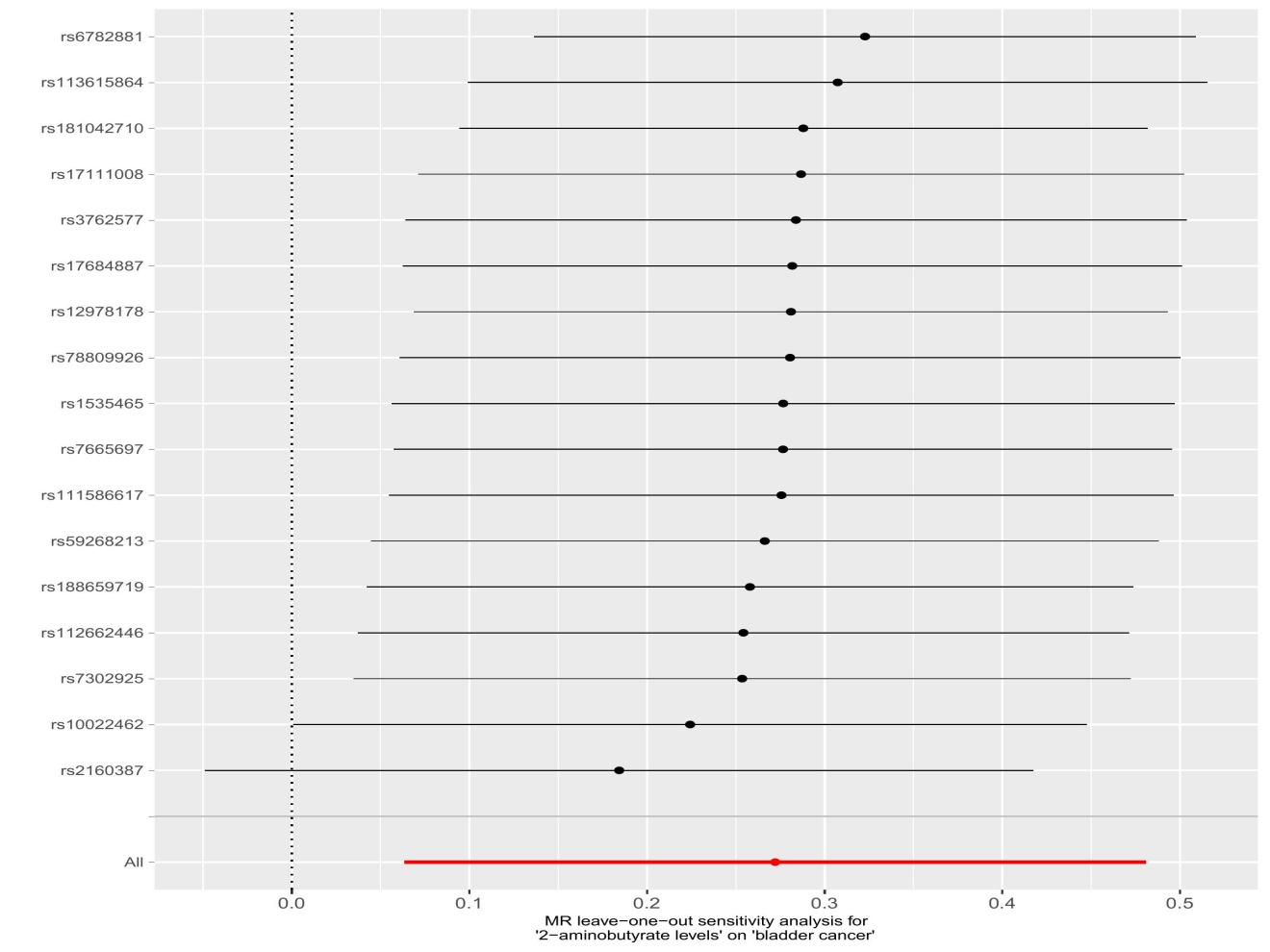


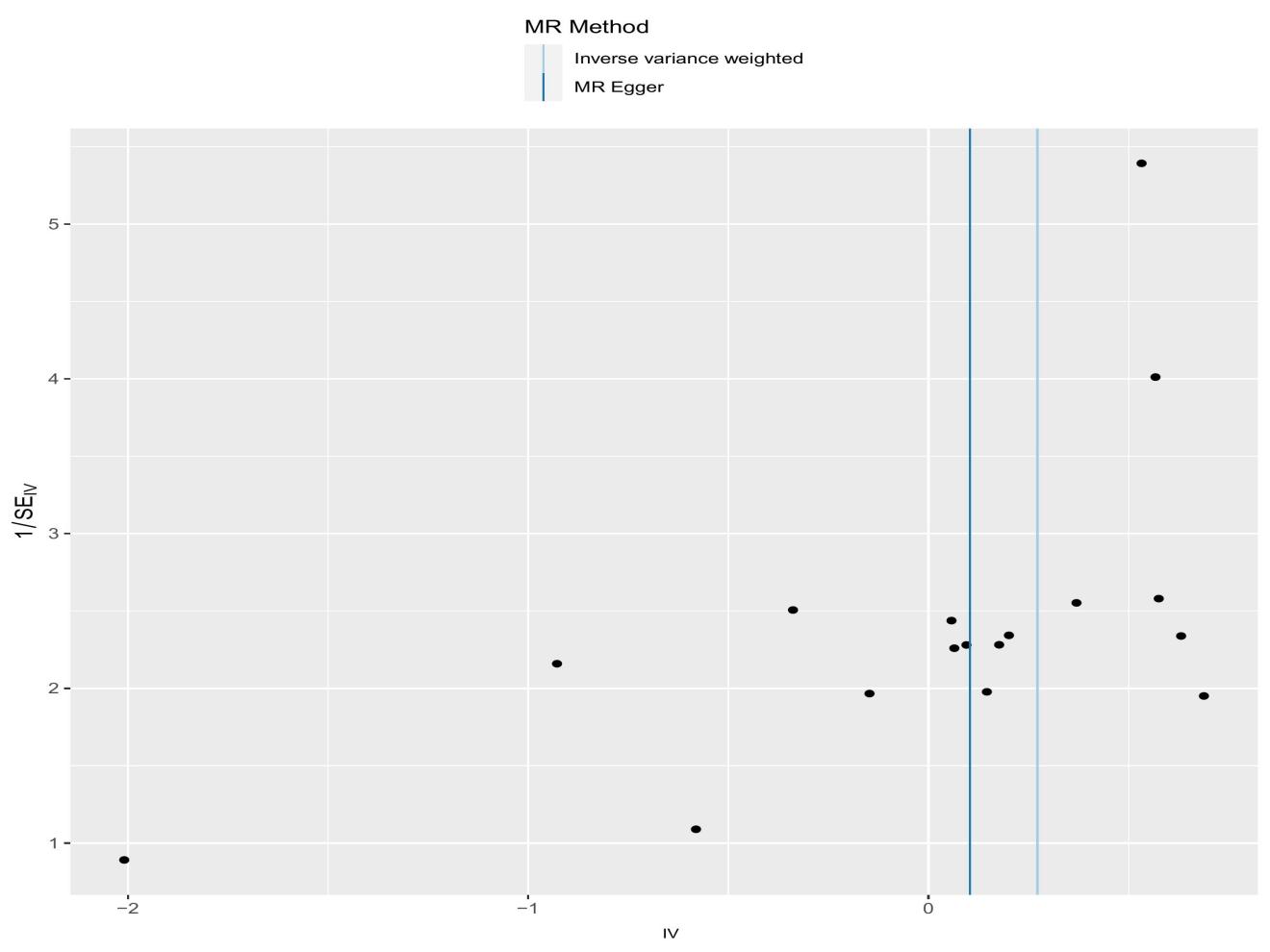


Kidney Cancer


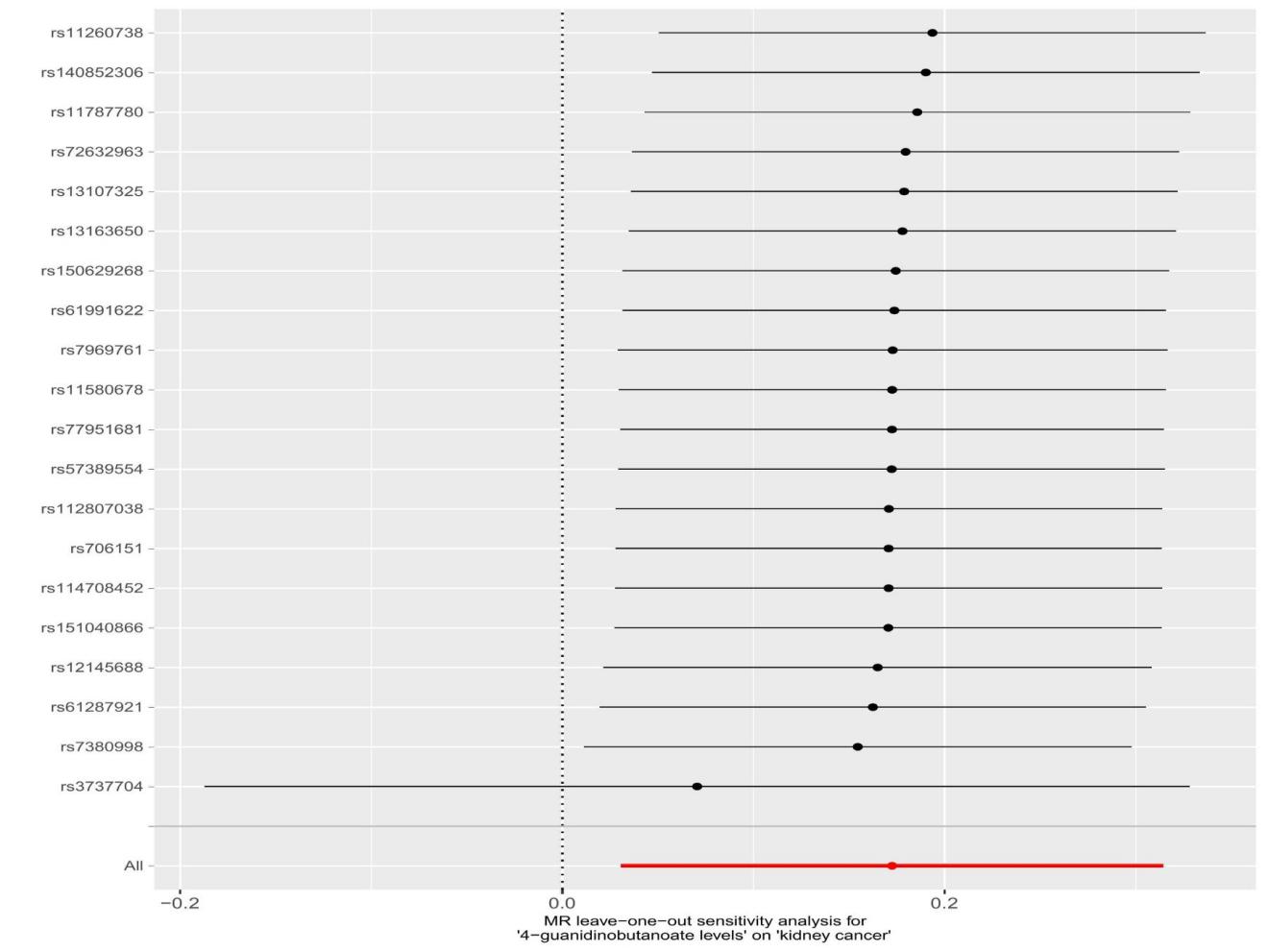


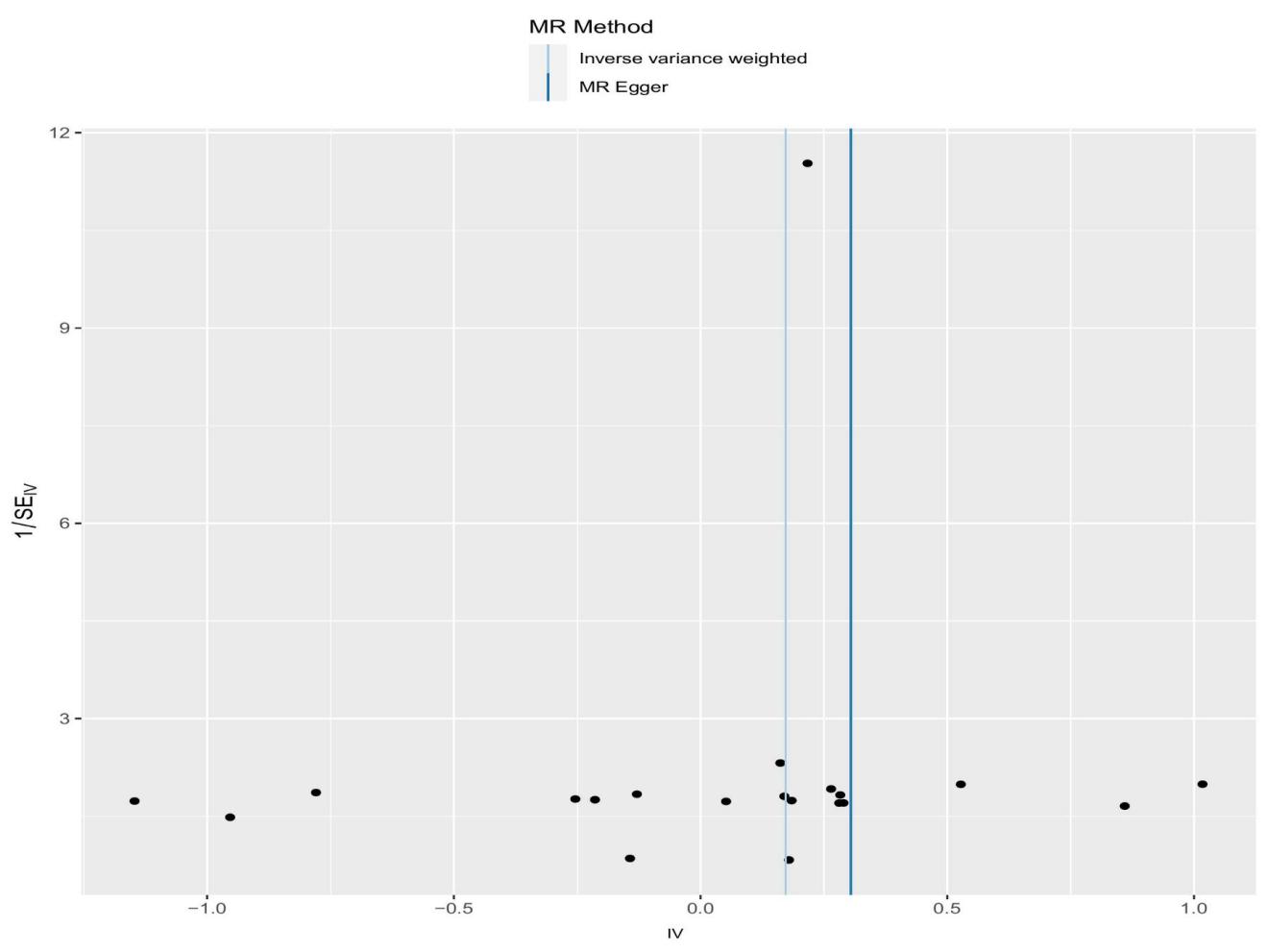


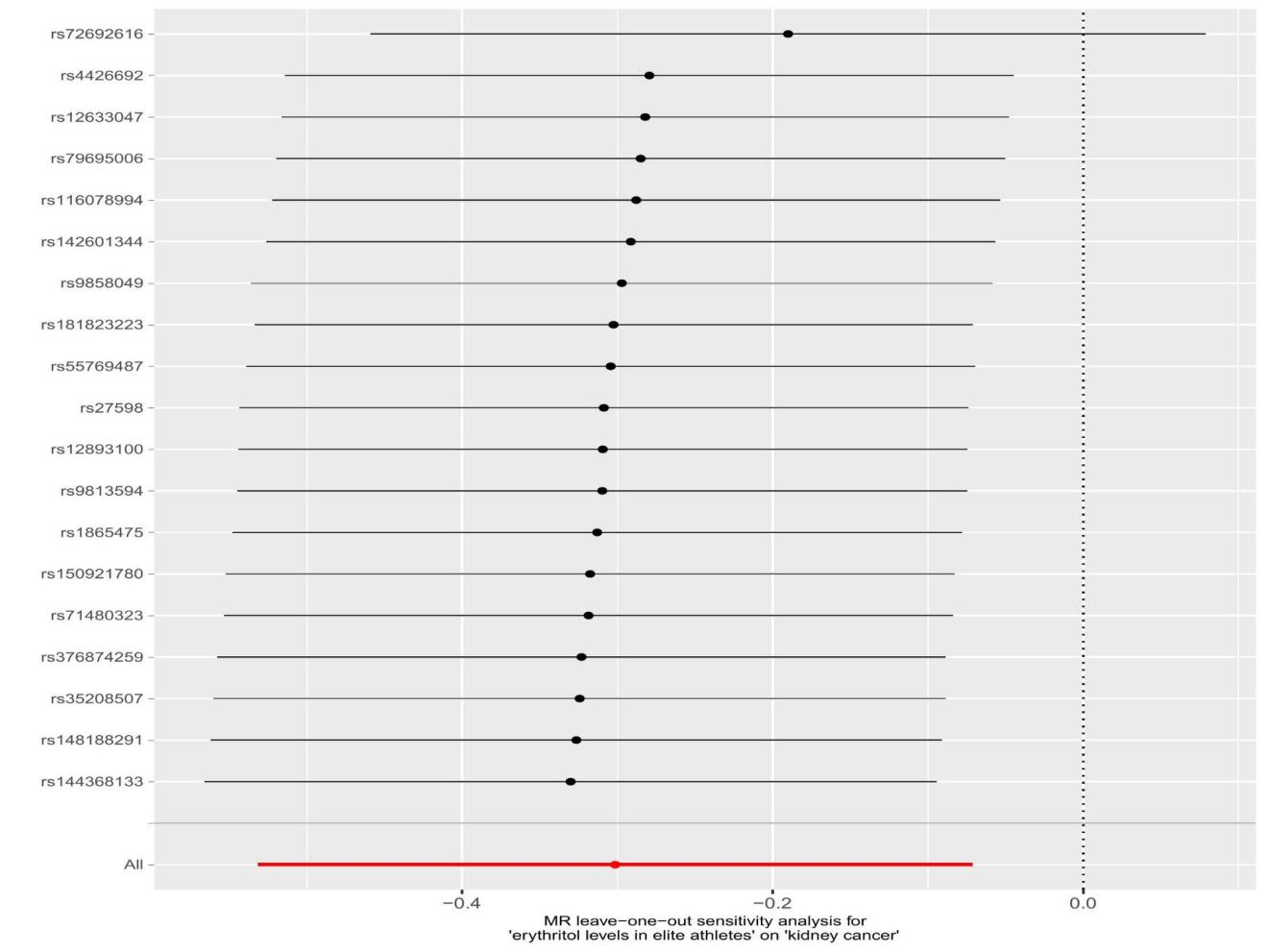


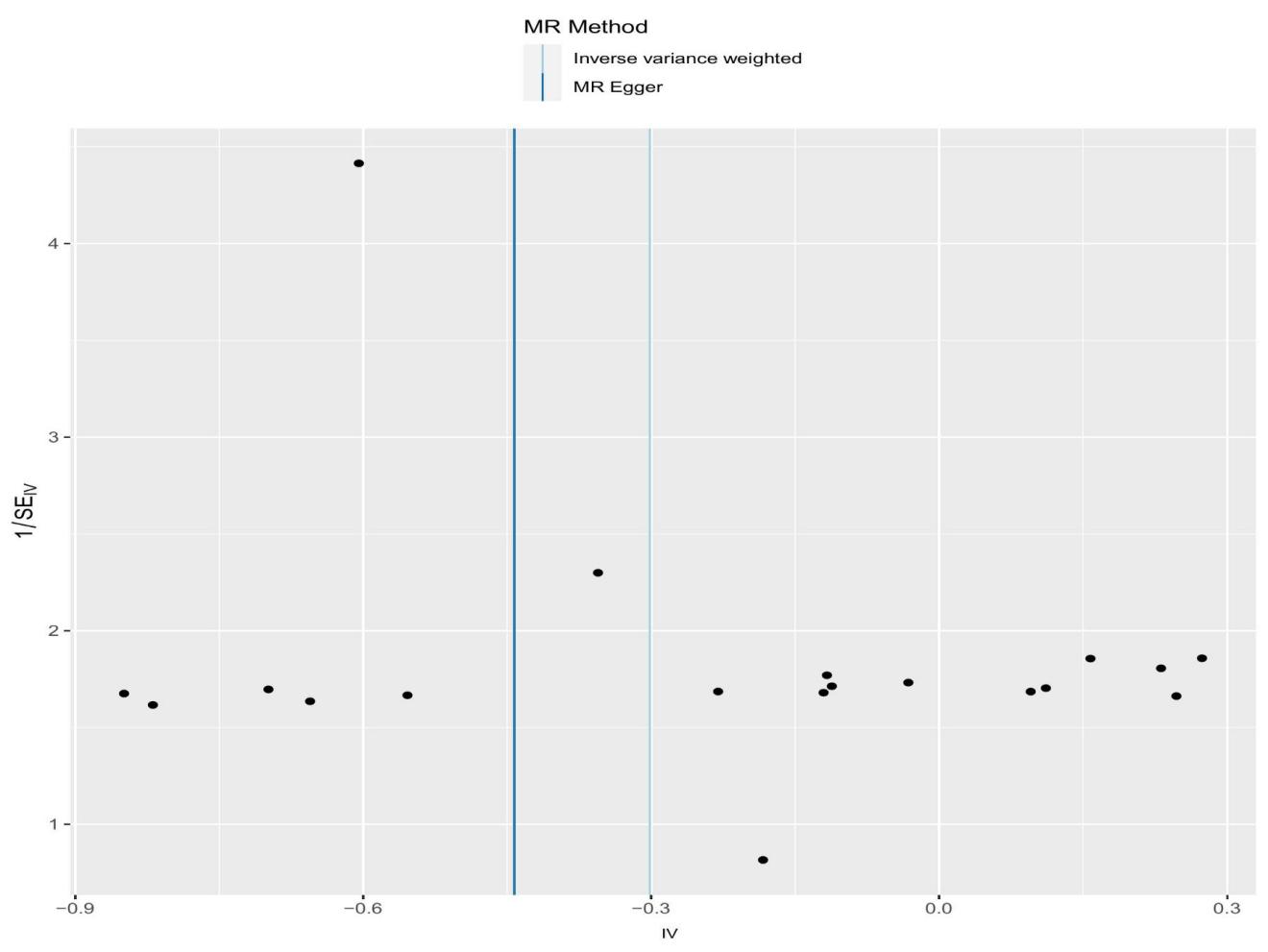


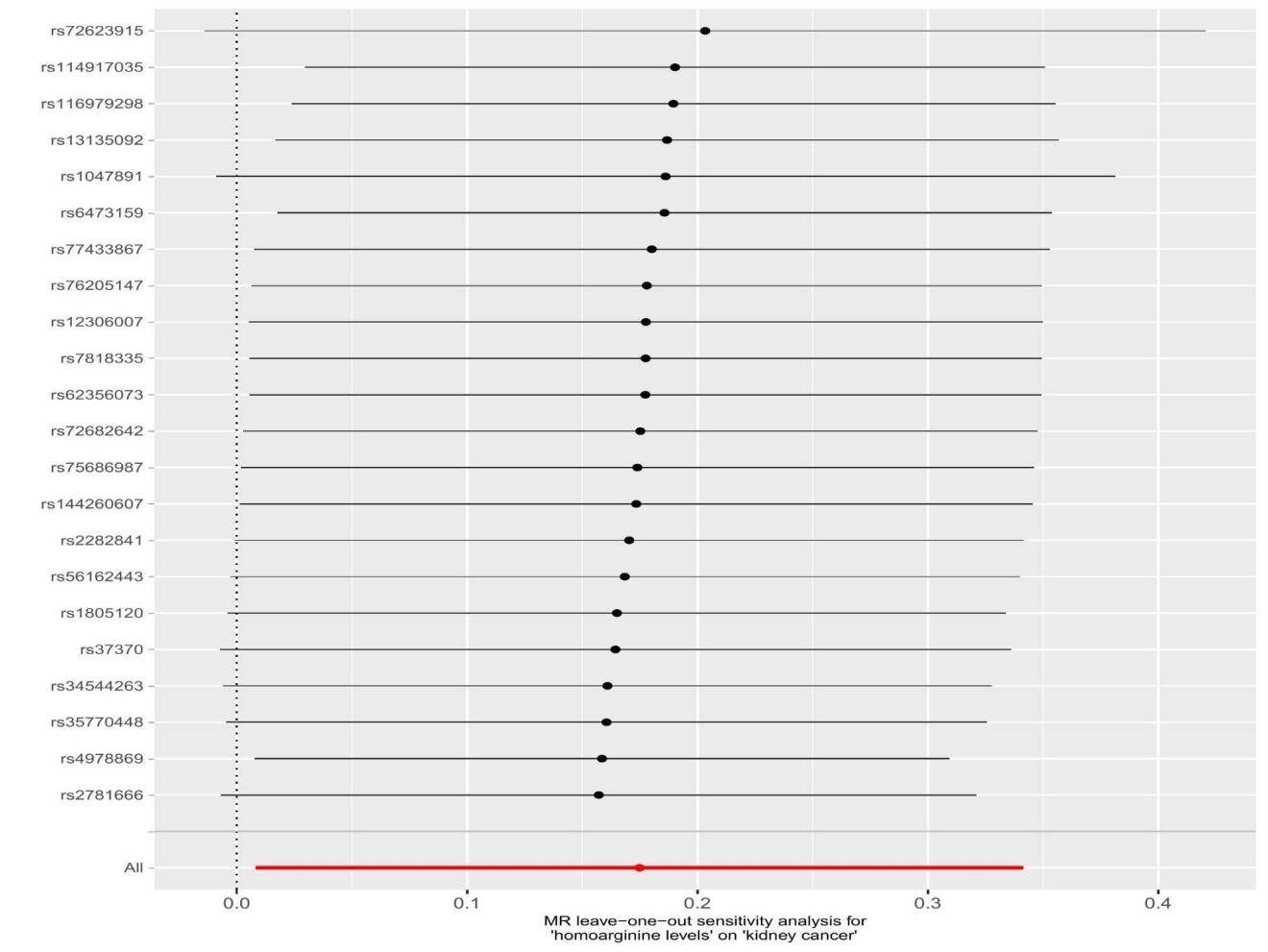


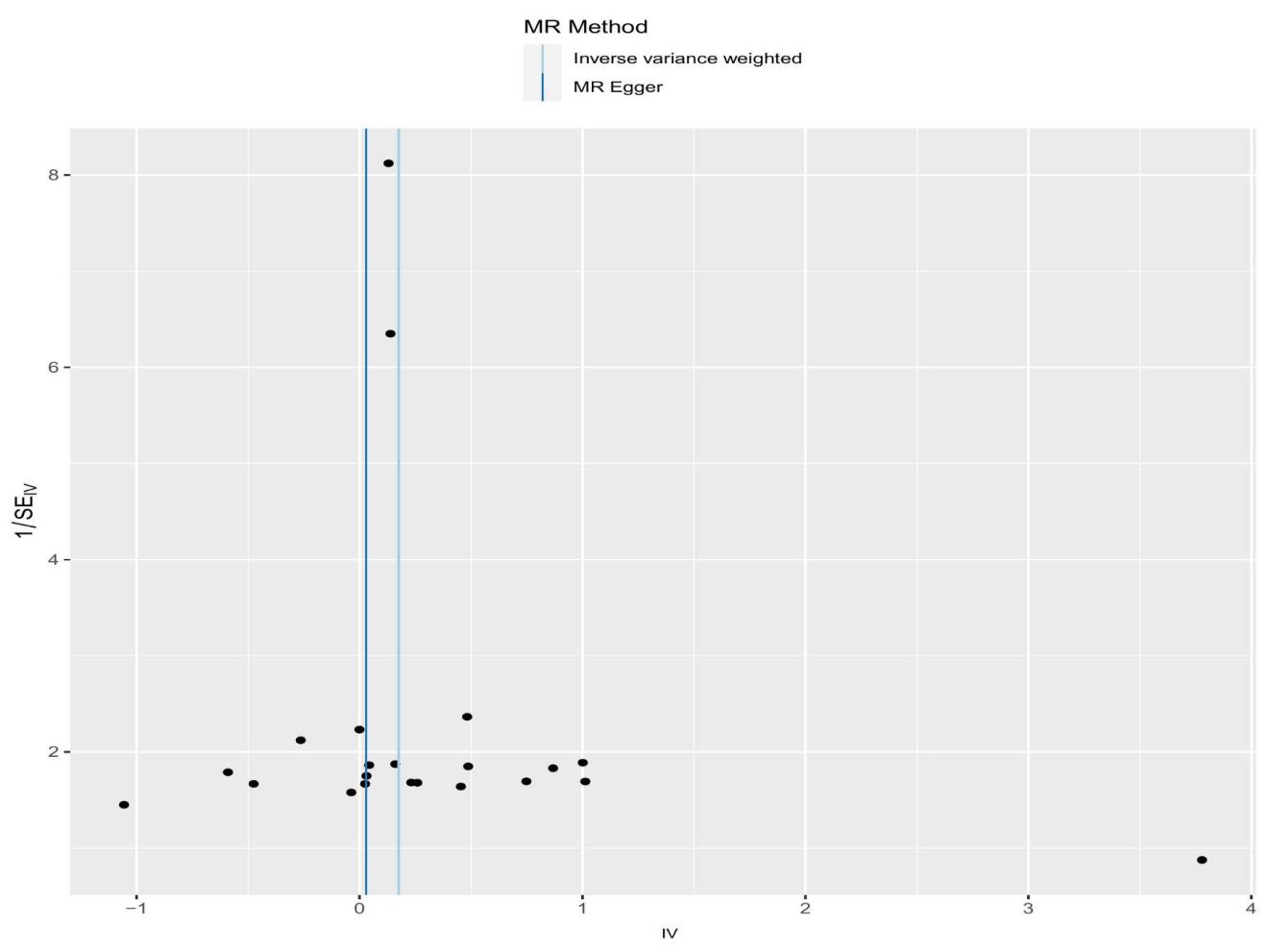


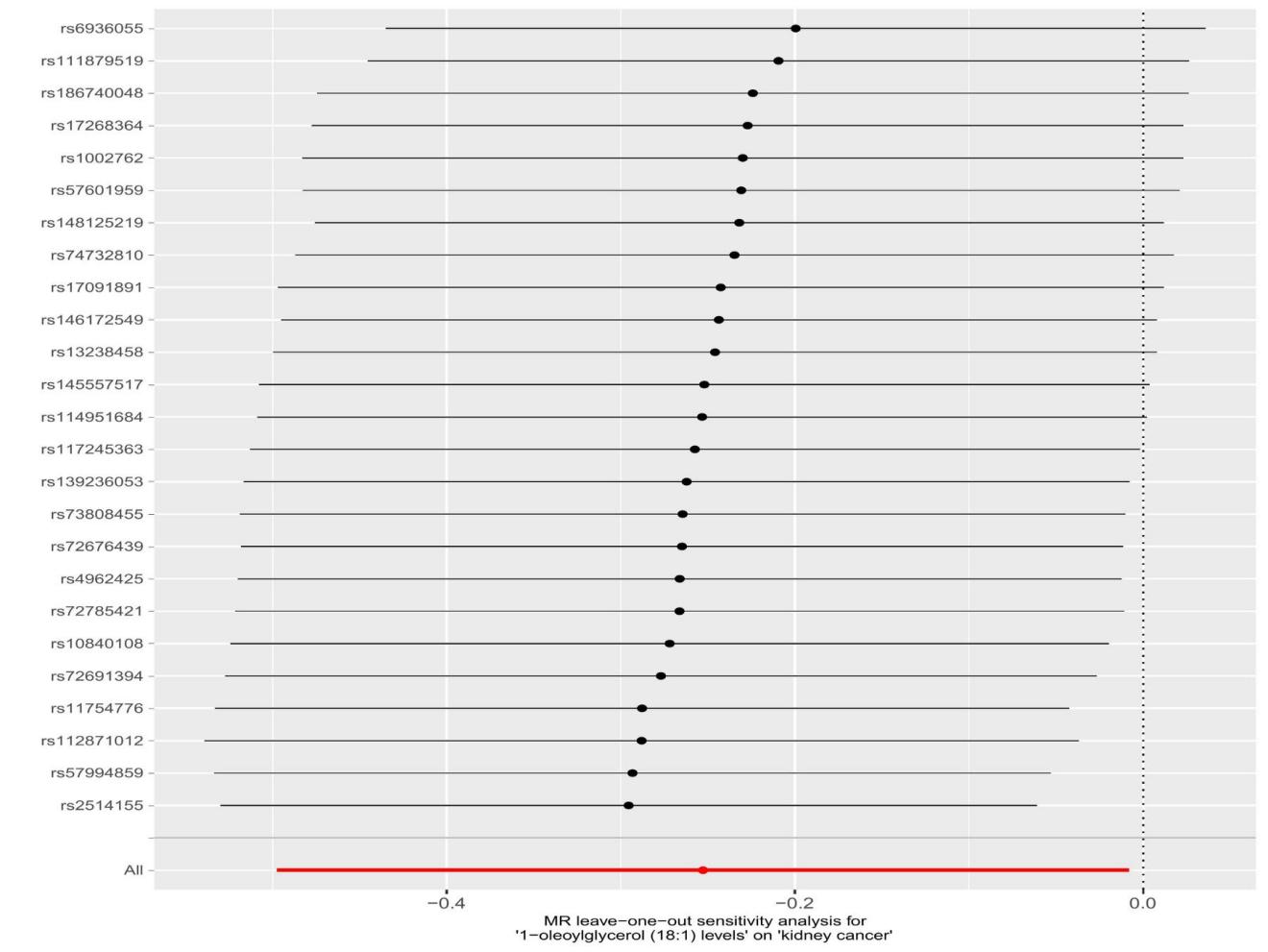


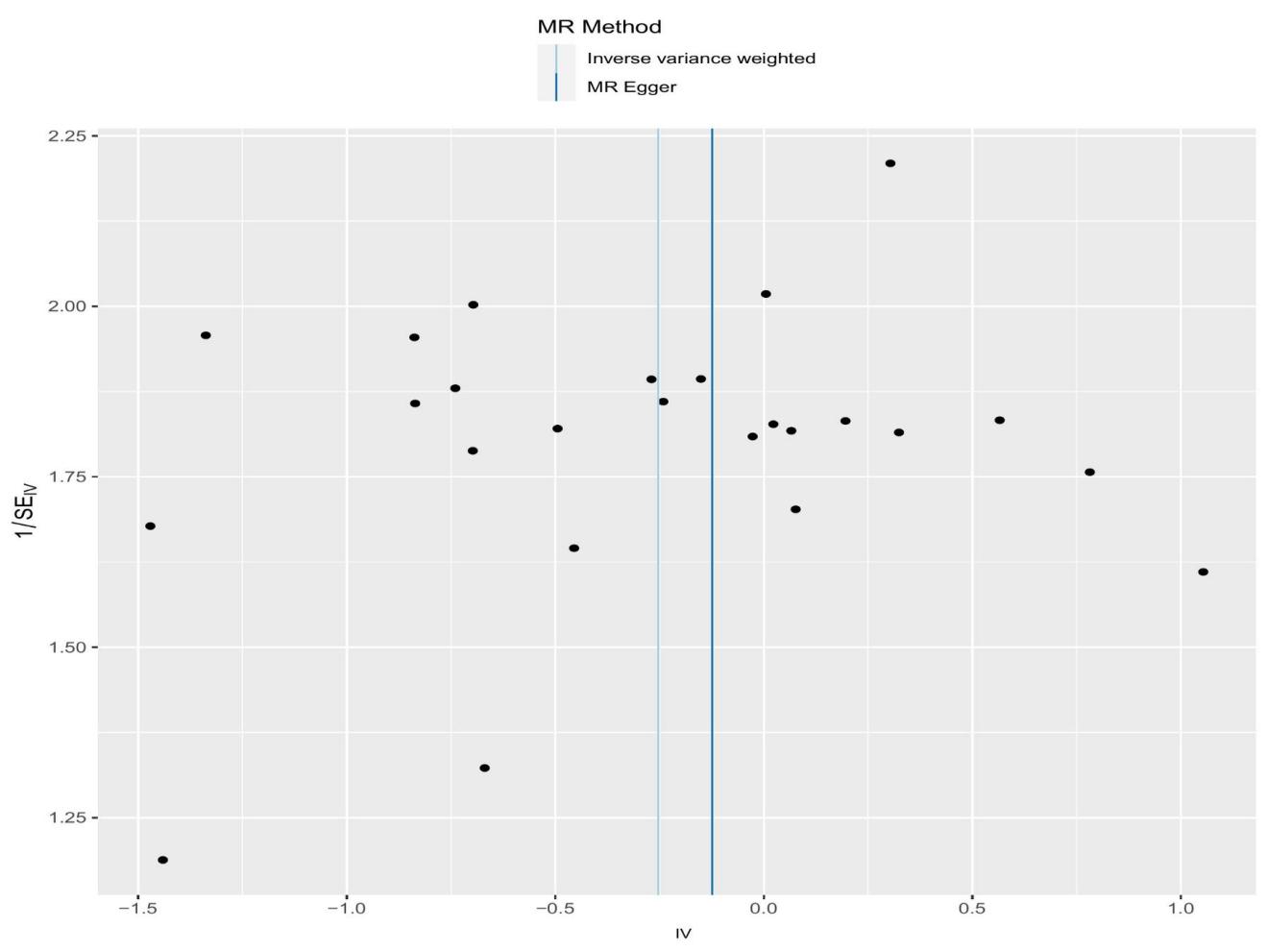


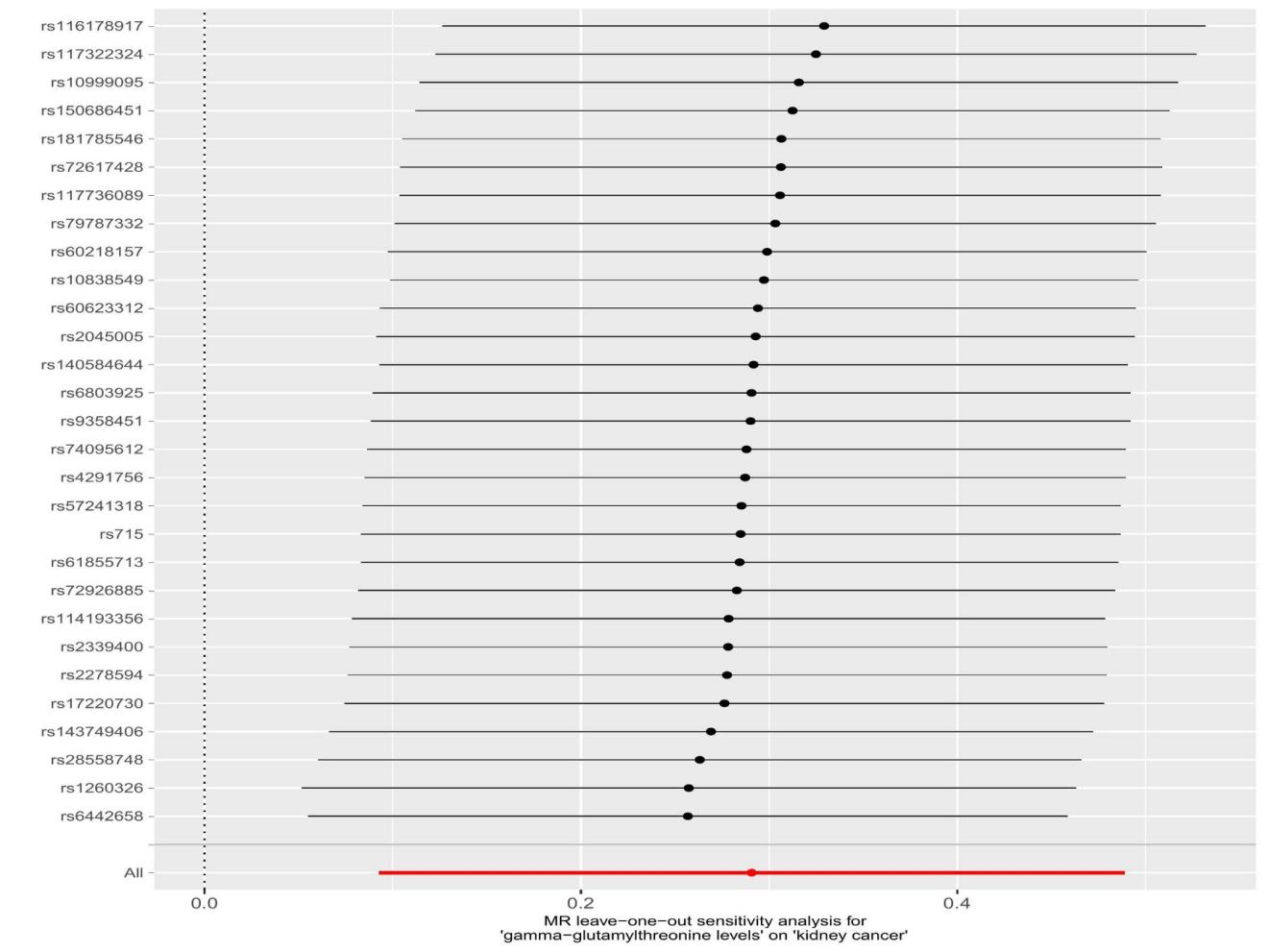


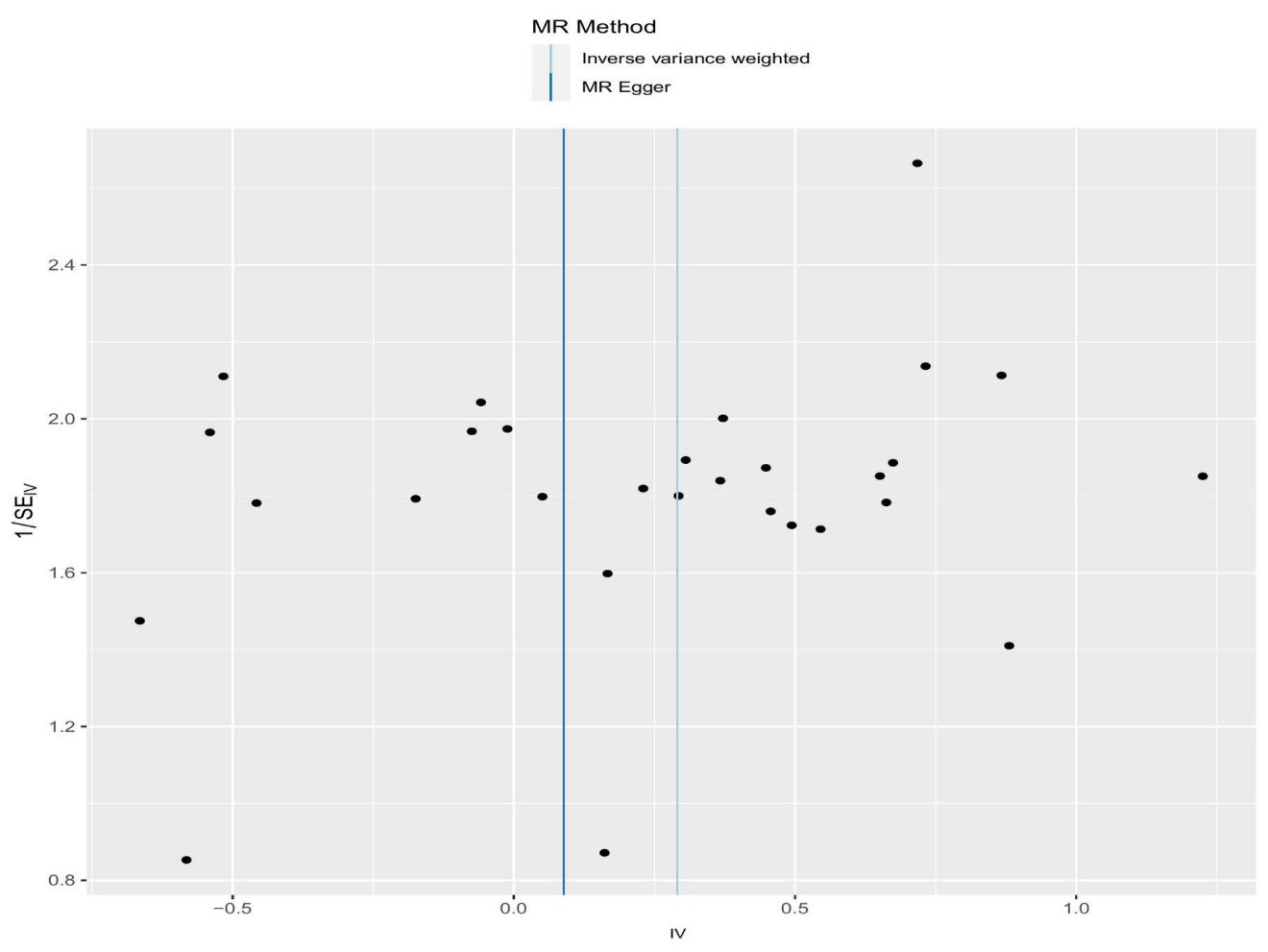


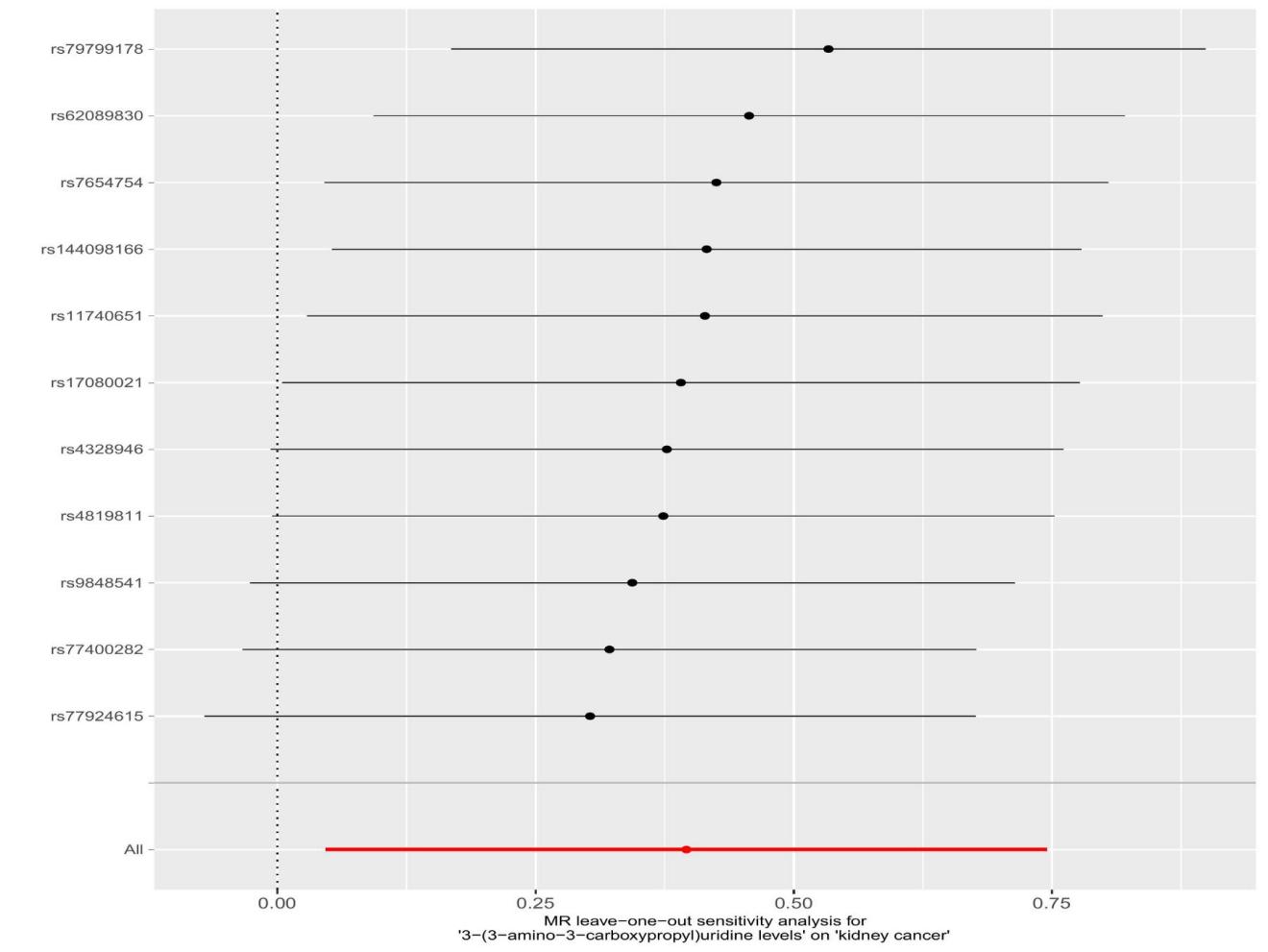


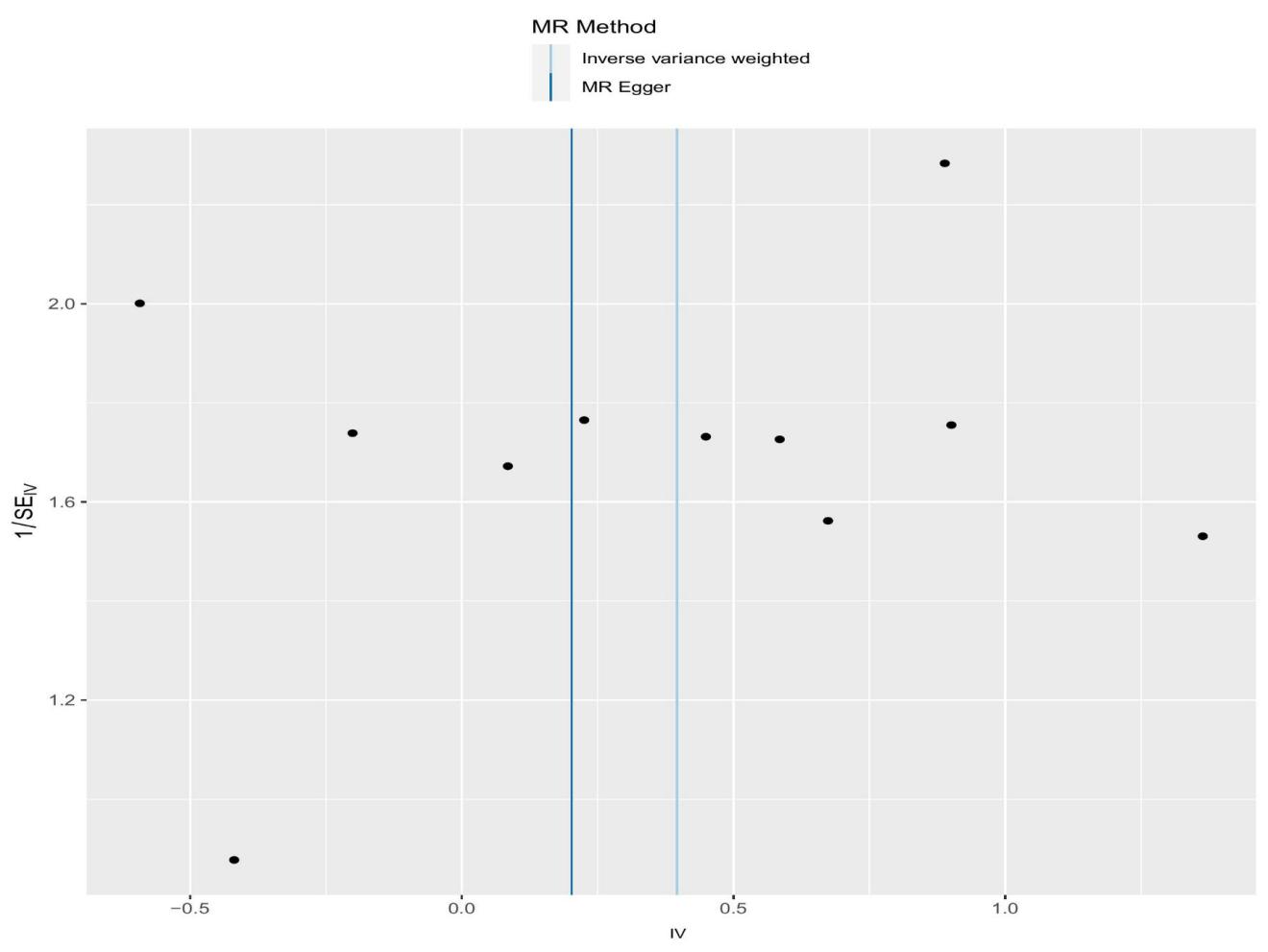


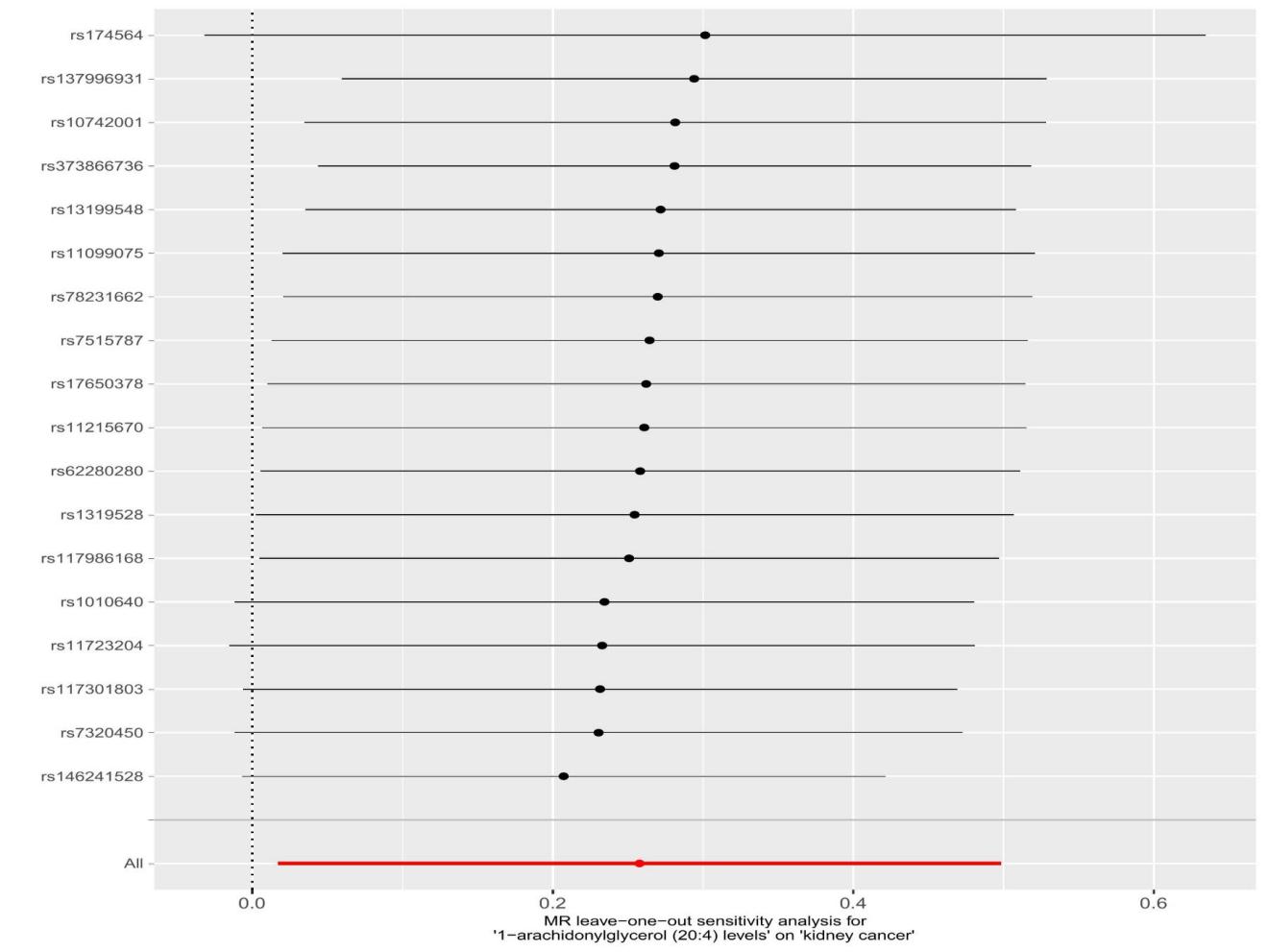


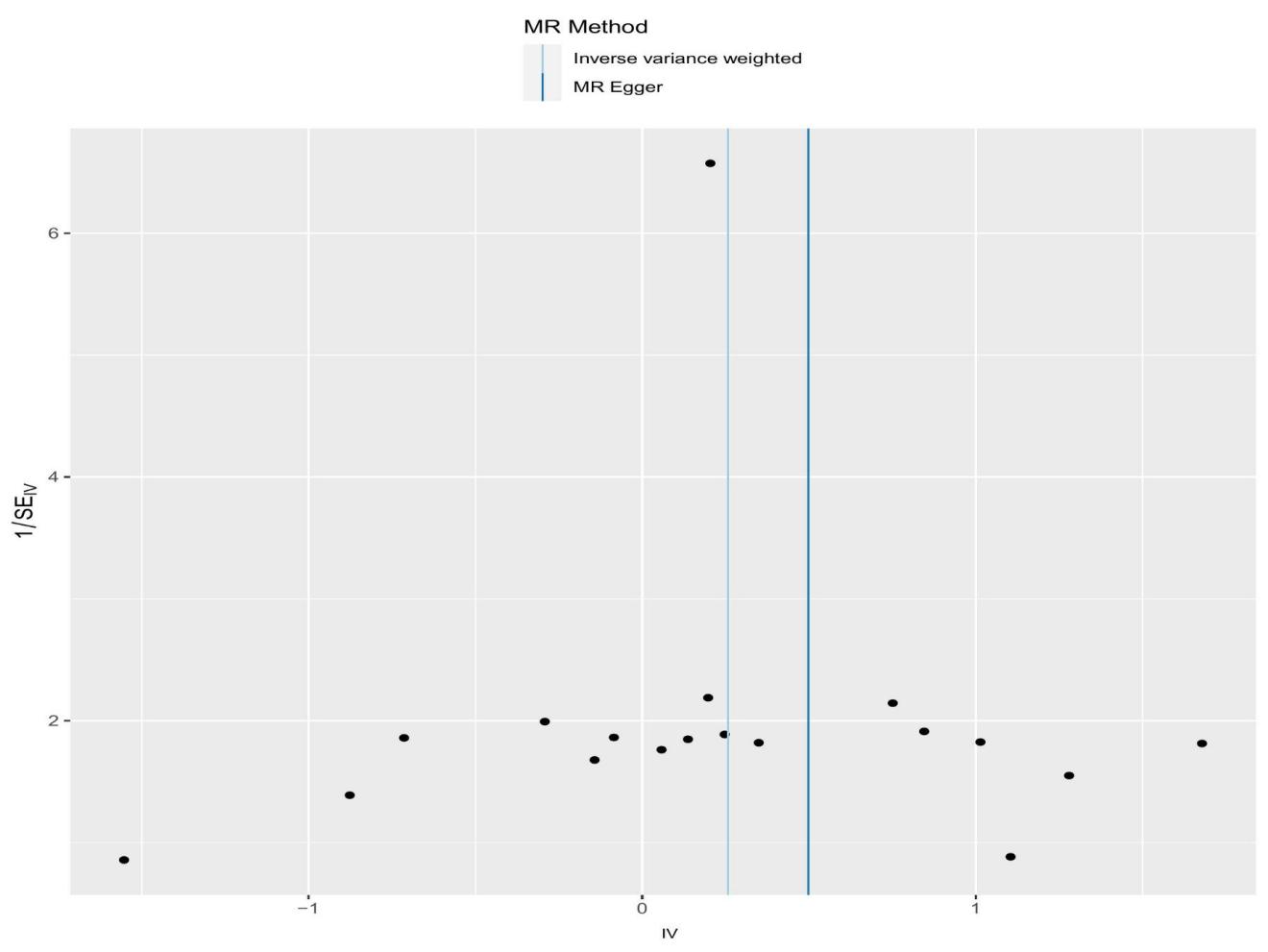


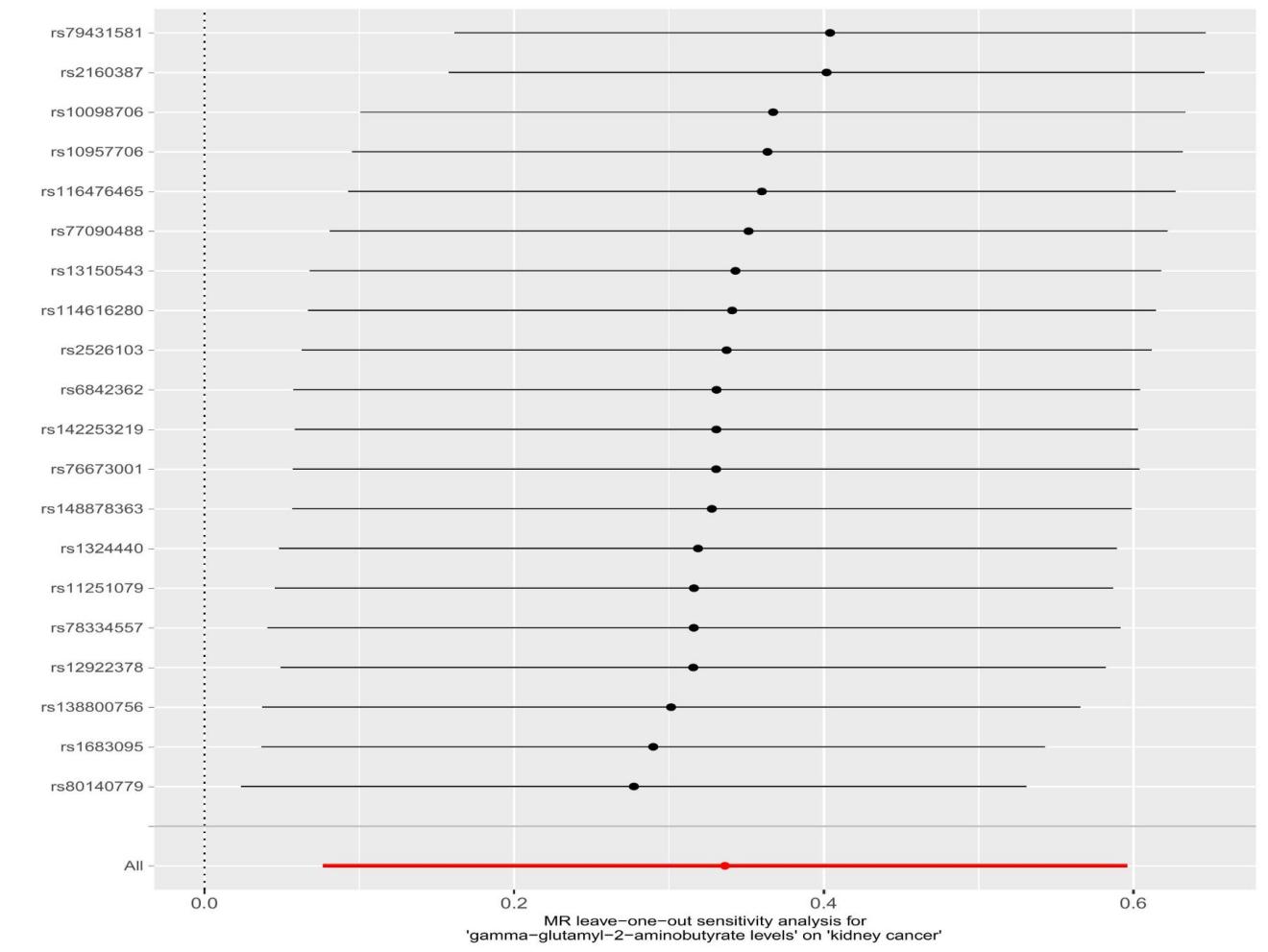


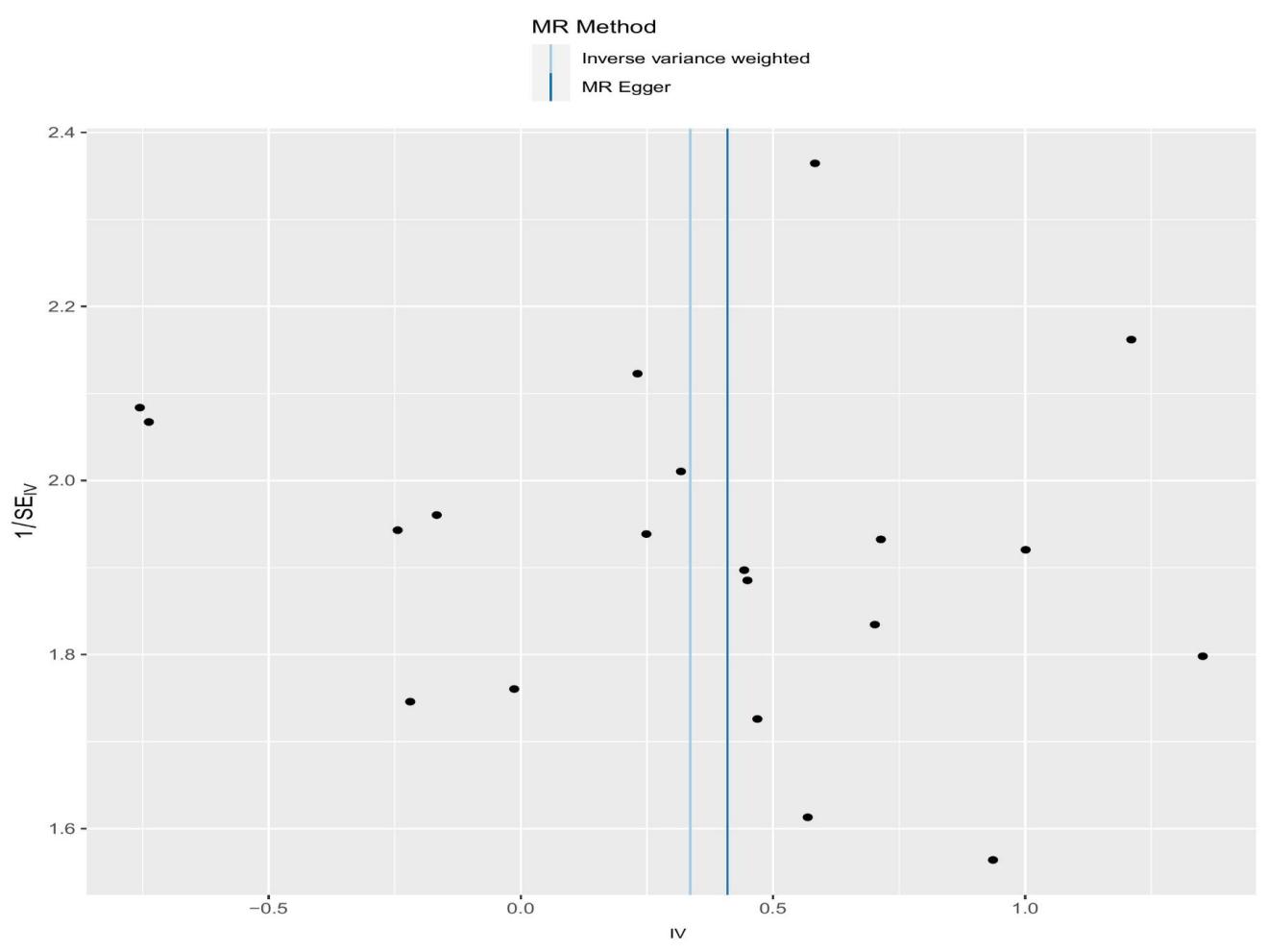


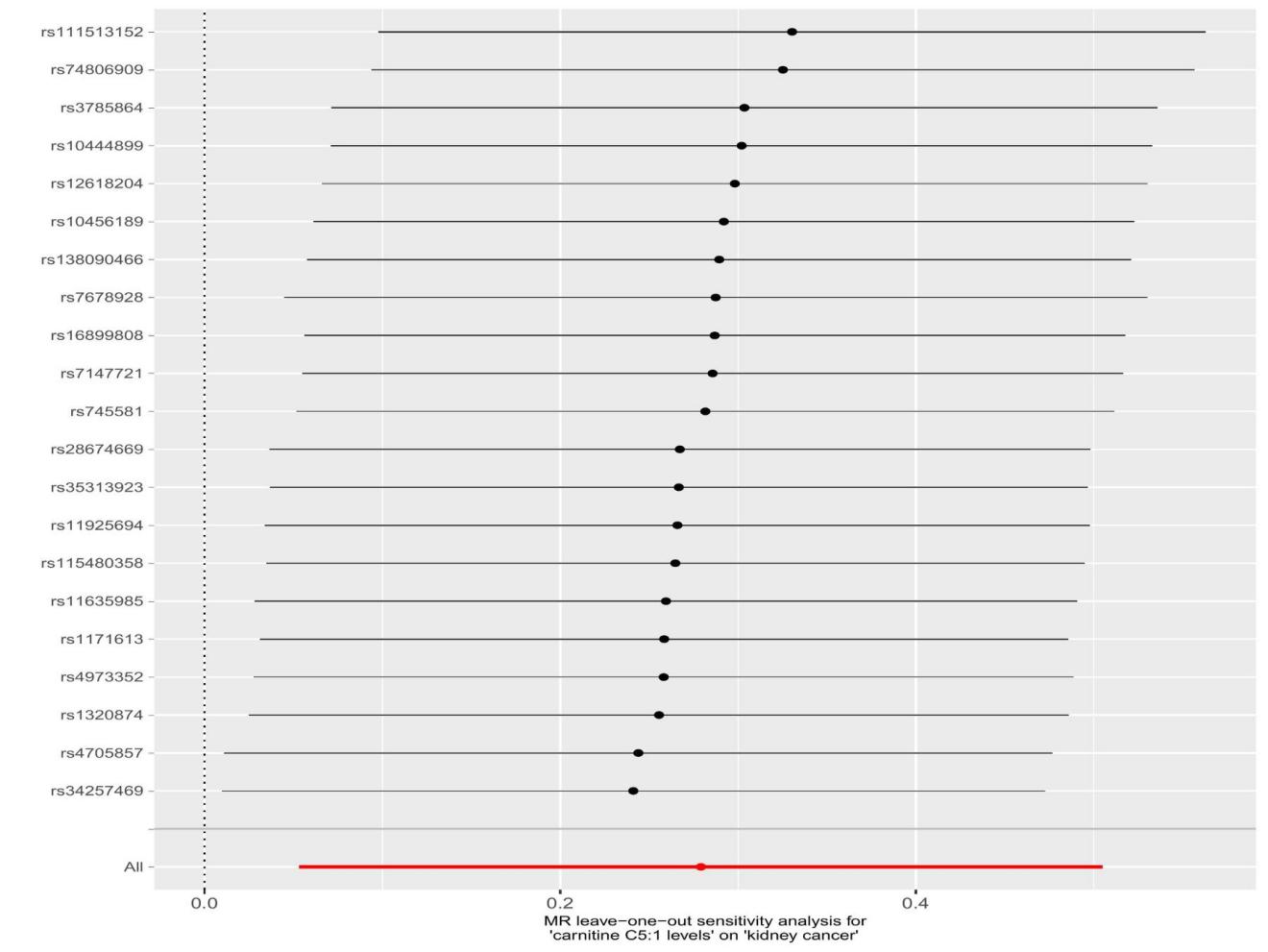


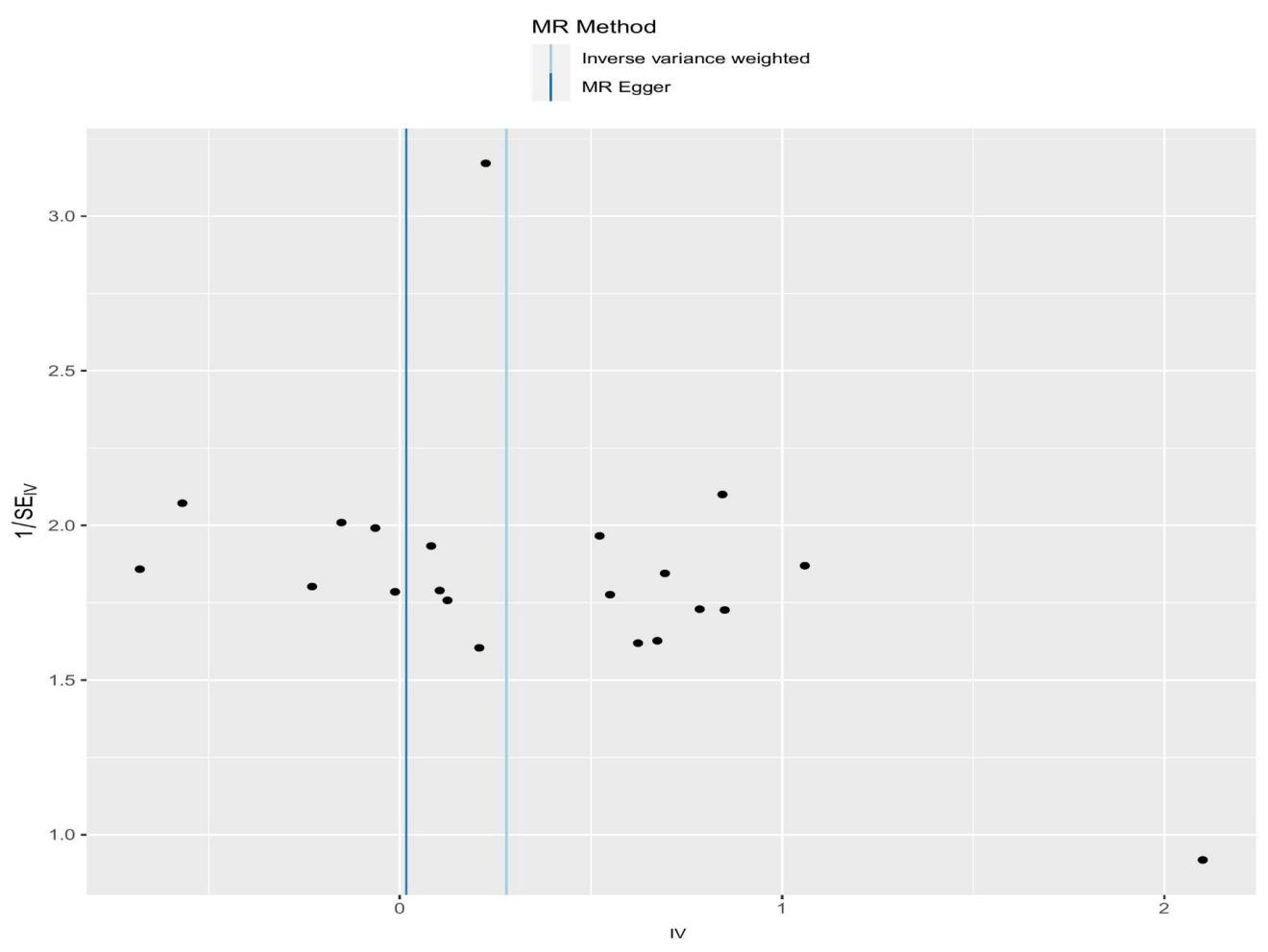


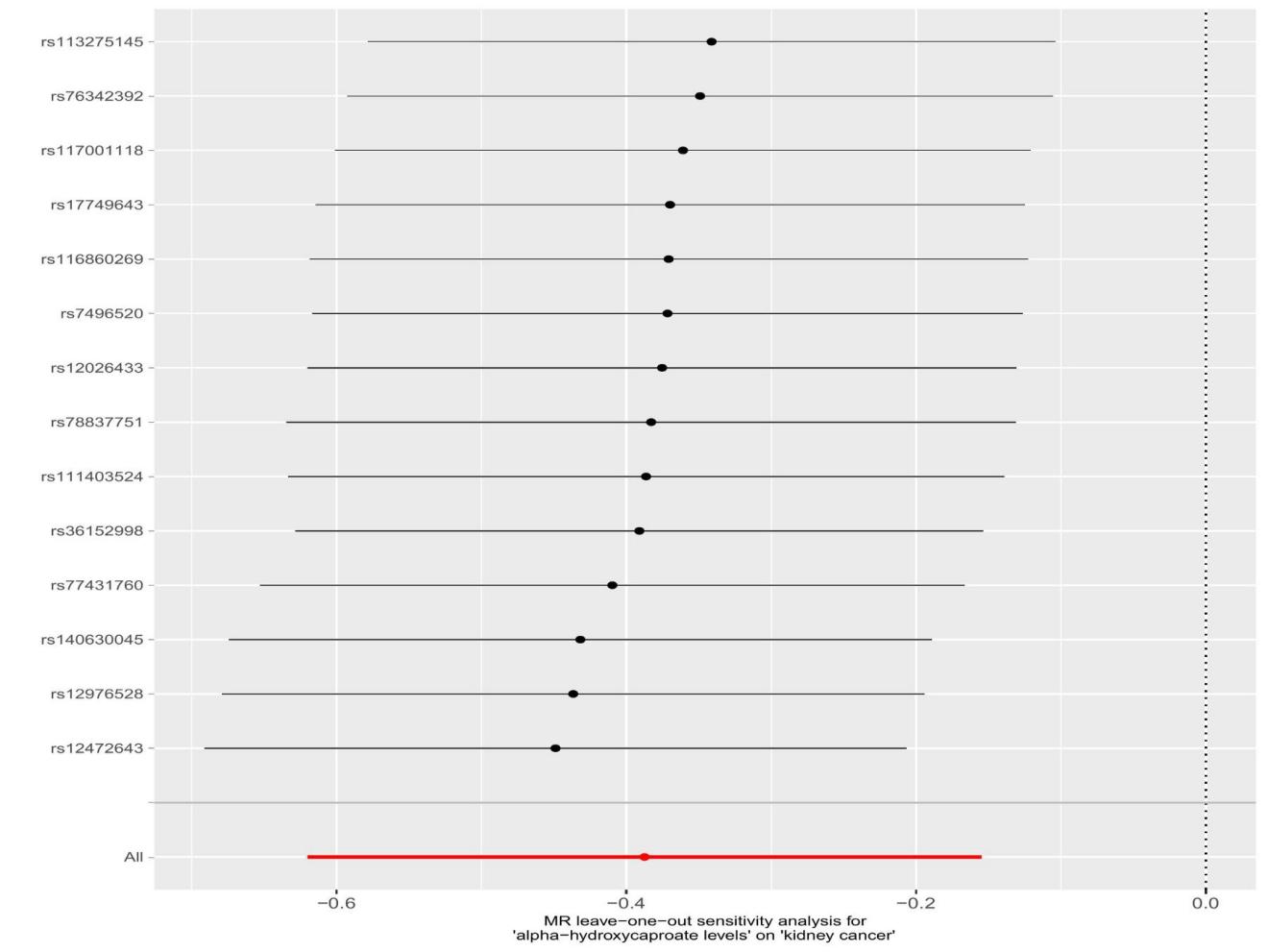


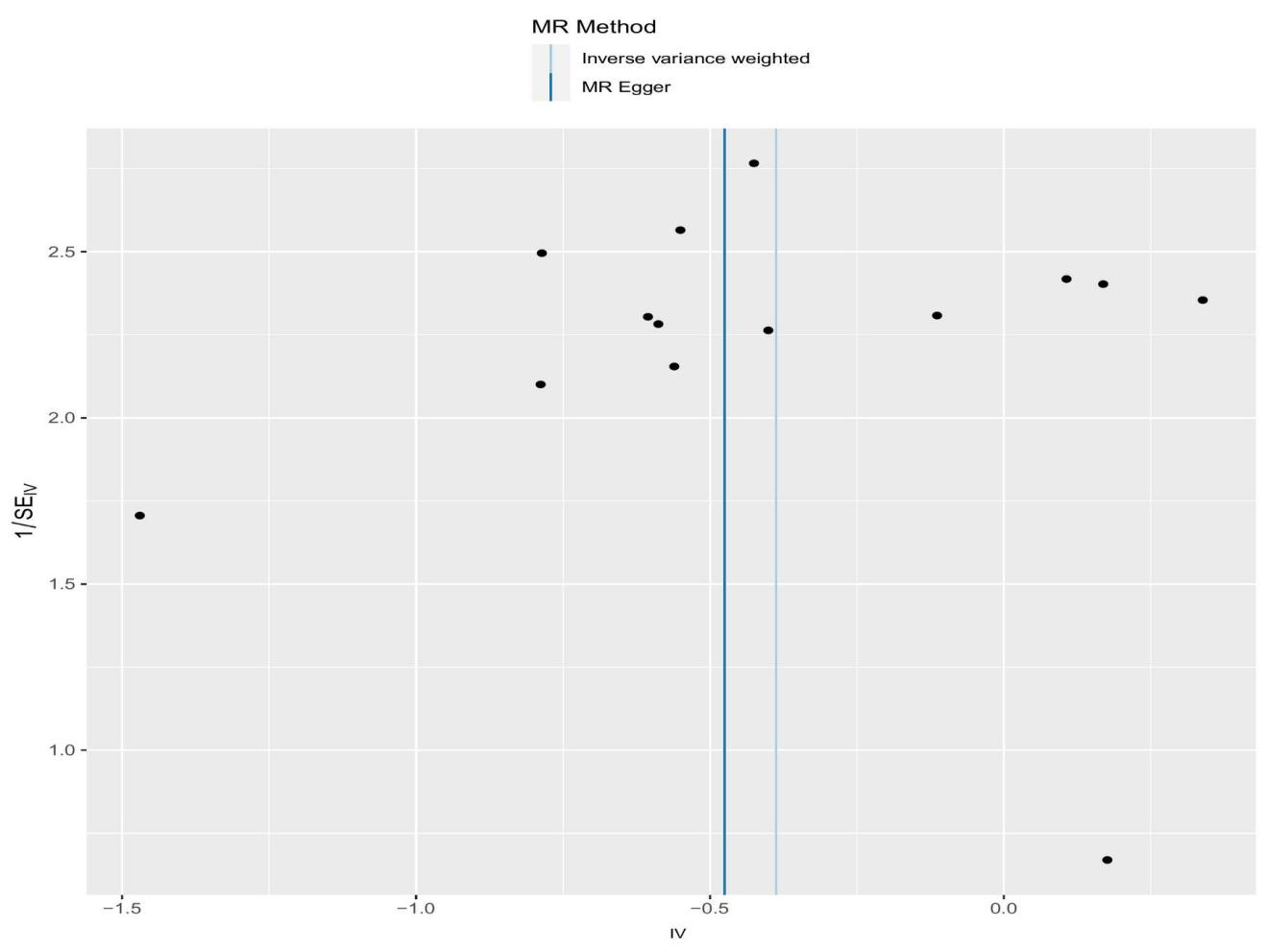


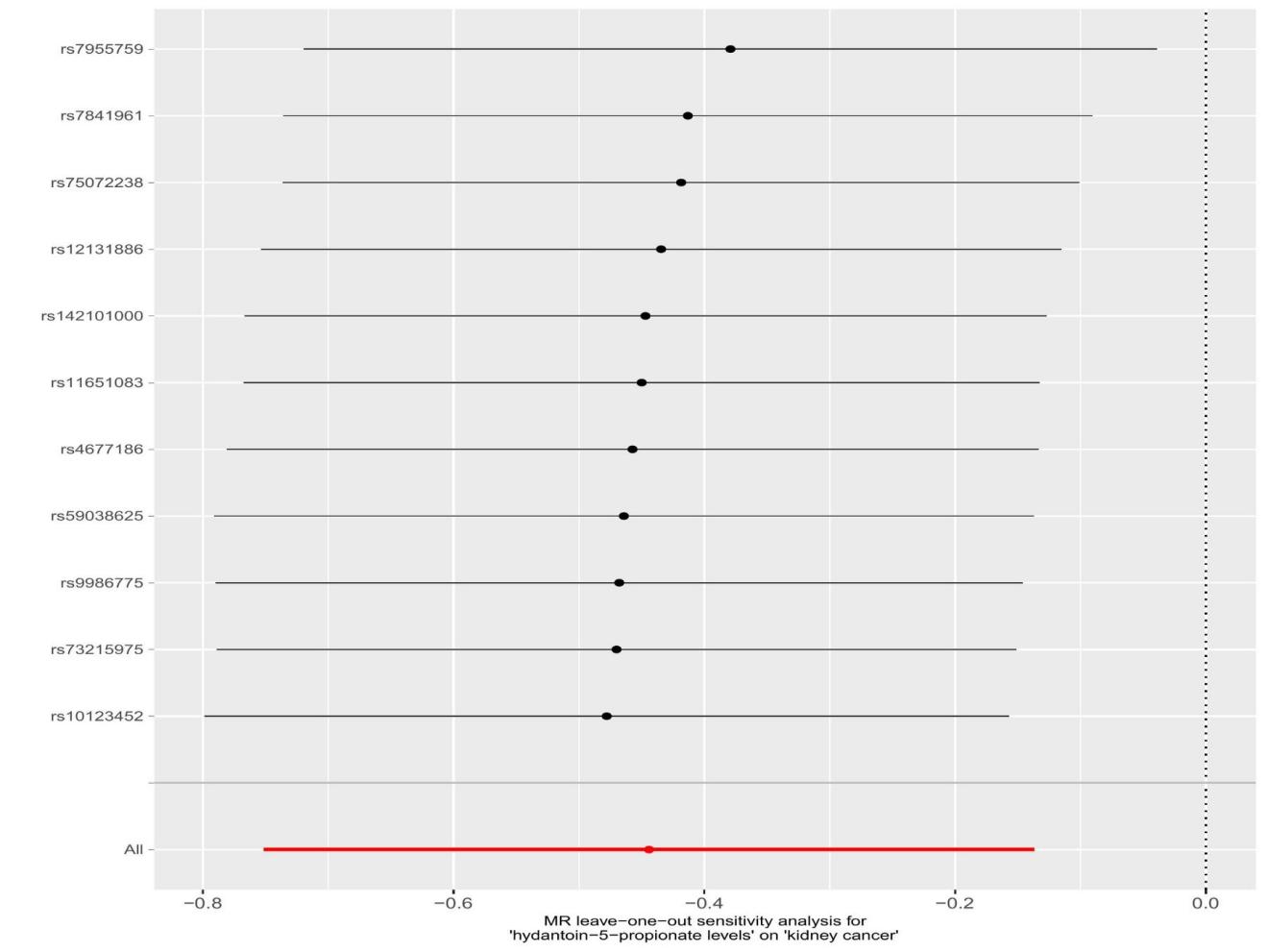


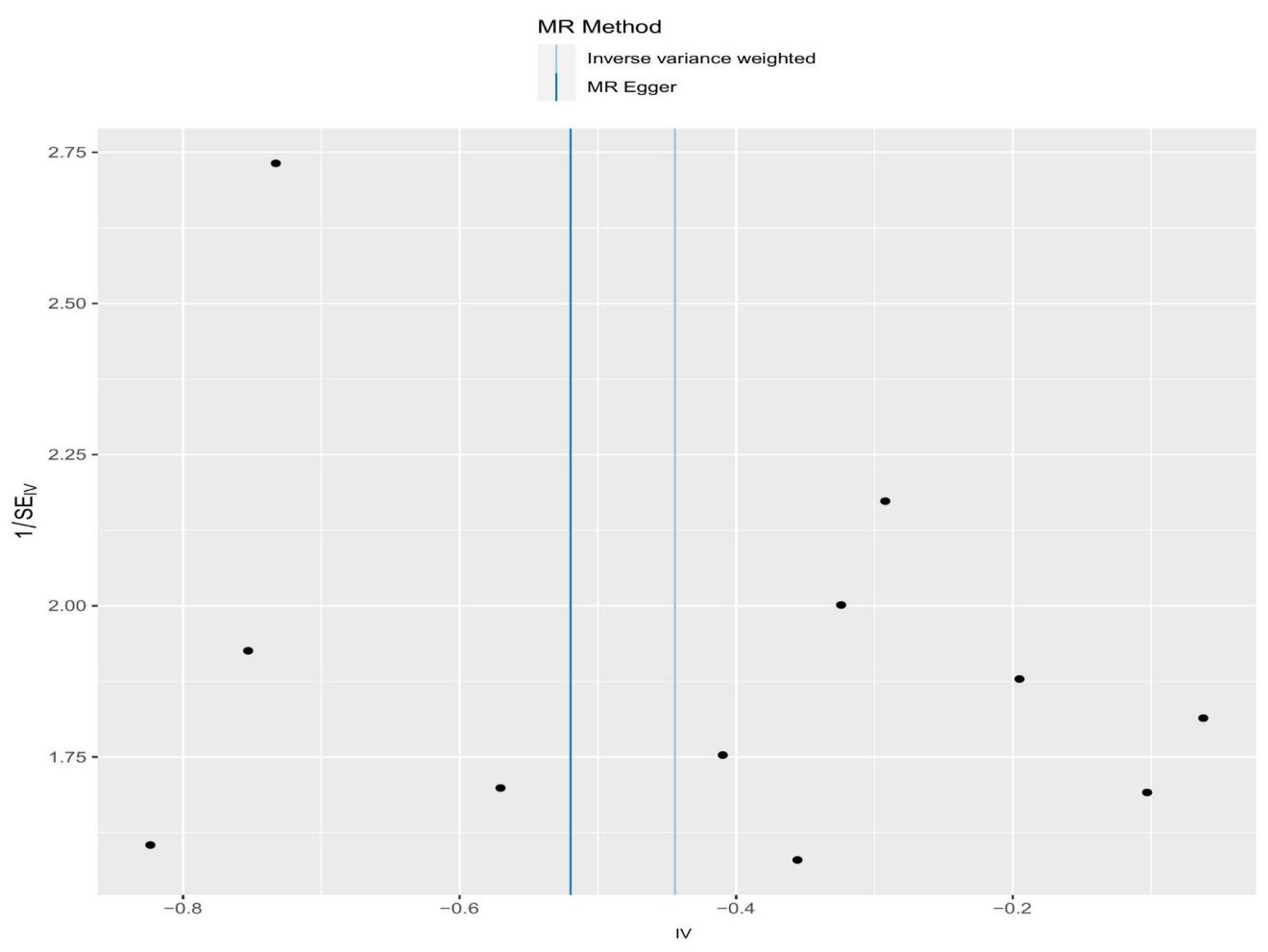


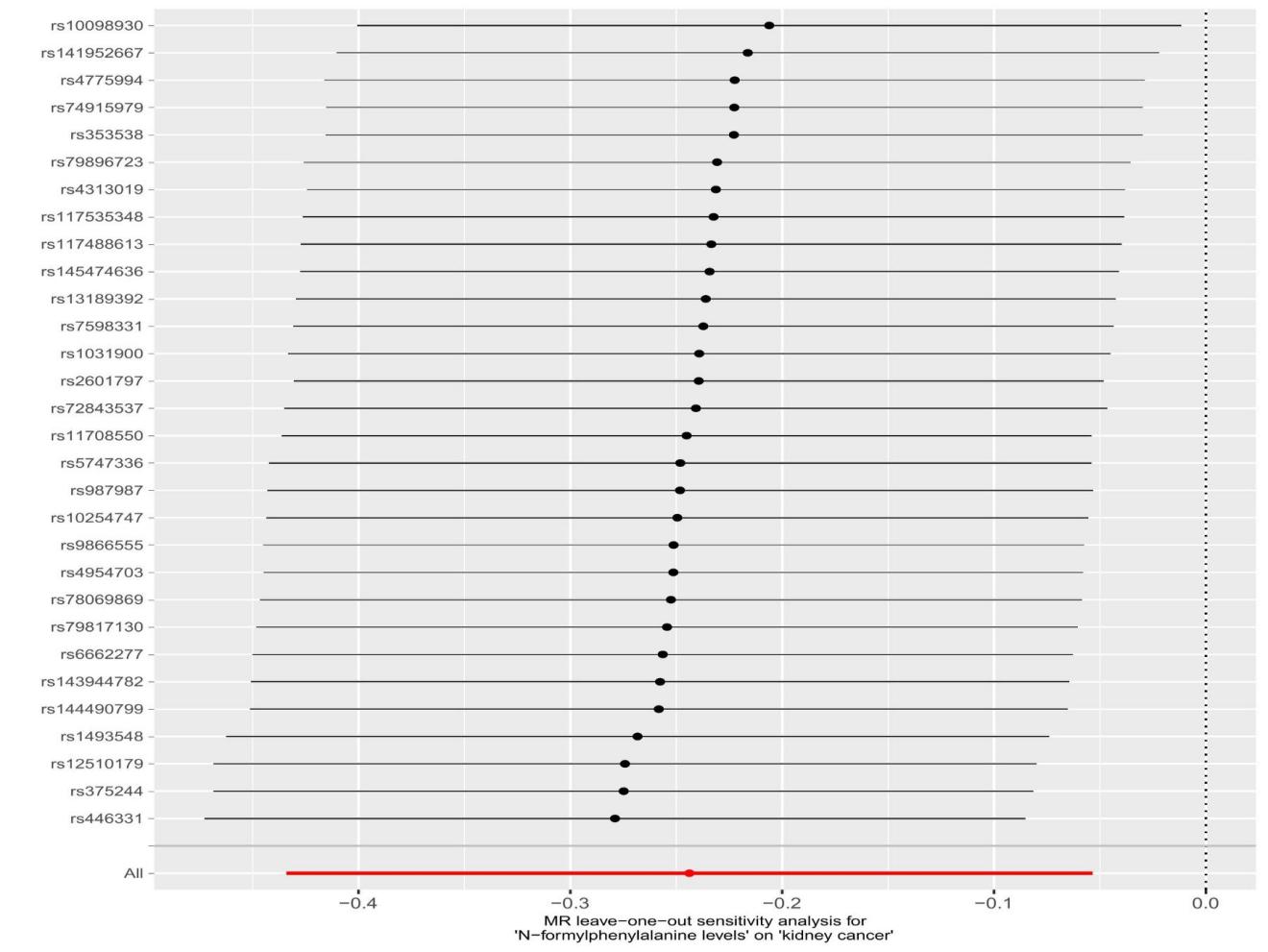


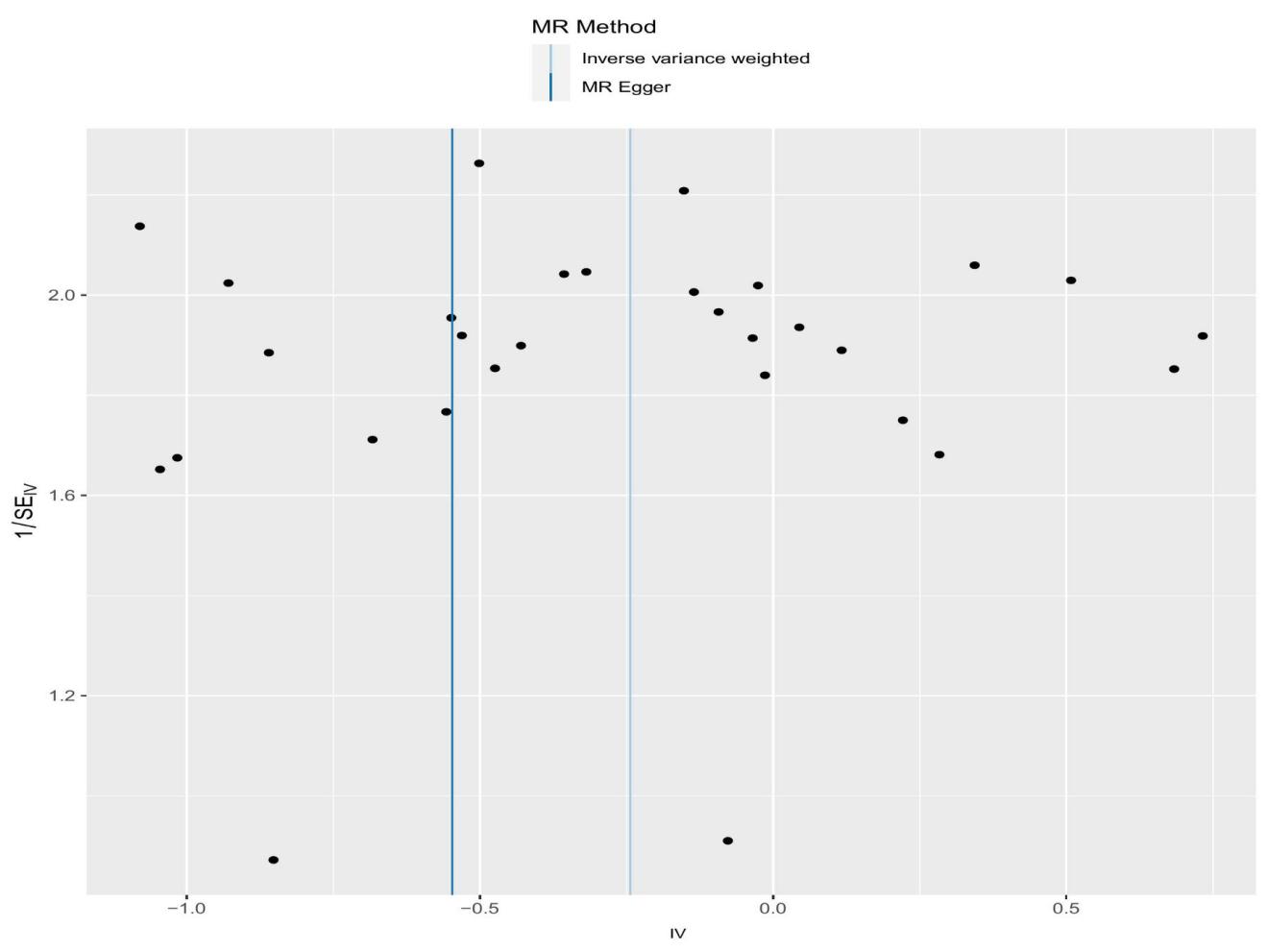


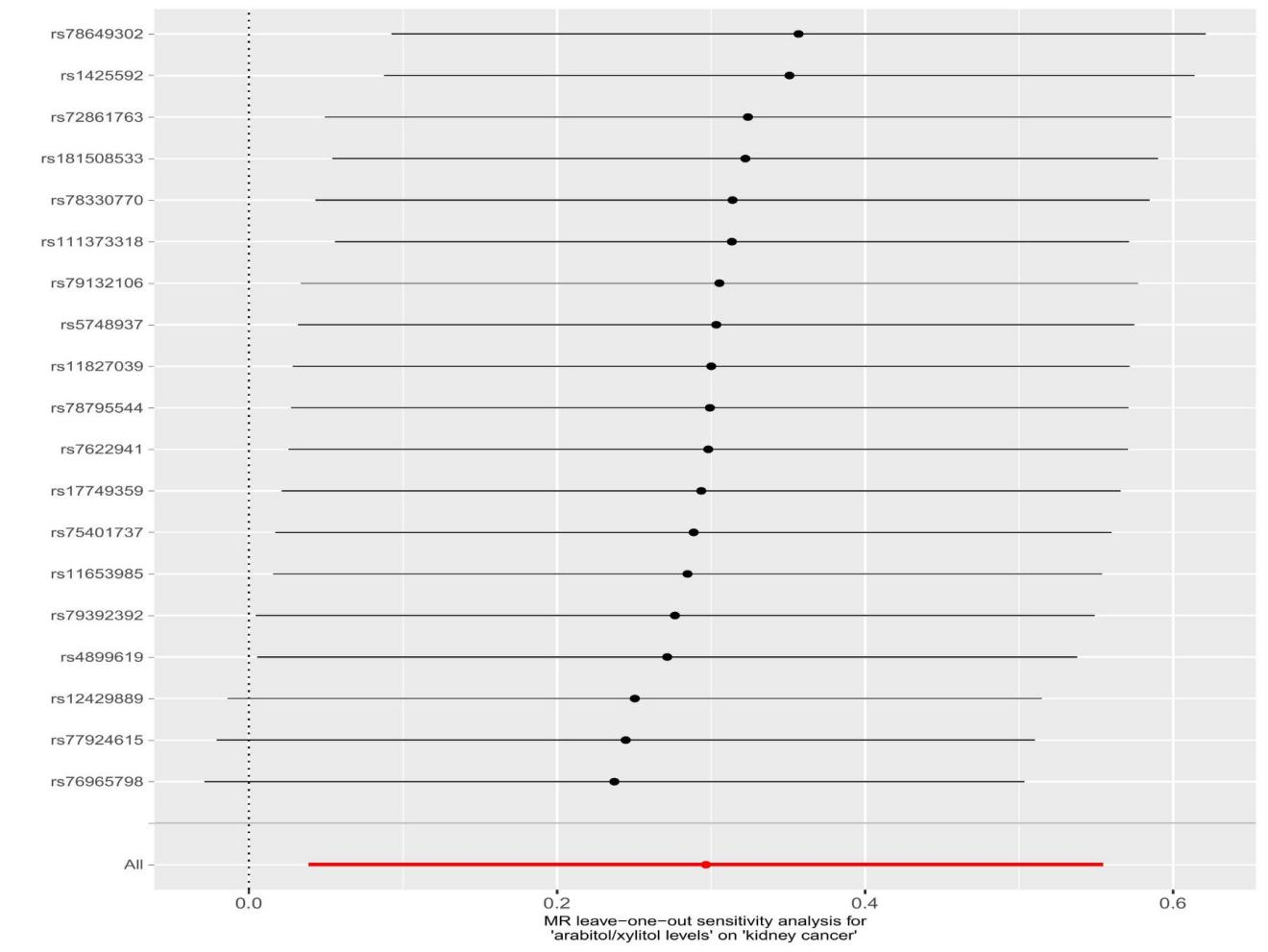


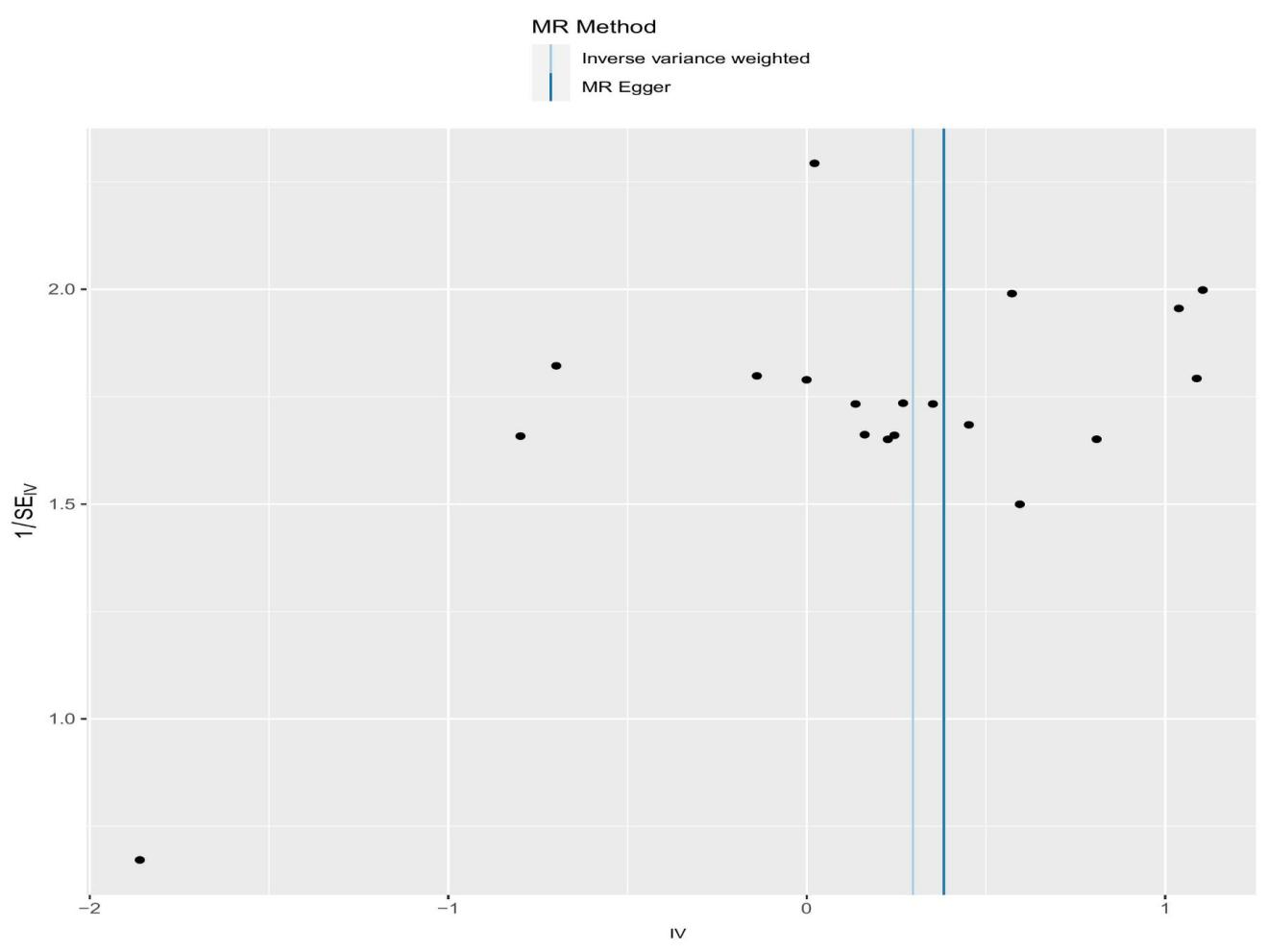


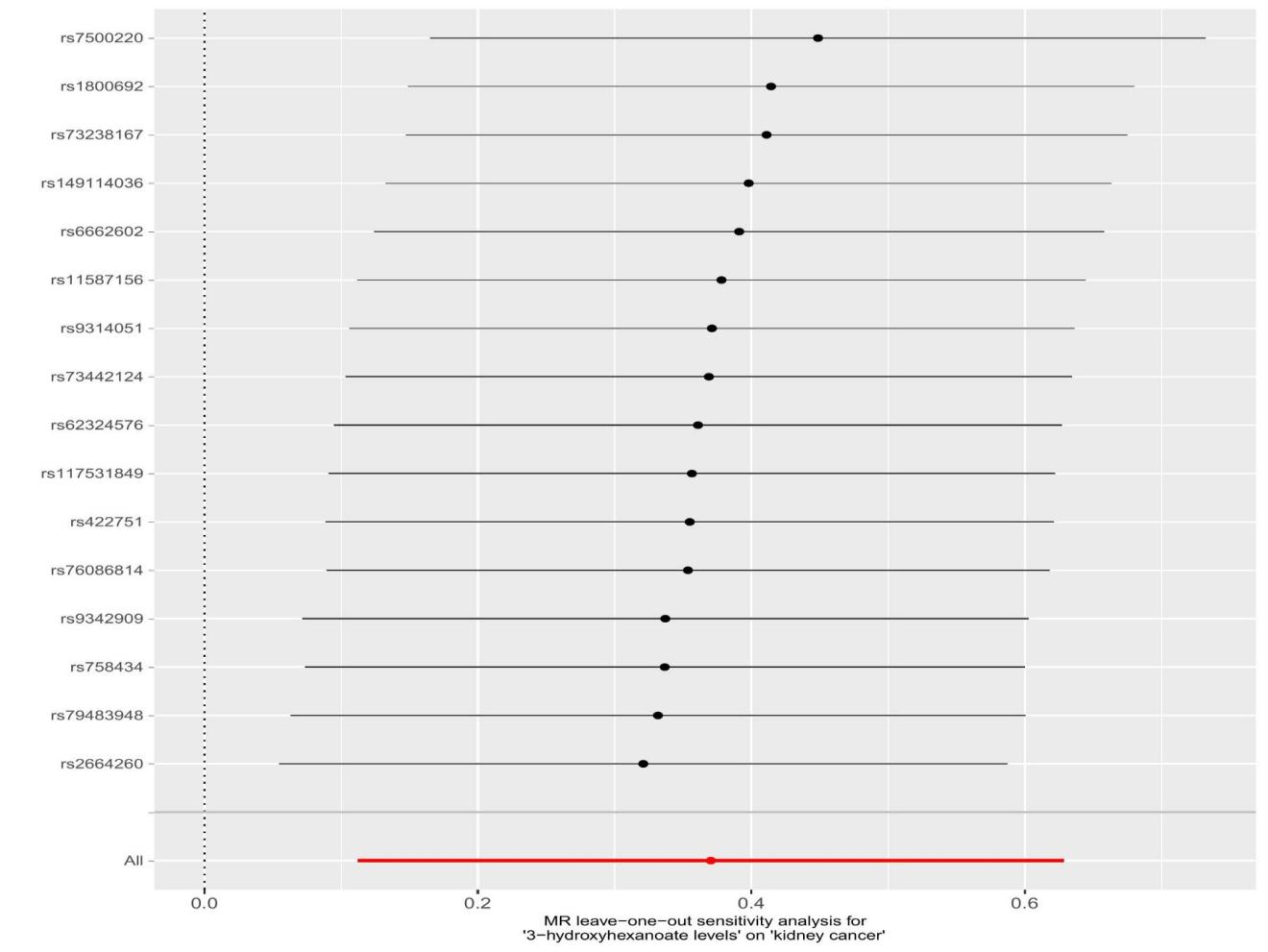


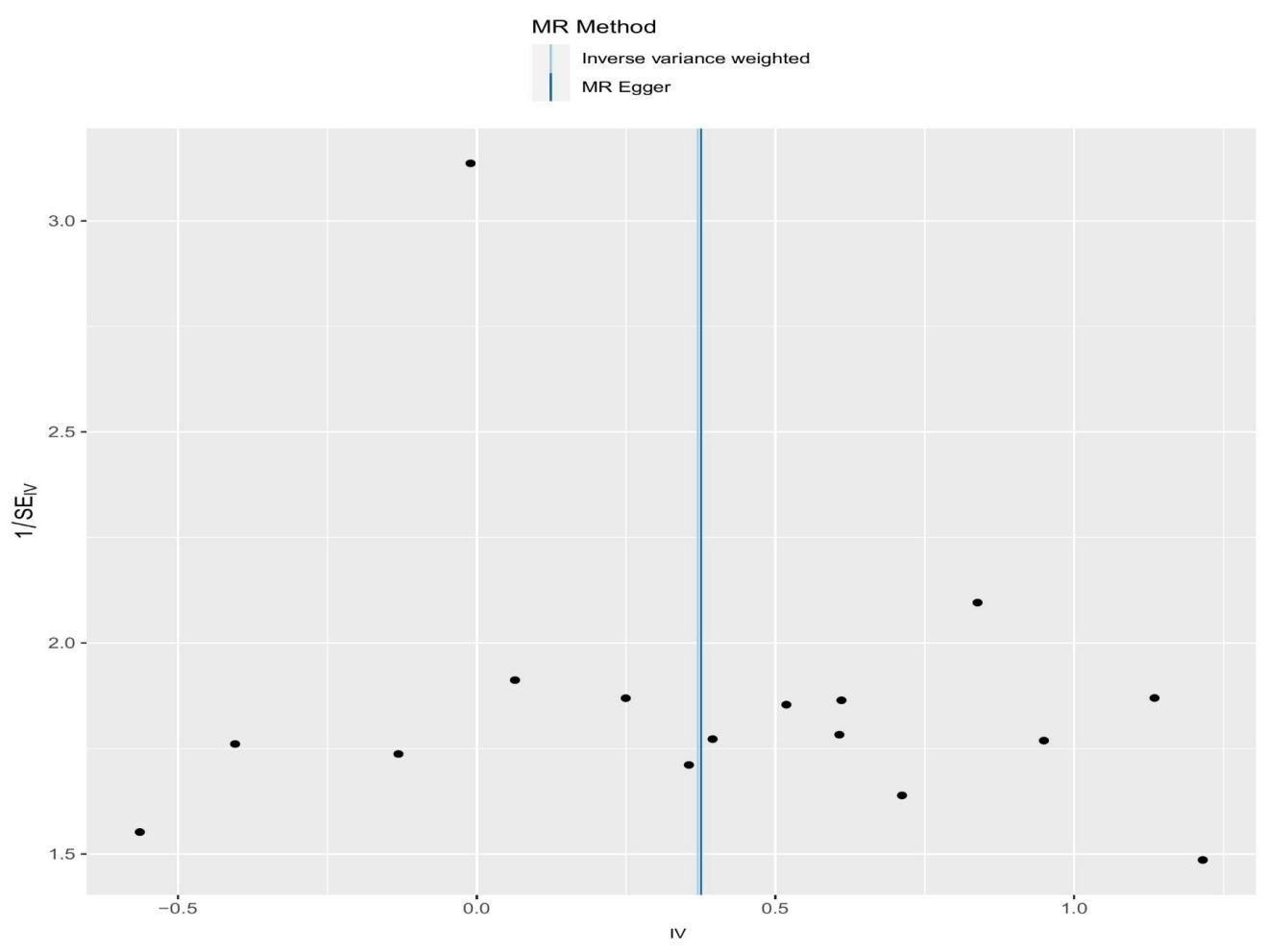


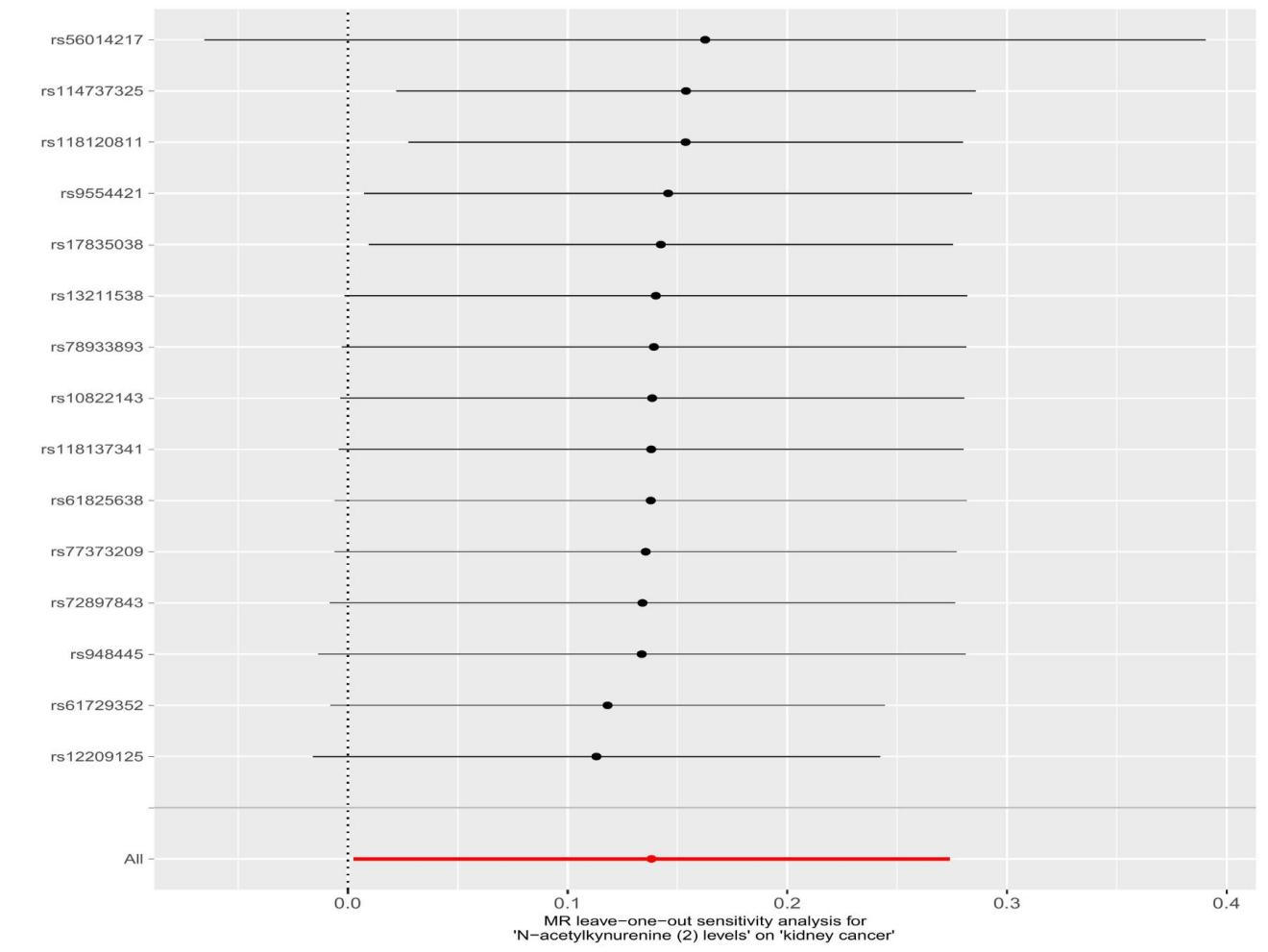


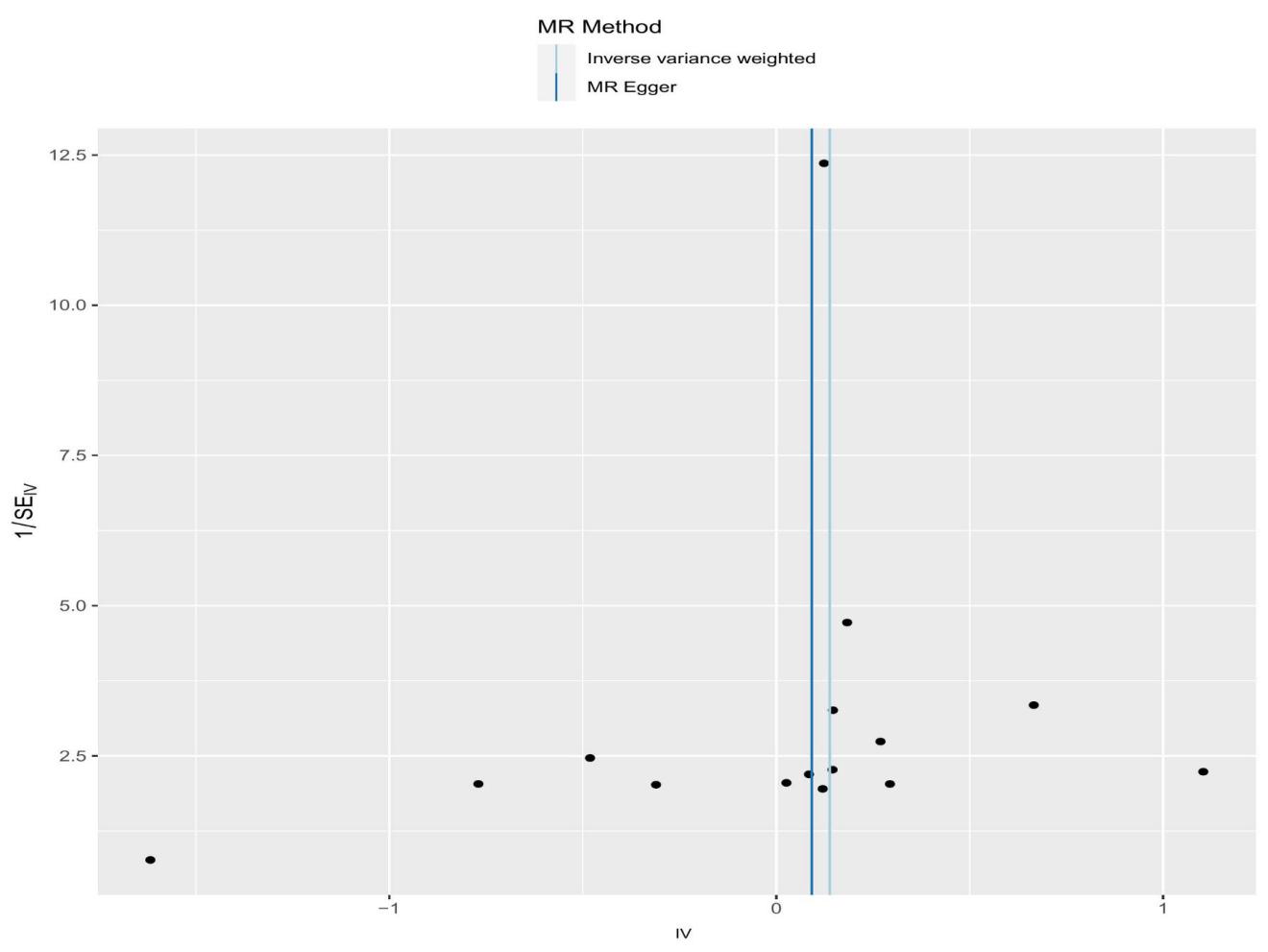


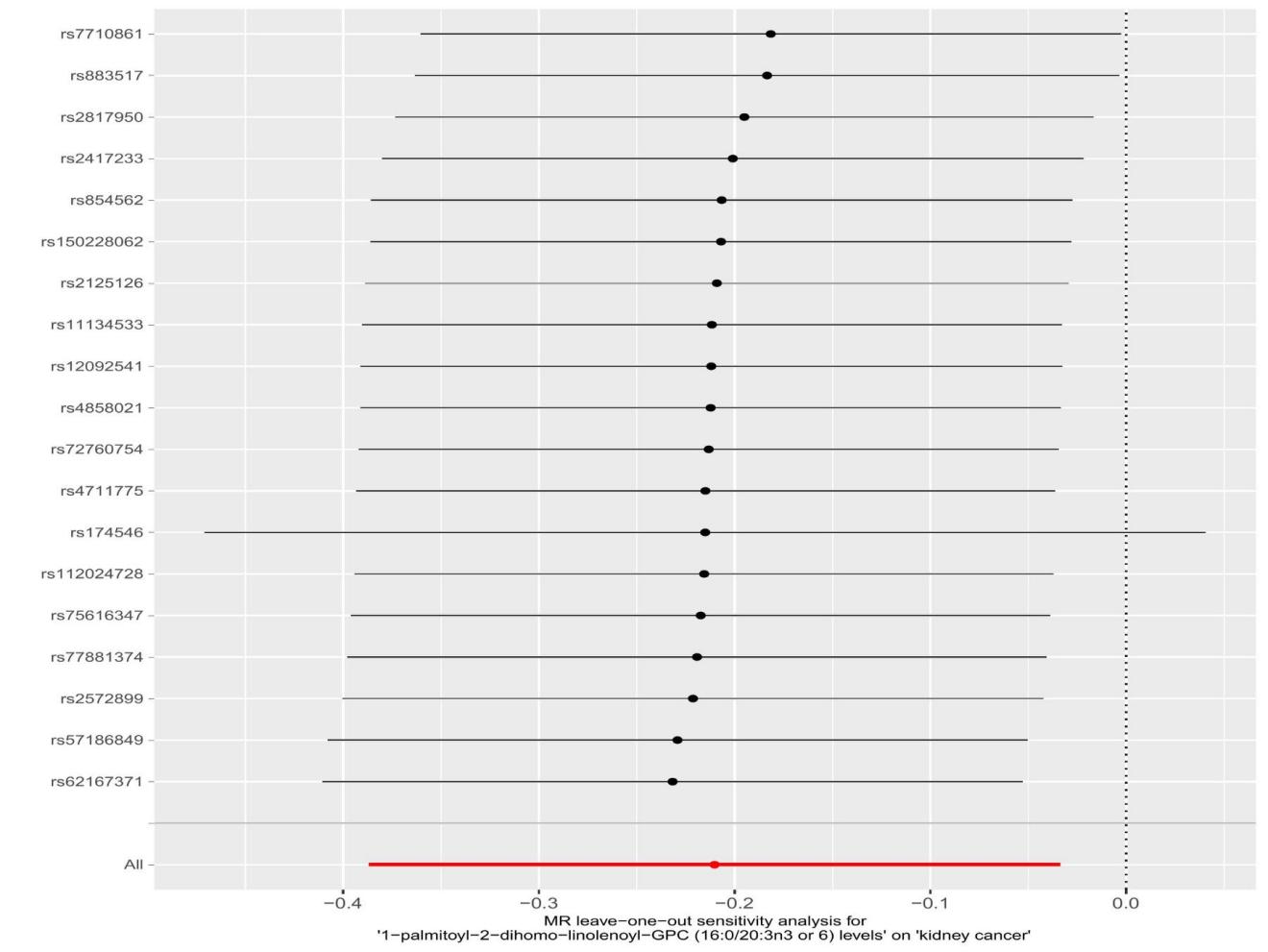


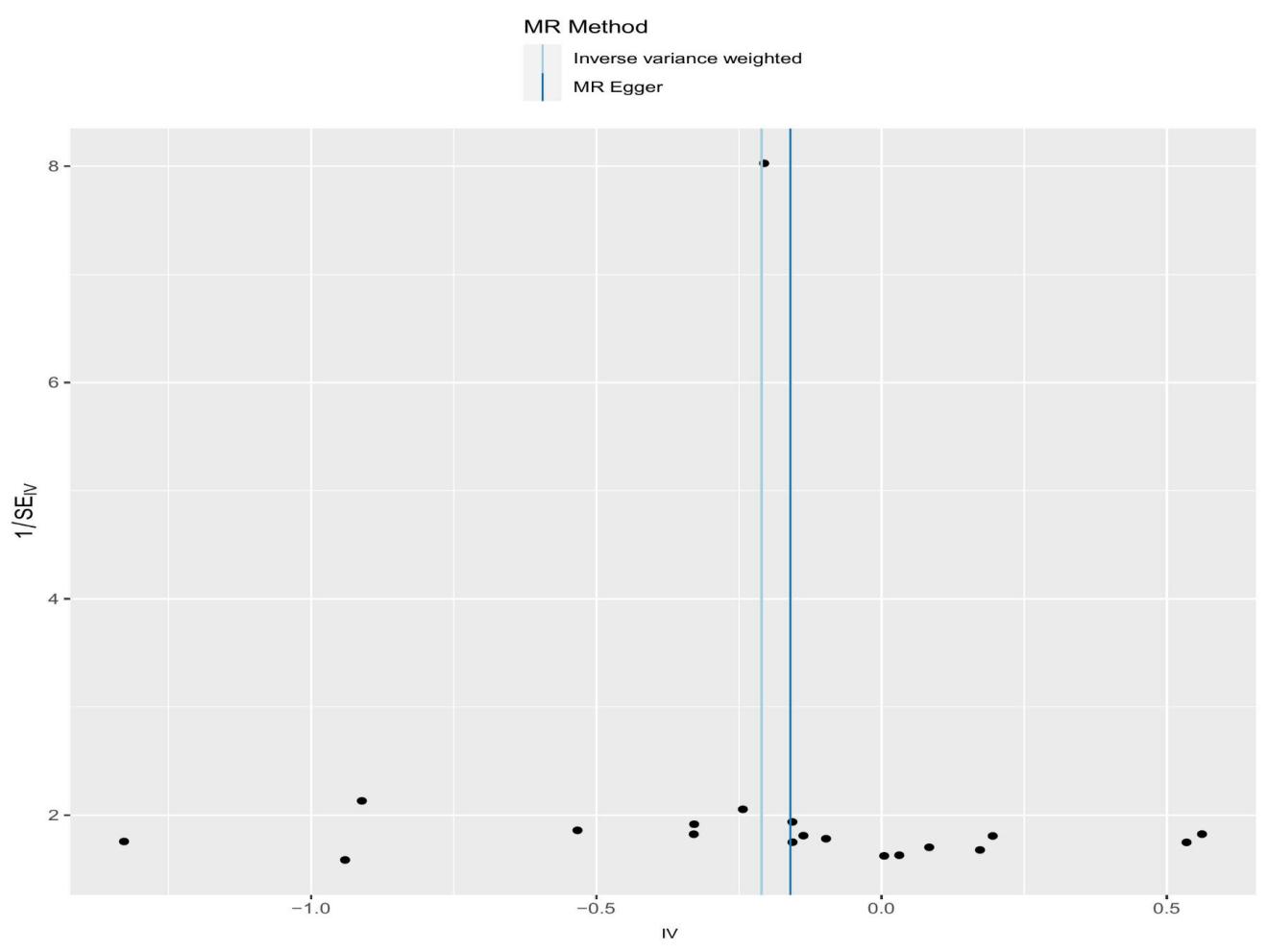


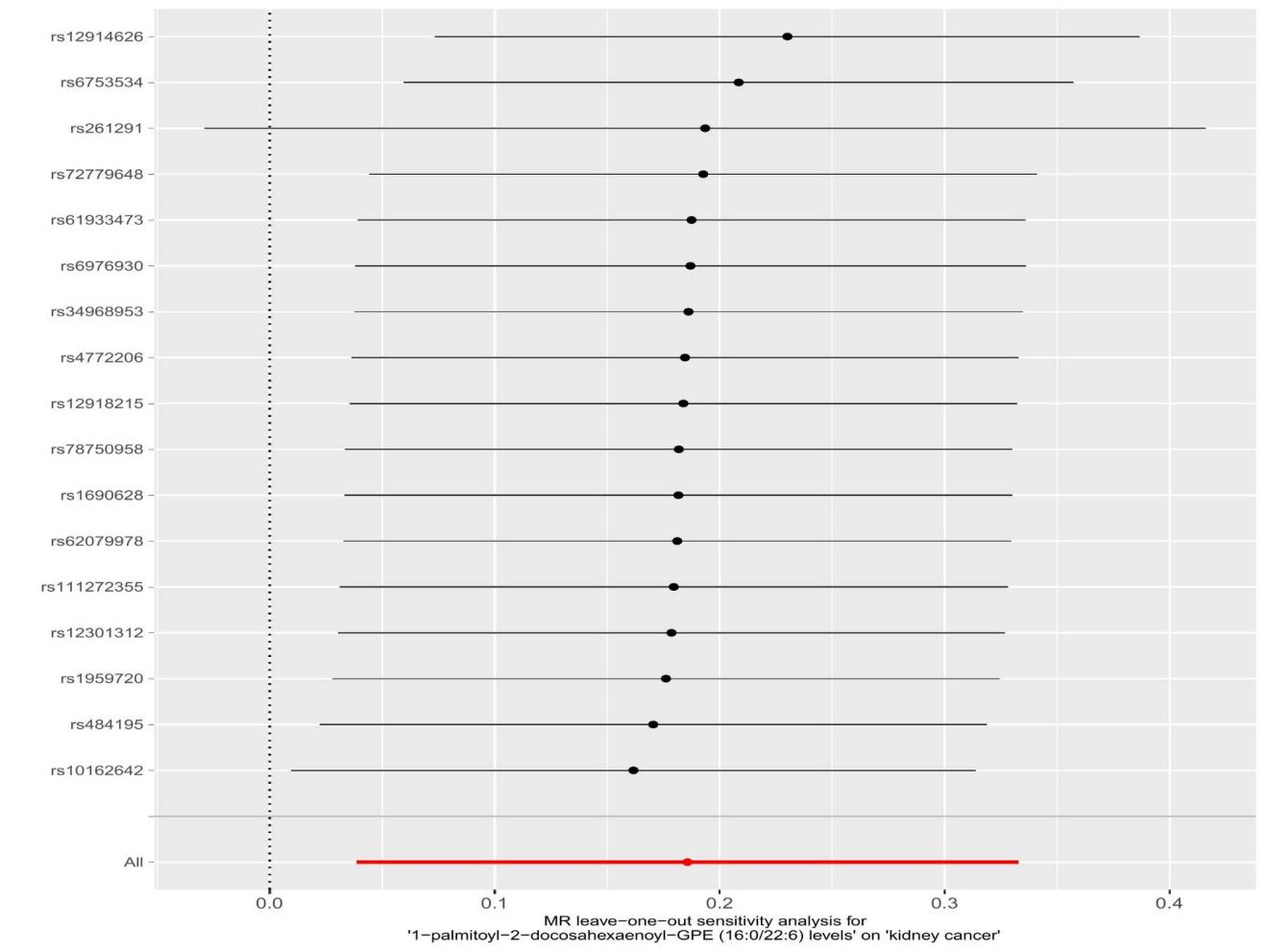


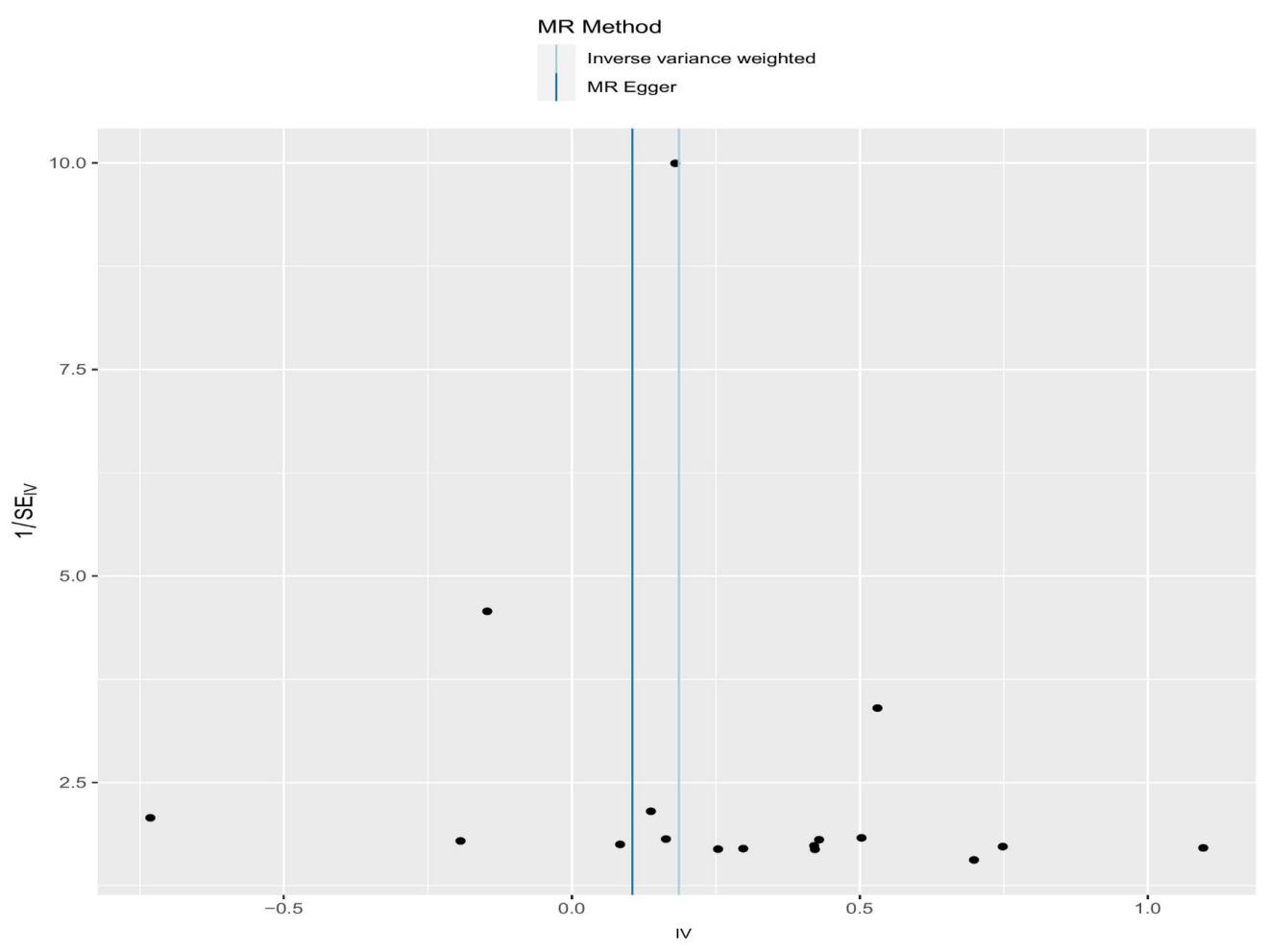


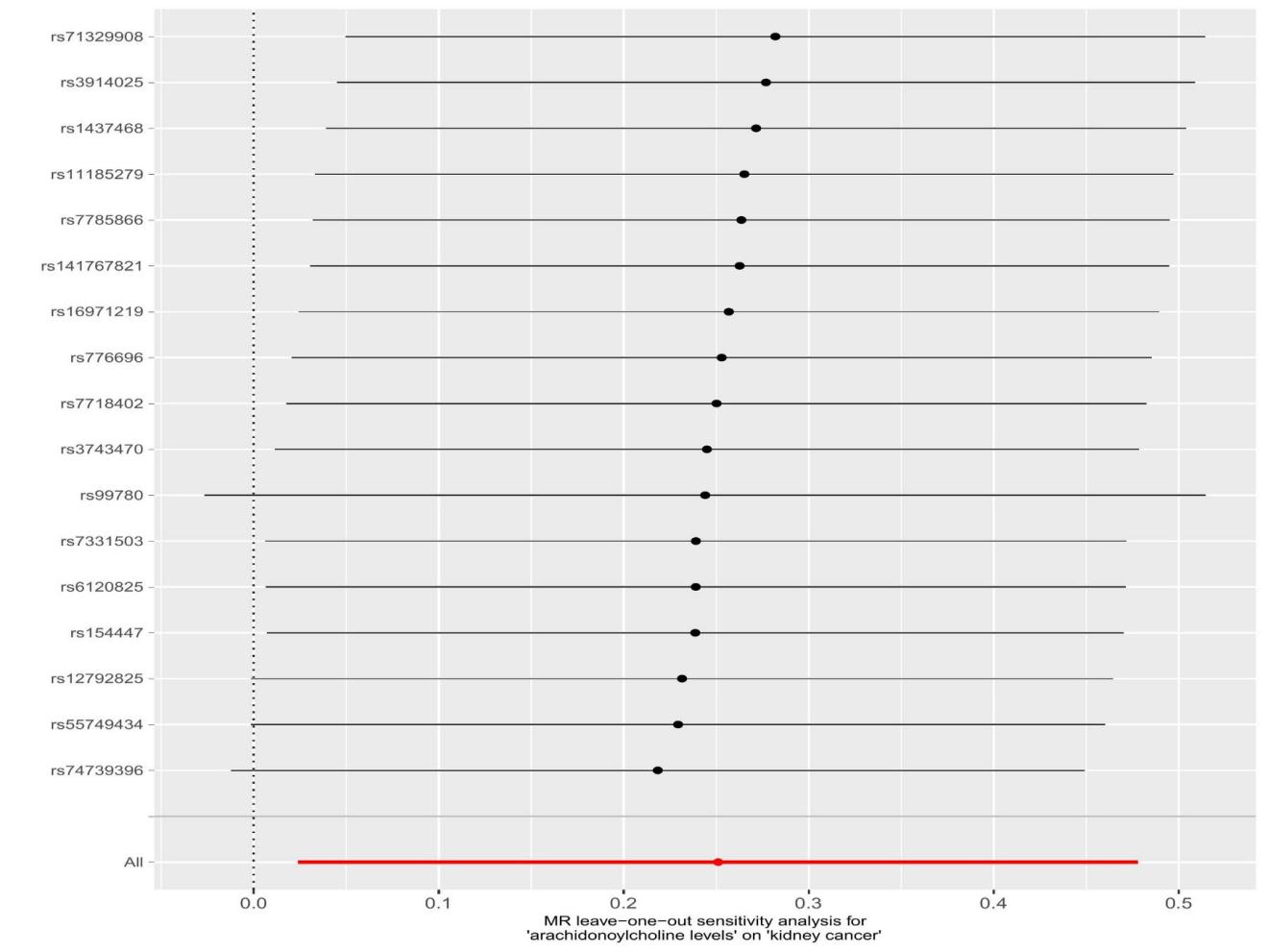


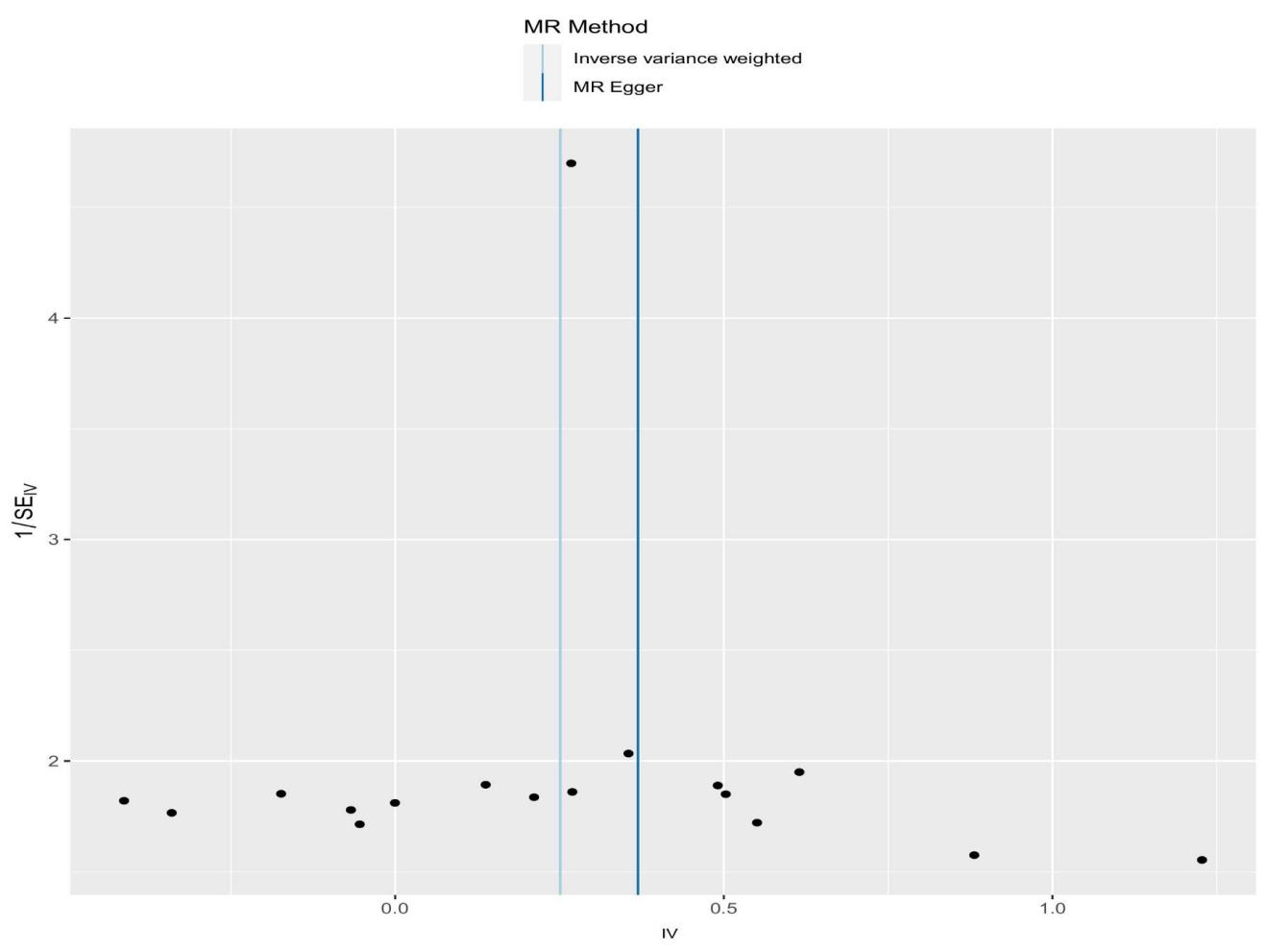


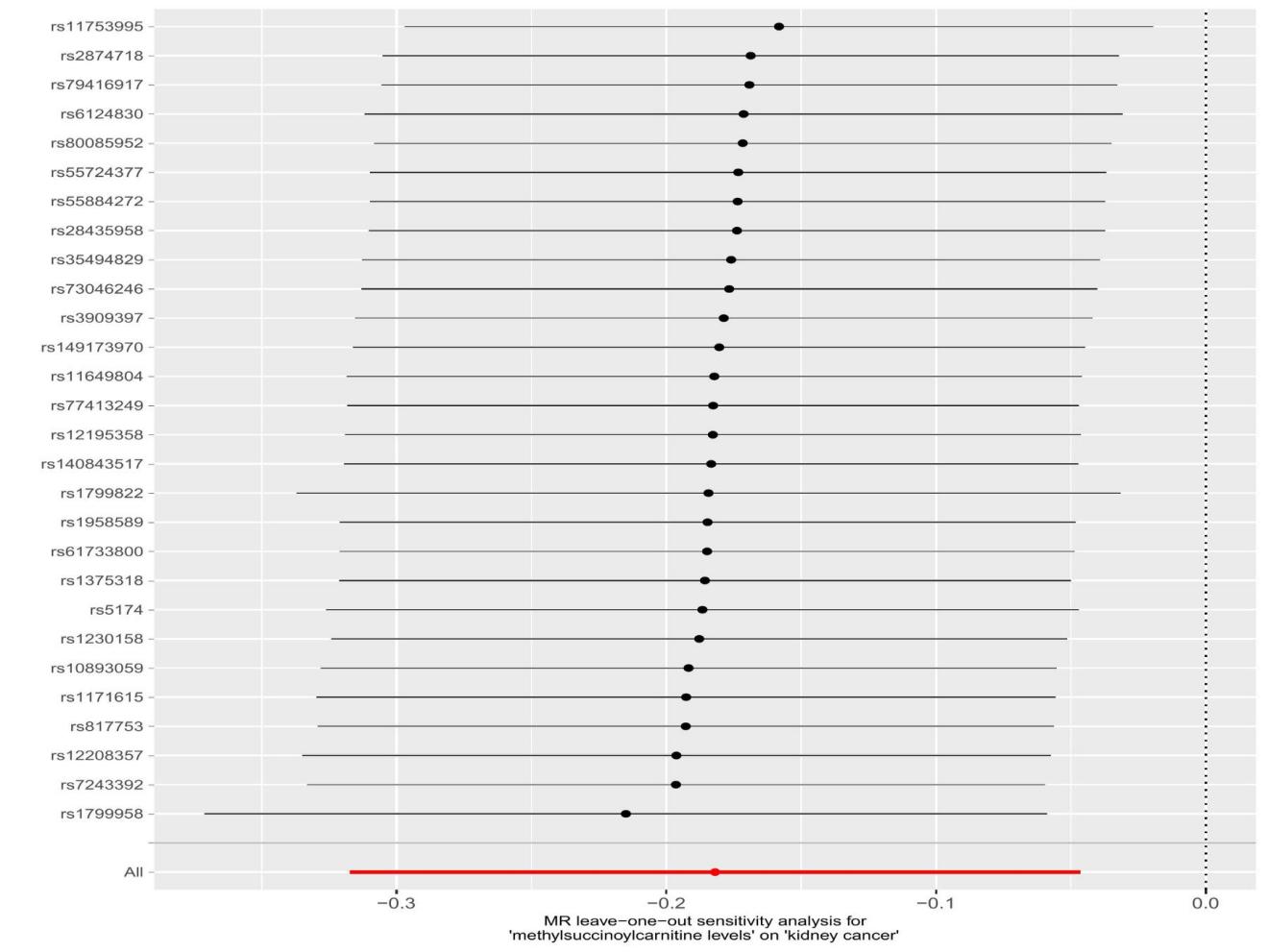


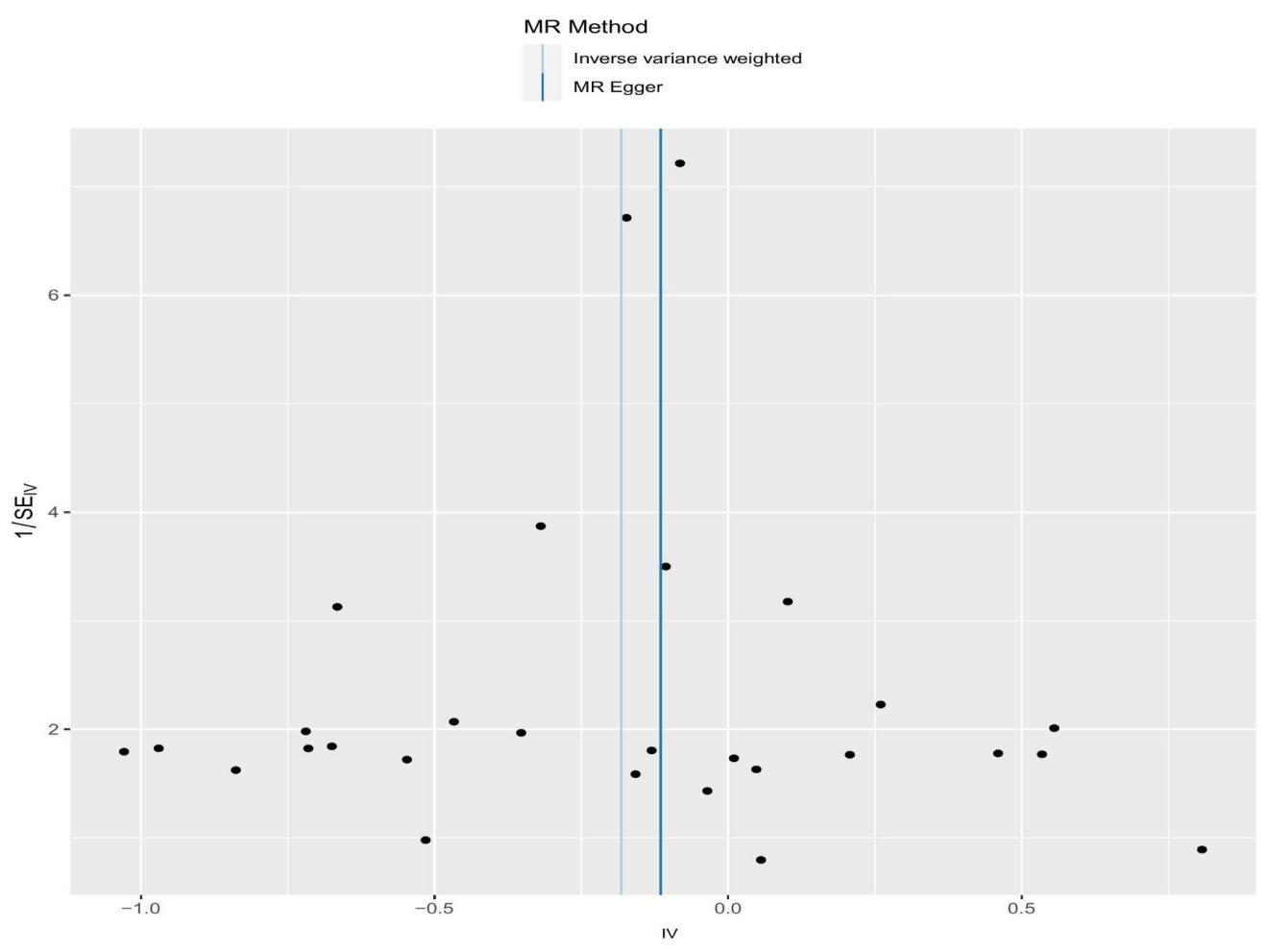


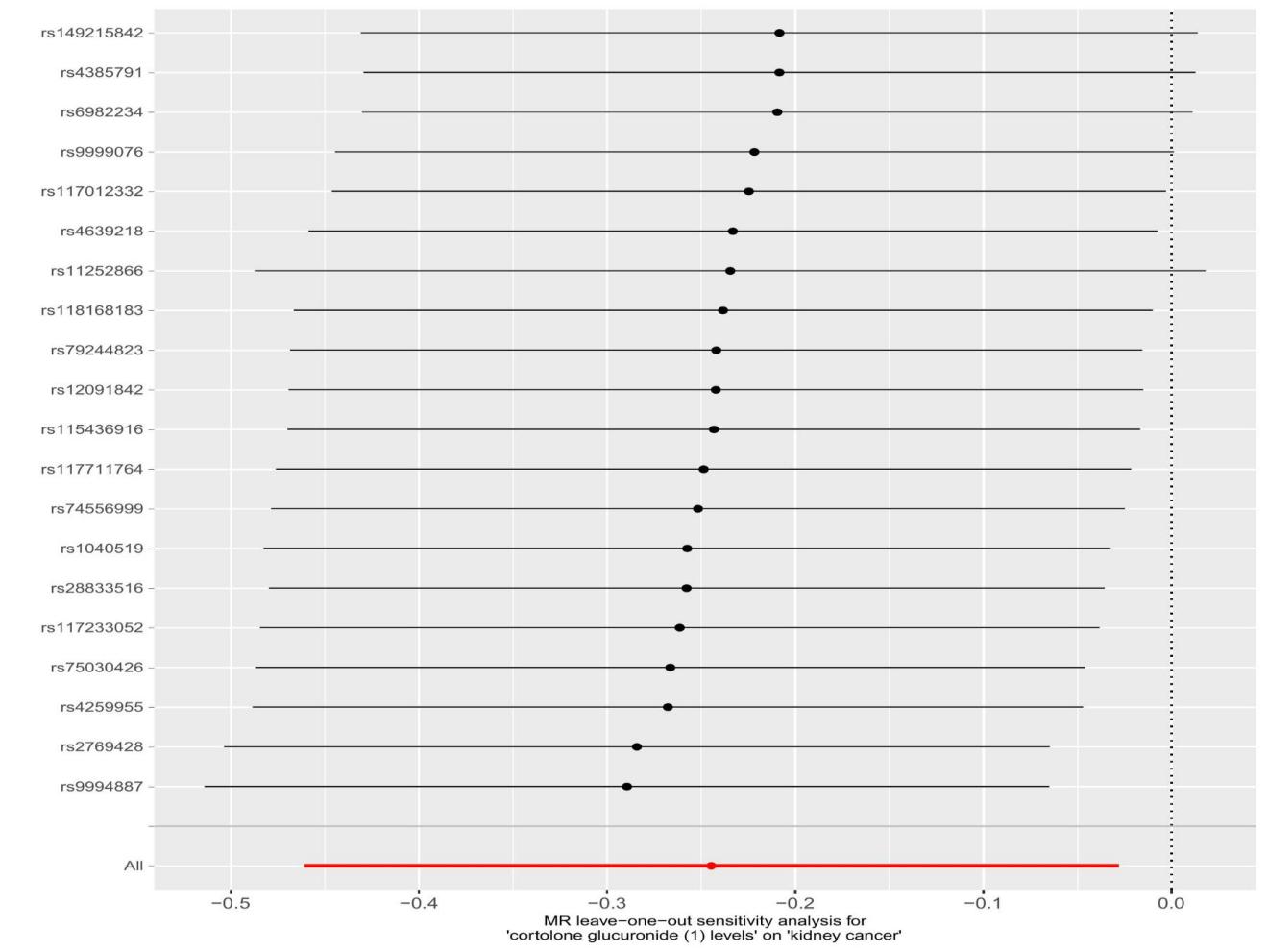


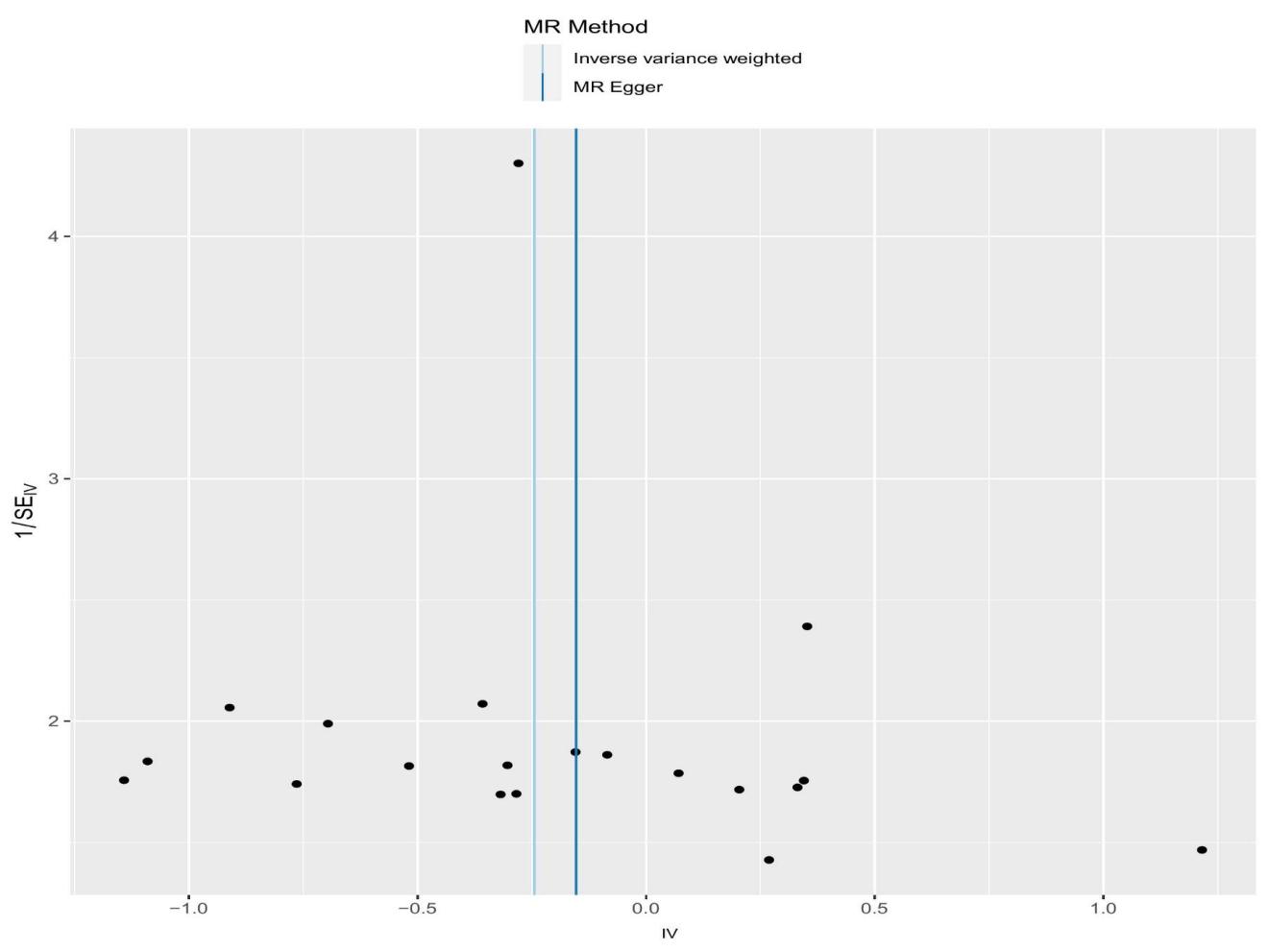


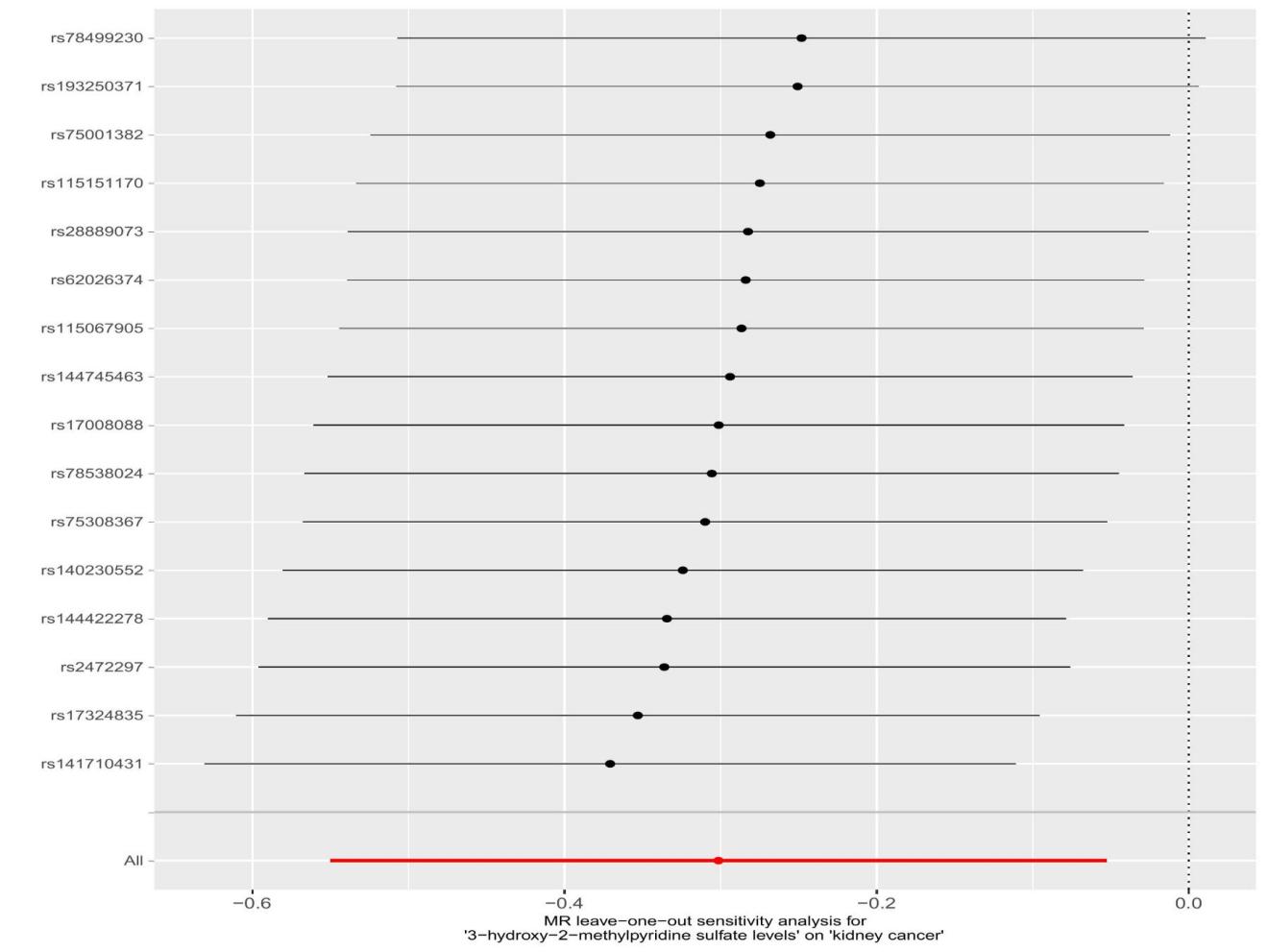


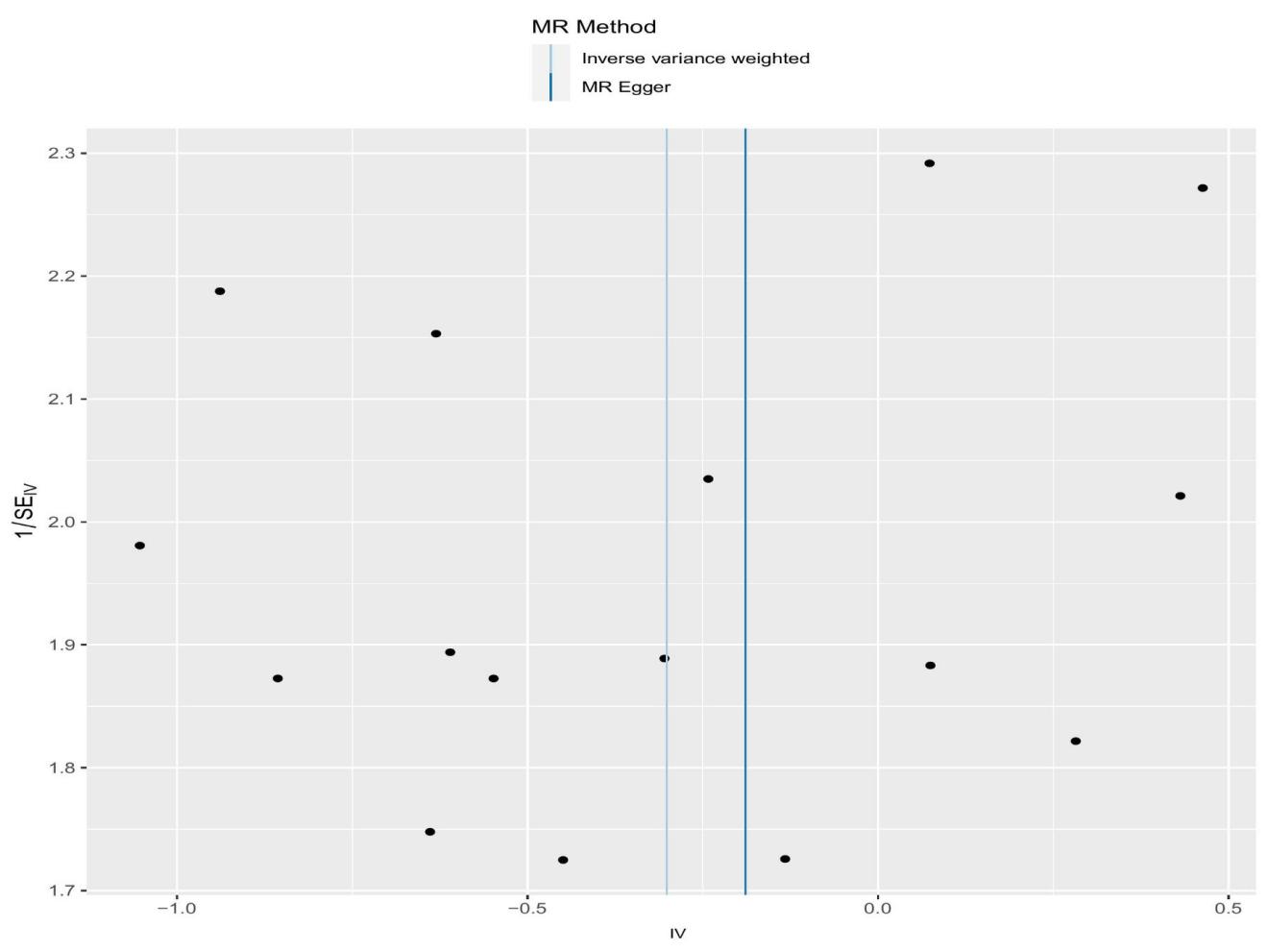


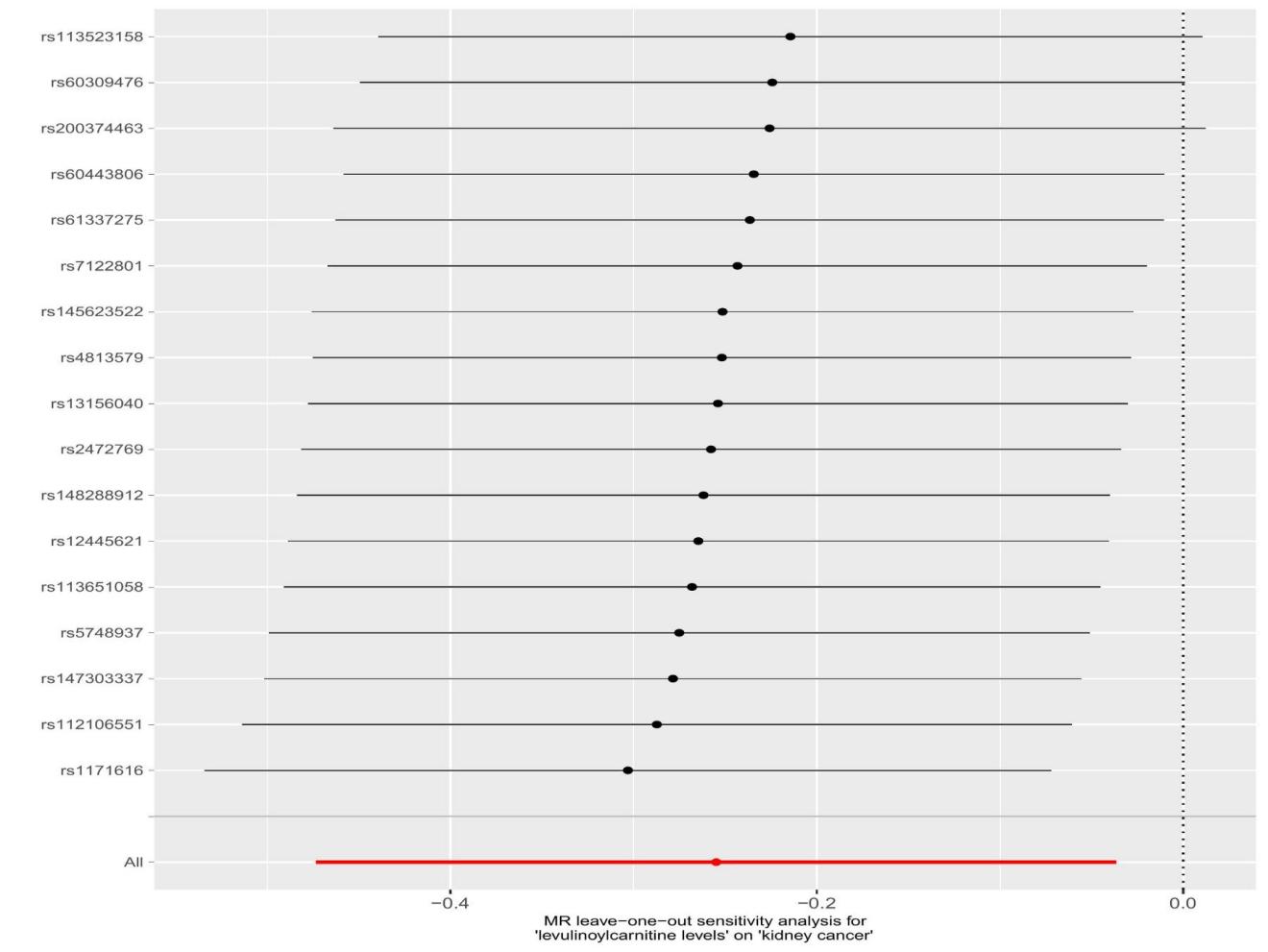


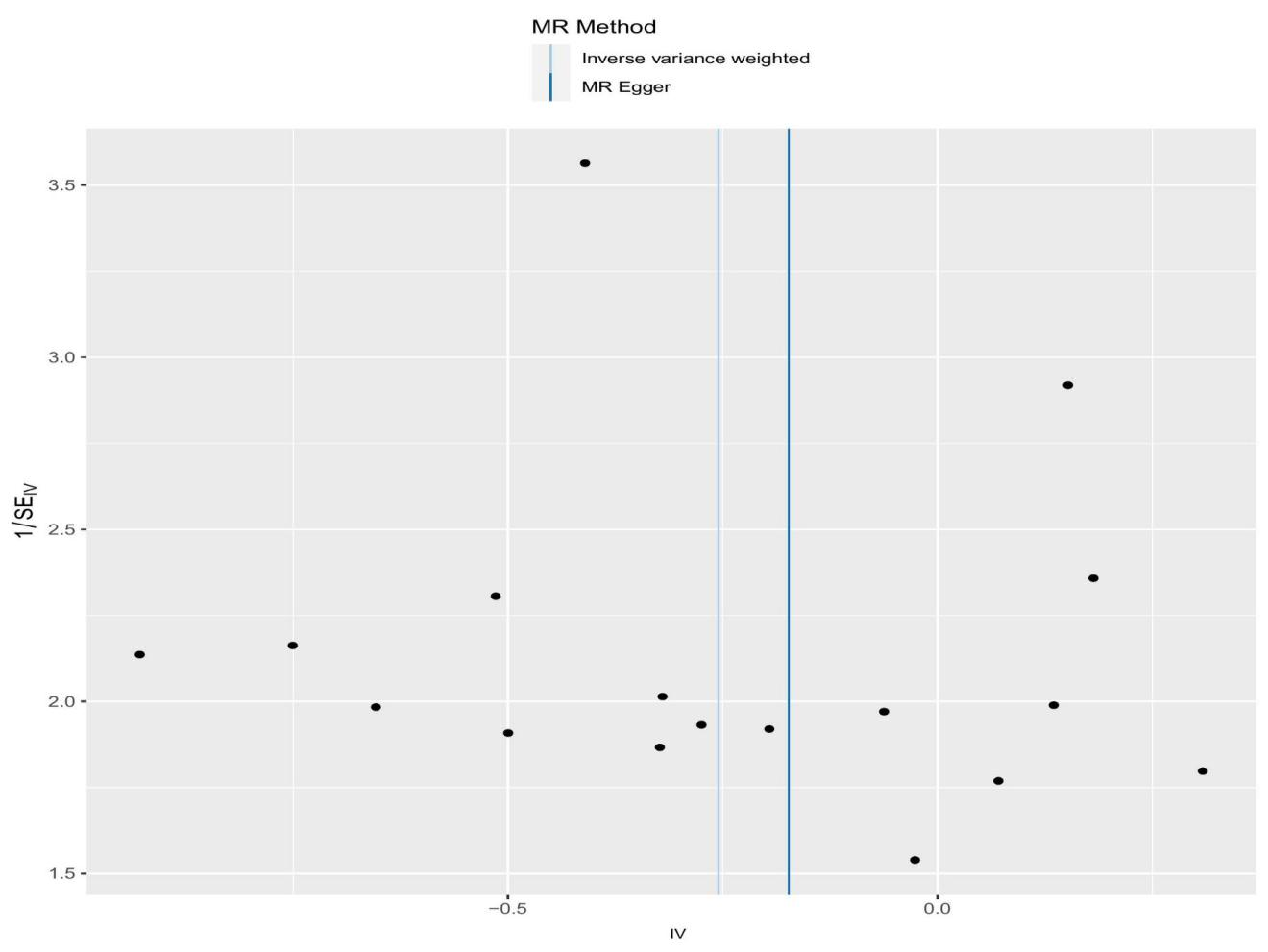


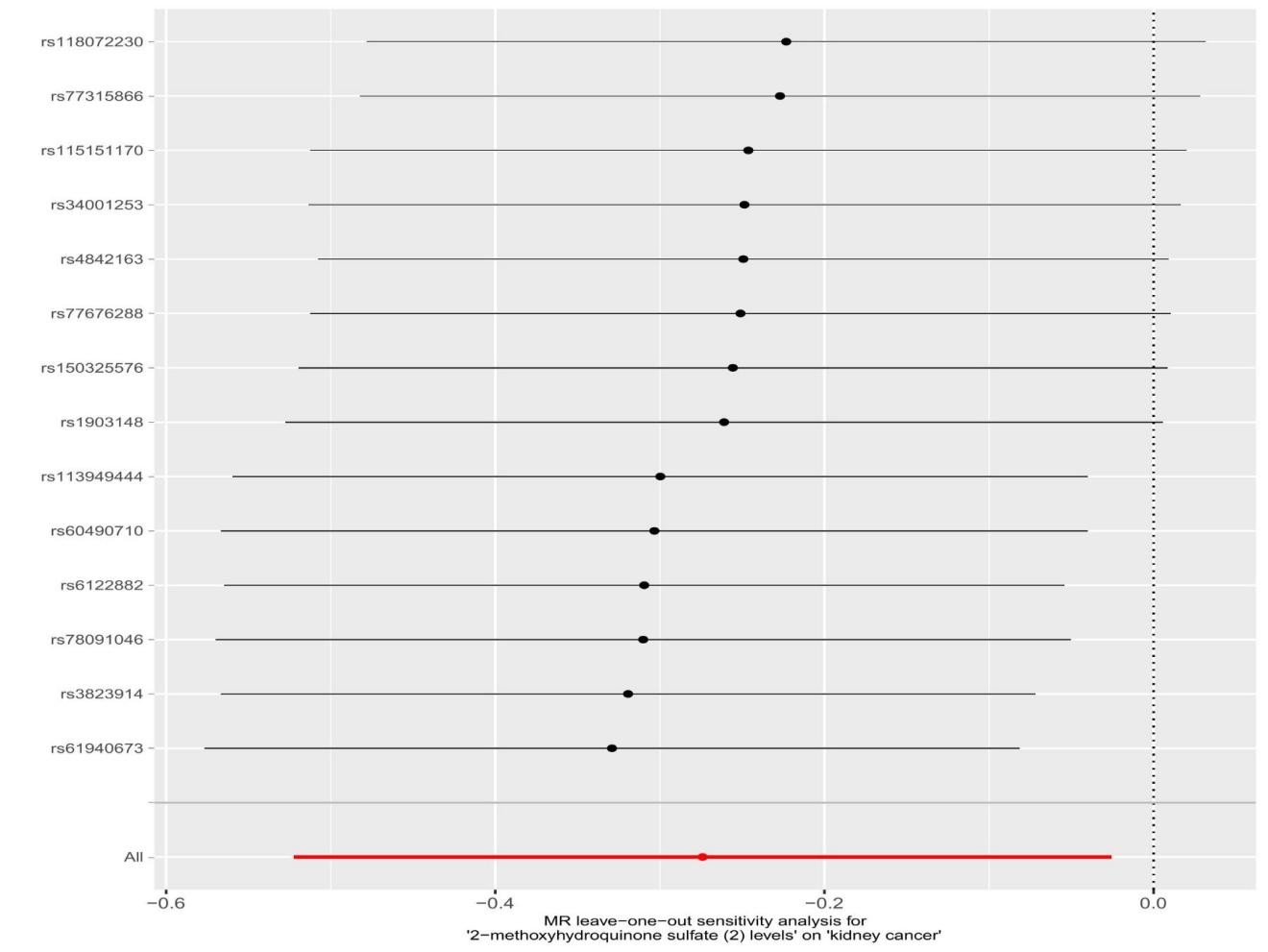


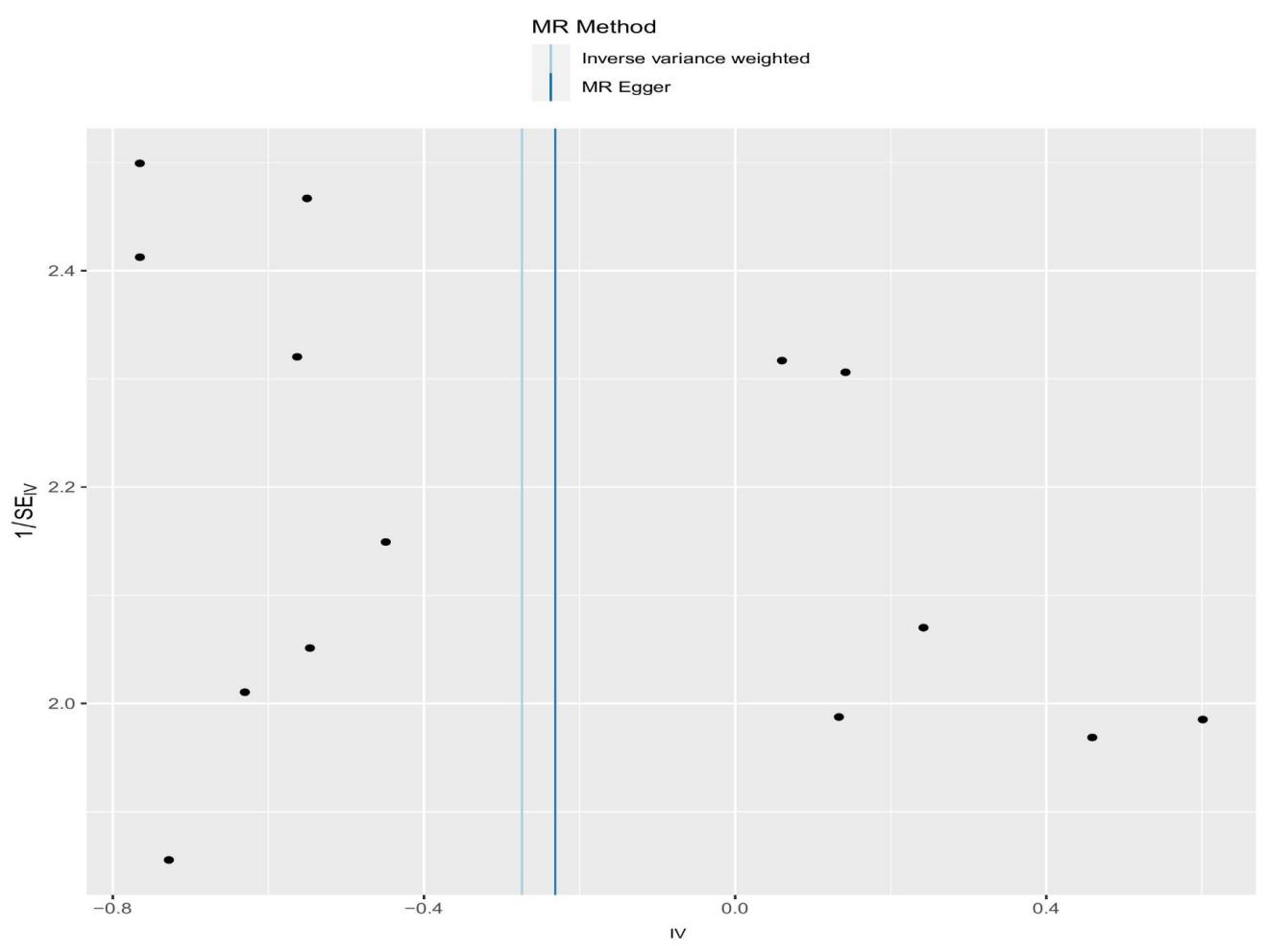


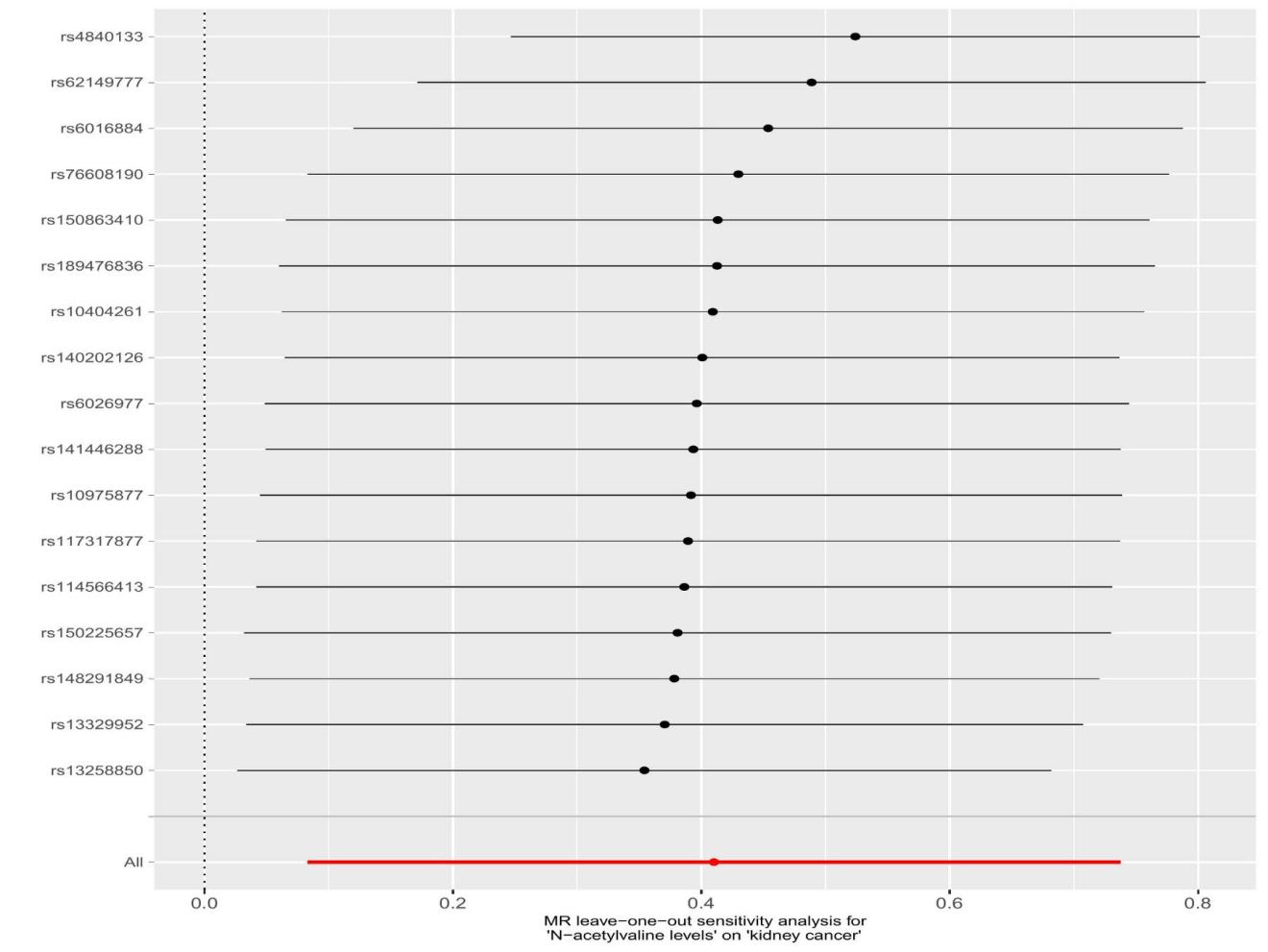


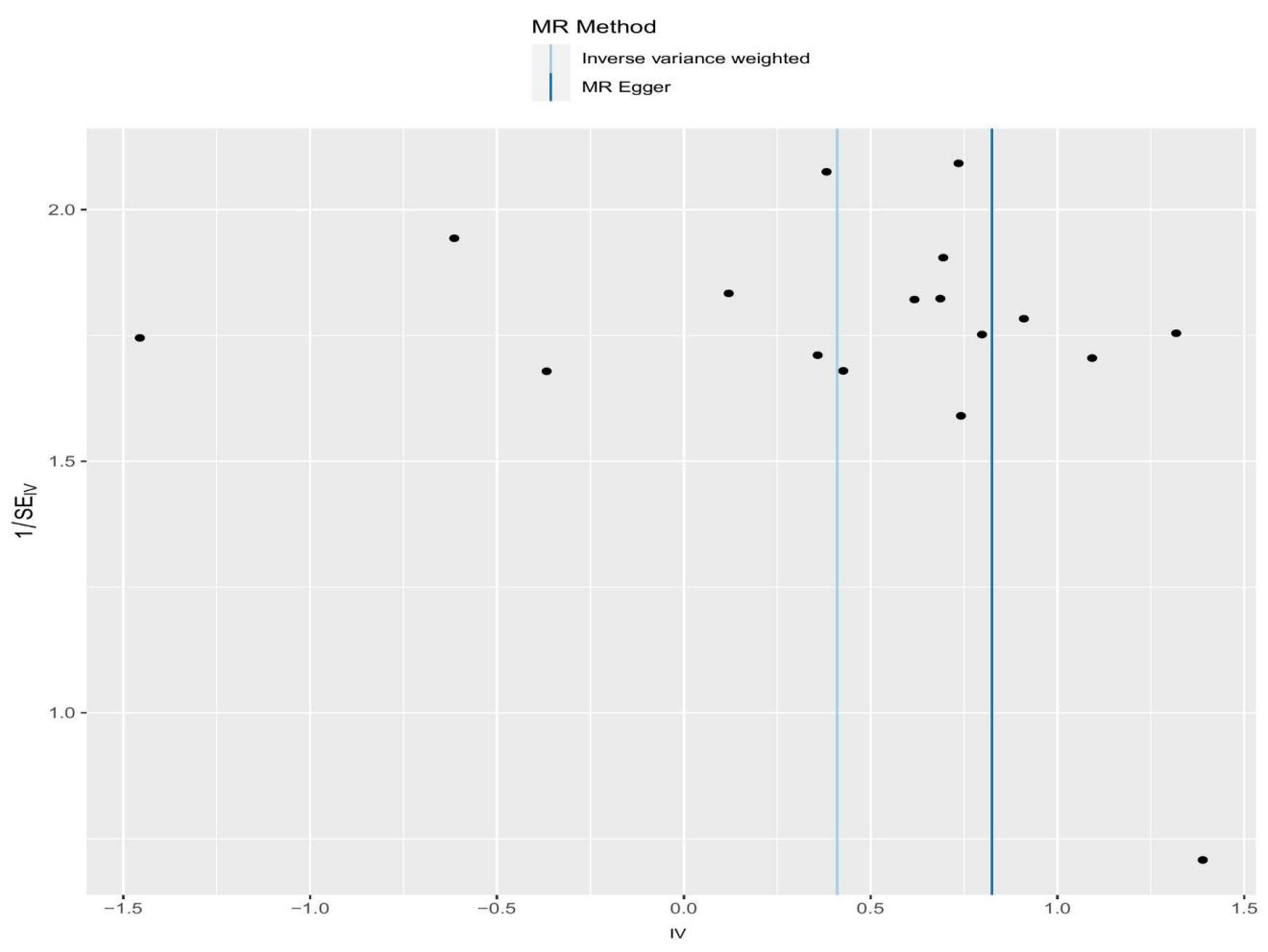


Prostate cancer


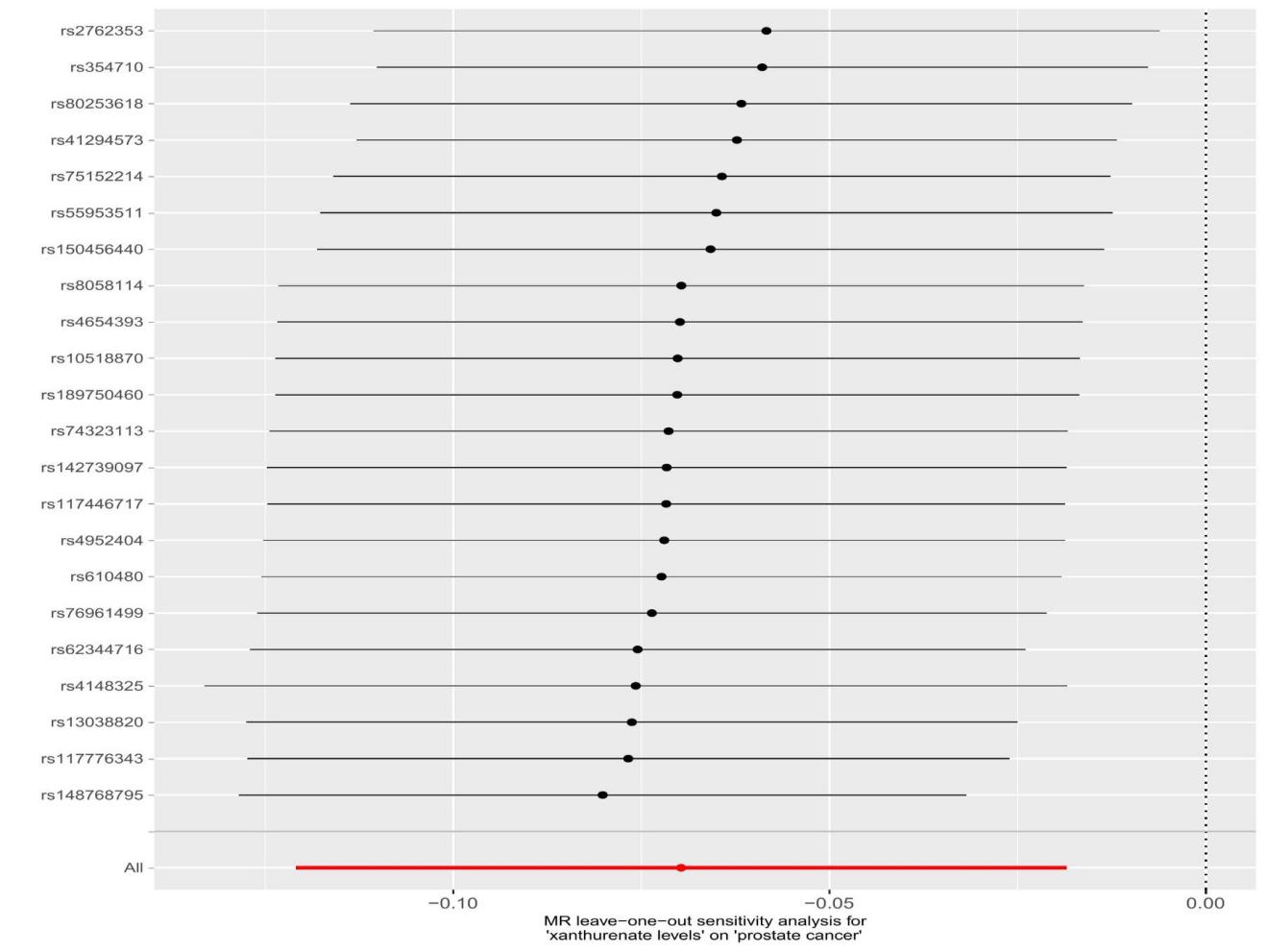


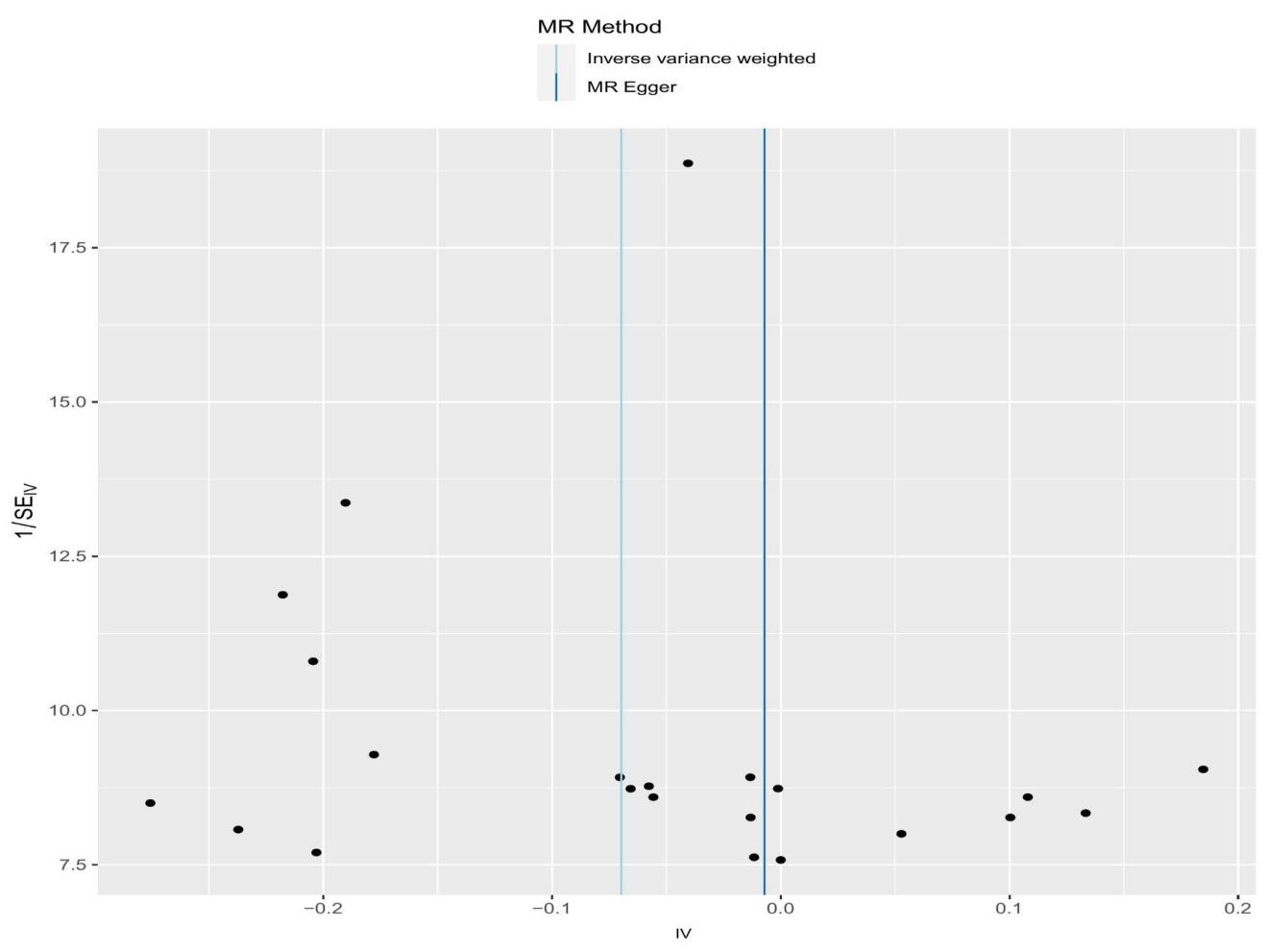


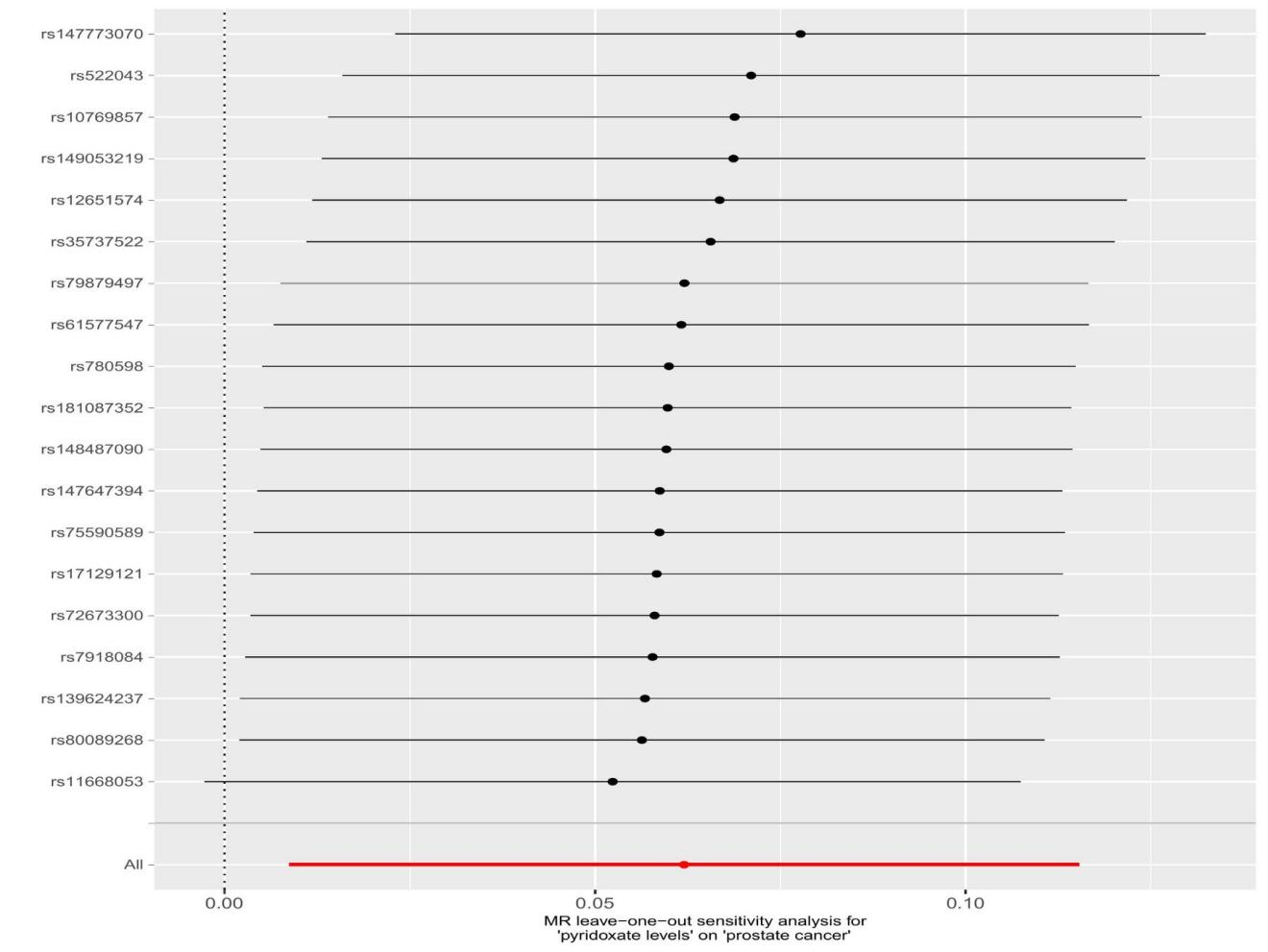


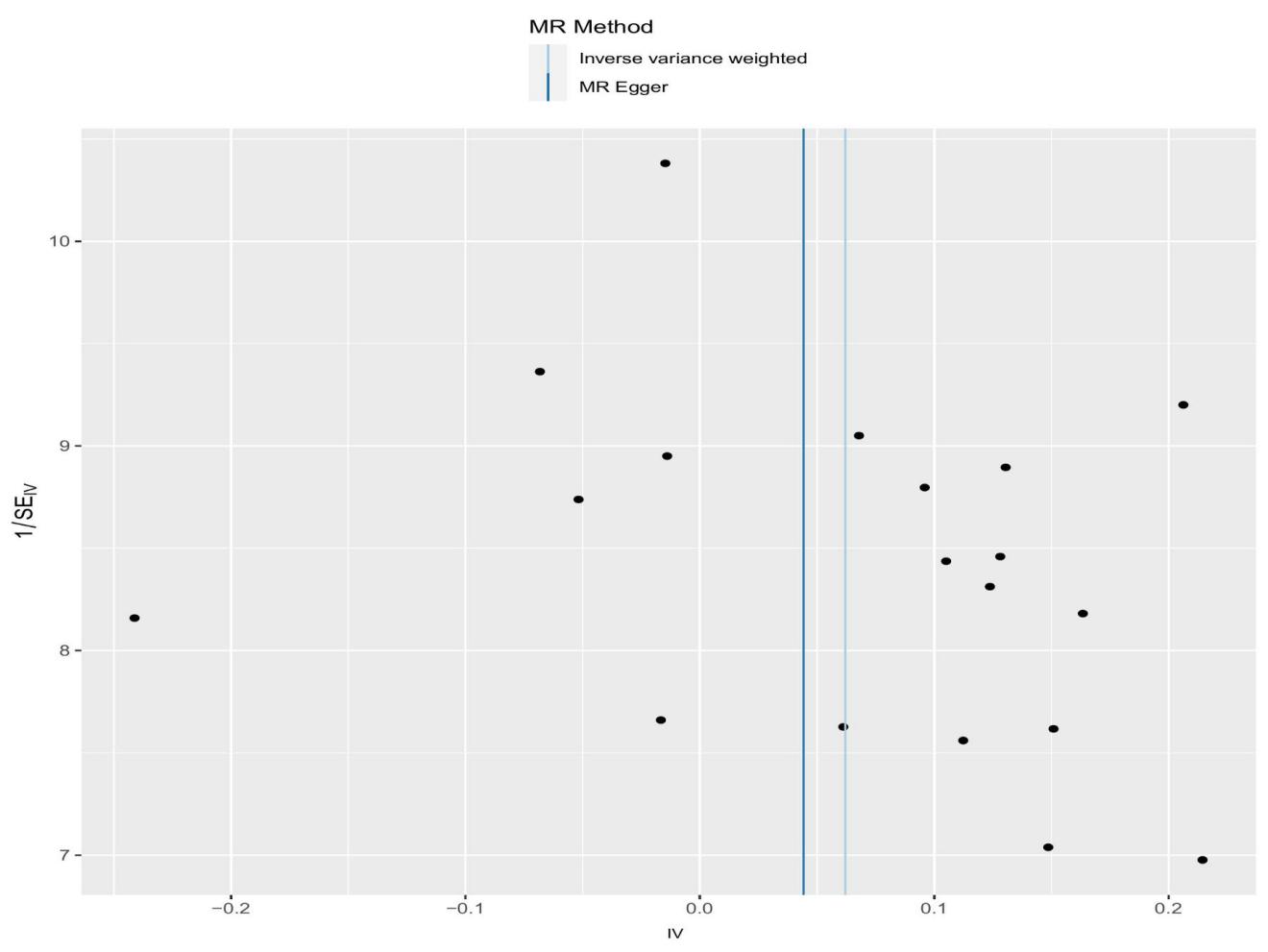


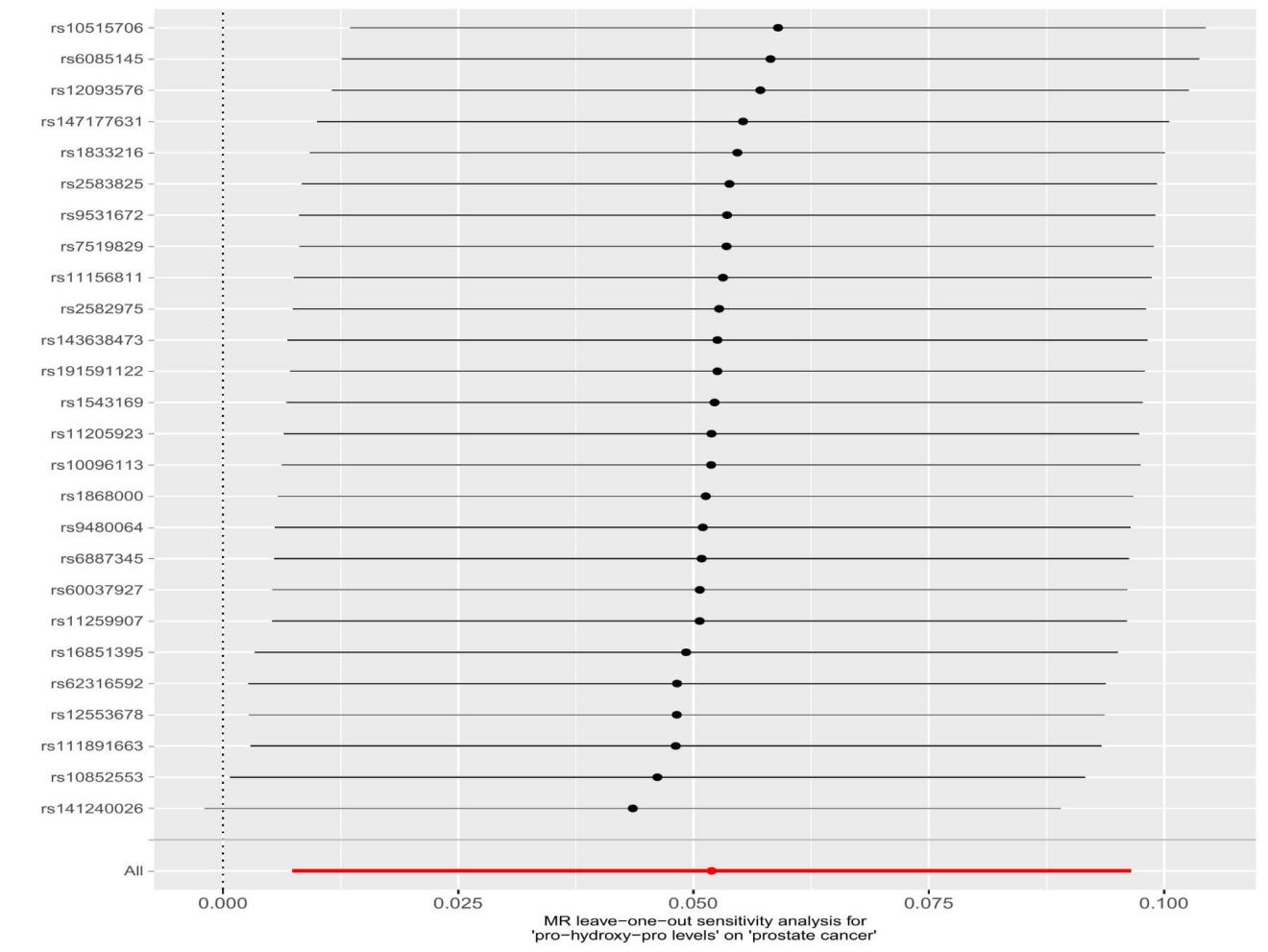


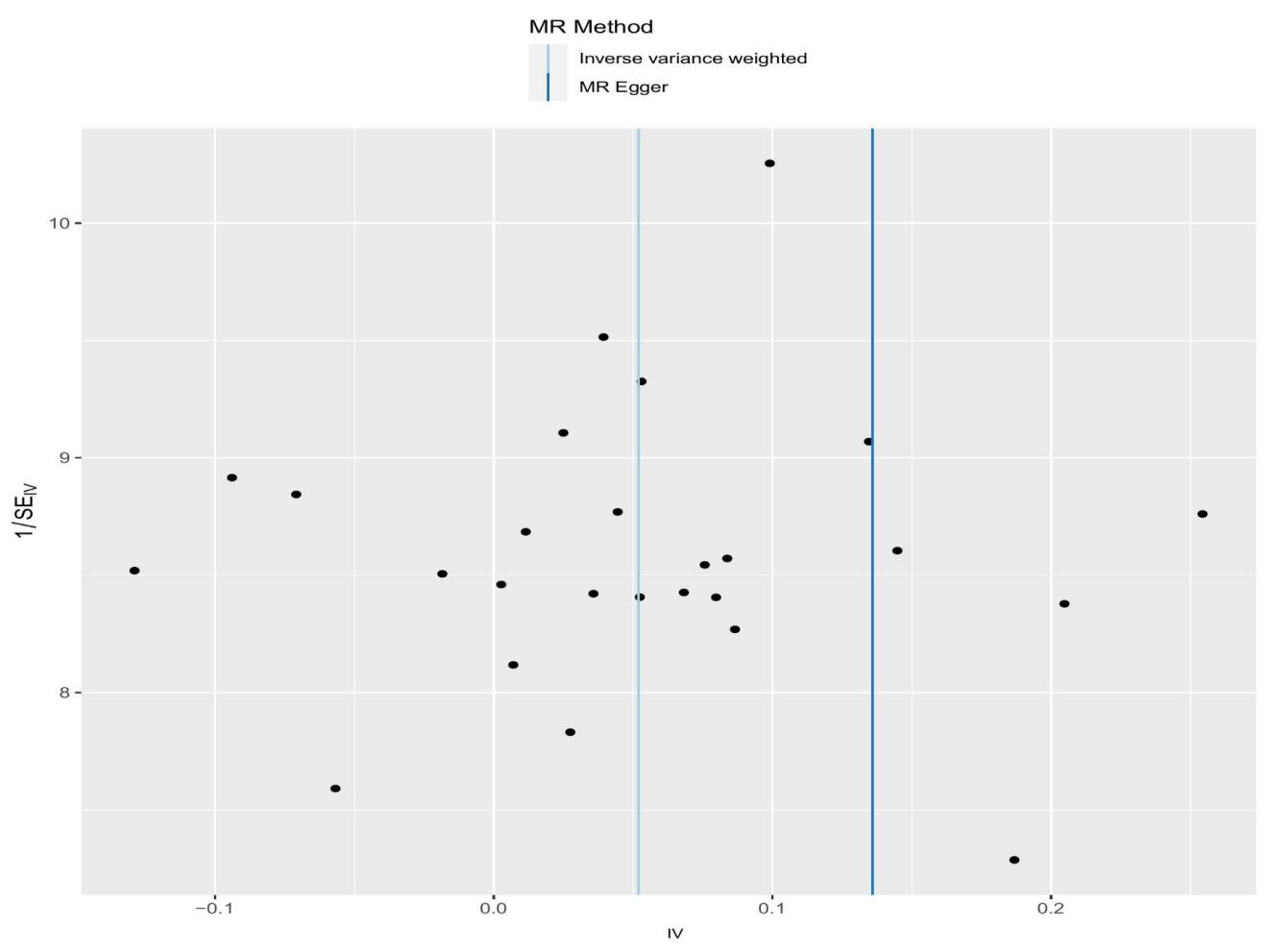


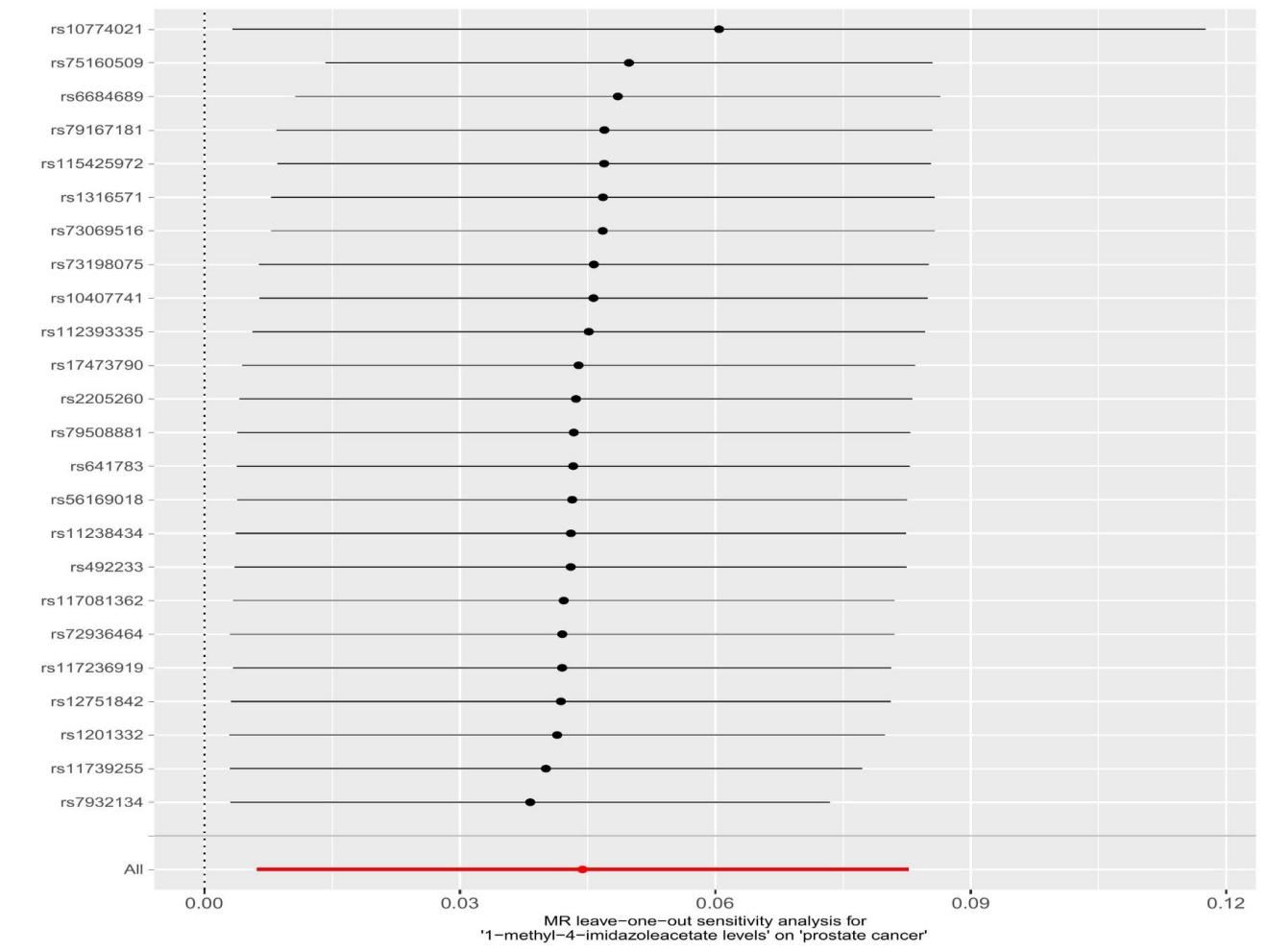


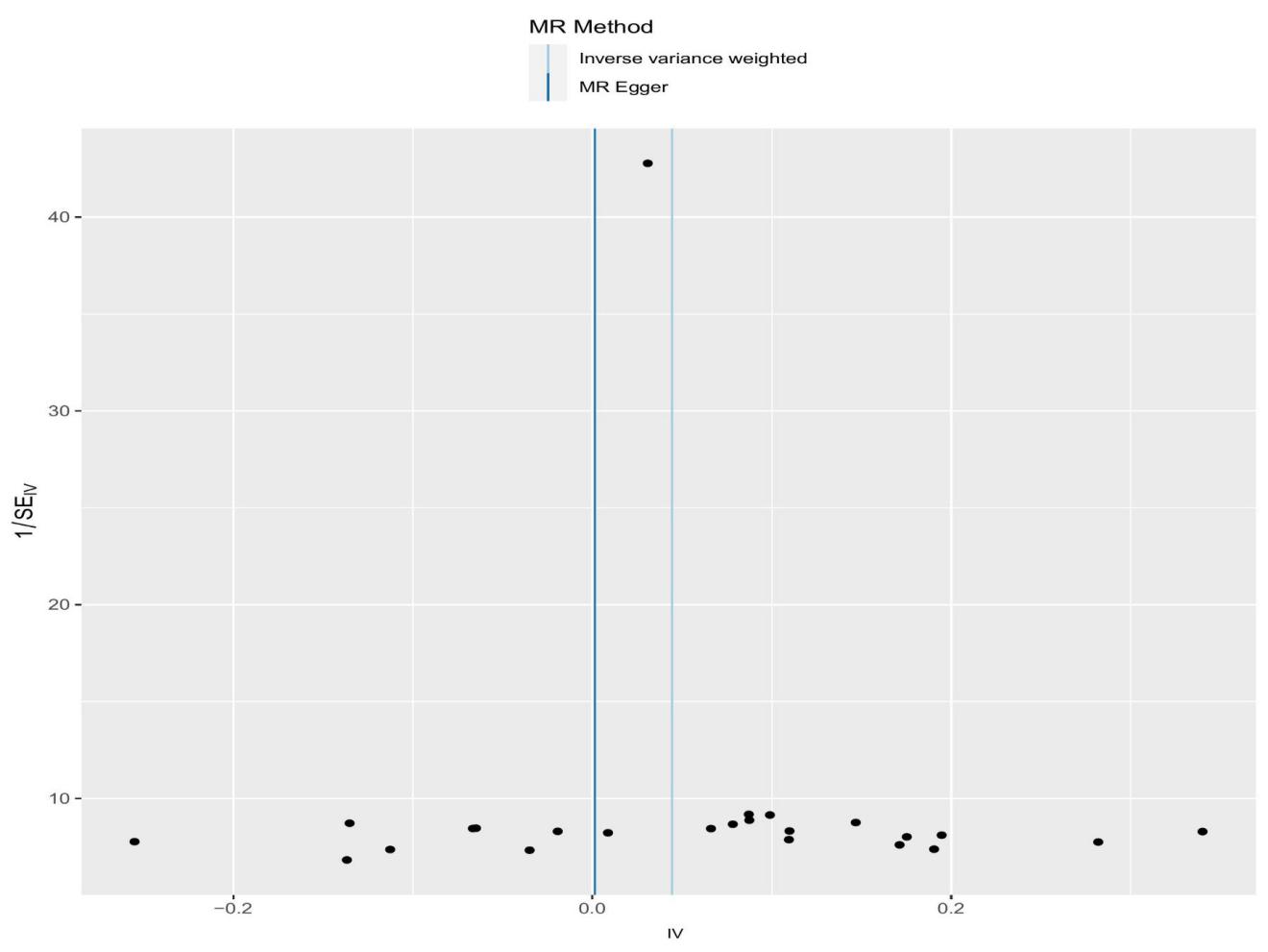


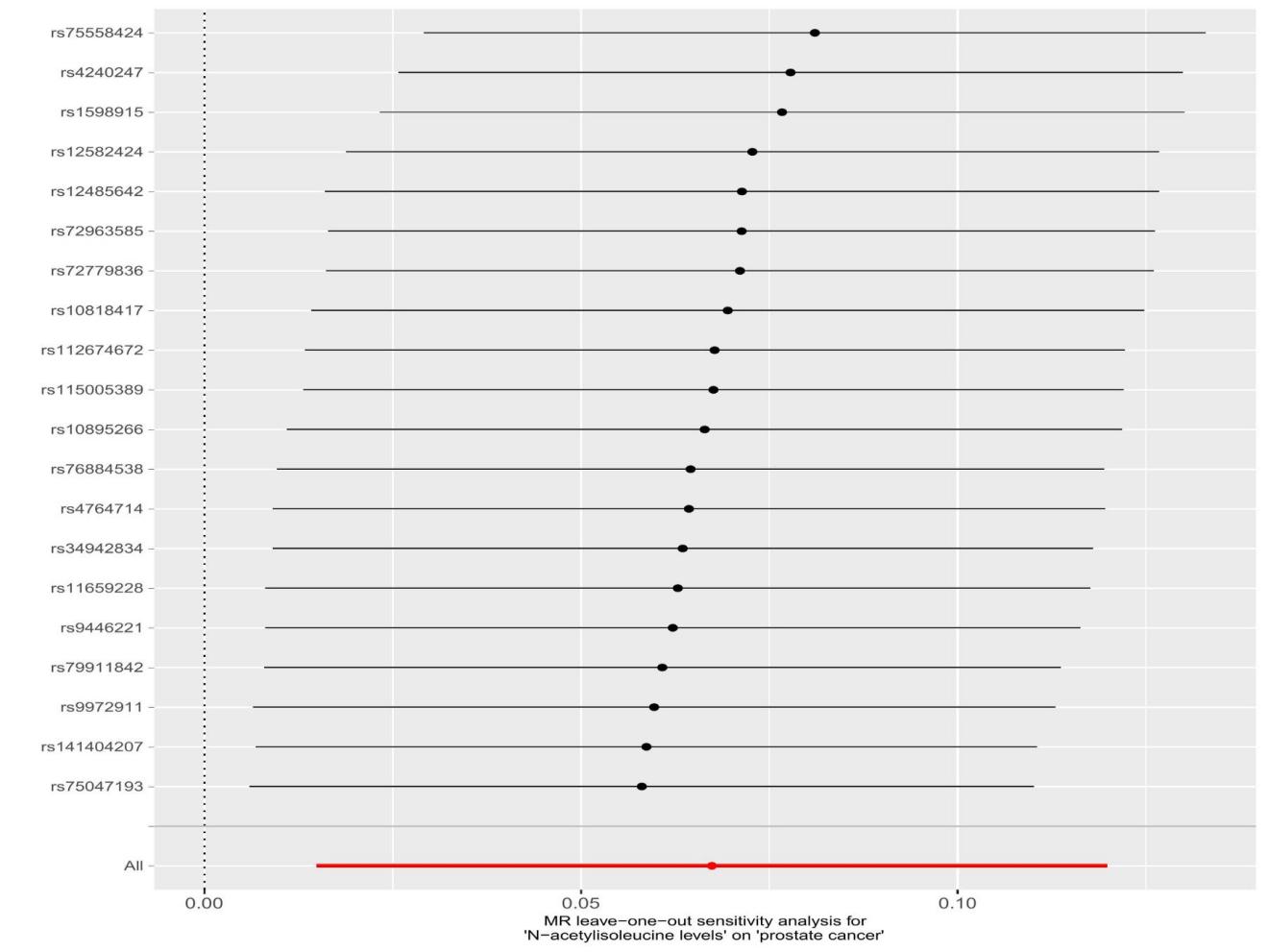


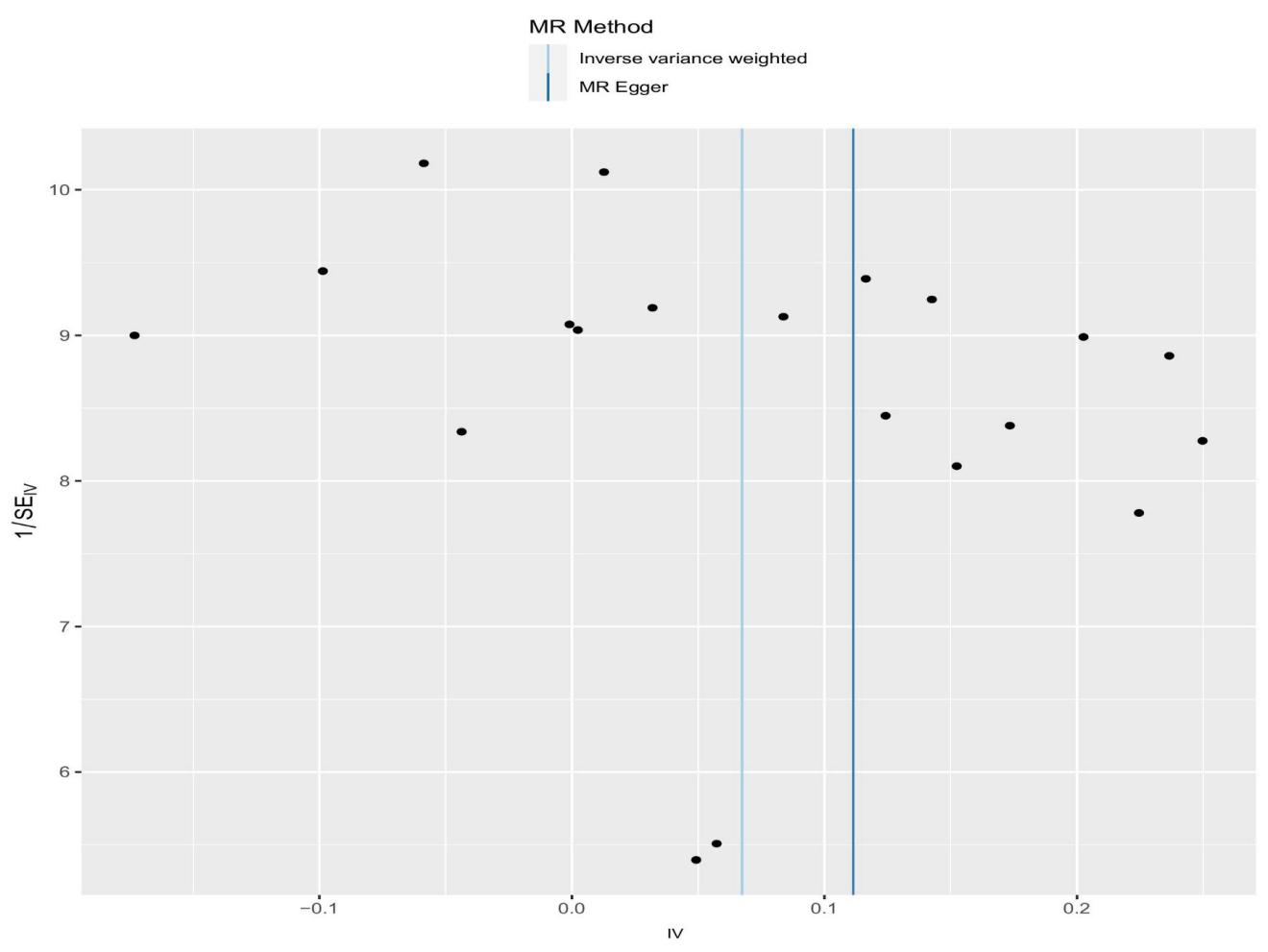


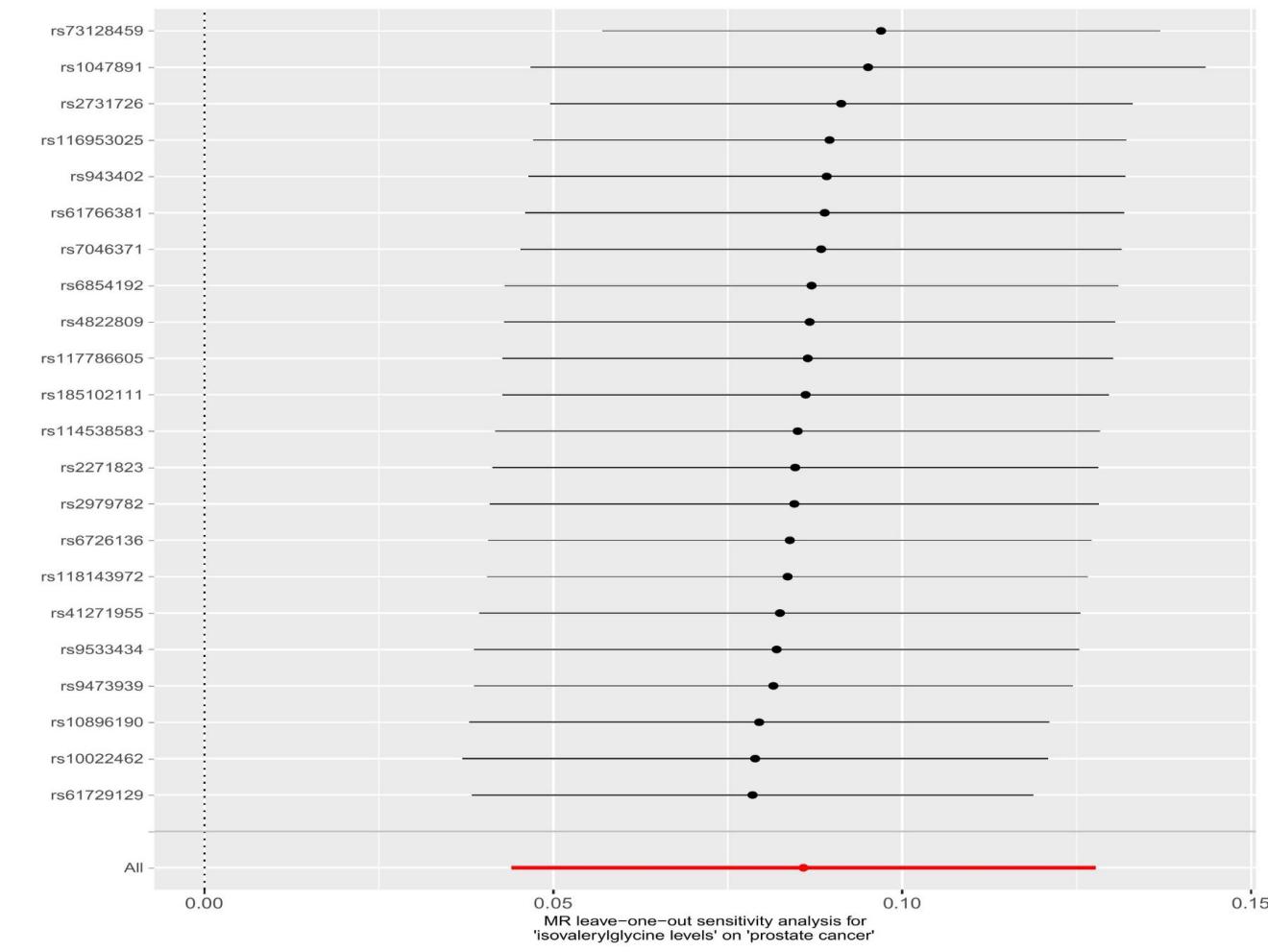


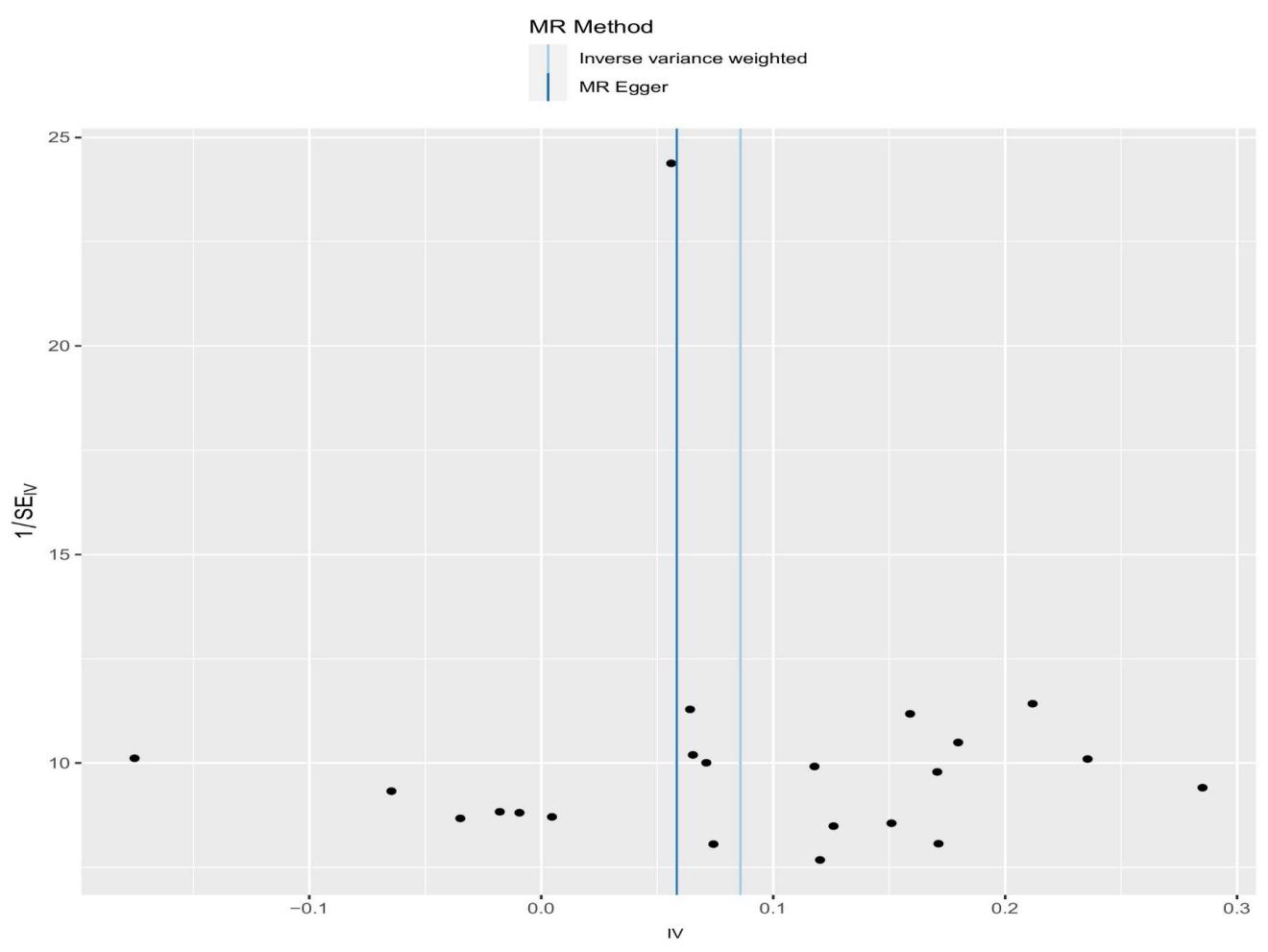


Testicular Carcinoma
