# Supplementary material for: Causality of genetically determined metabolites on susceptibility to prevalent urological cancers: a two-sample Mendelian randomization study and meta-analysis
Source: Front Genet. 2024 Jul 1;15:1398165. doi: 10.3389/fgene.2024.1398165 (PMC11246892; doi:10.3389/fgene.2024.1398165)
Supplement: Supplementary file 4 [file Table4.DOCX]

**Additional file 4: Leave-one-out analyses and funnel plots of potential causality in secondary MR analysis based on FinnGen outcome.**

Bladder Cancer


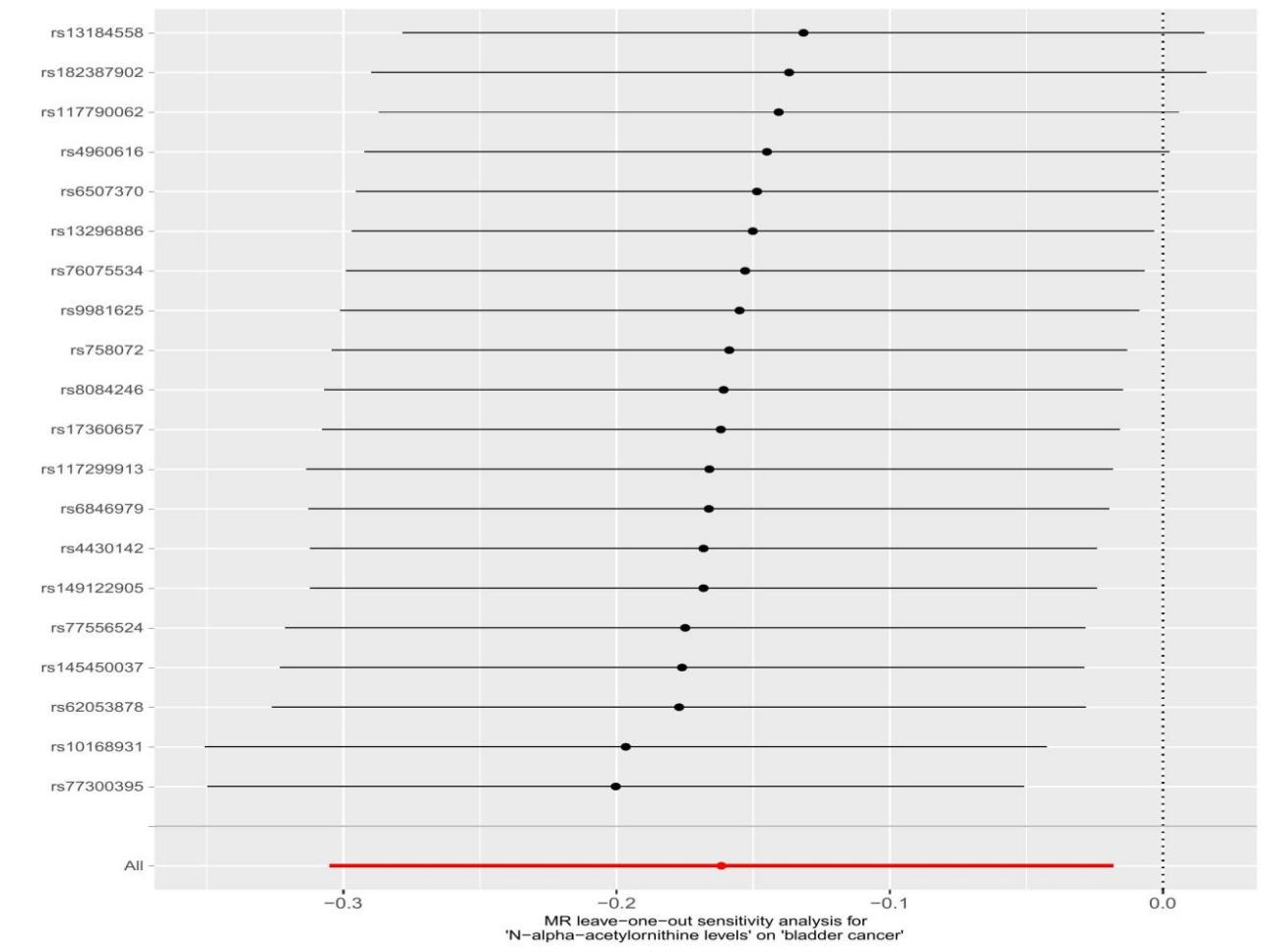


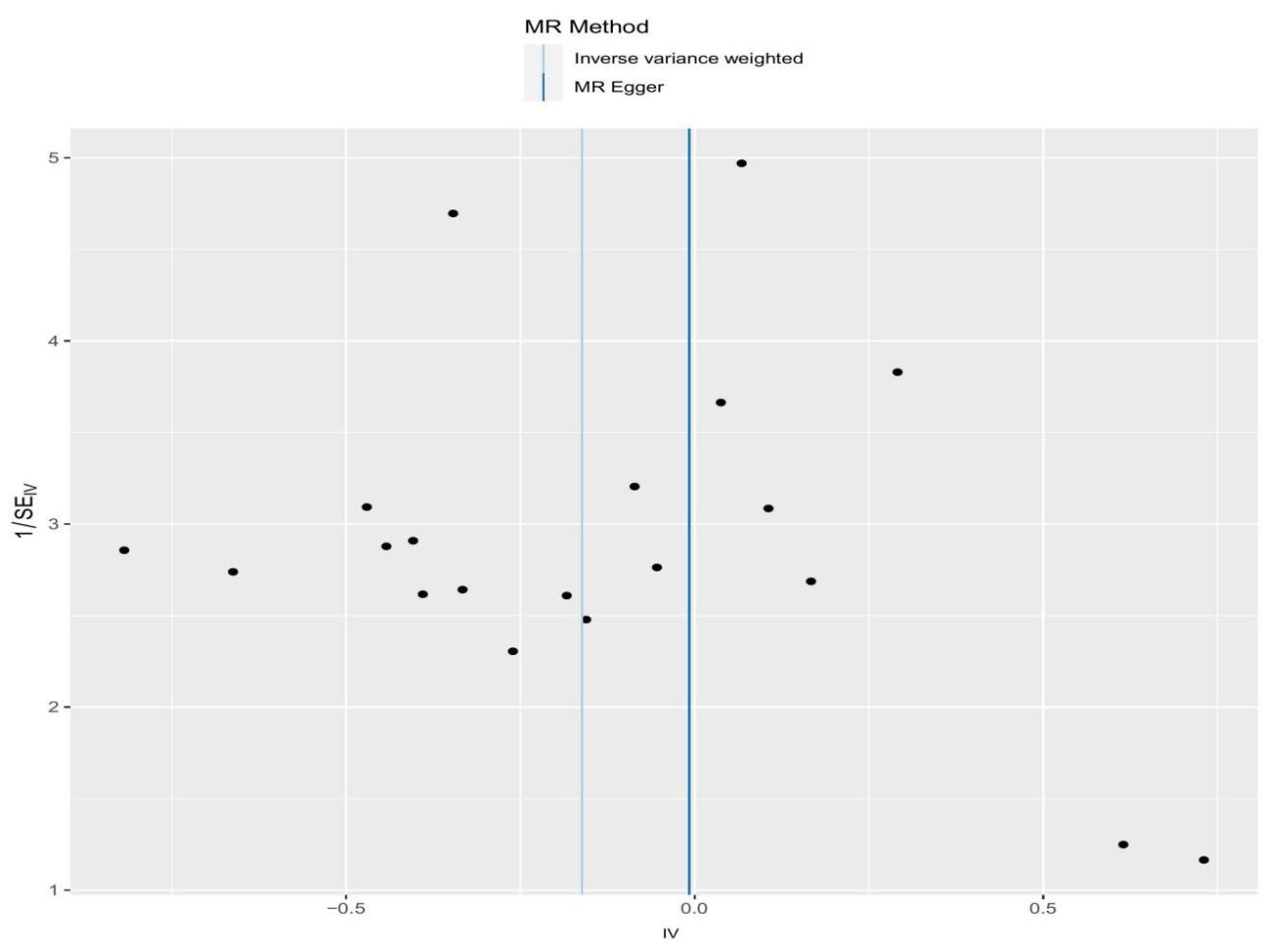


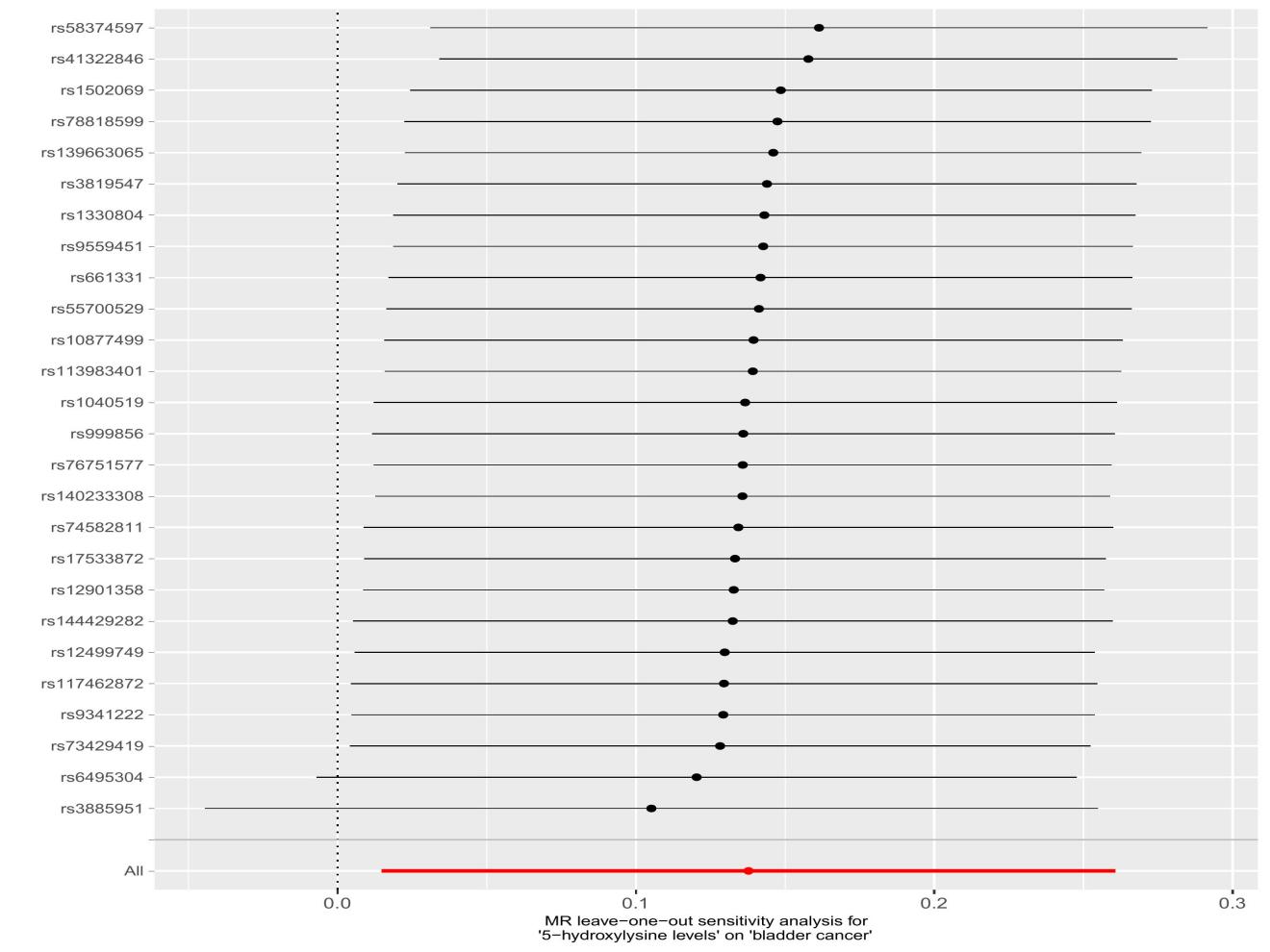


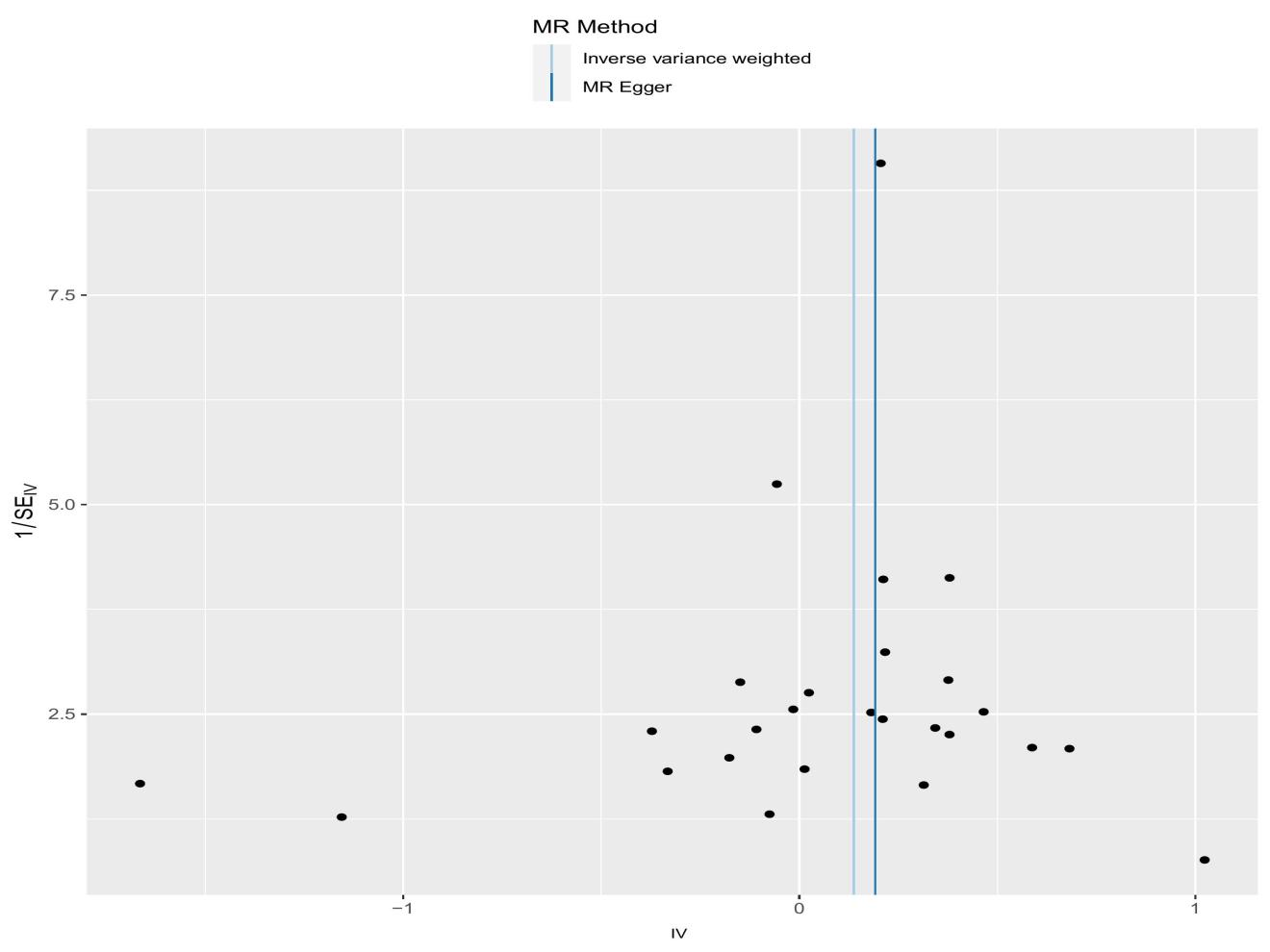


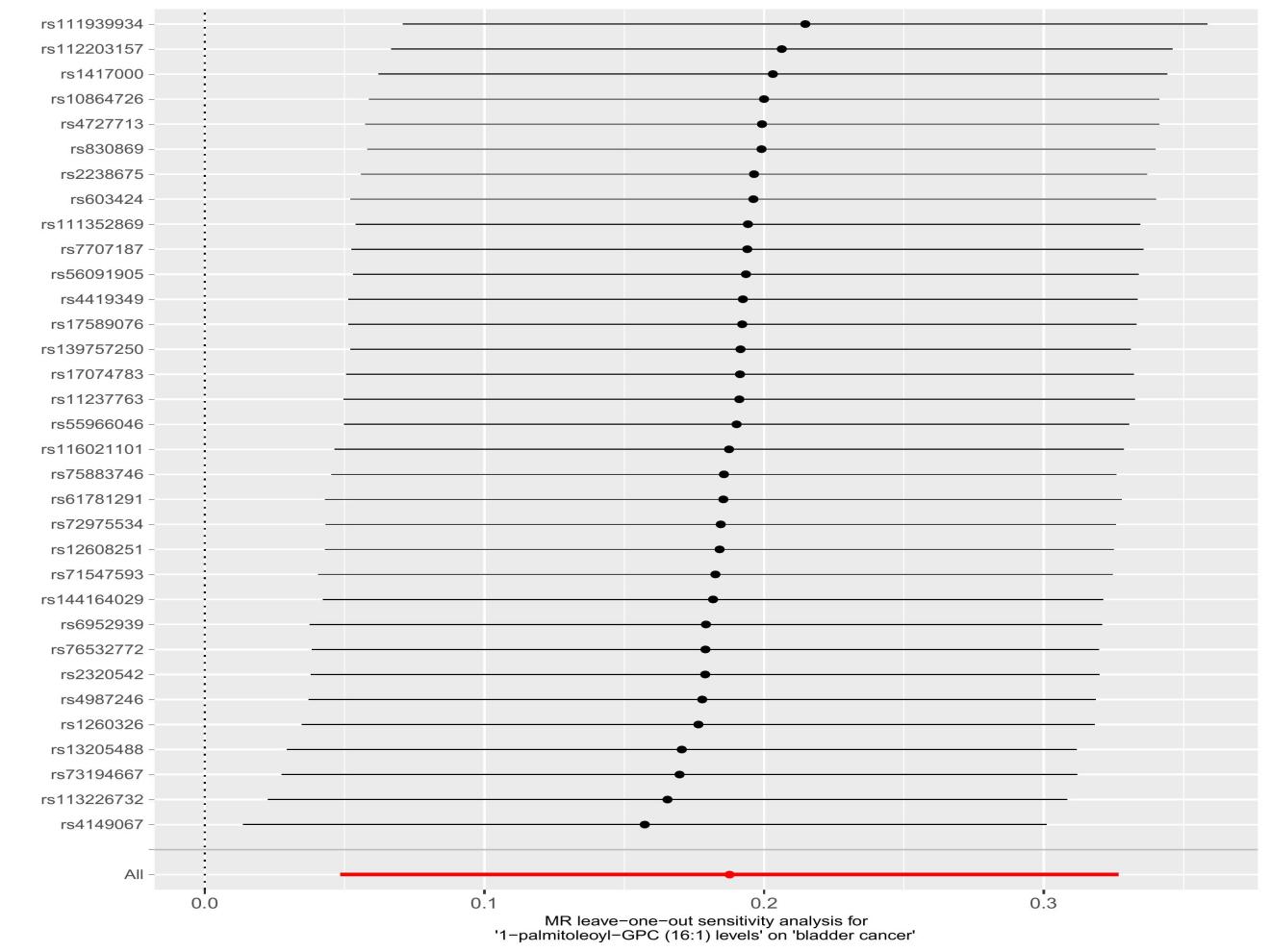


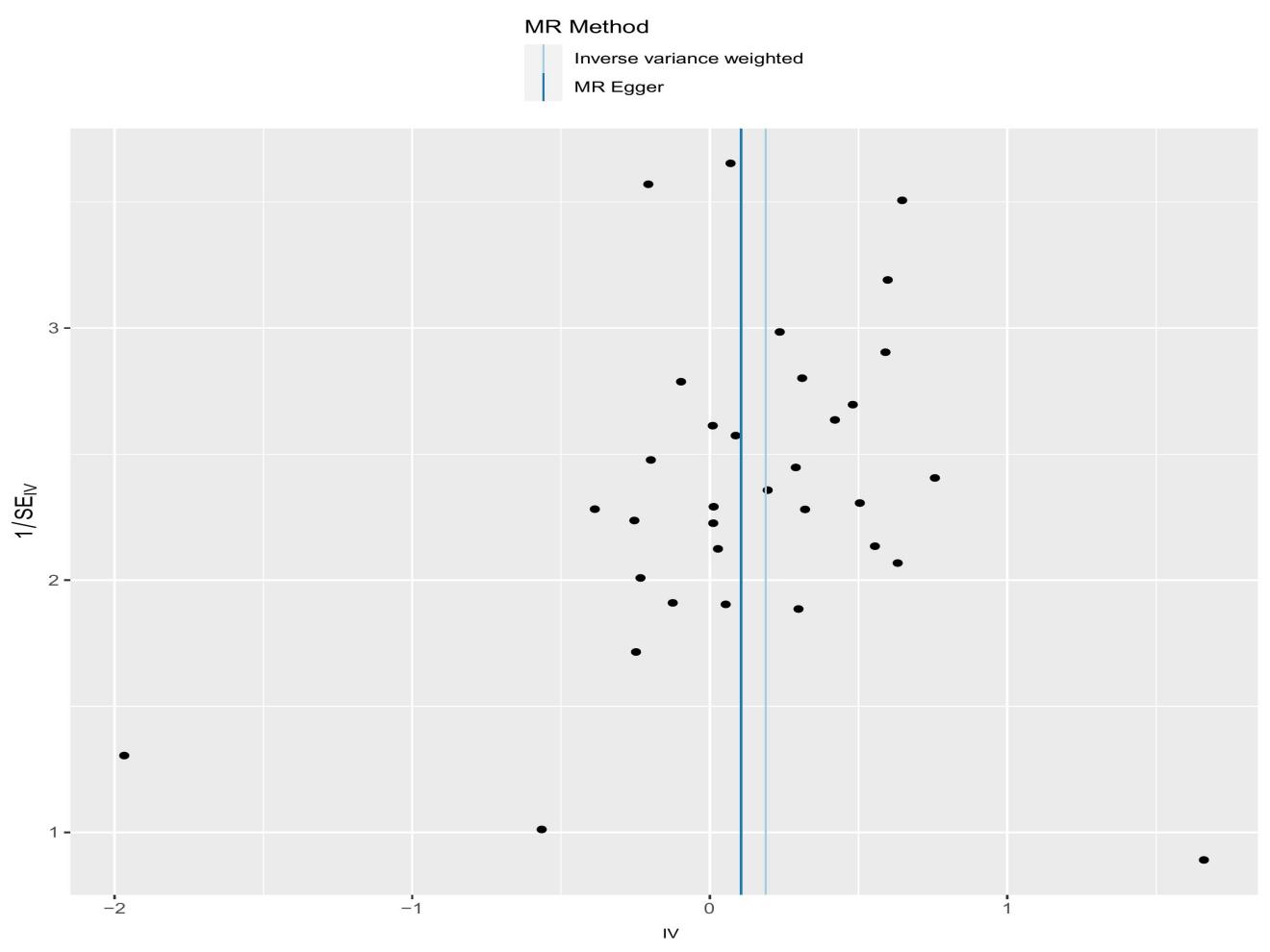


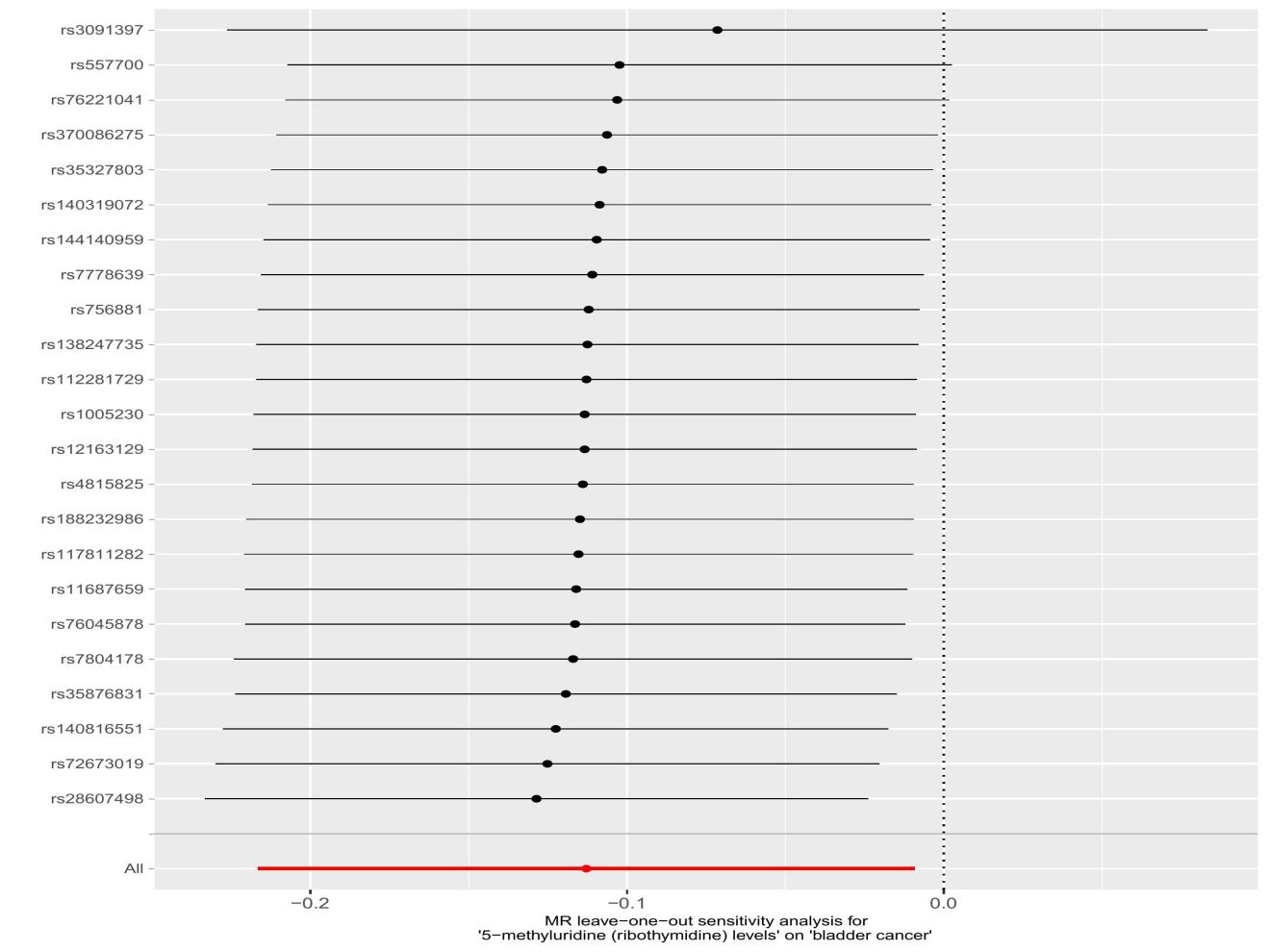


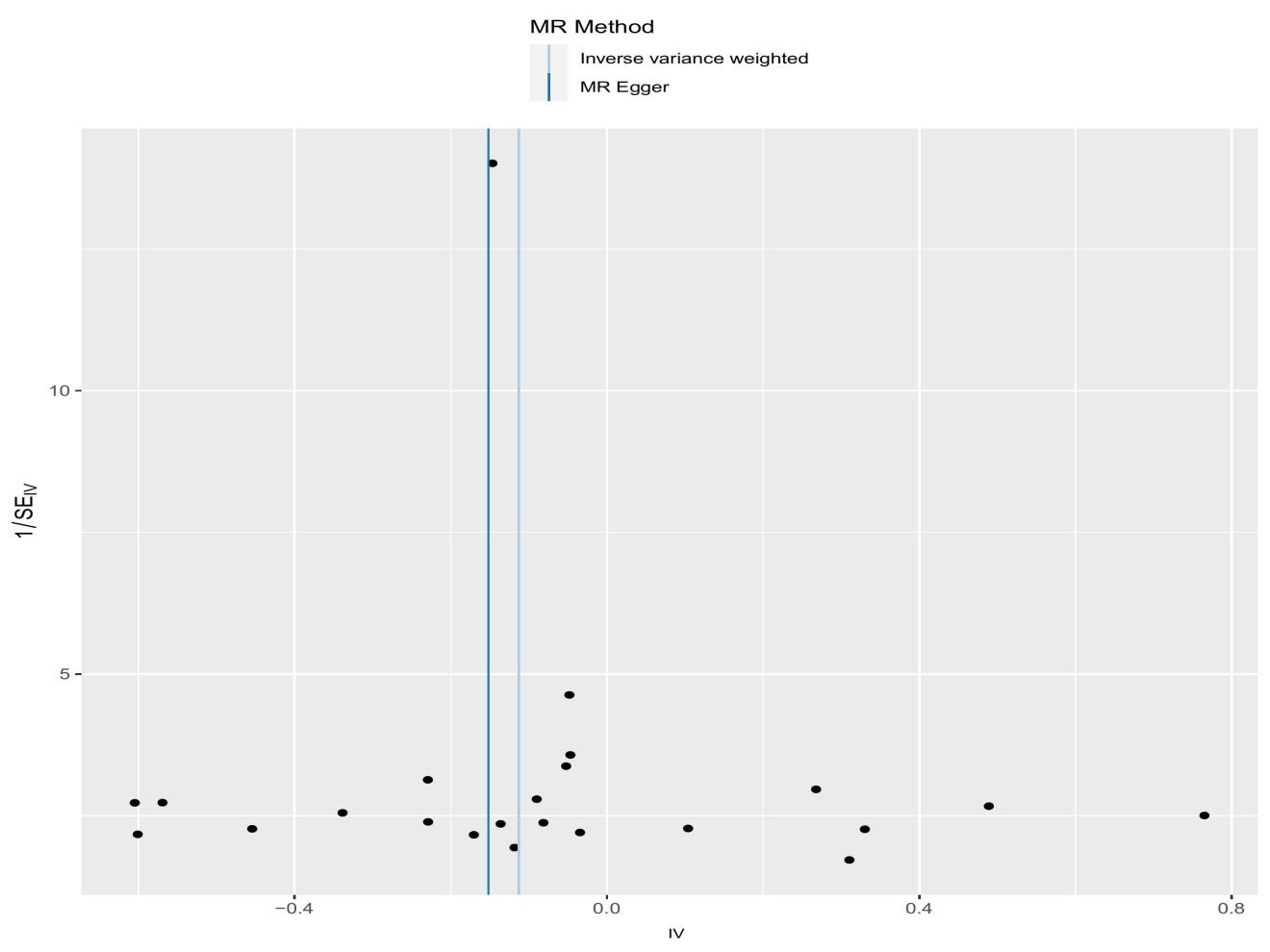


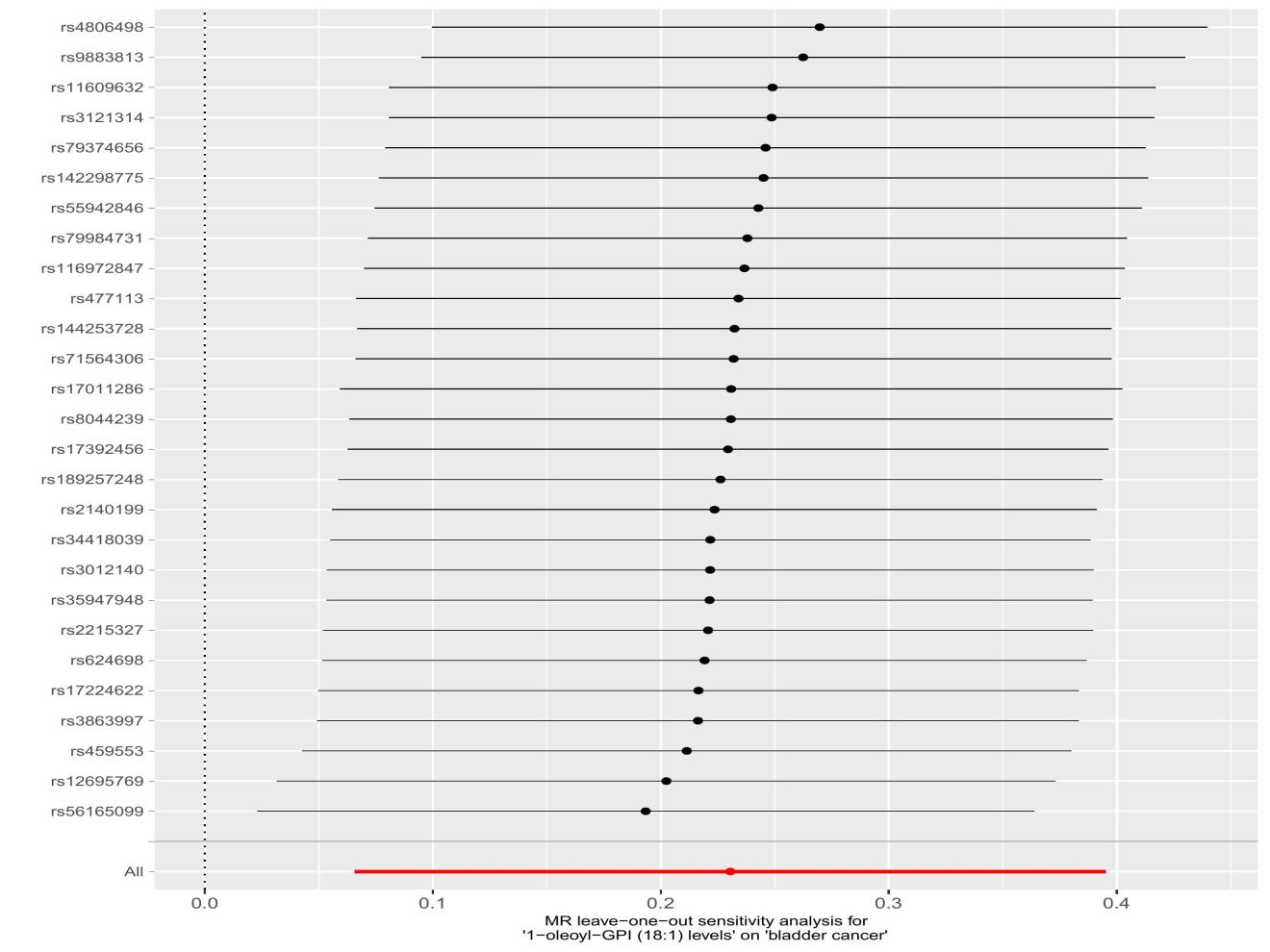


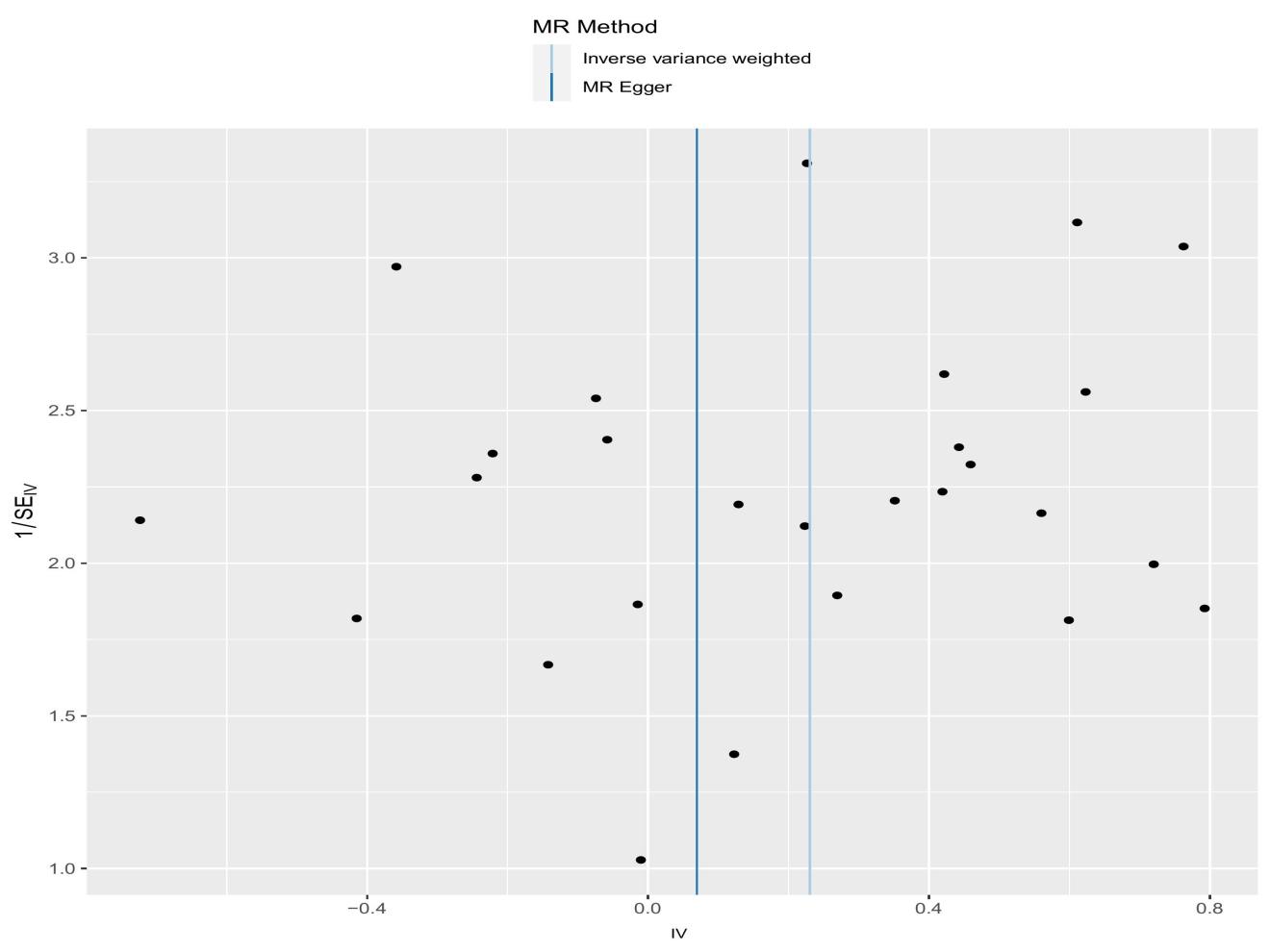


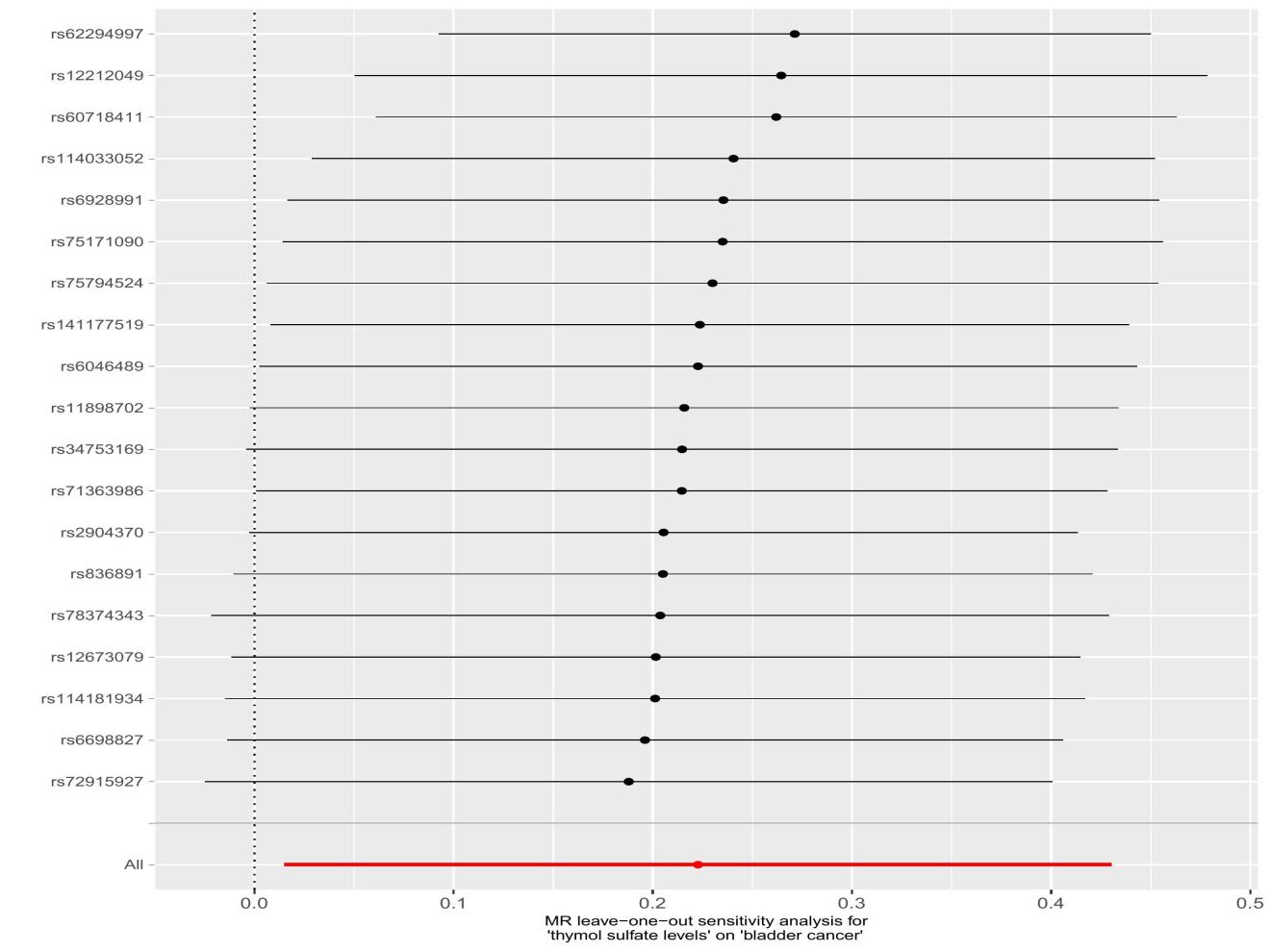


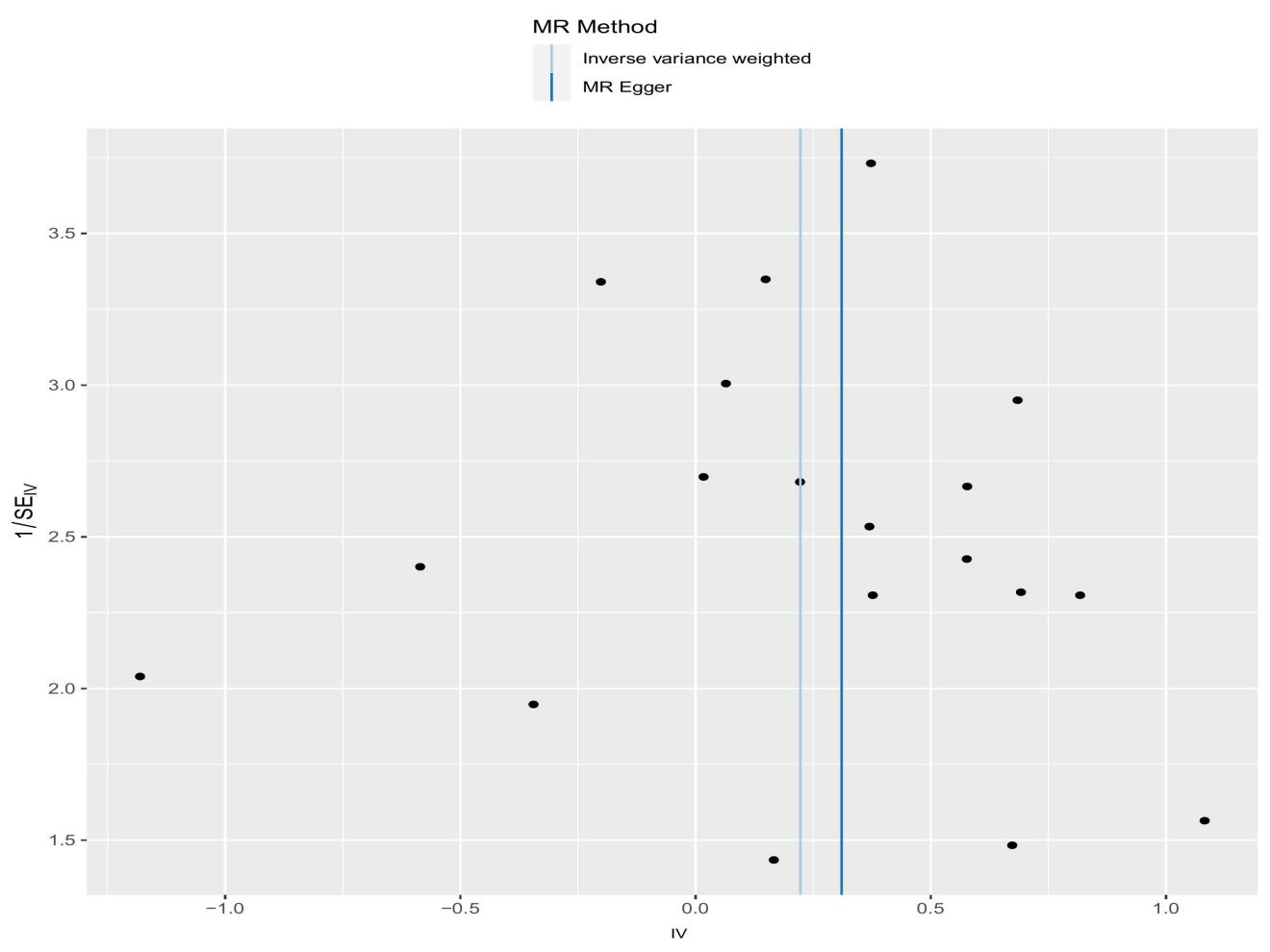


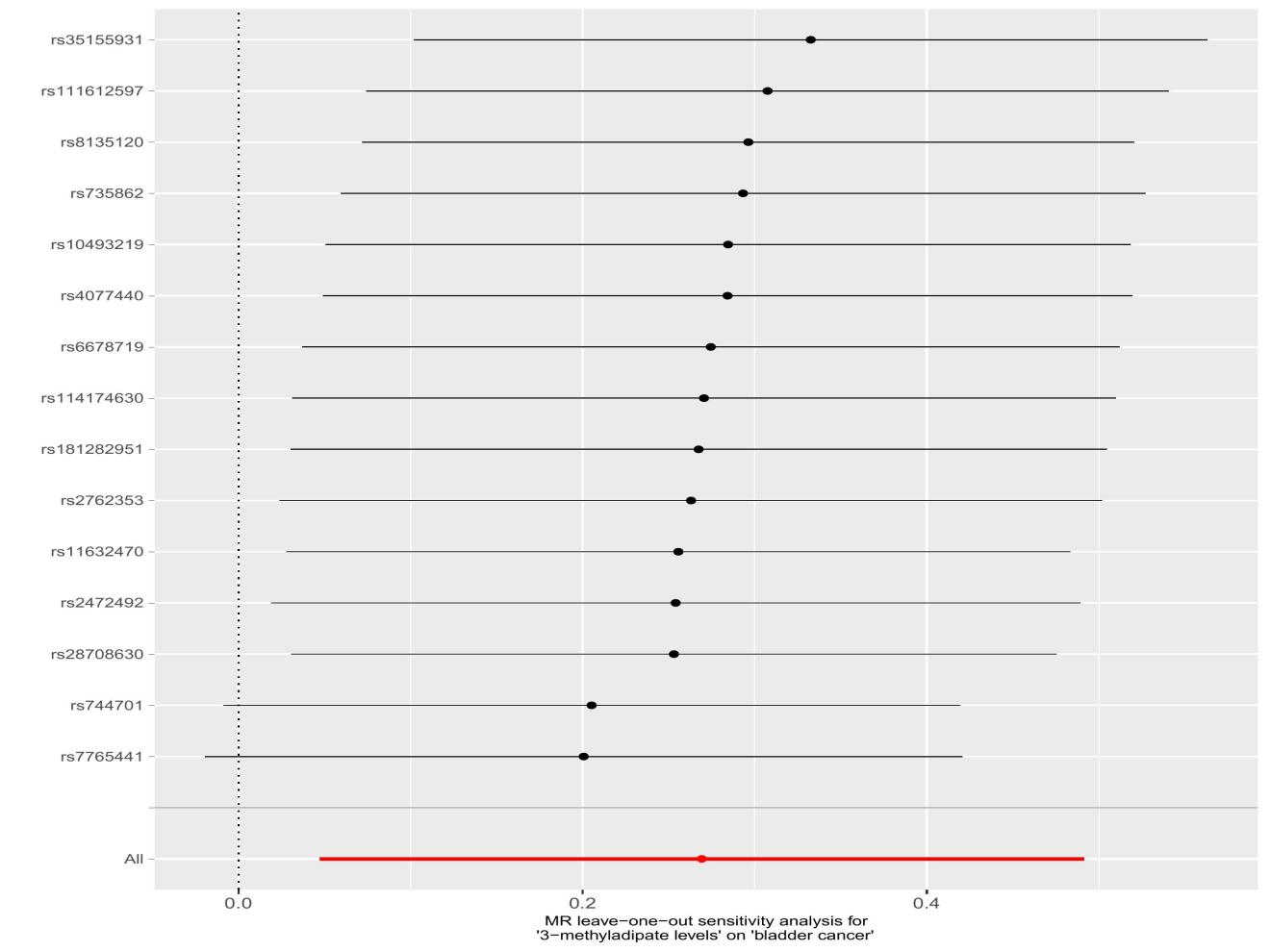


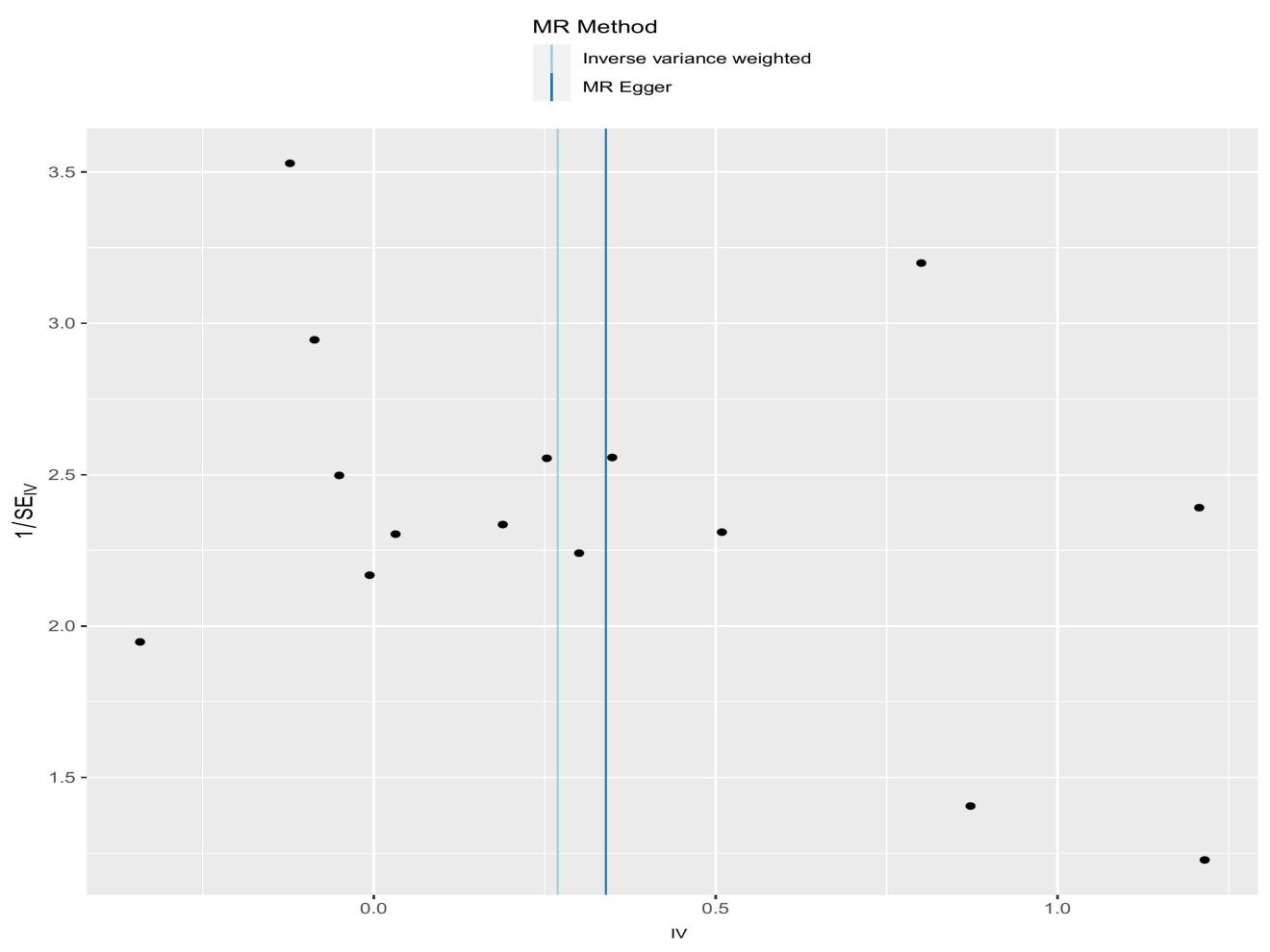


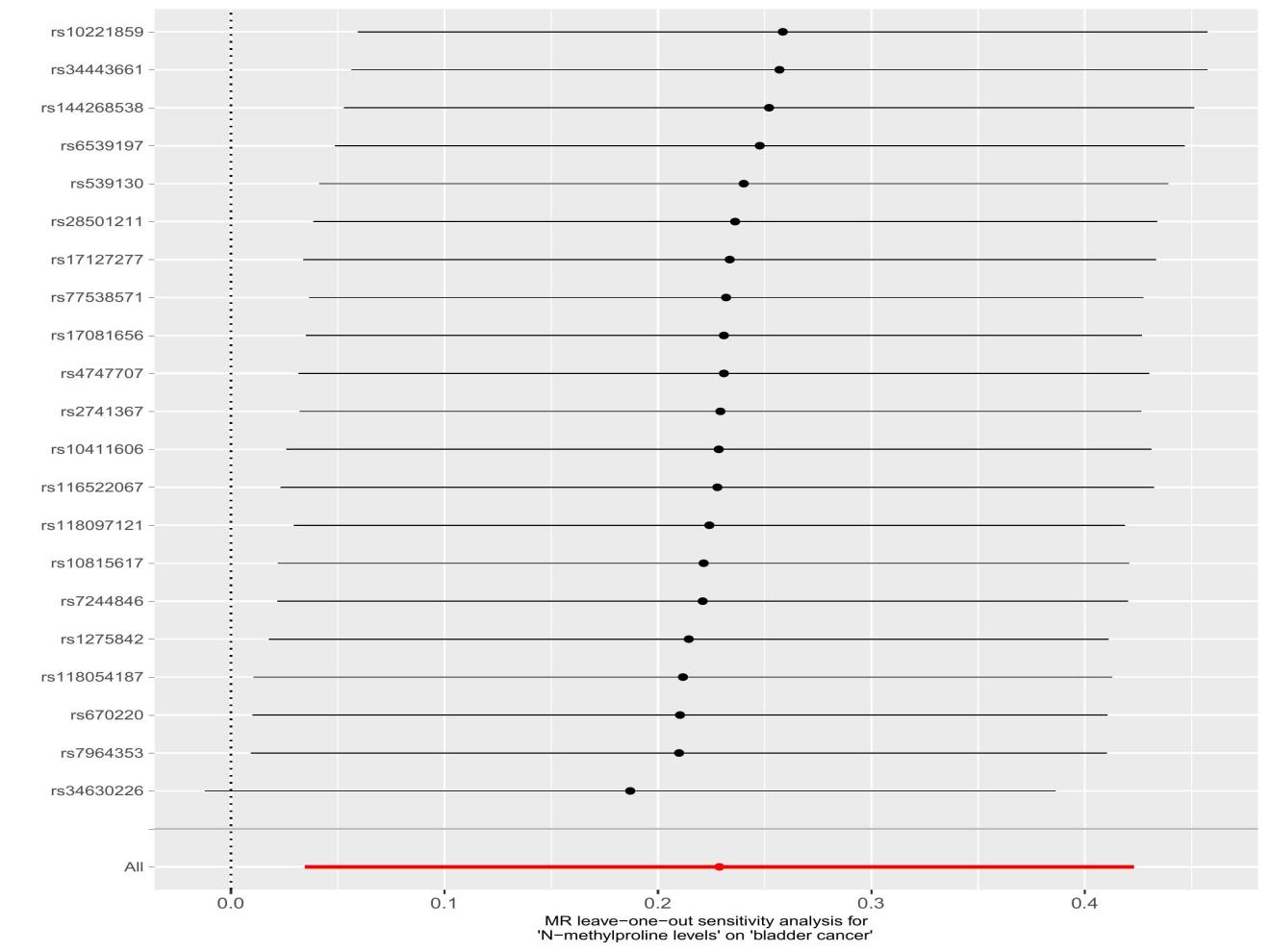


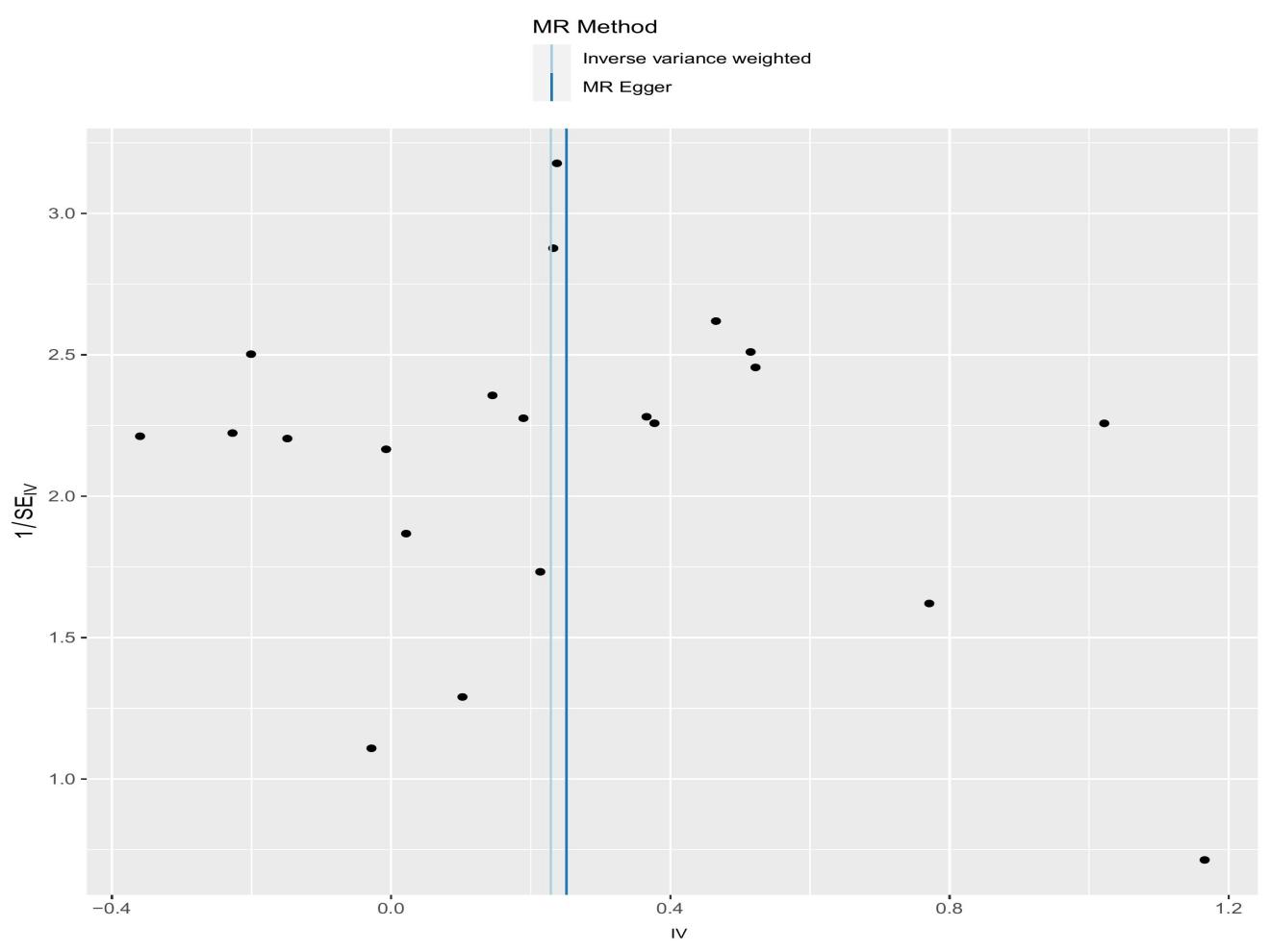


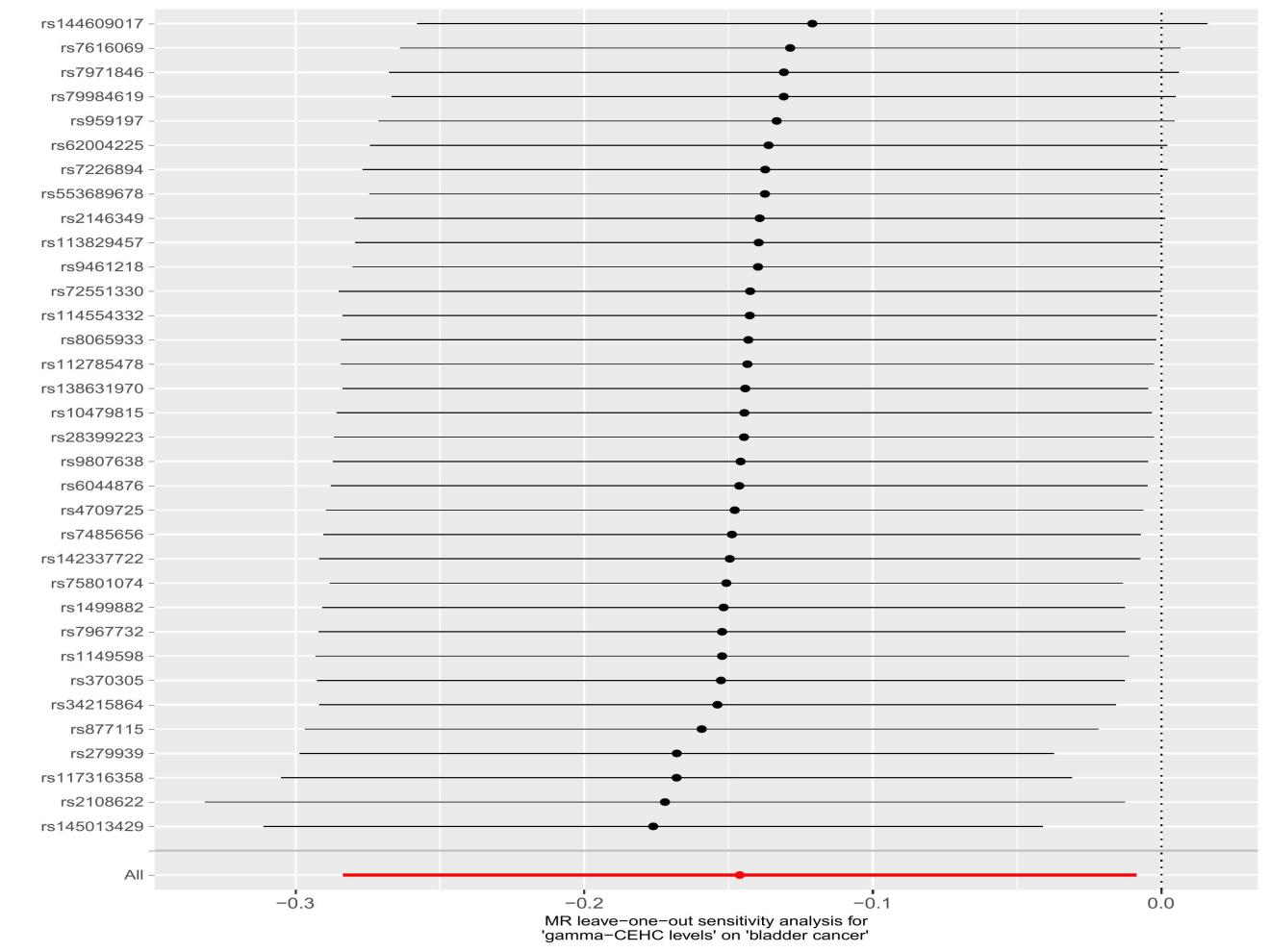


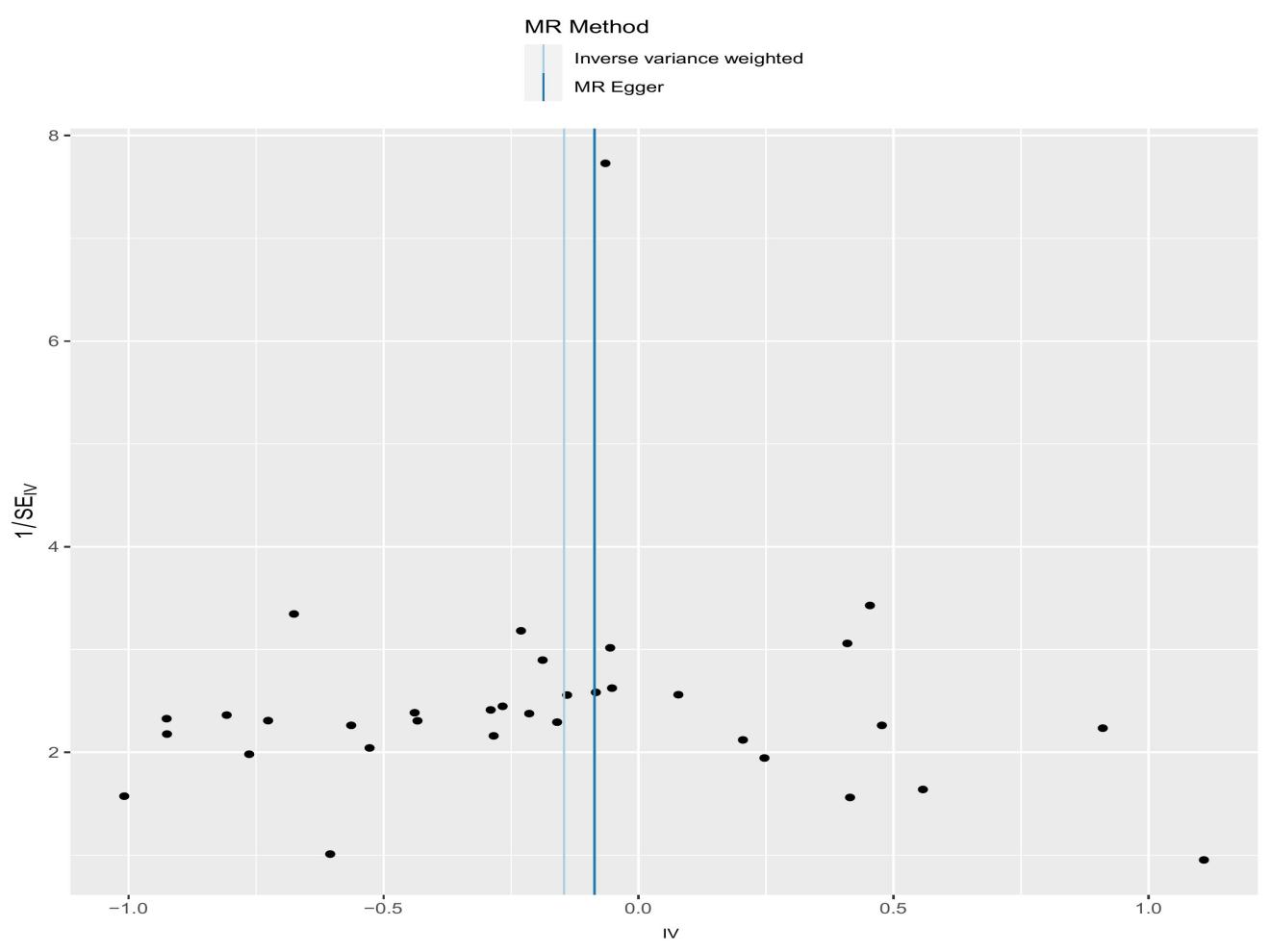


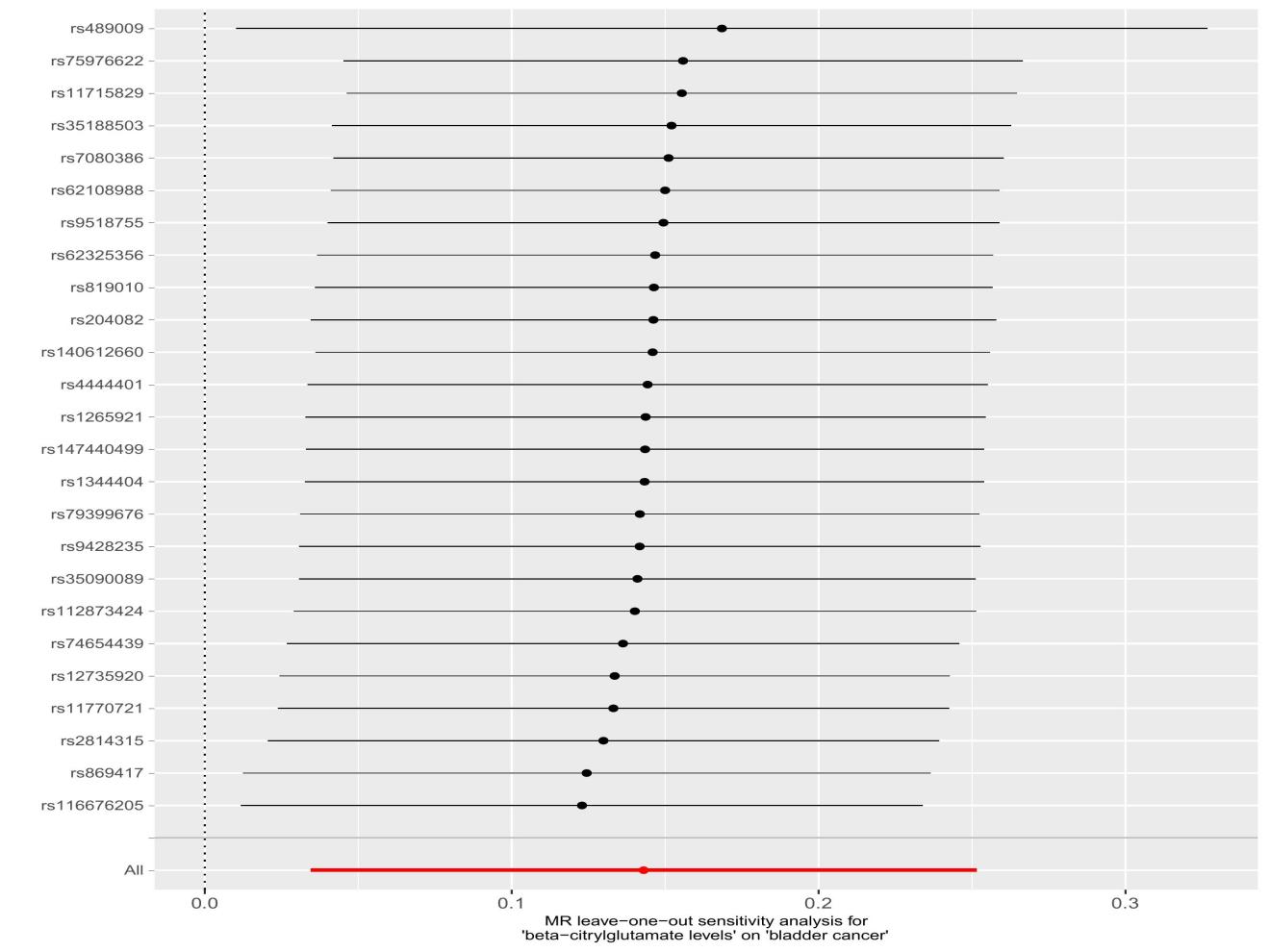


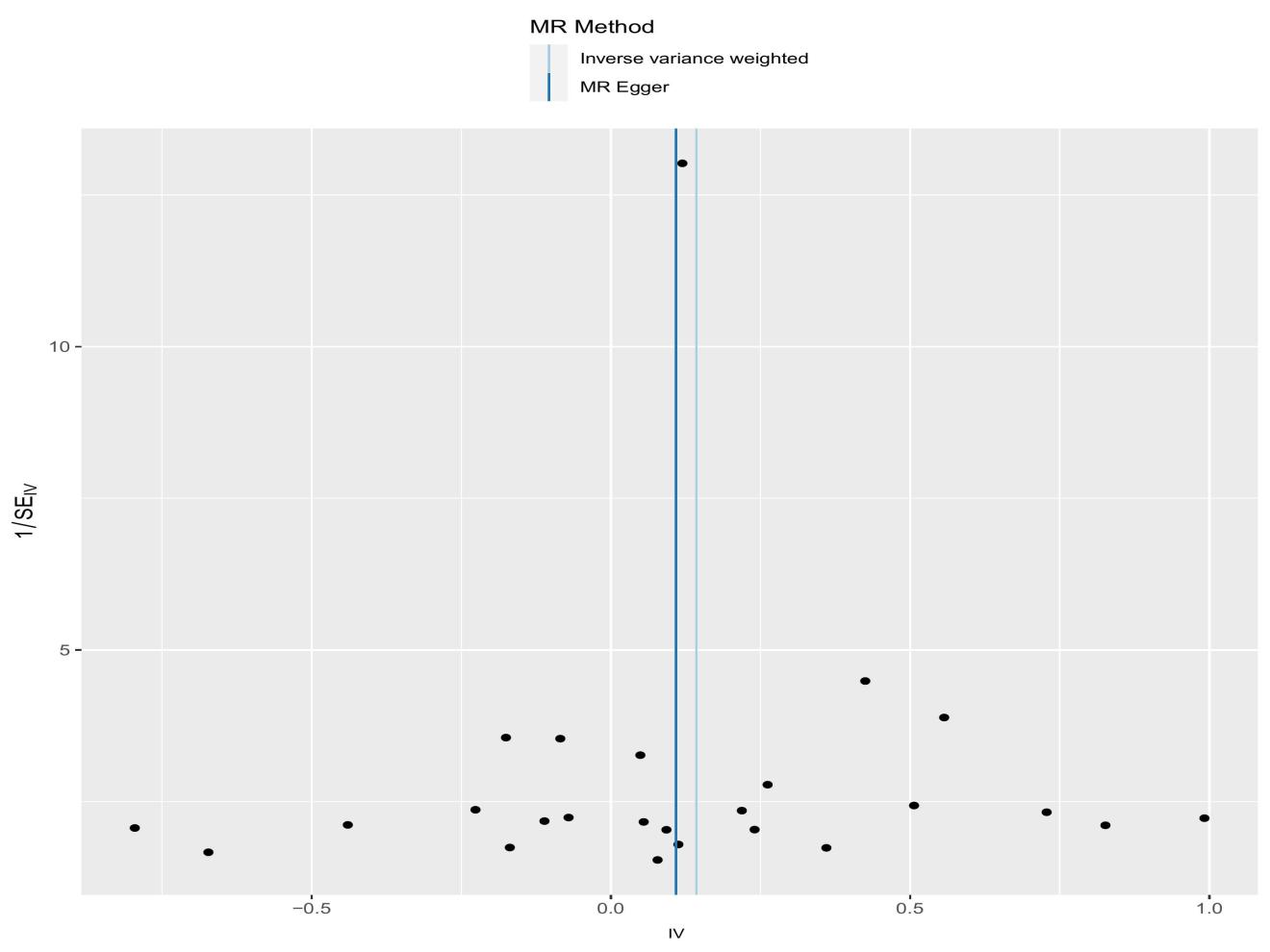


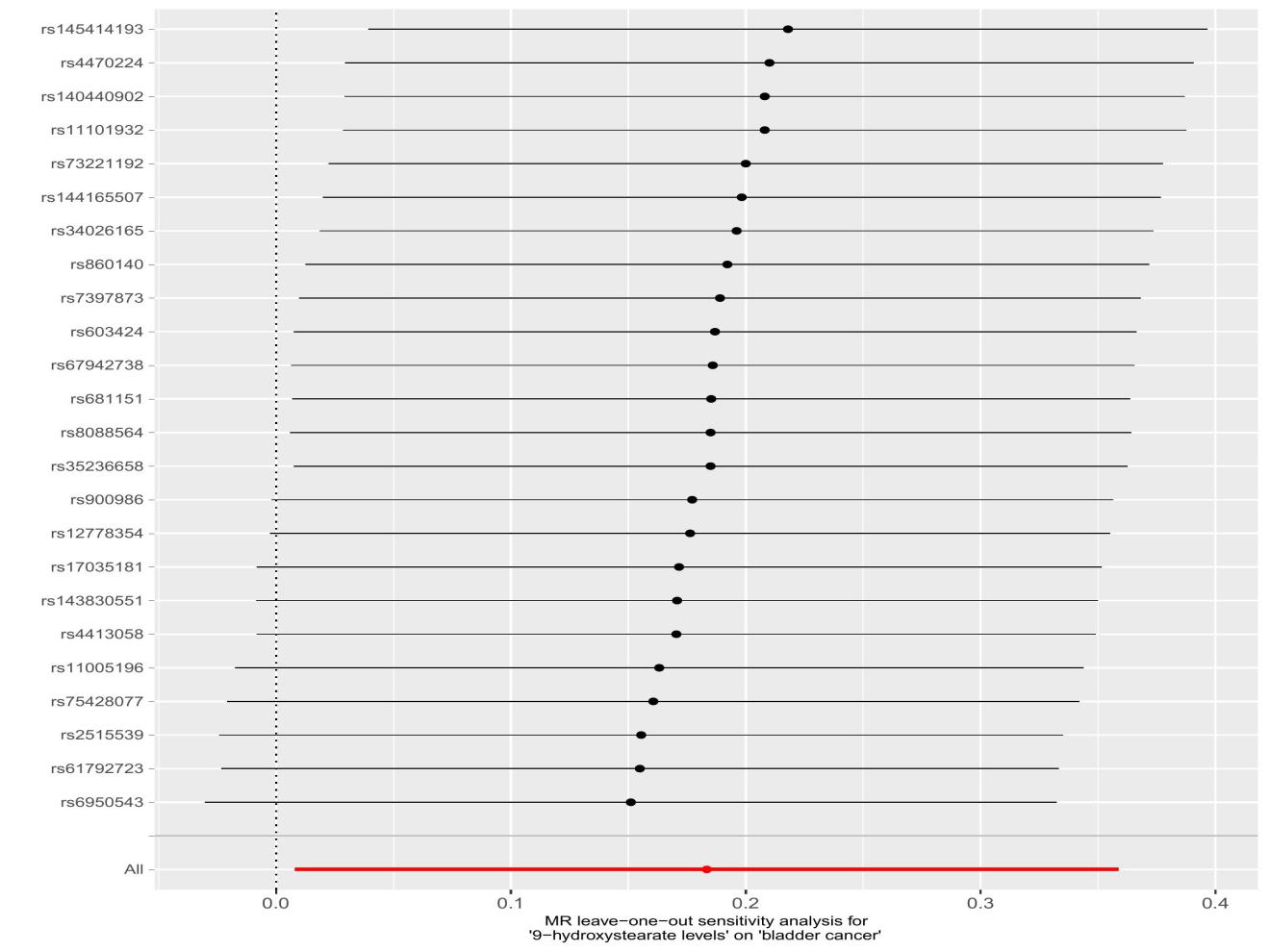


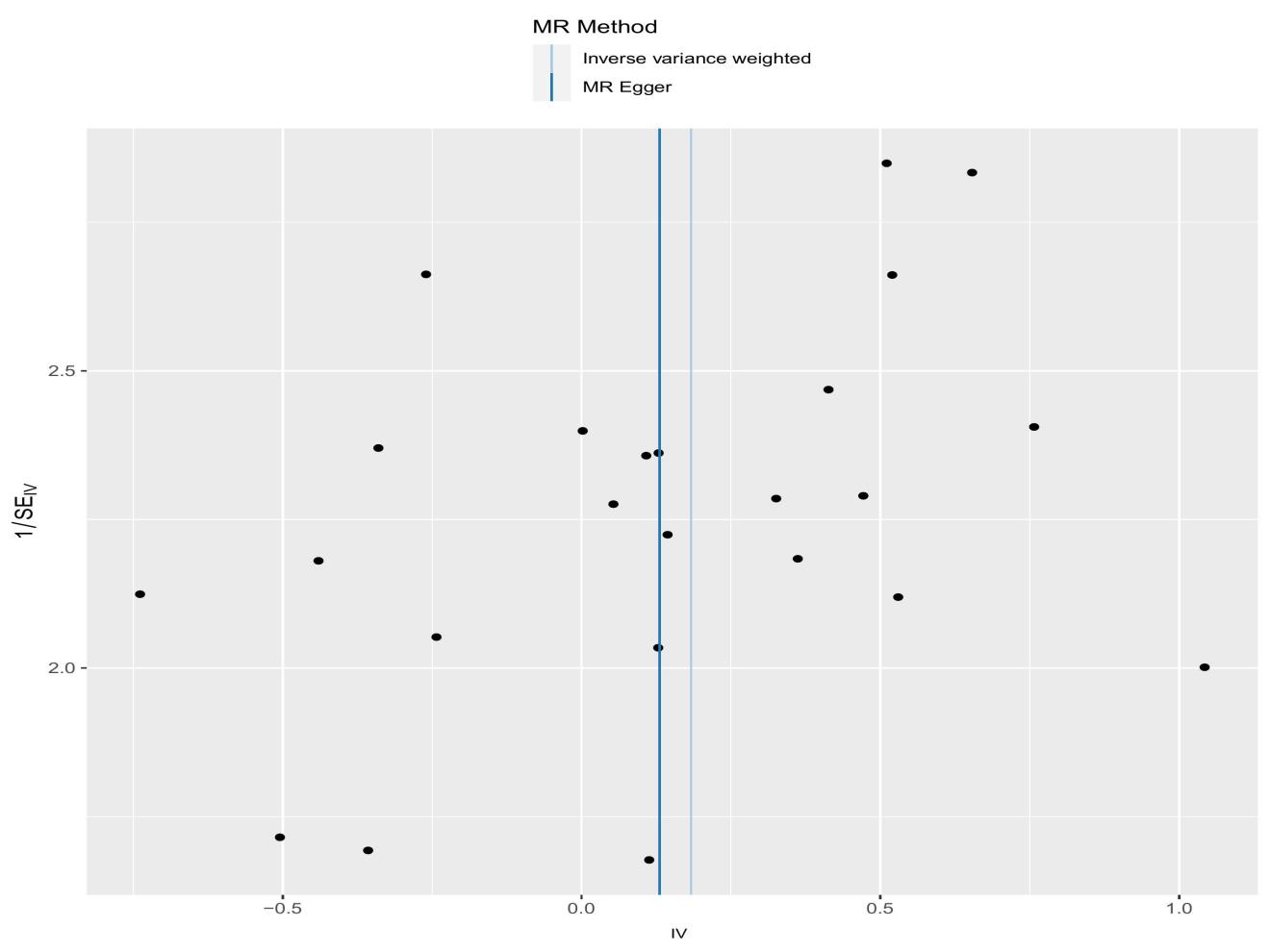


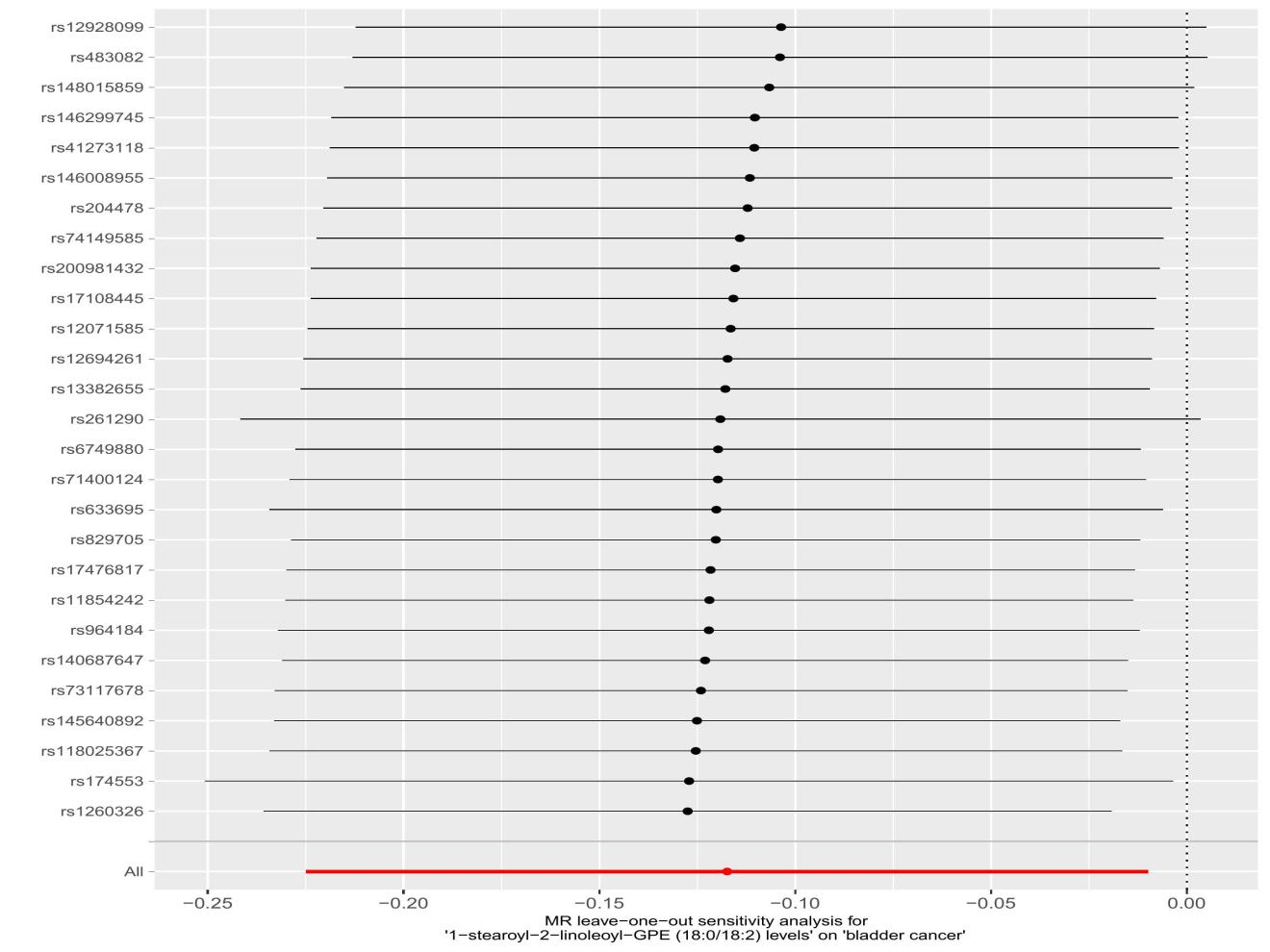


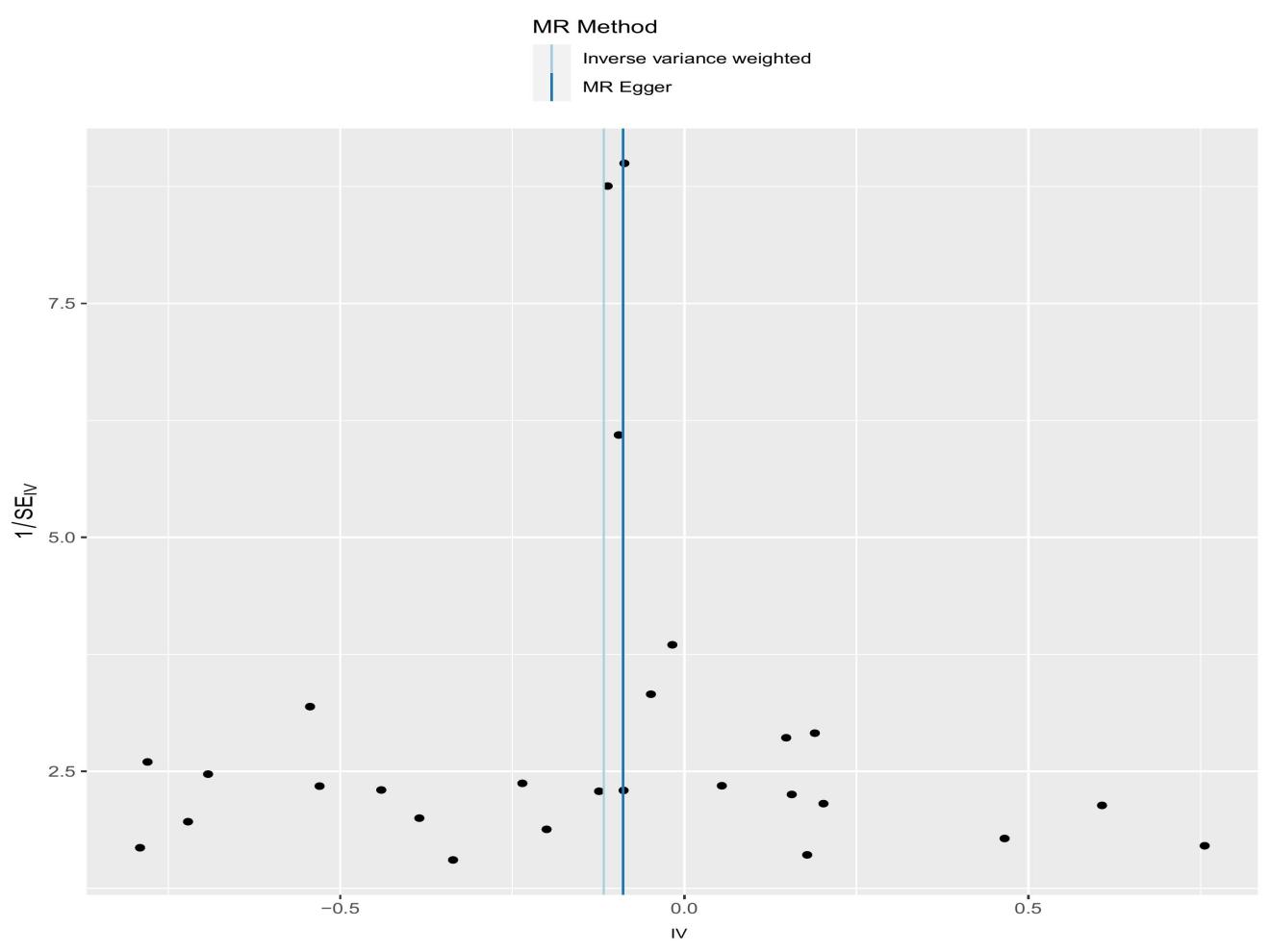


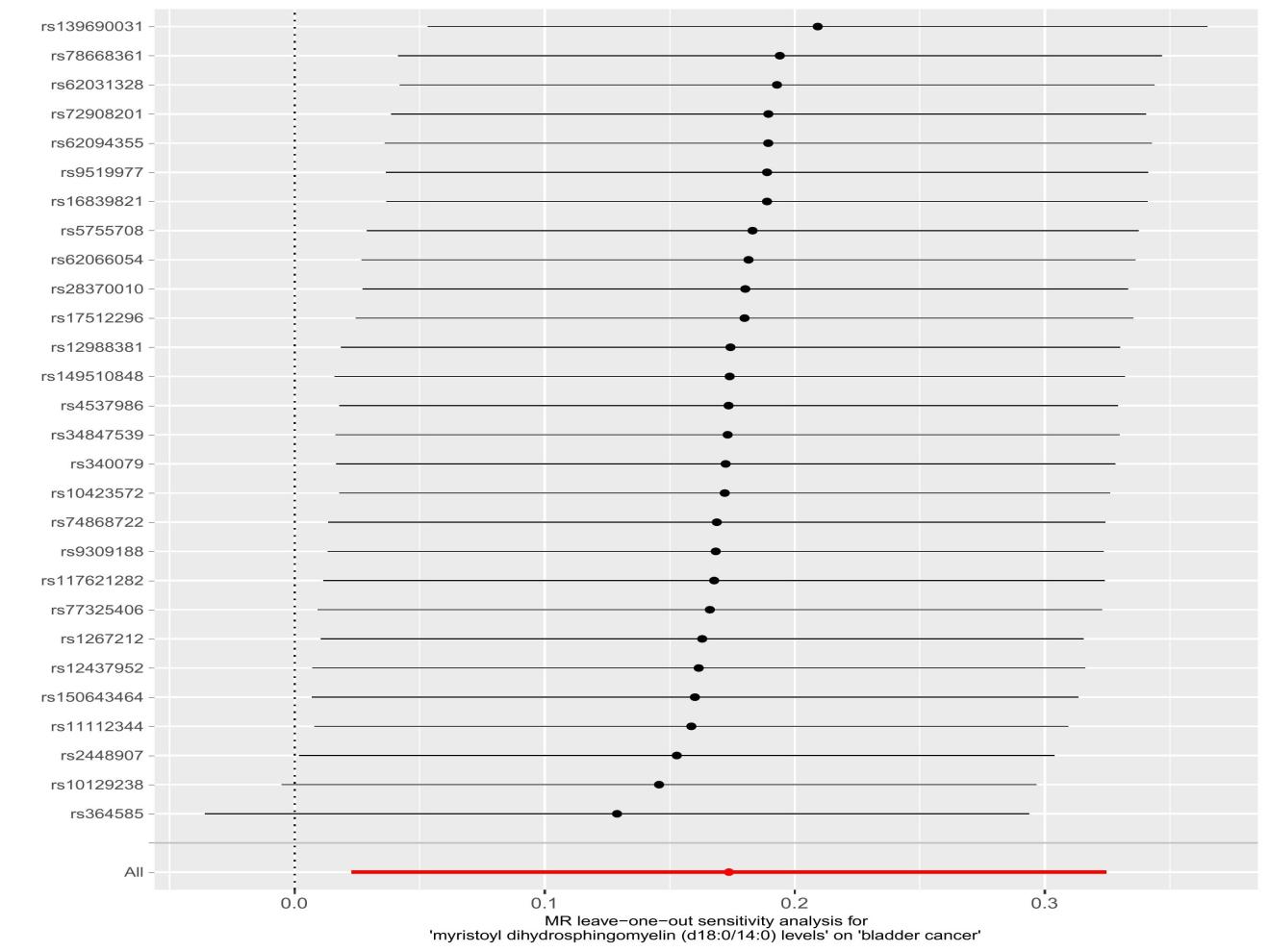


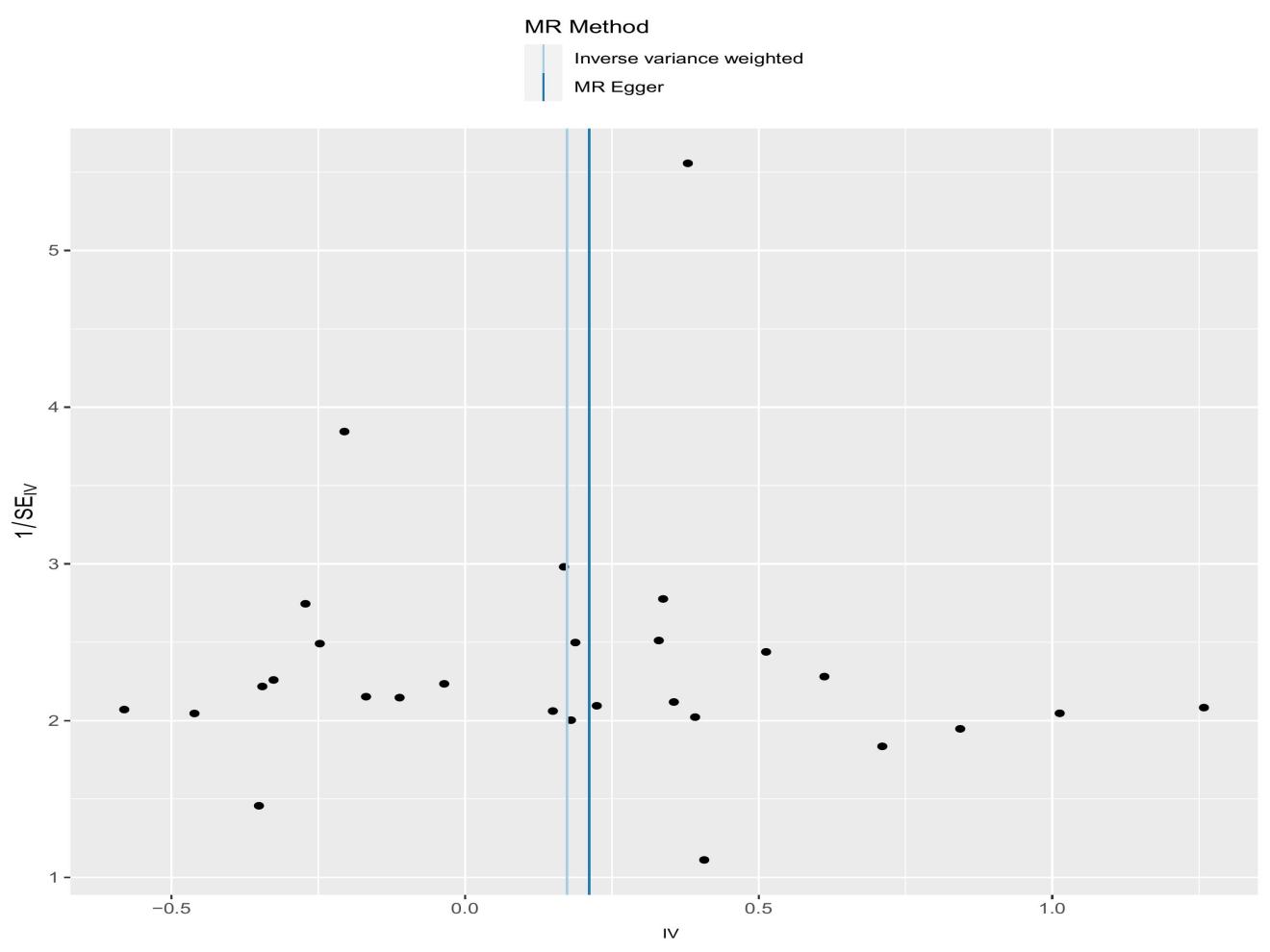


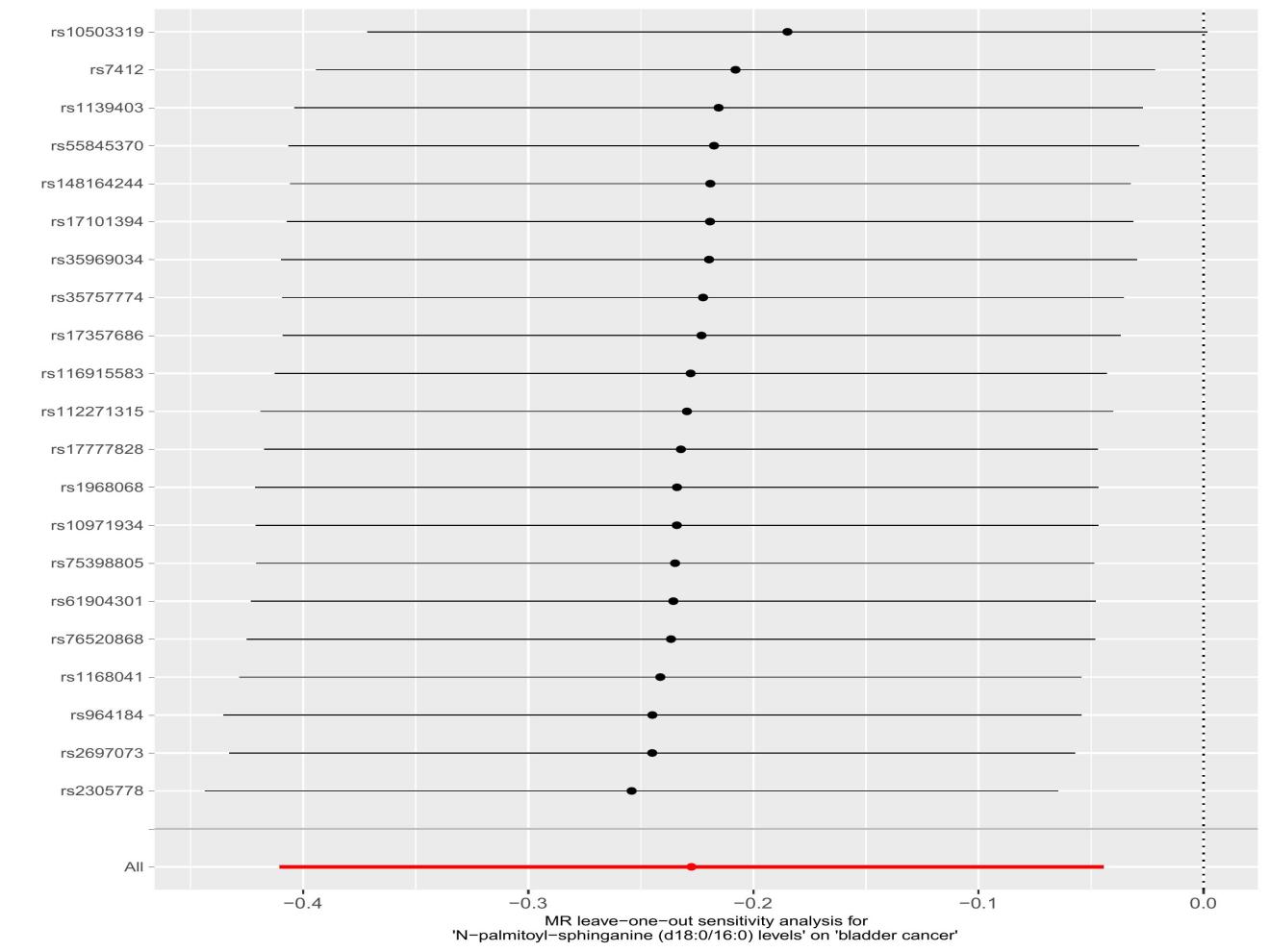


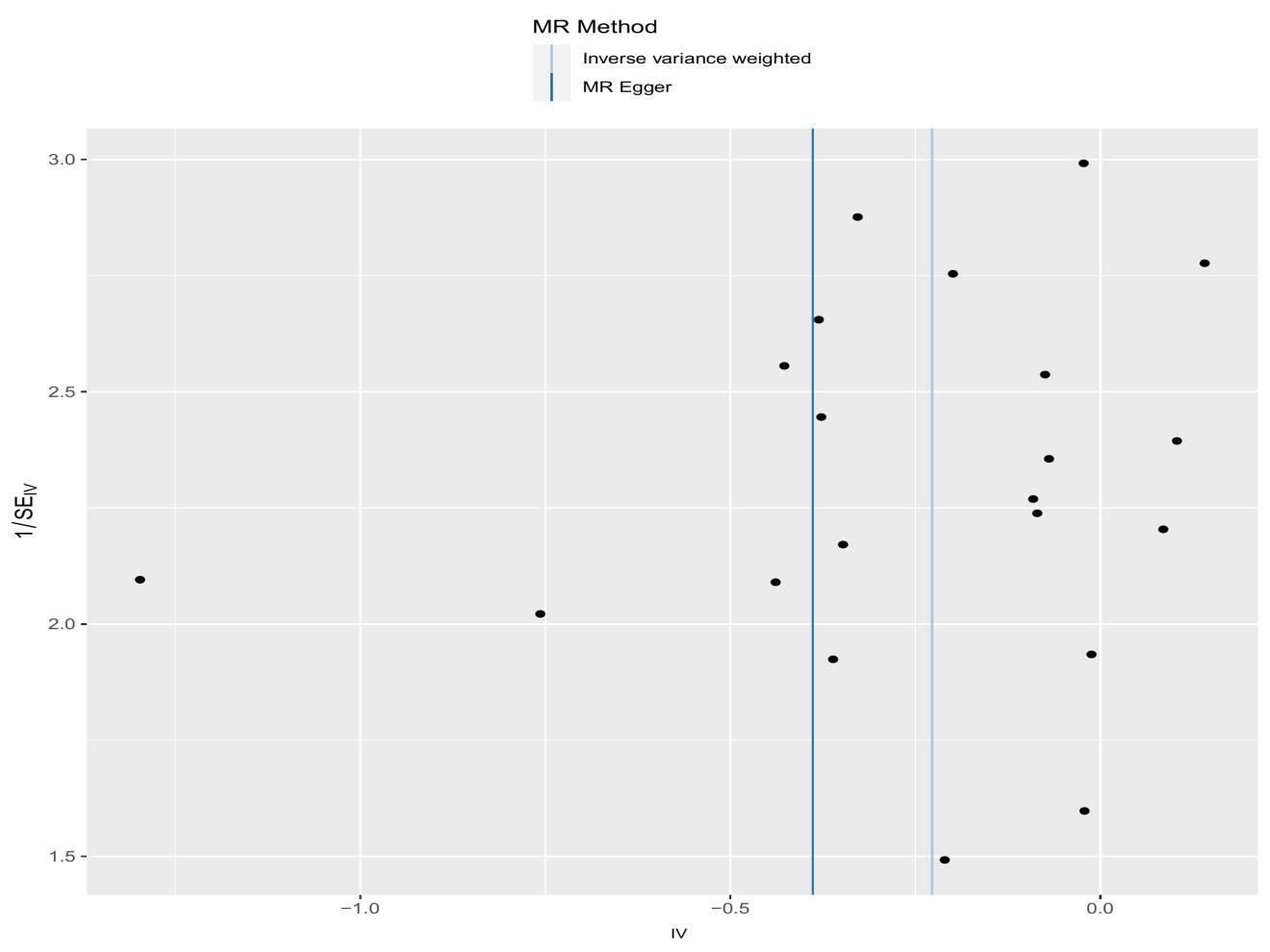


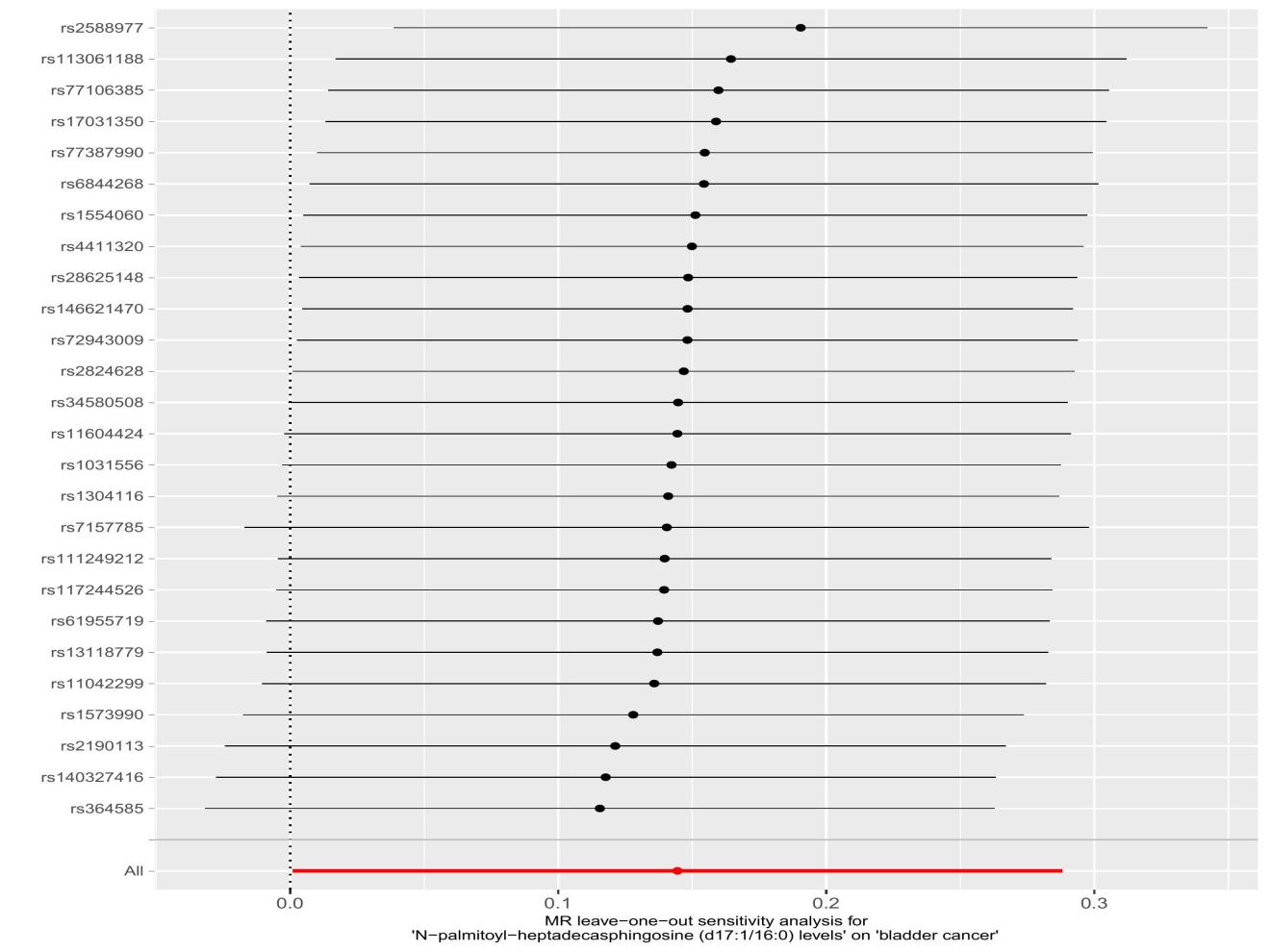


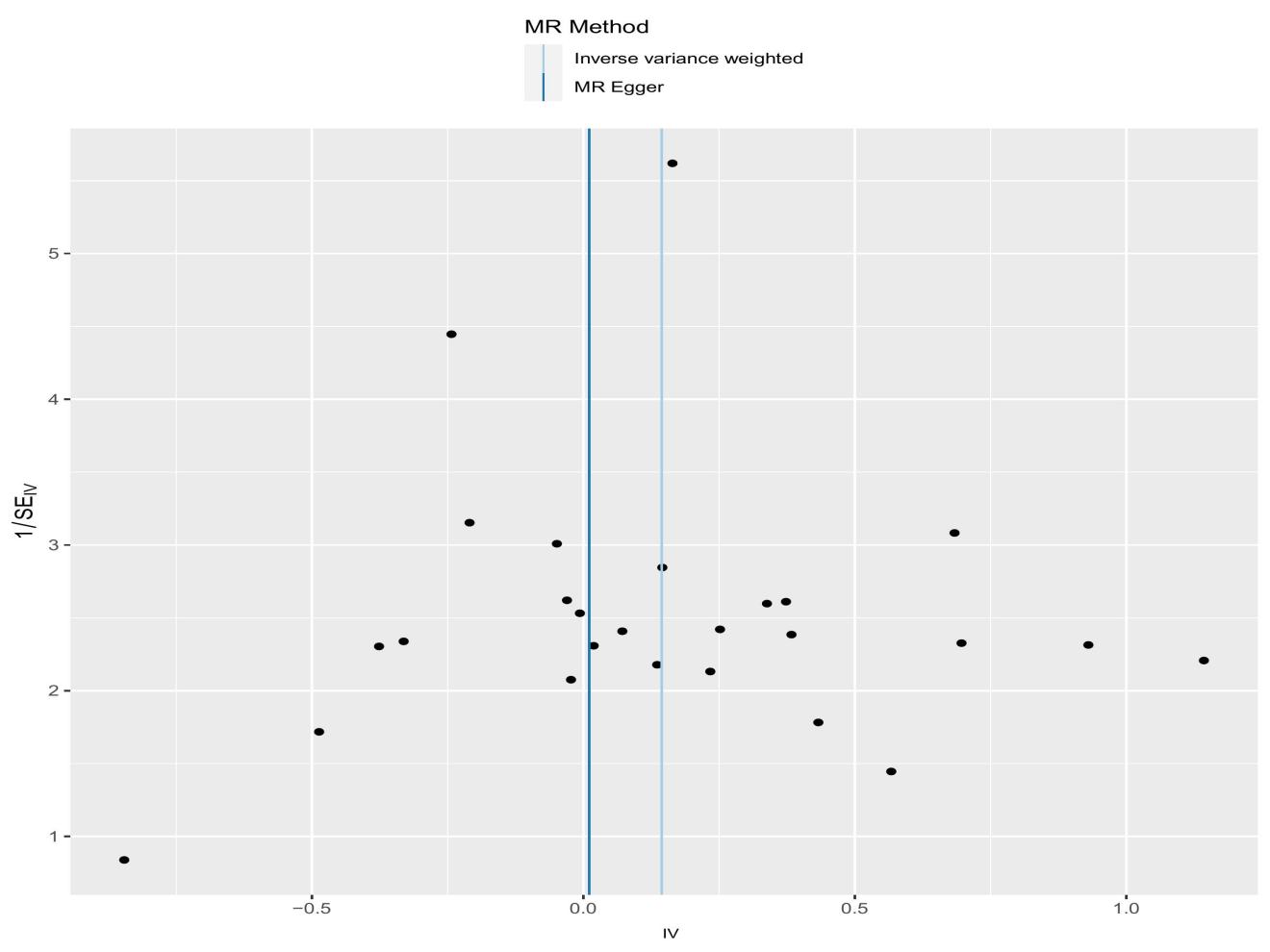


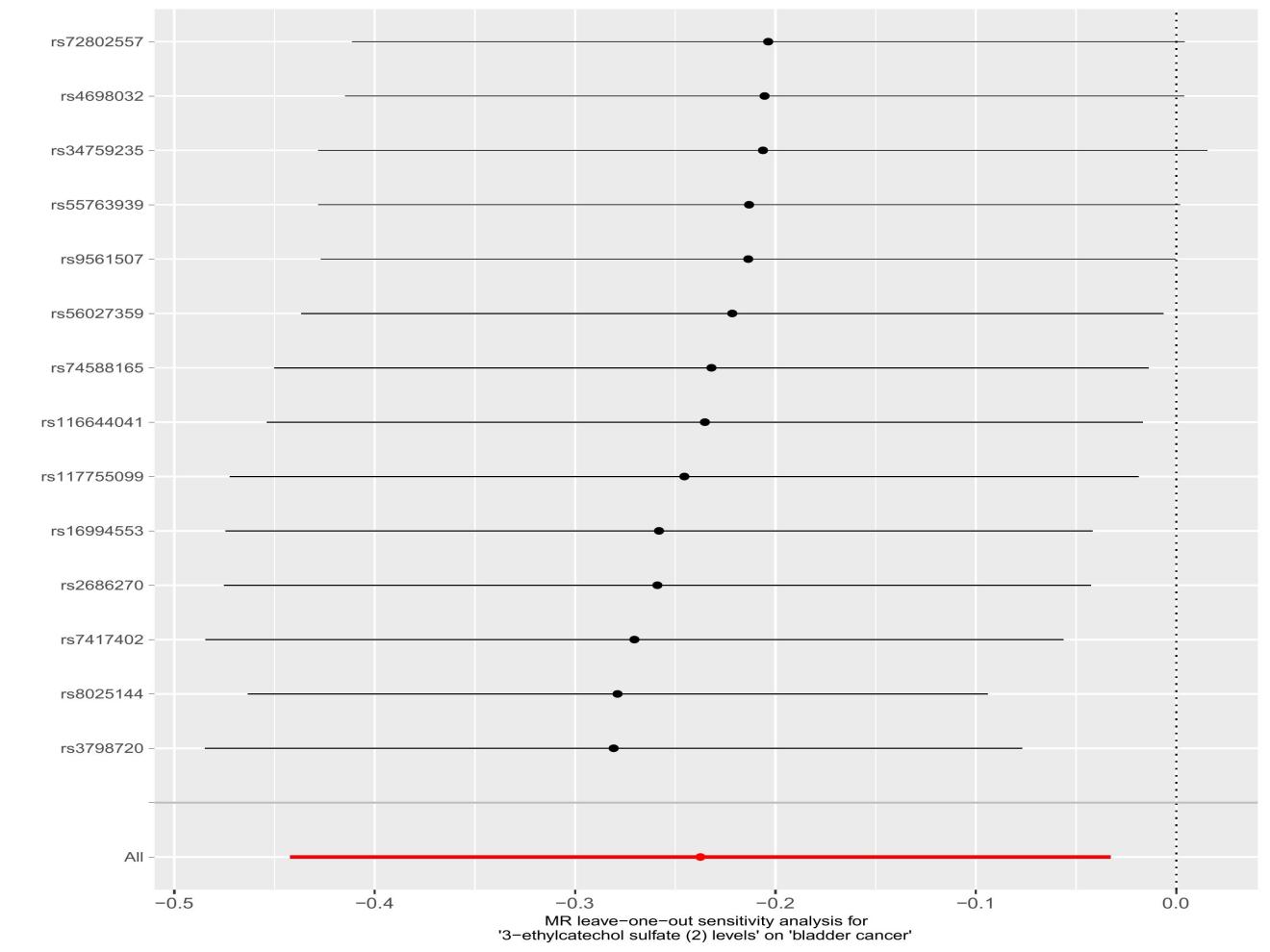


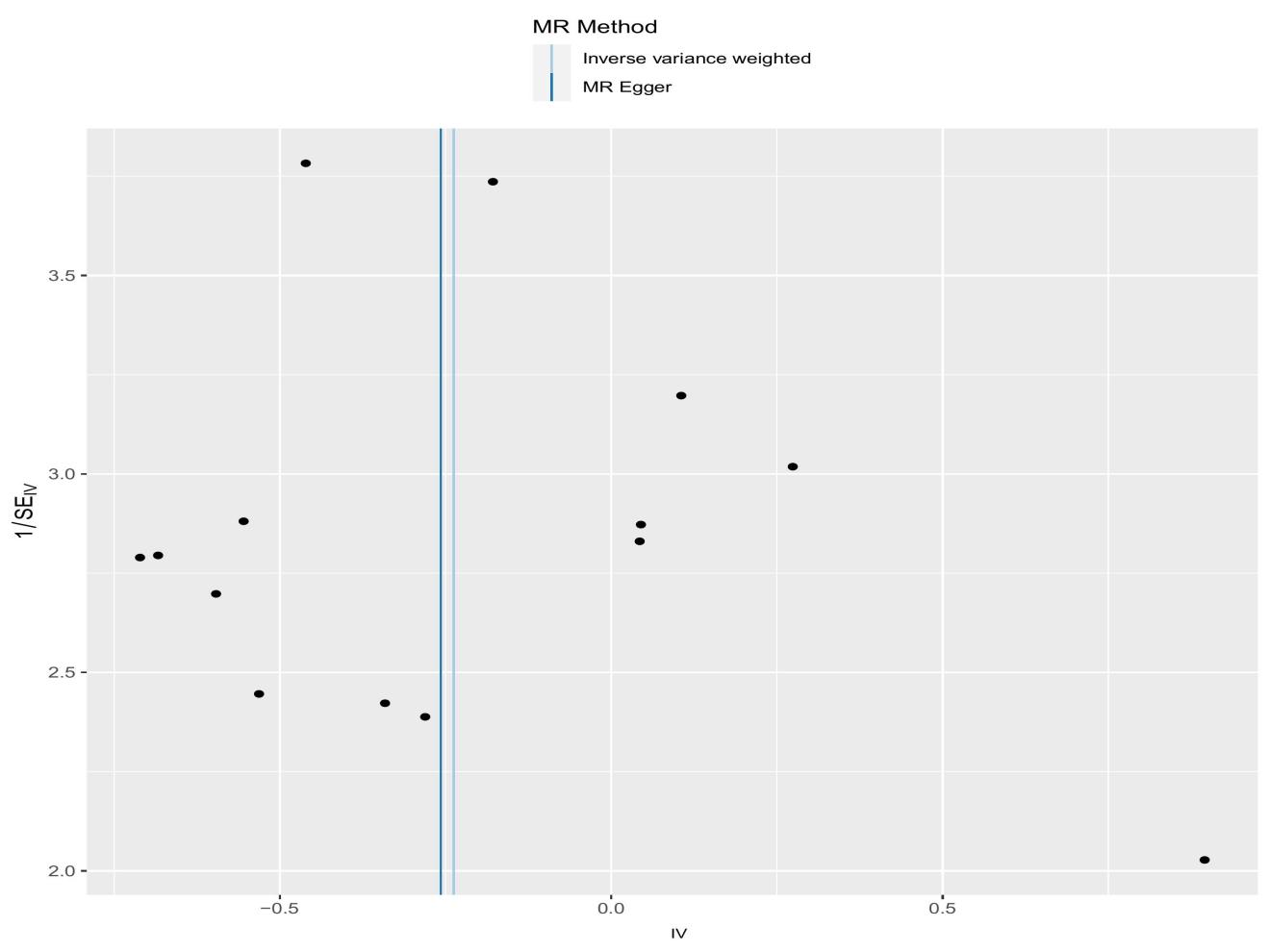


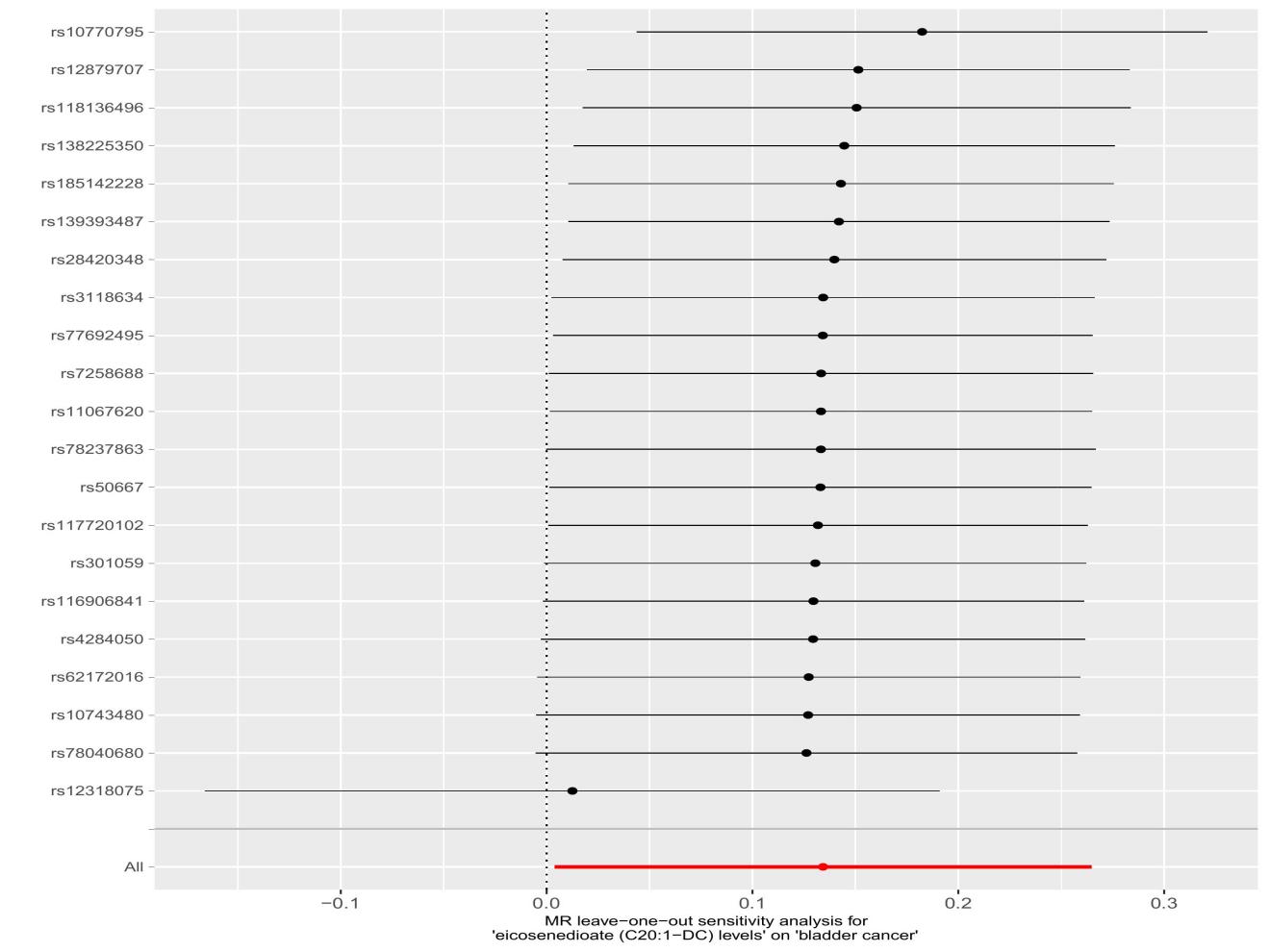


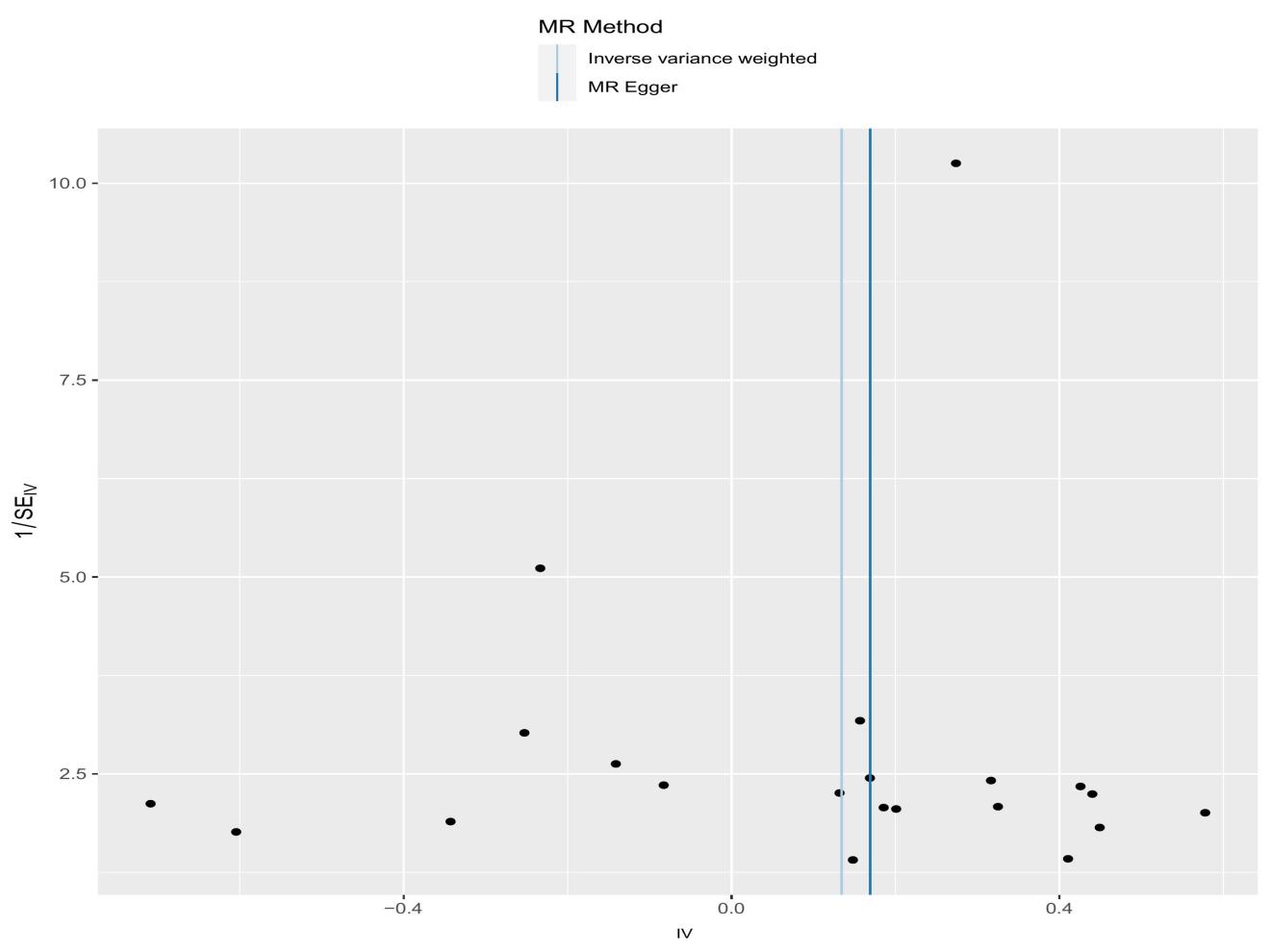


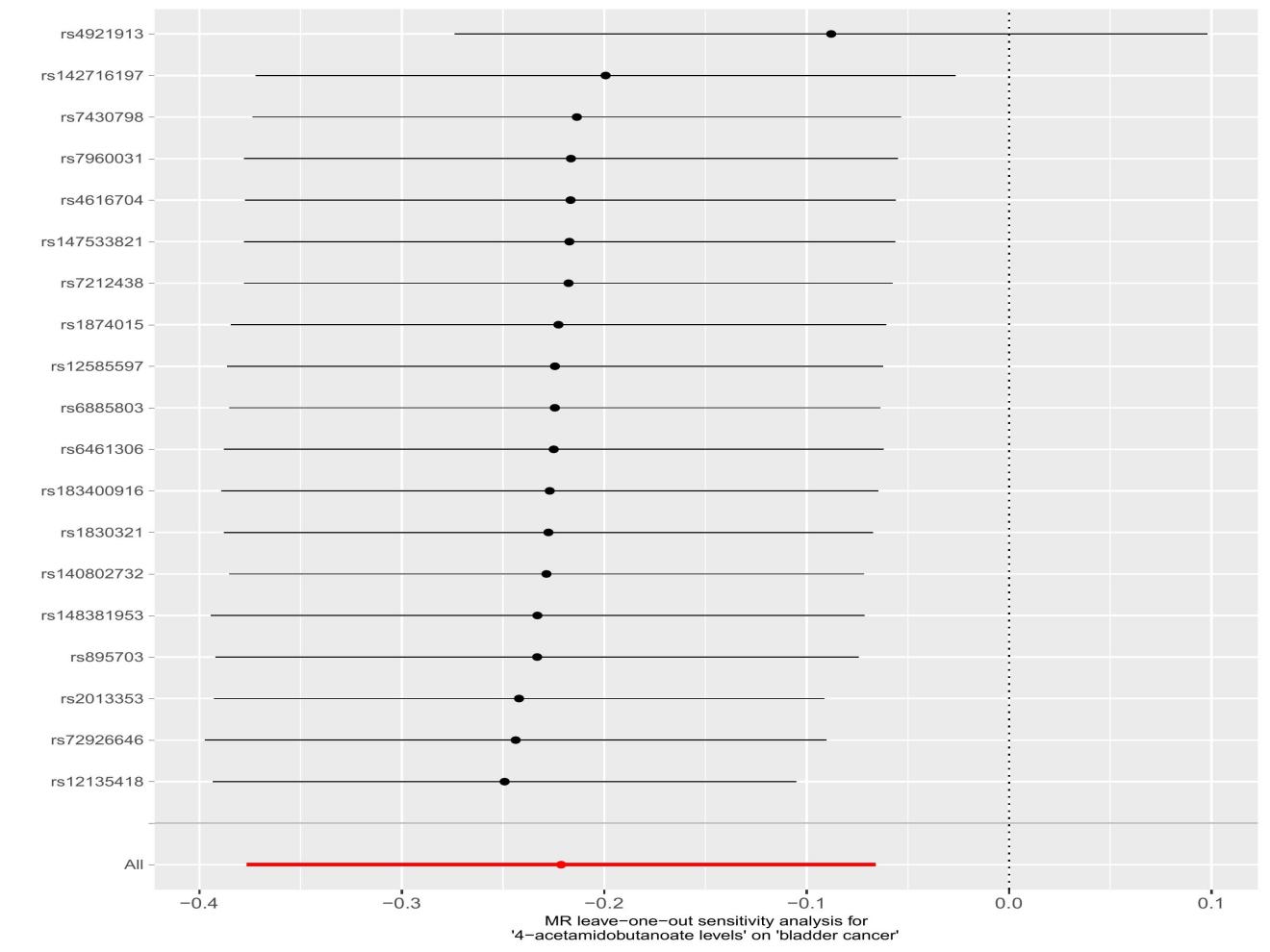


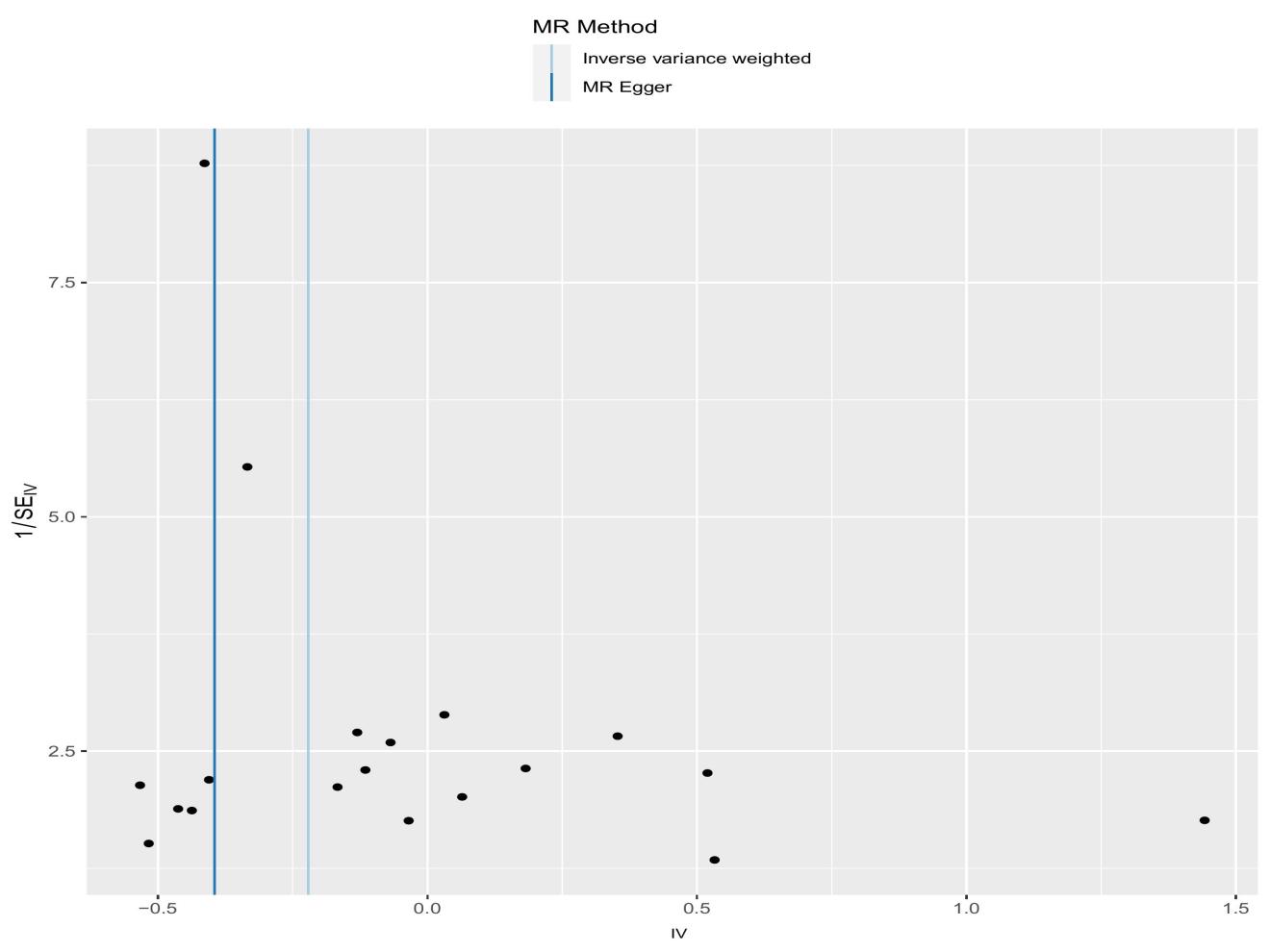


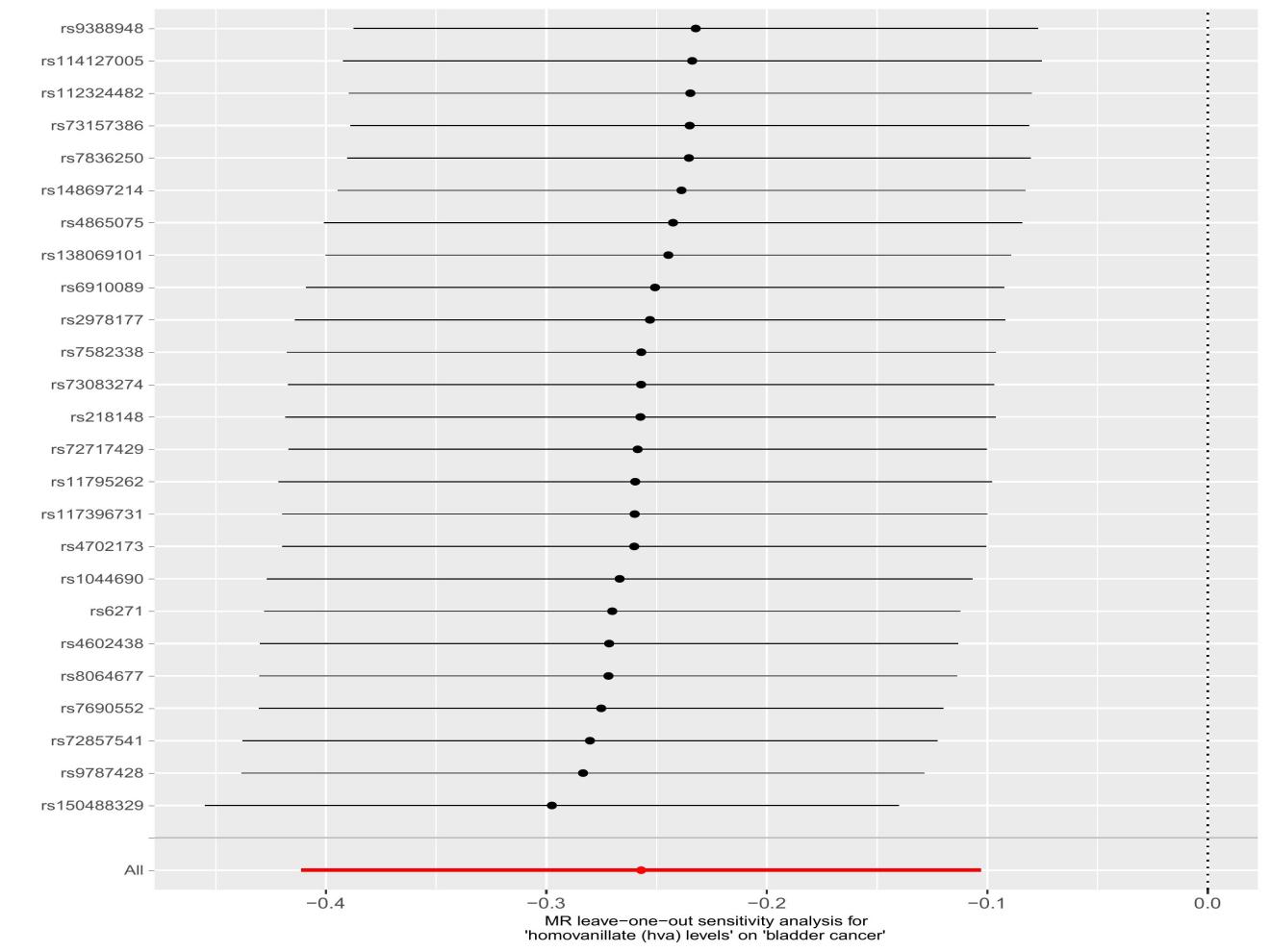


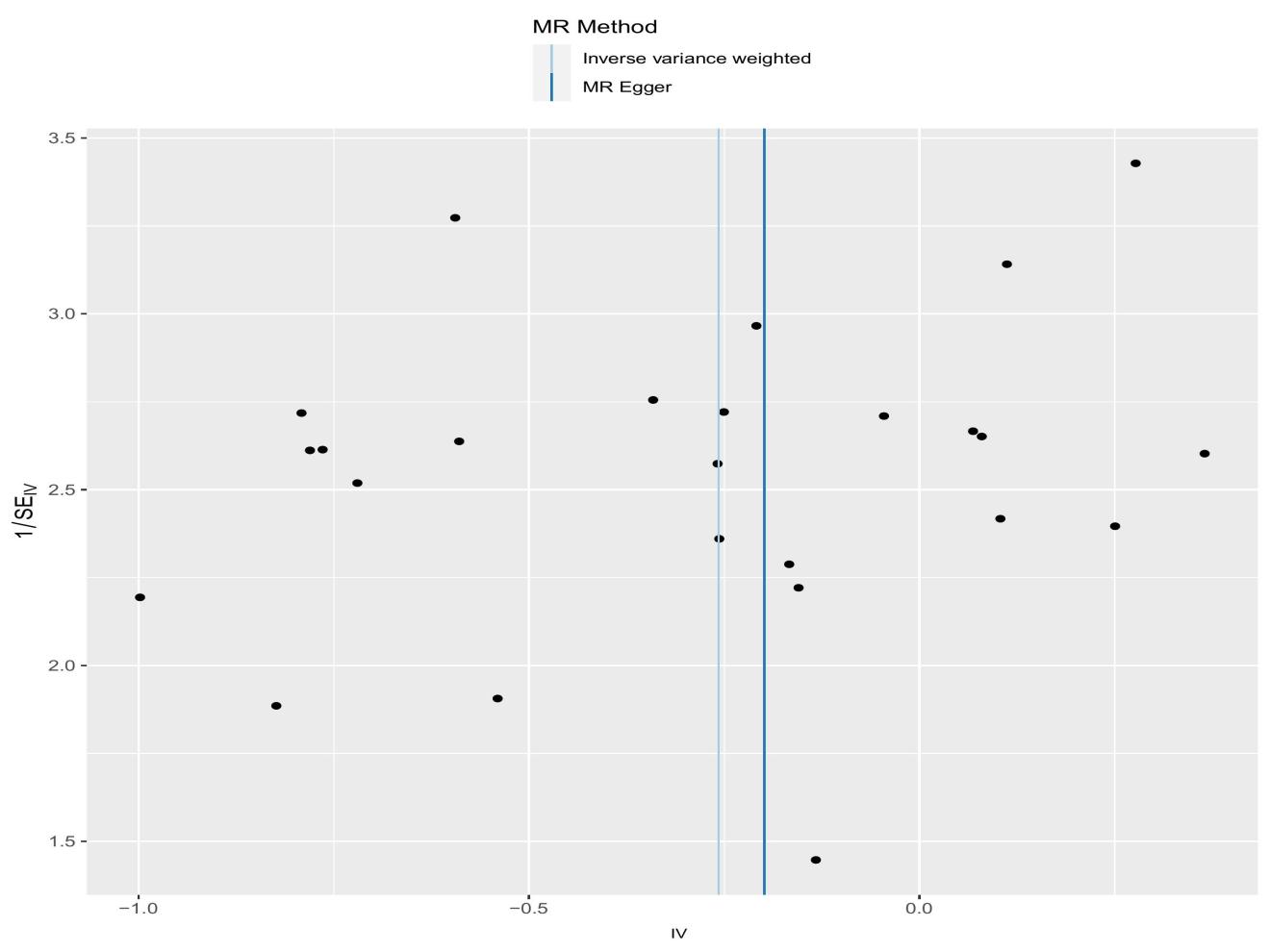


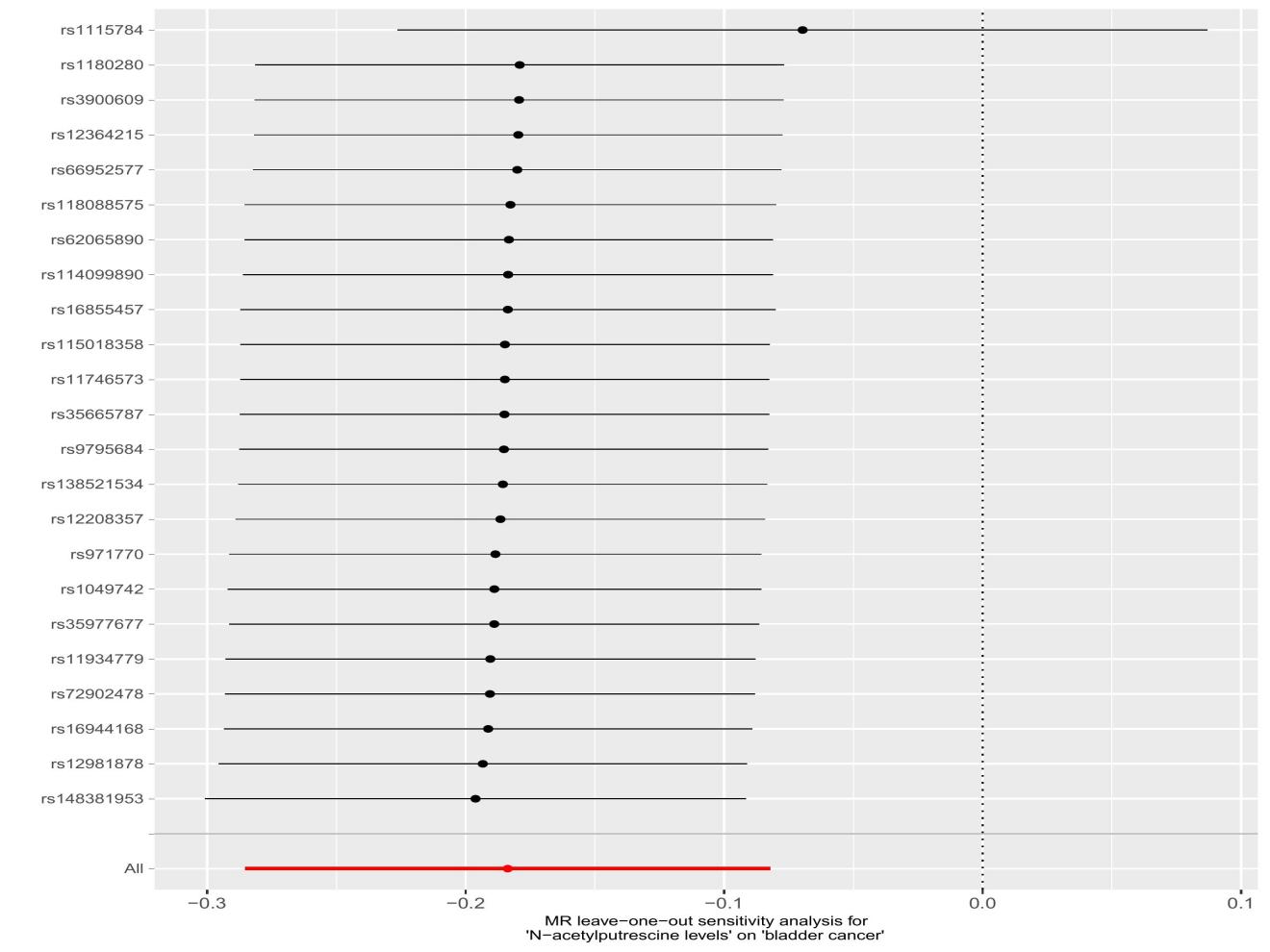


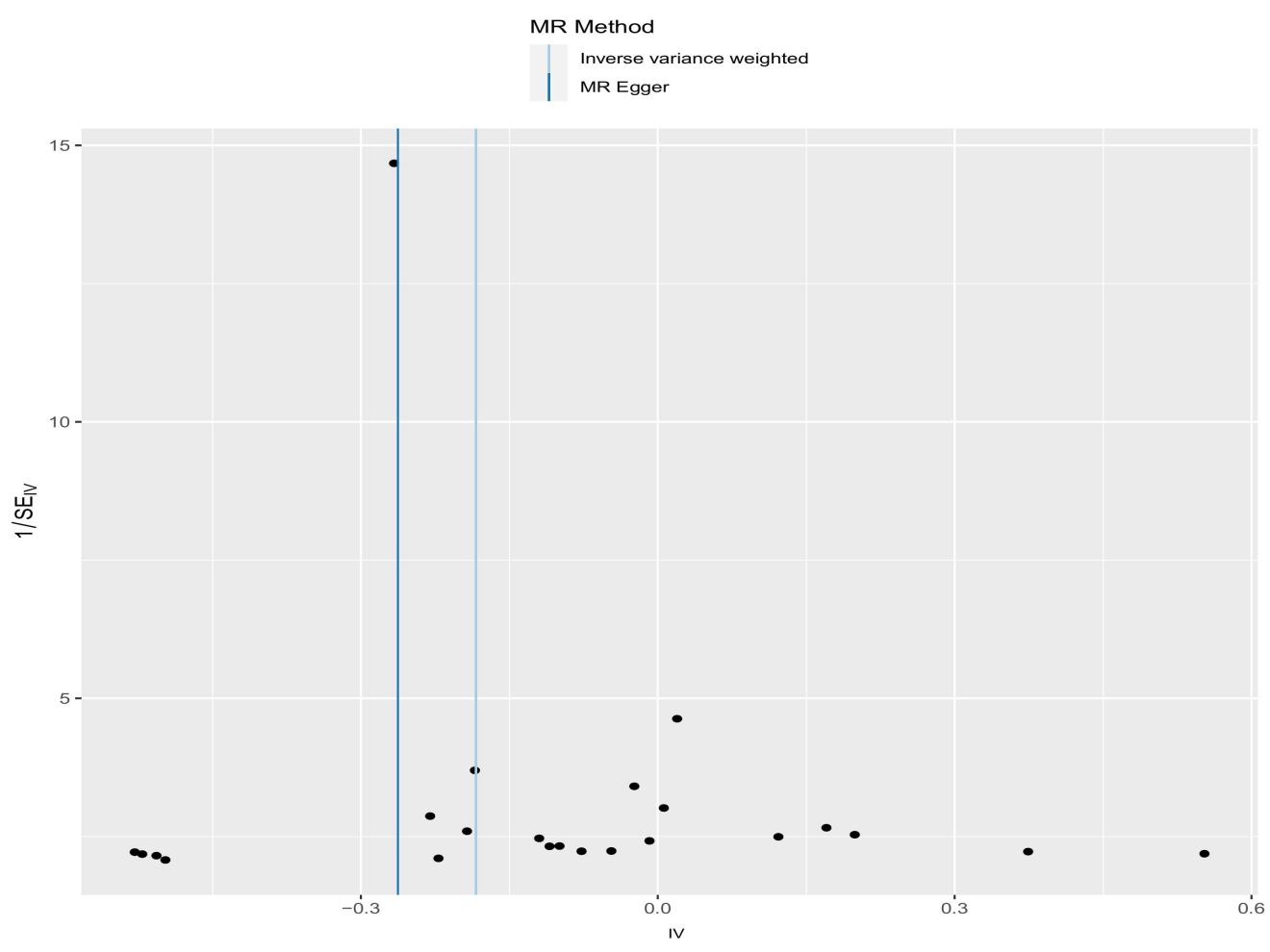


Kidney Cancer


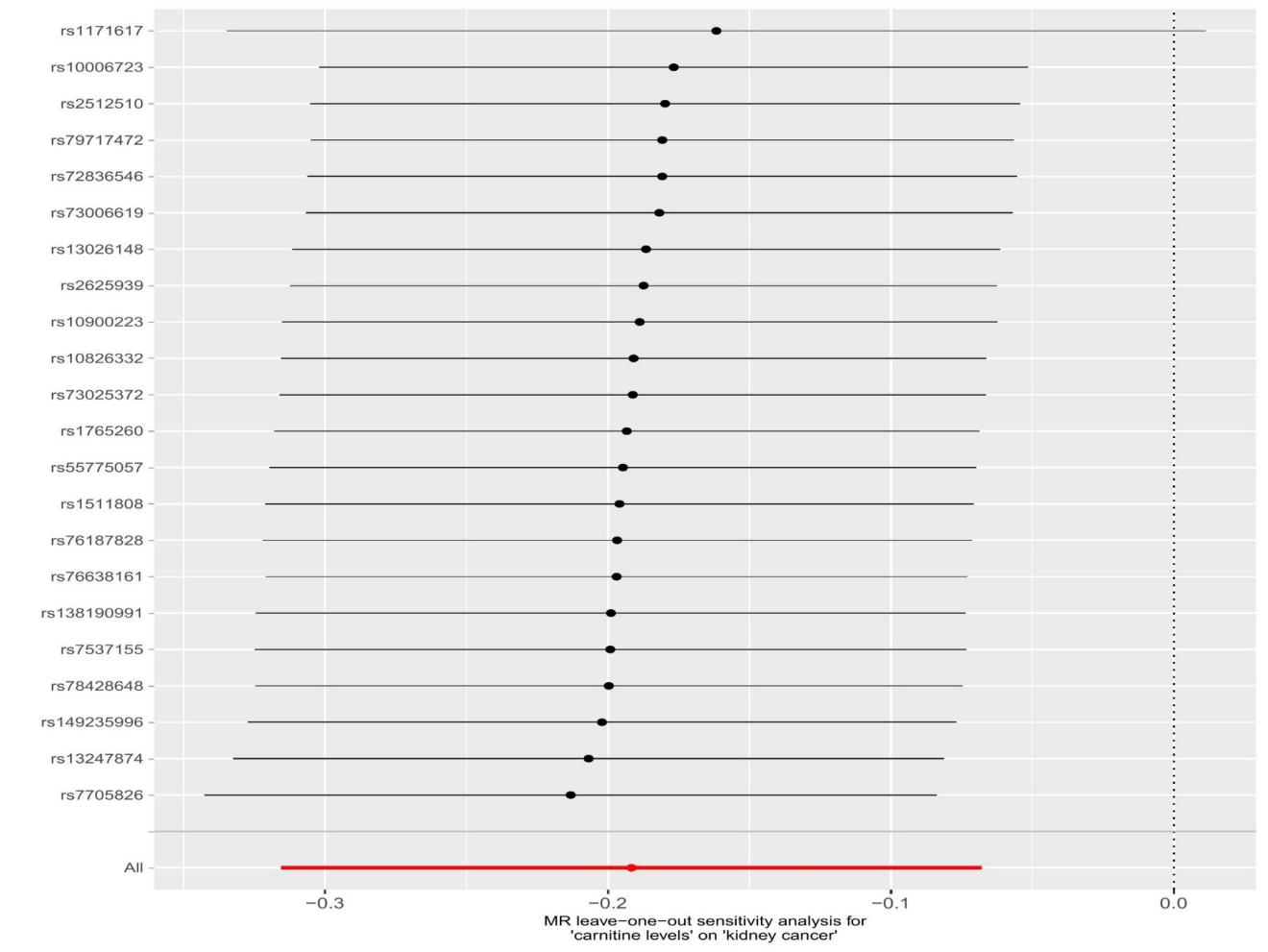


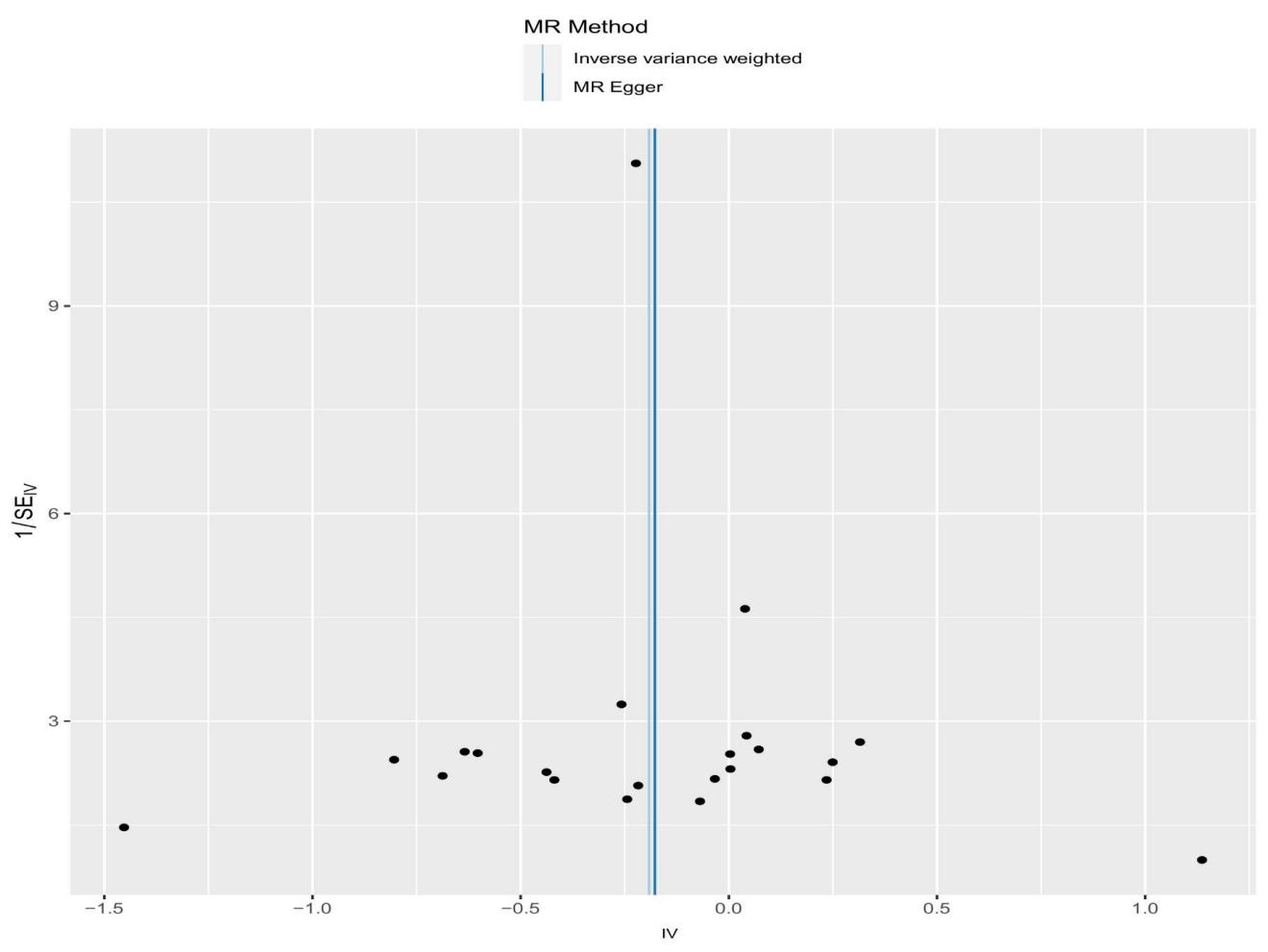


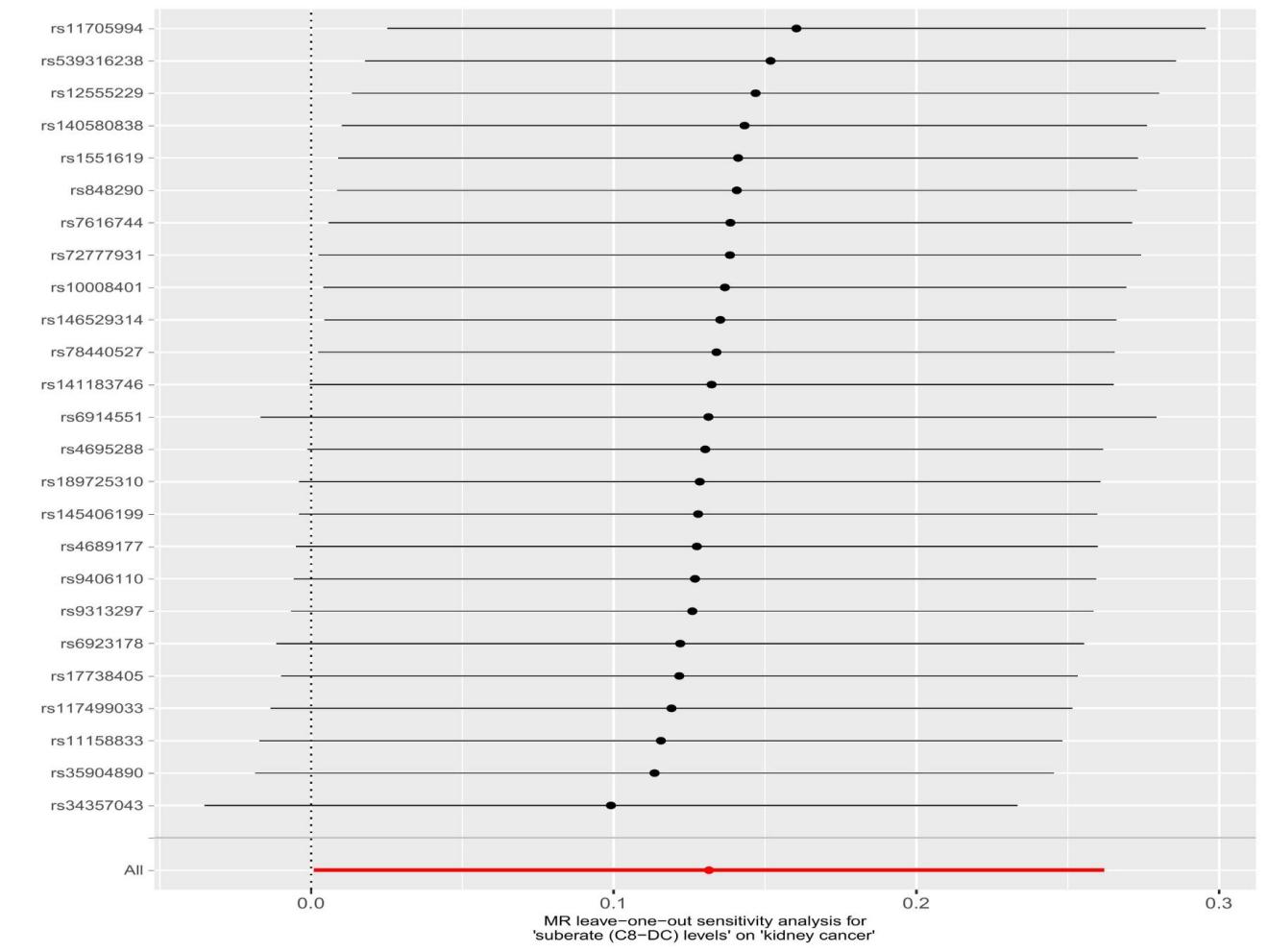


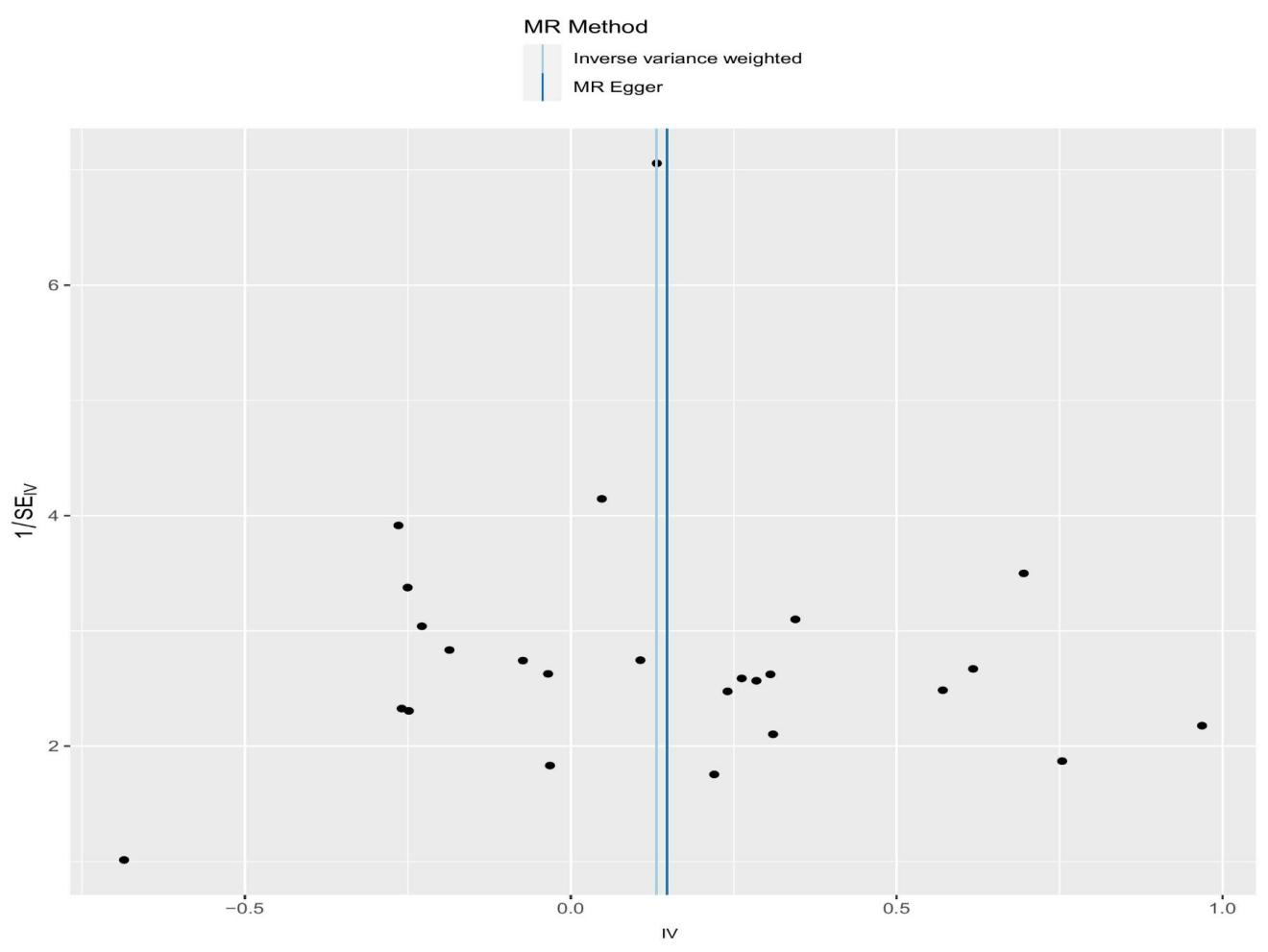


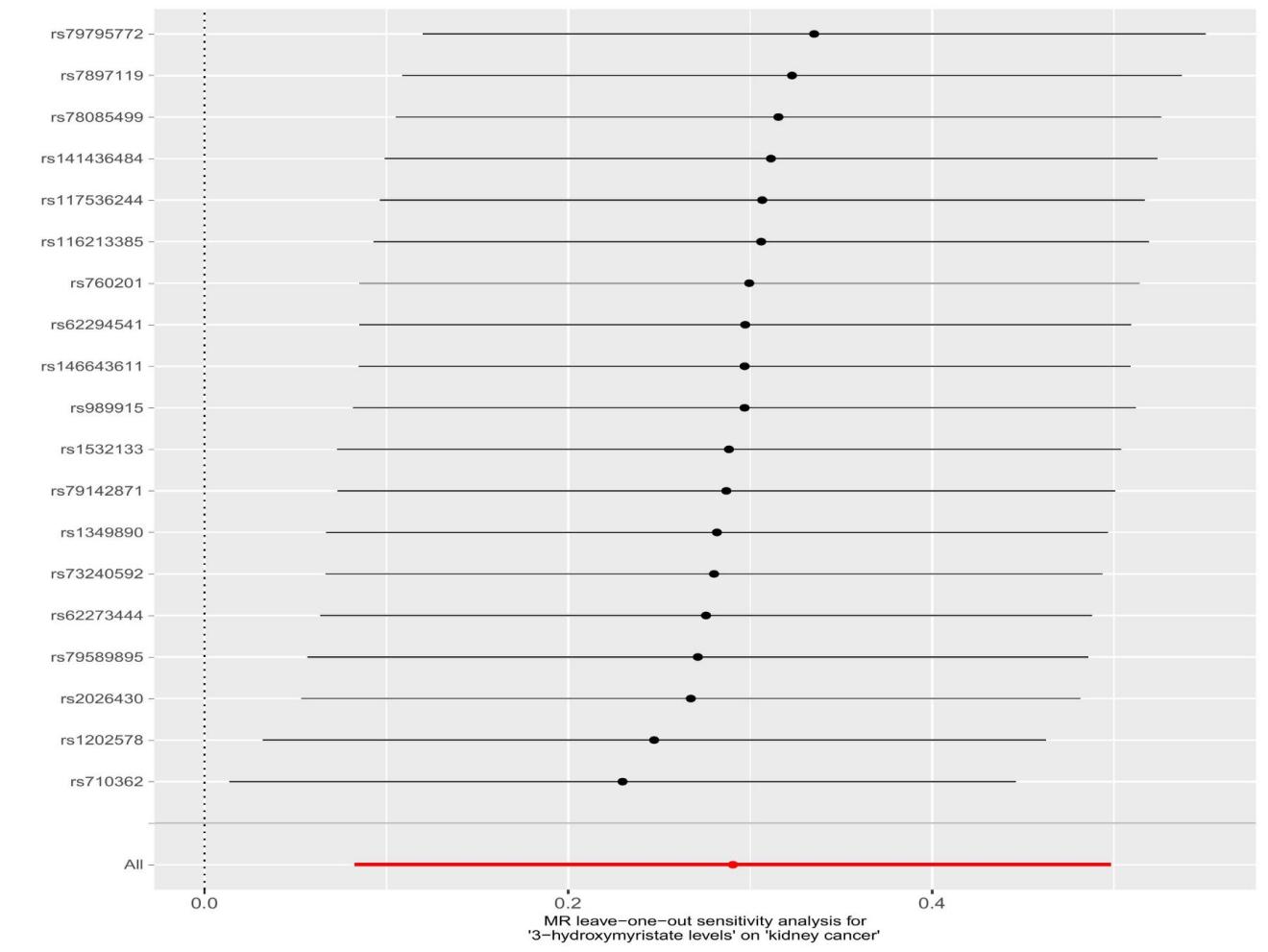


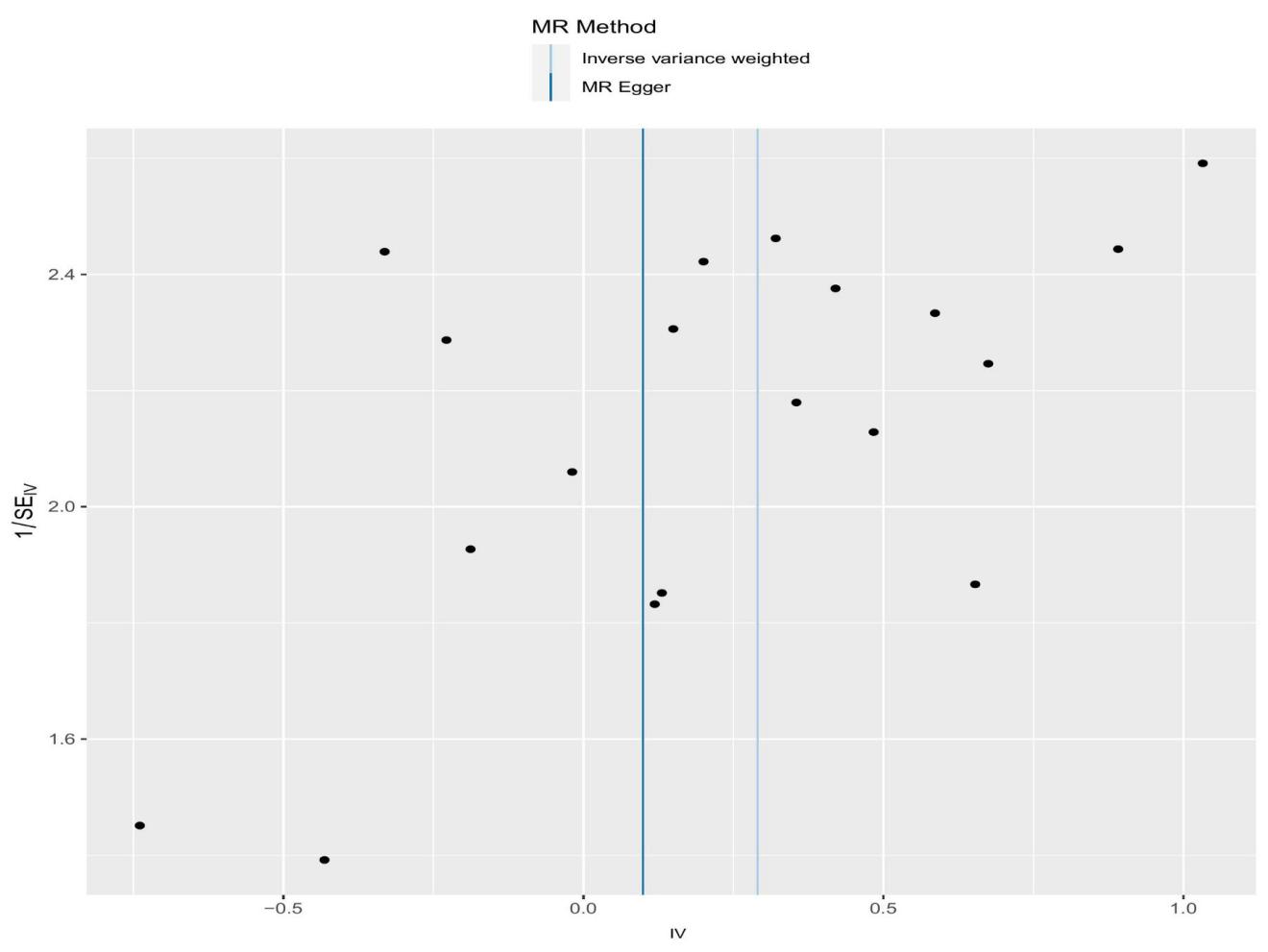


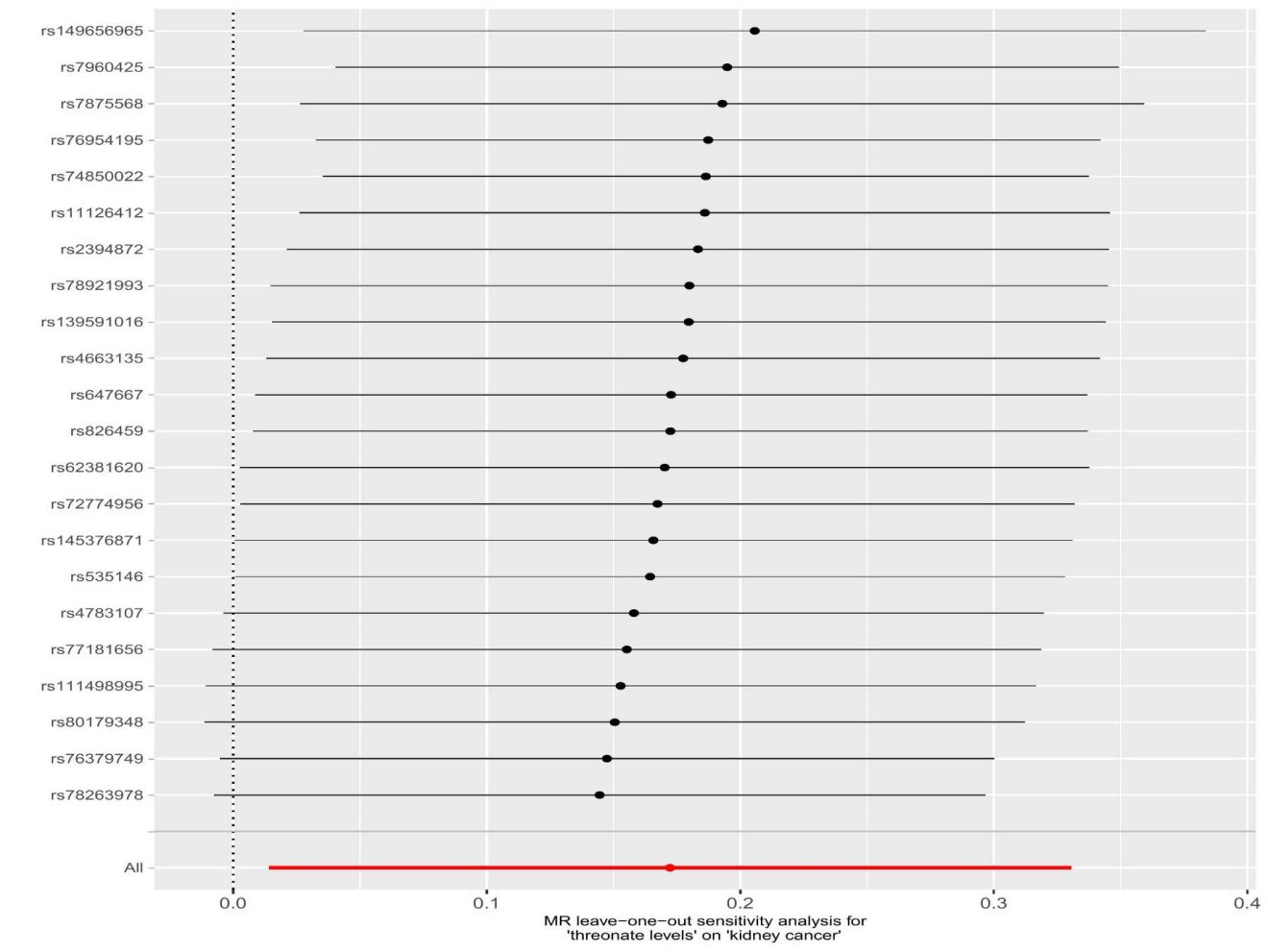


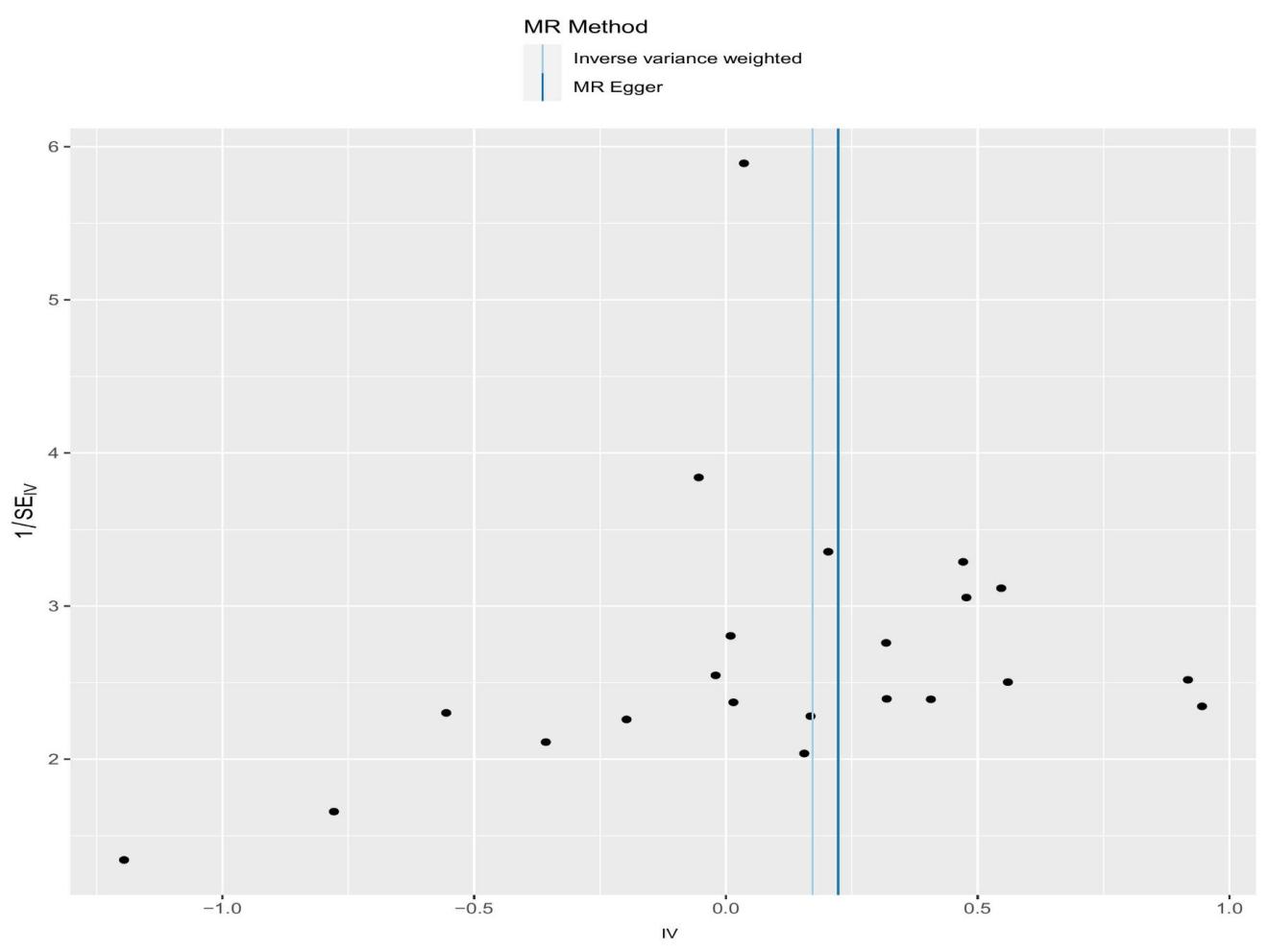


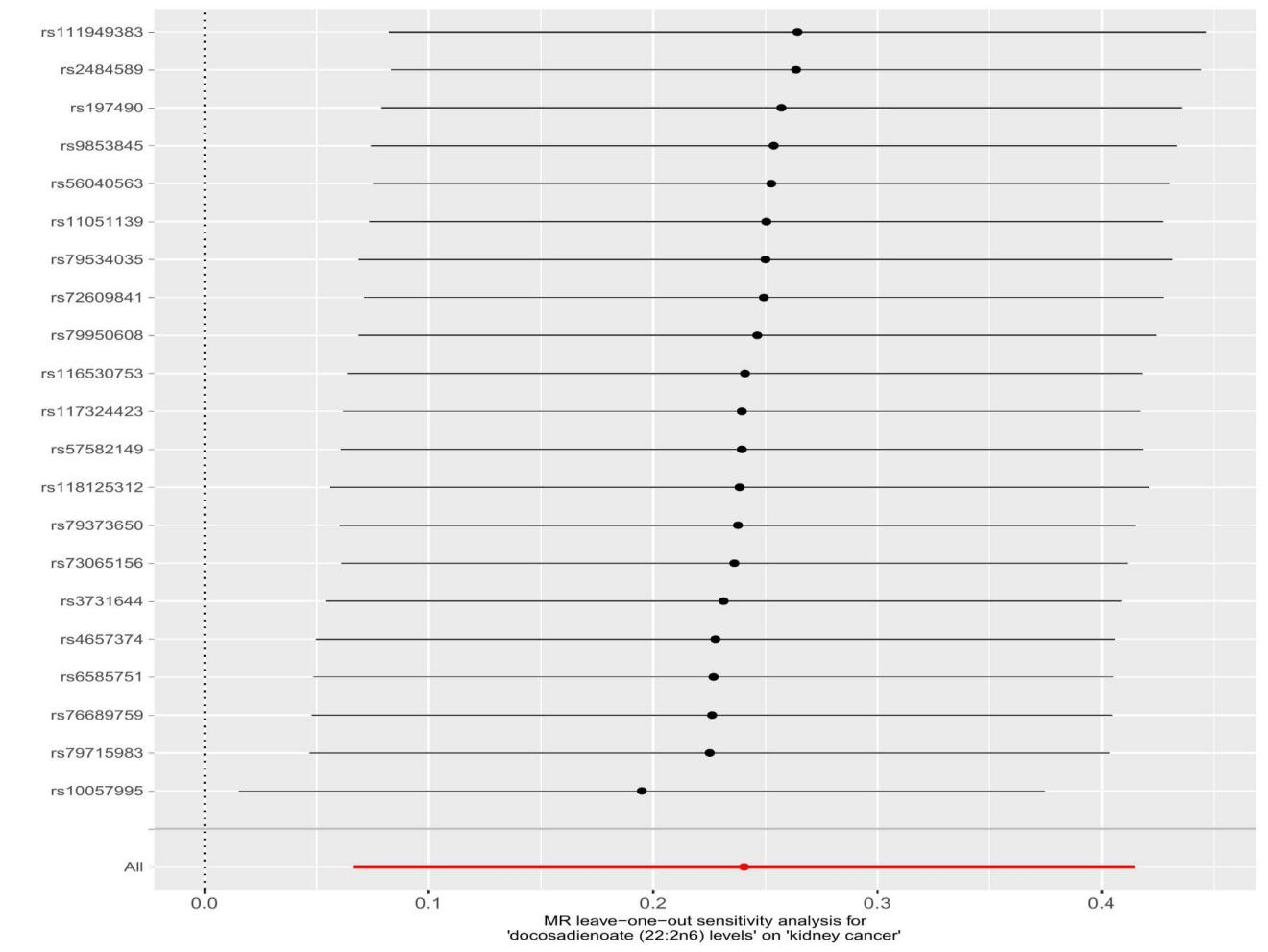


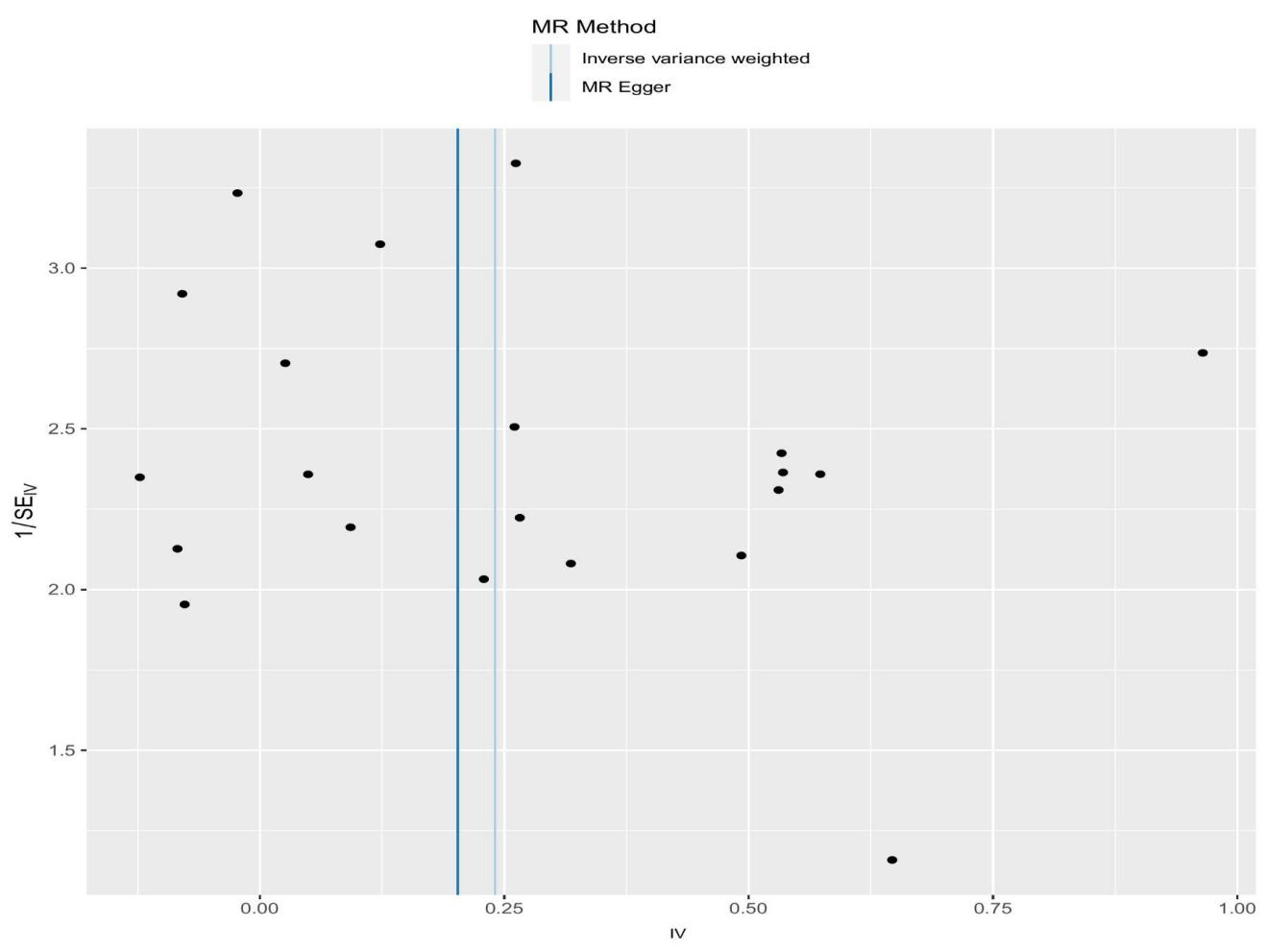


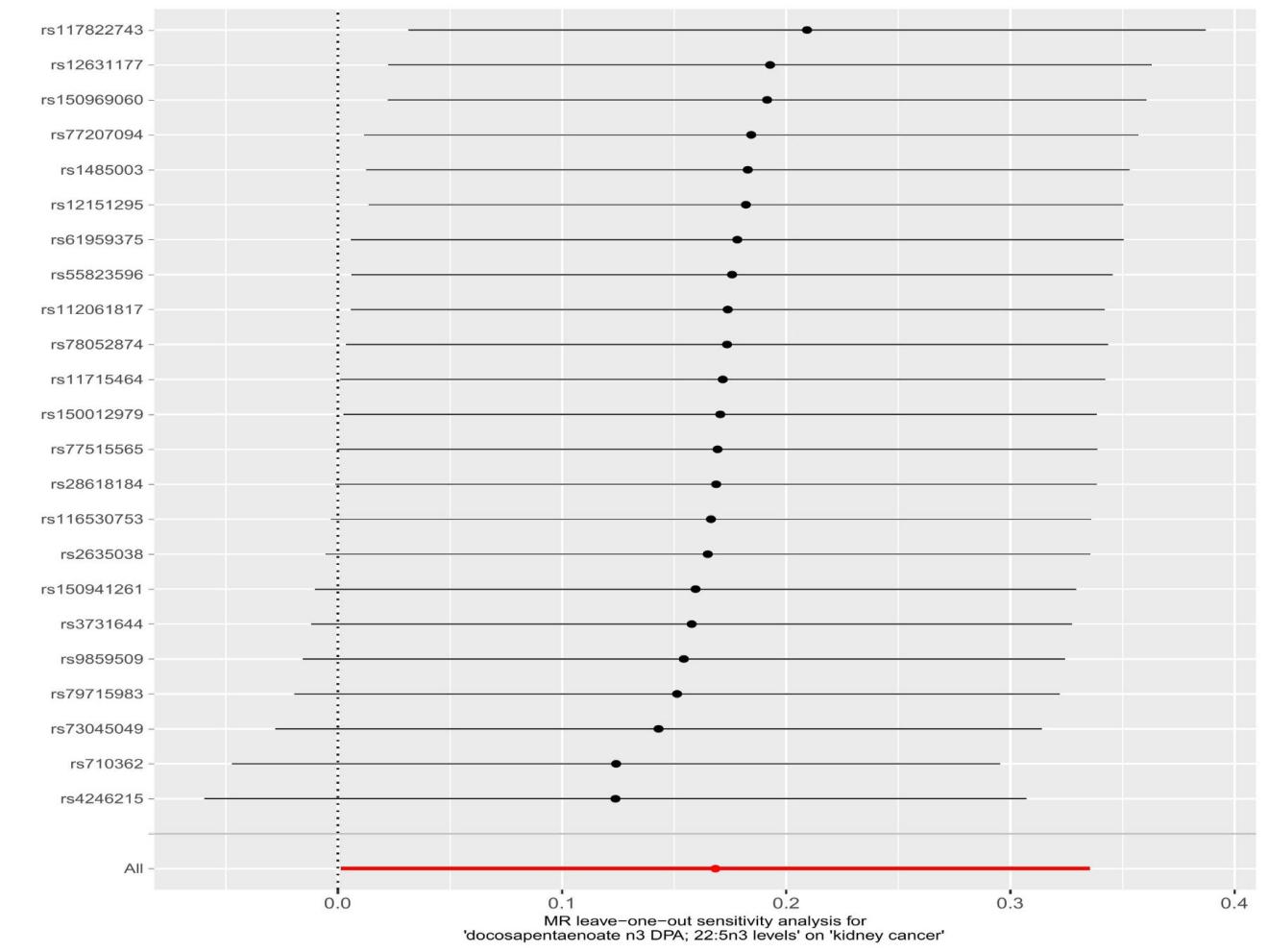


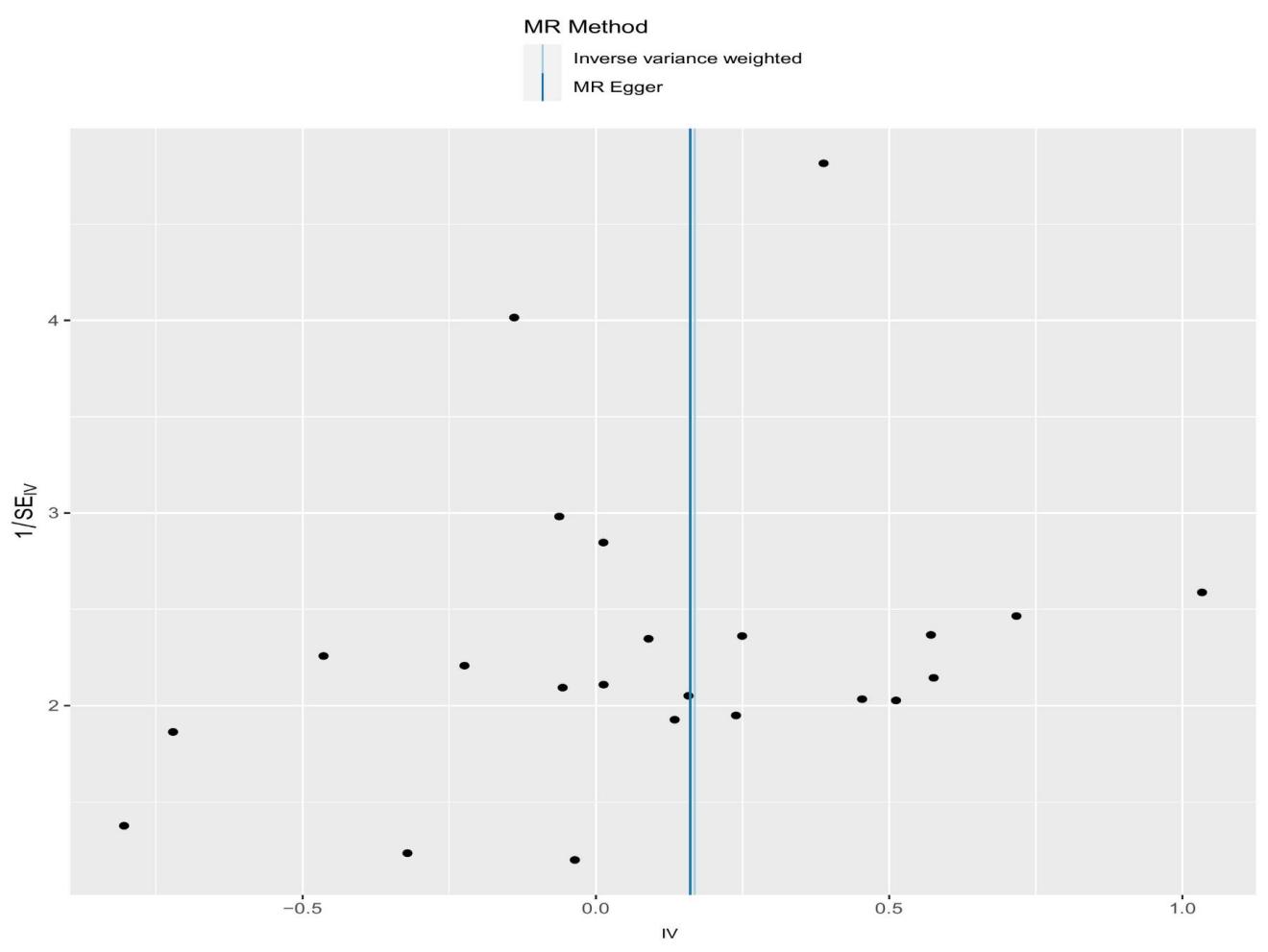


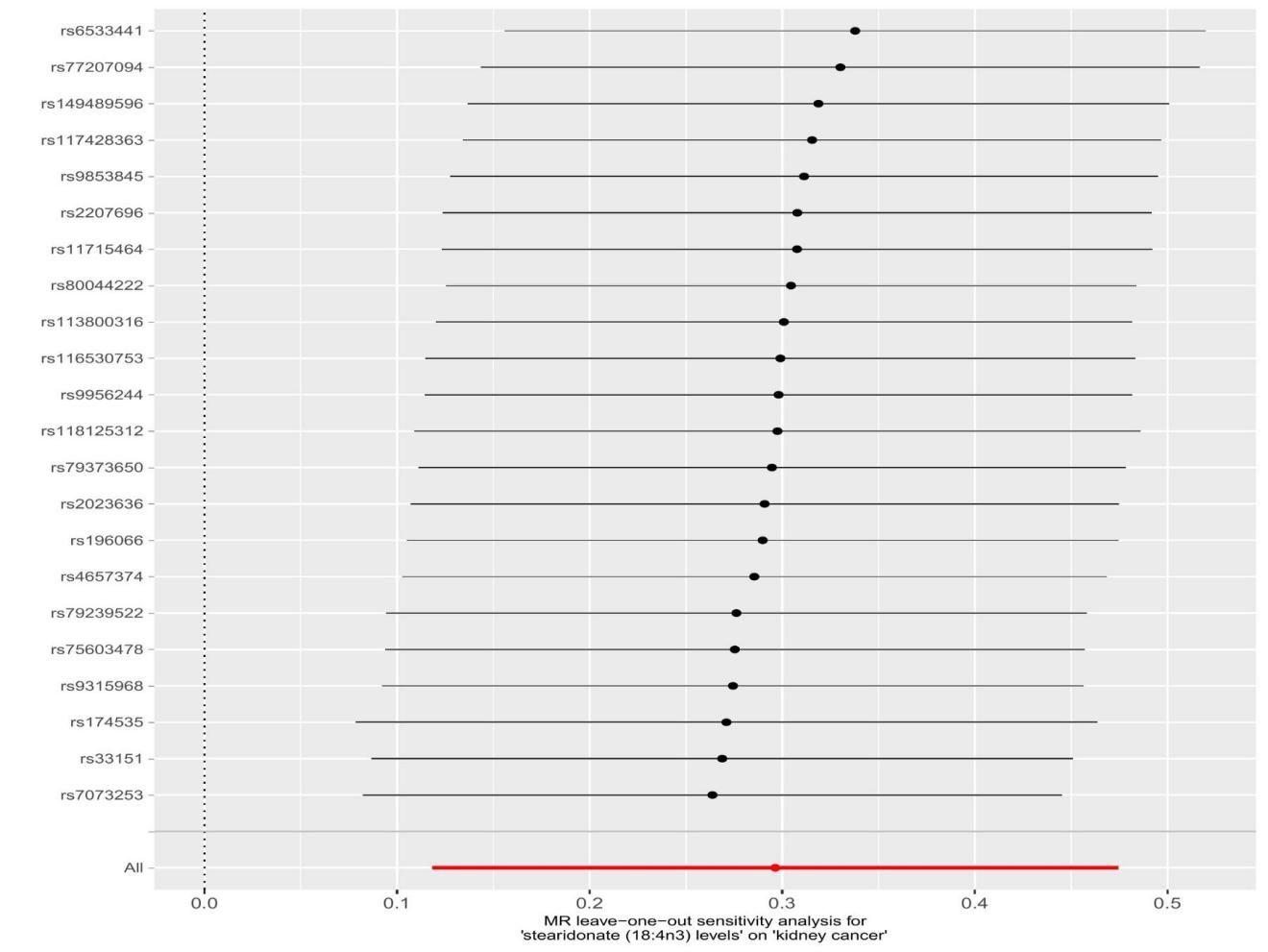


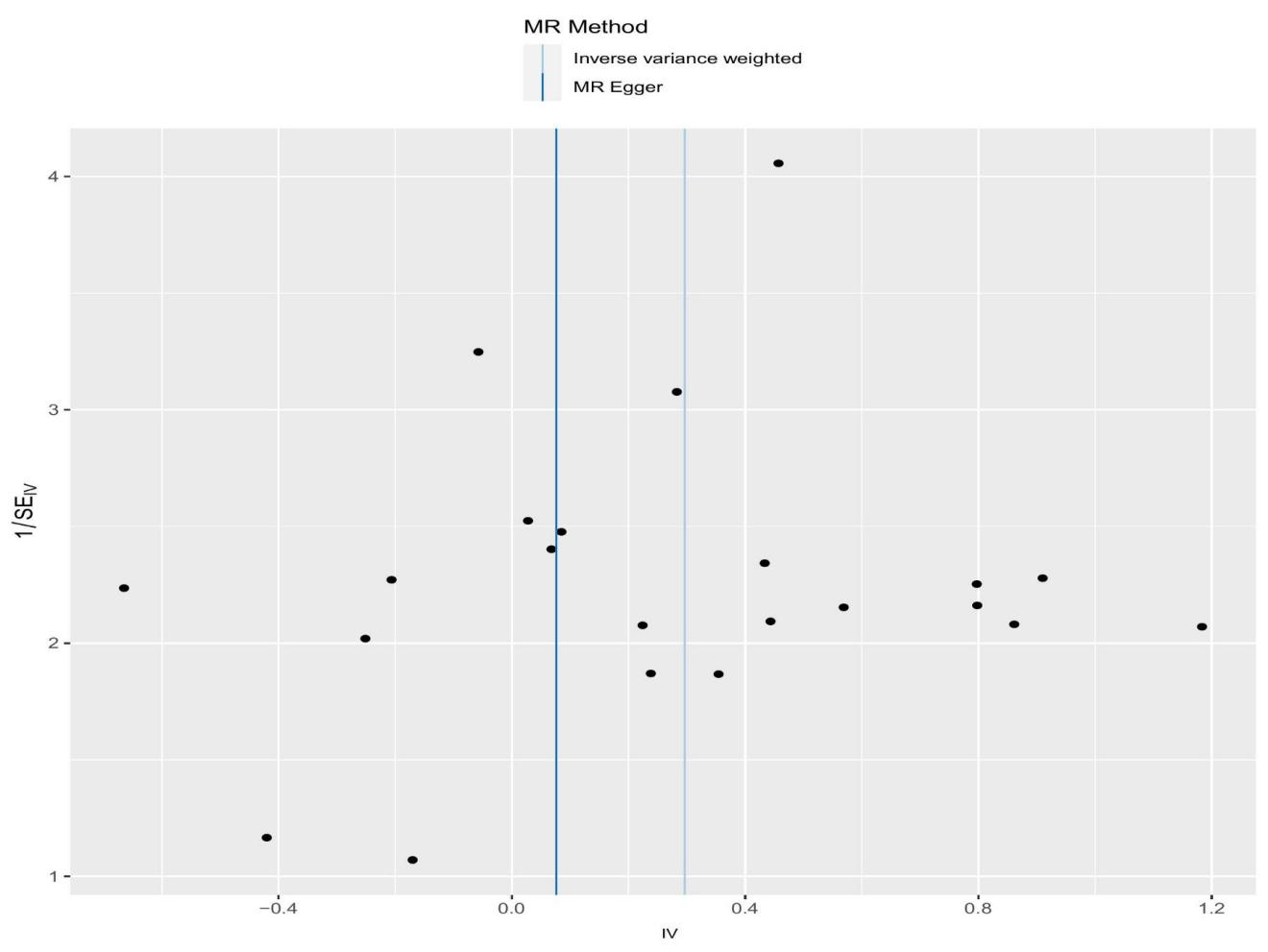


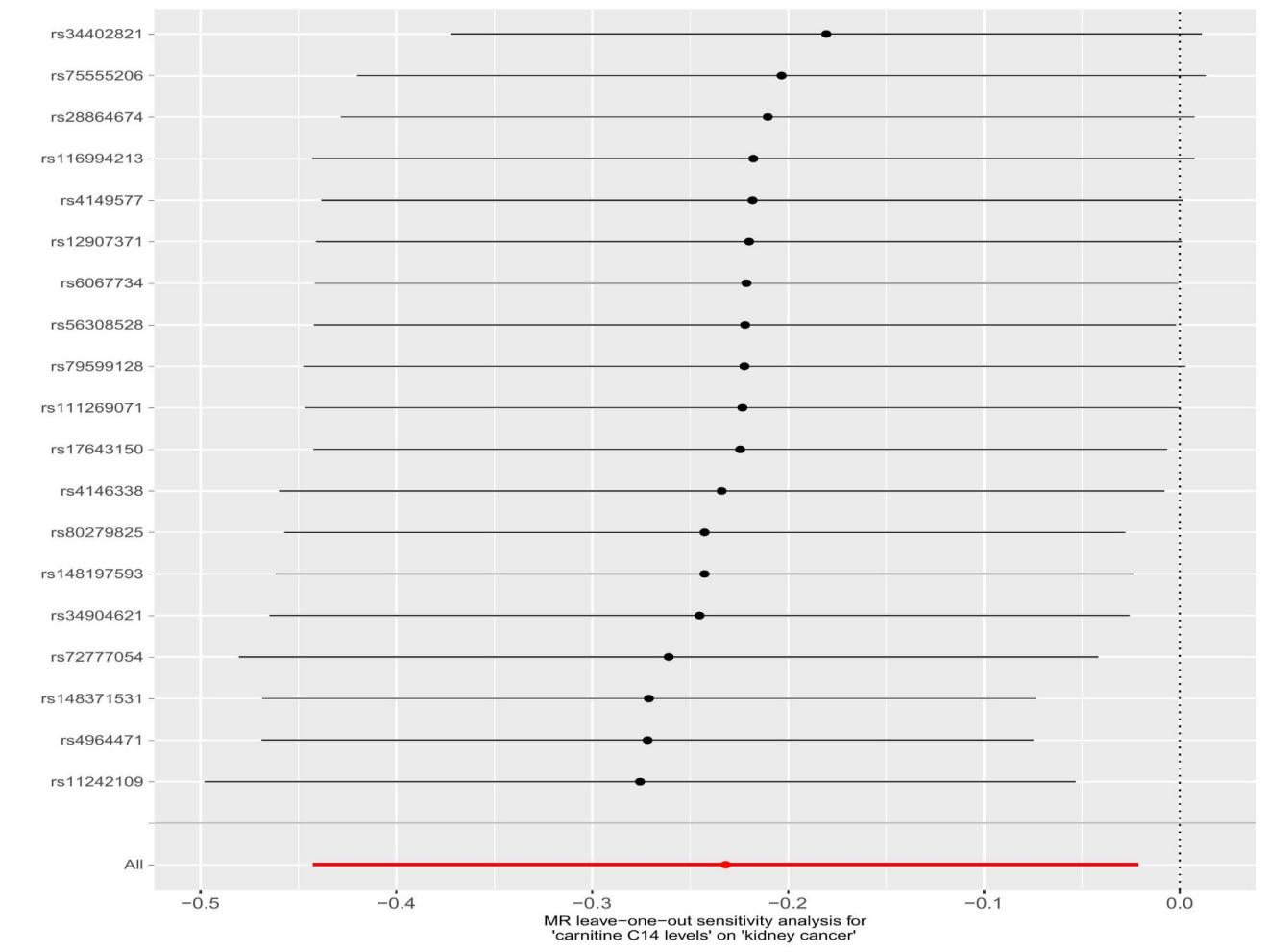


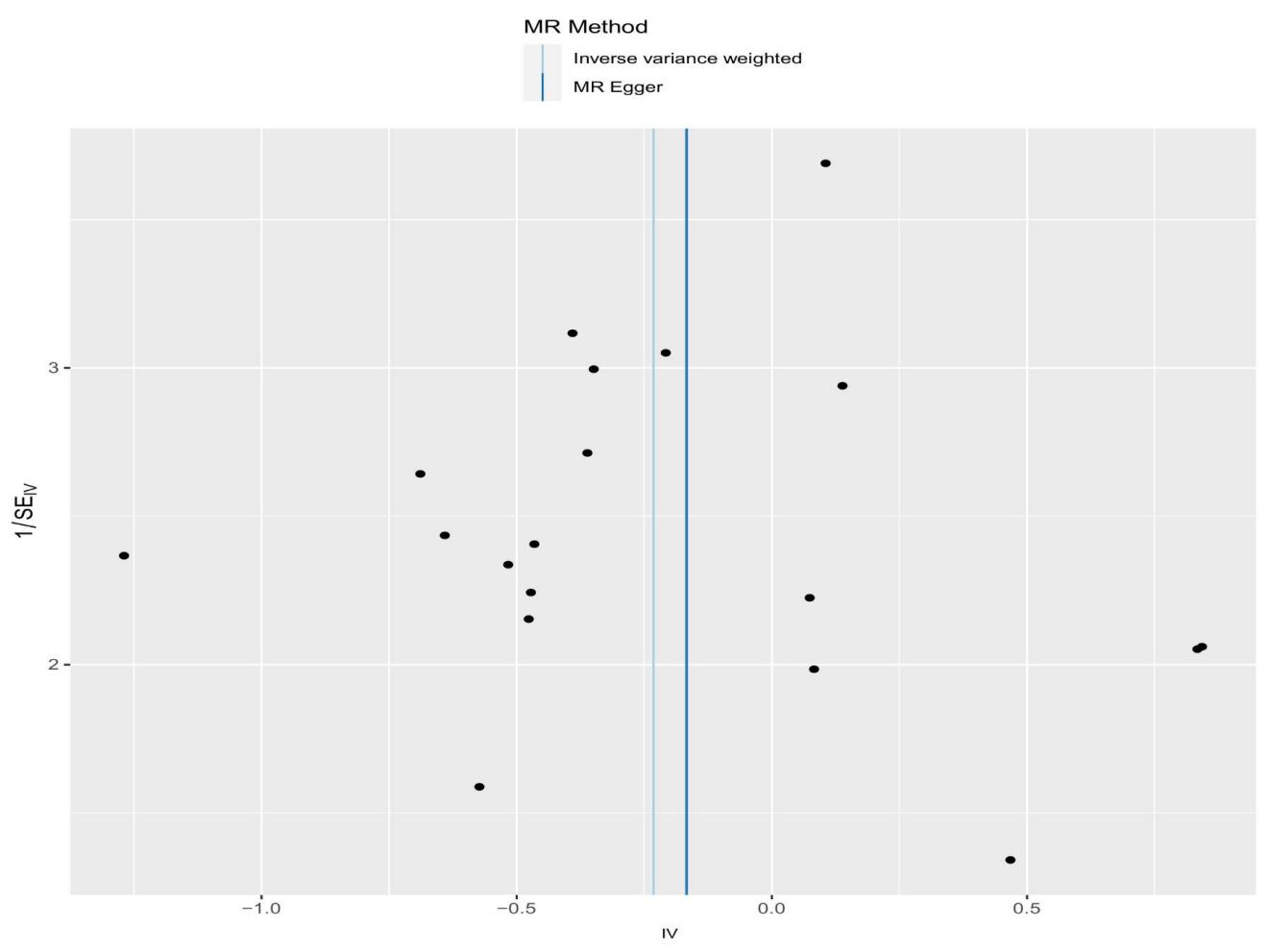


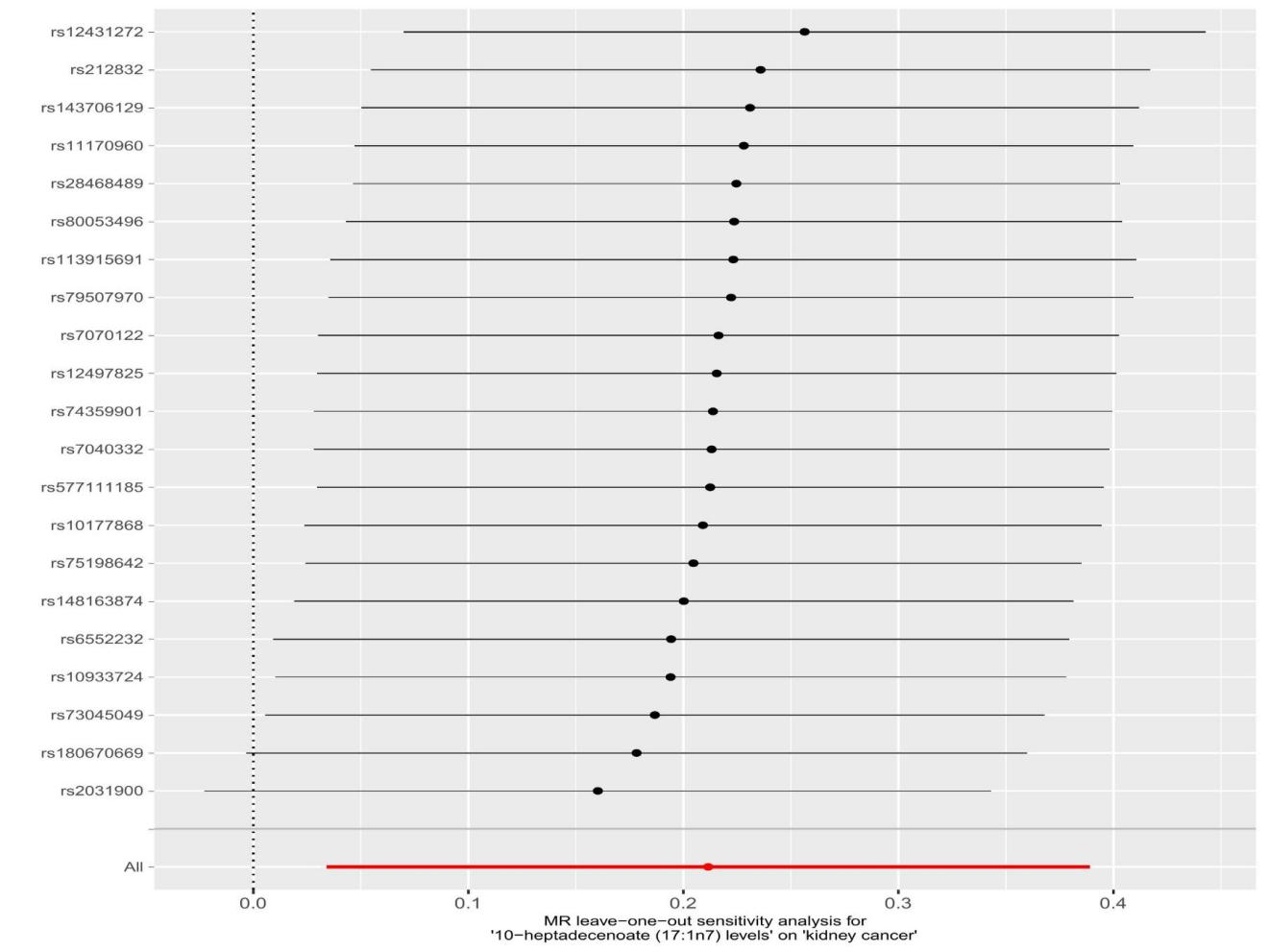


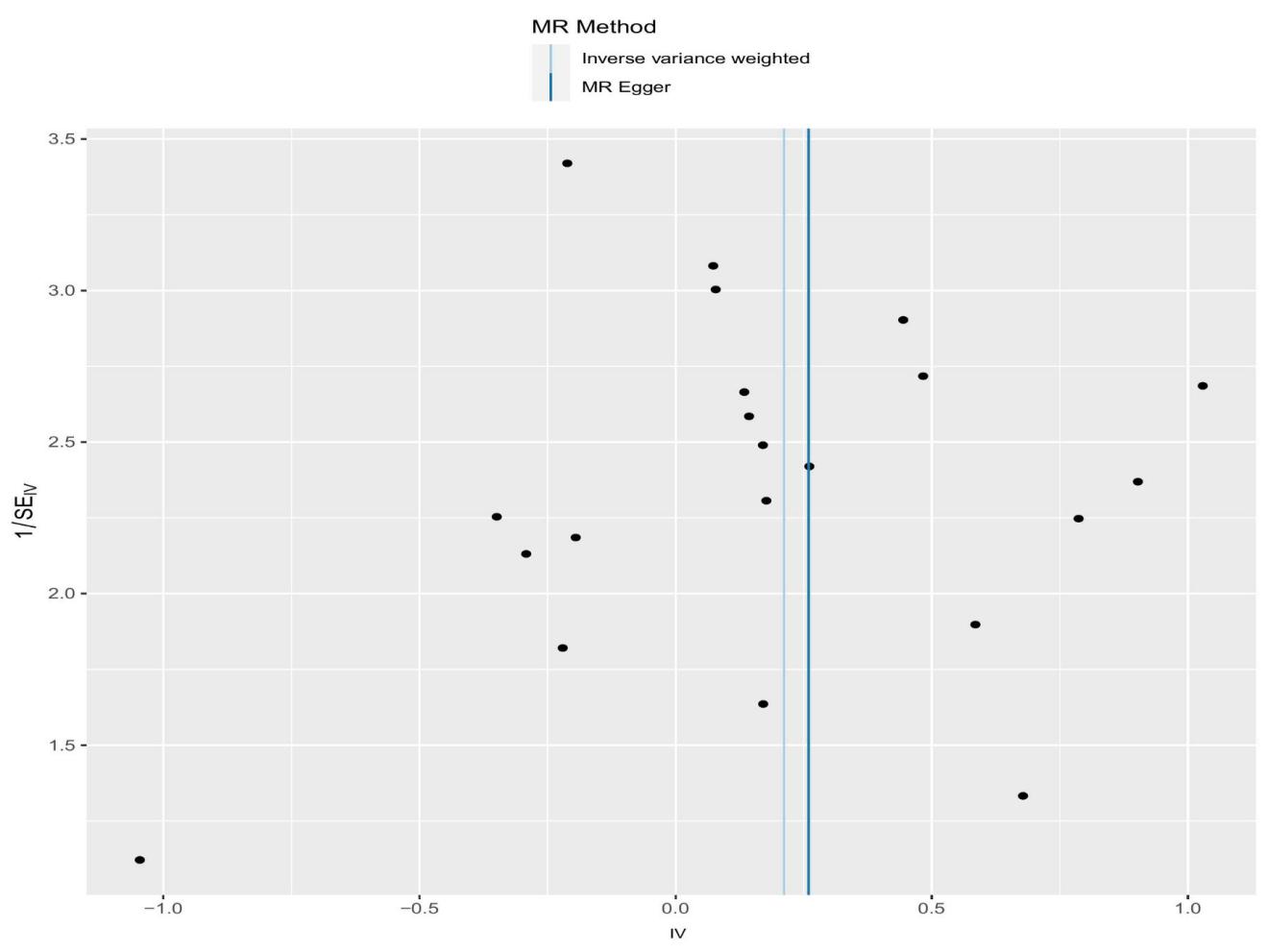


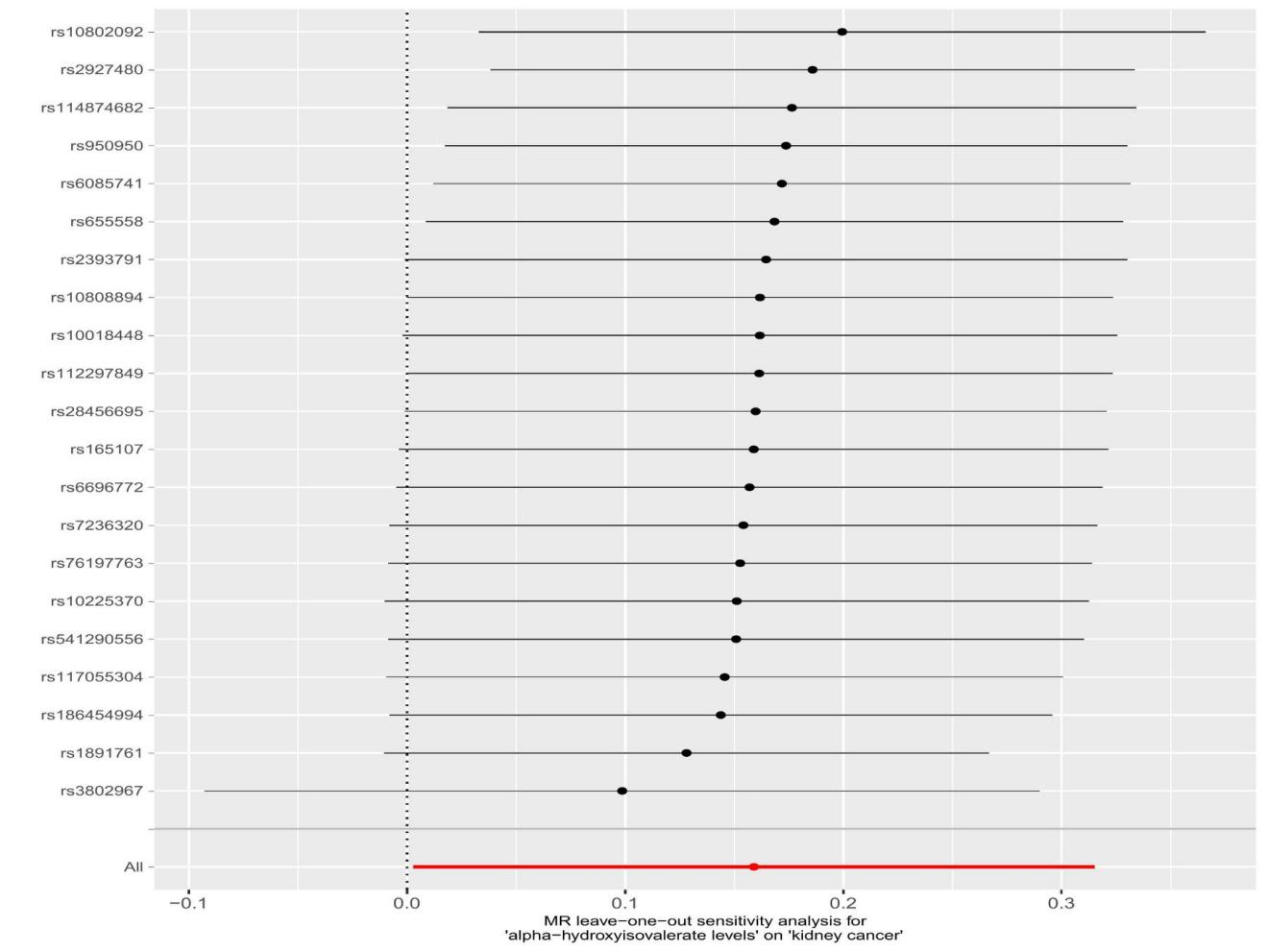


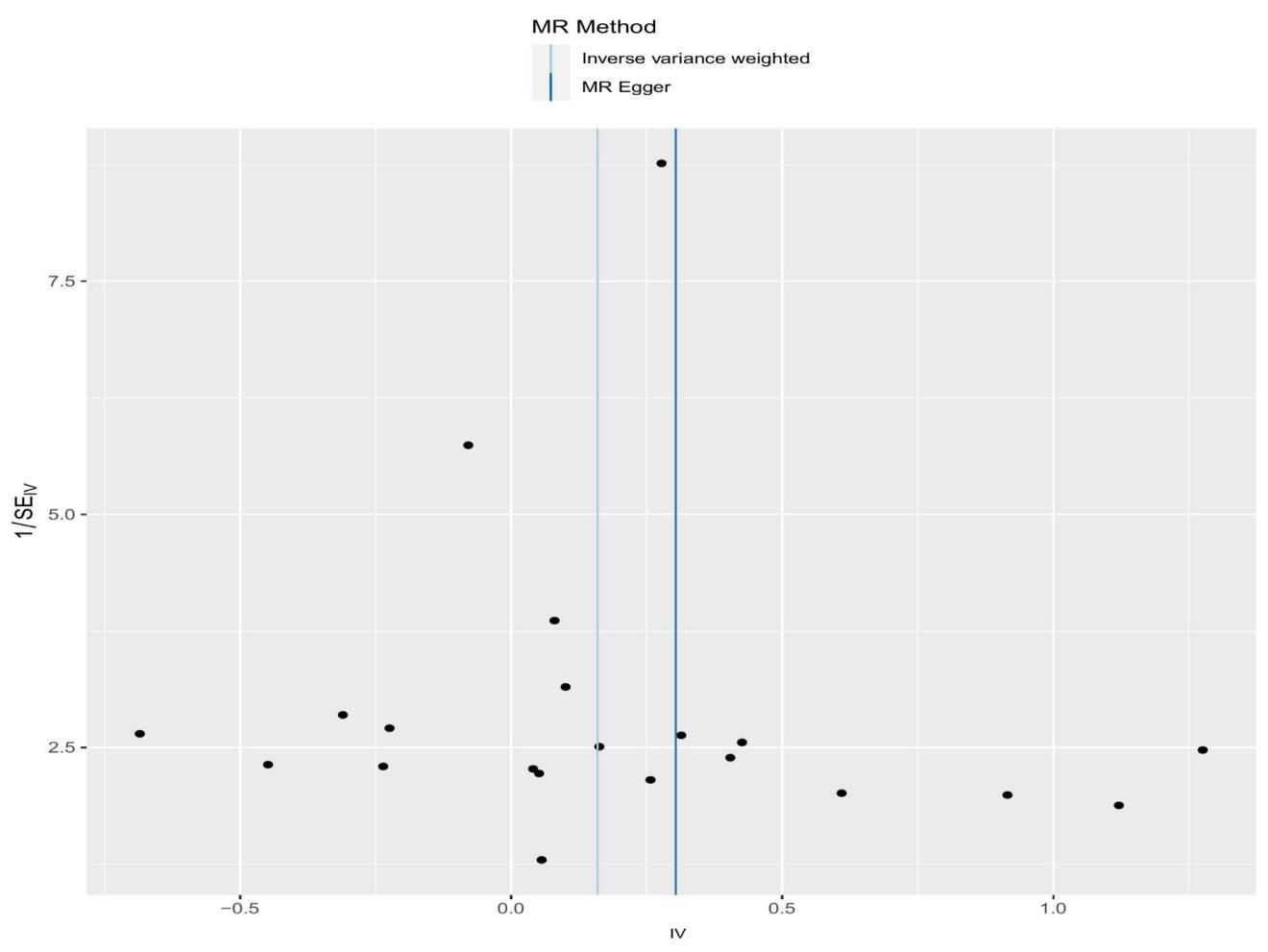


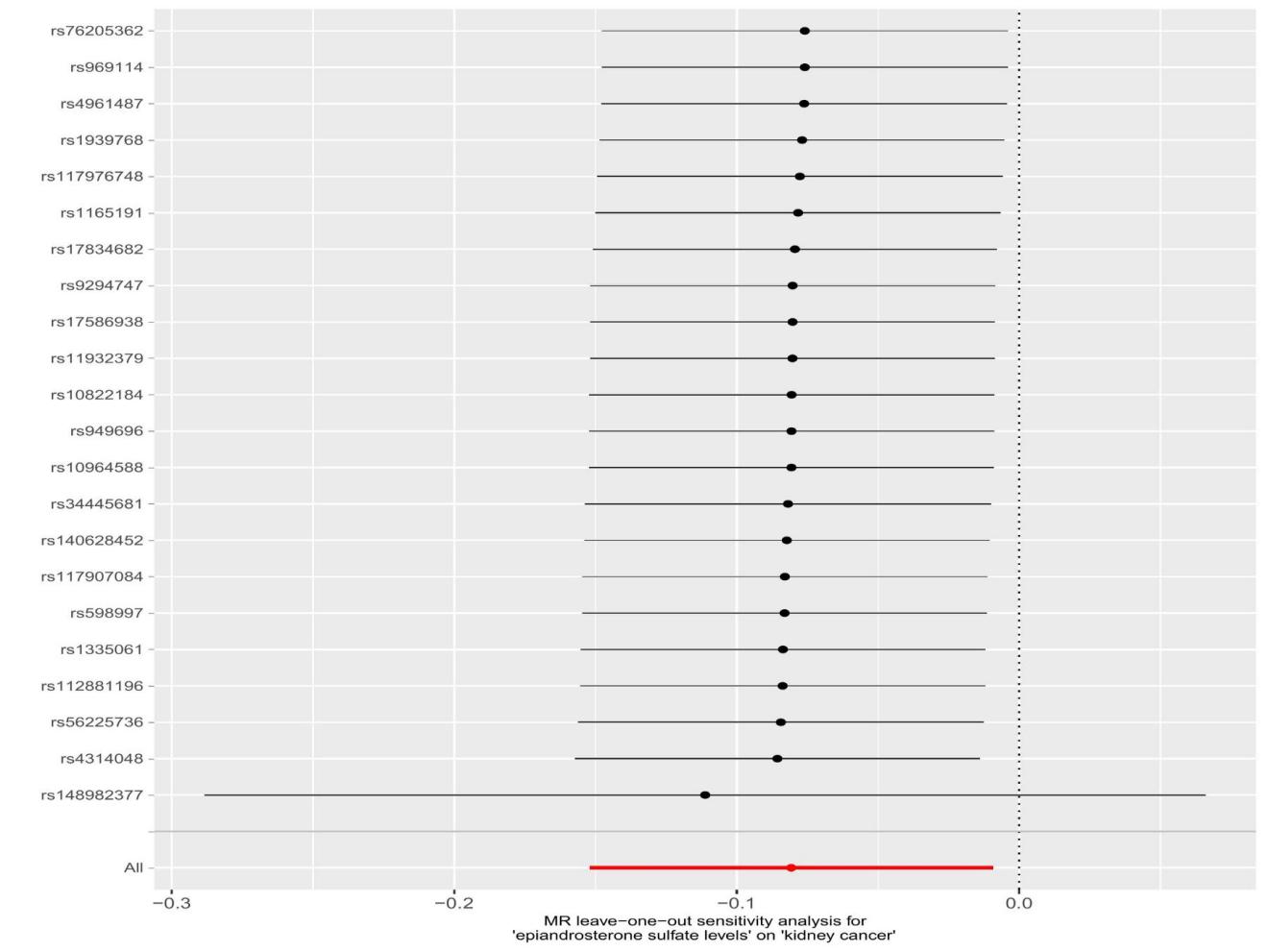


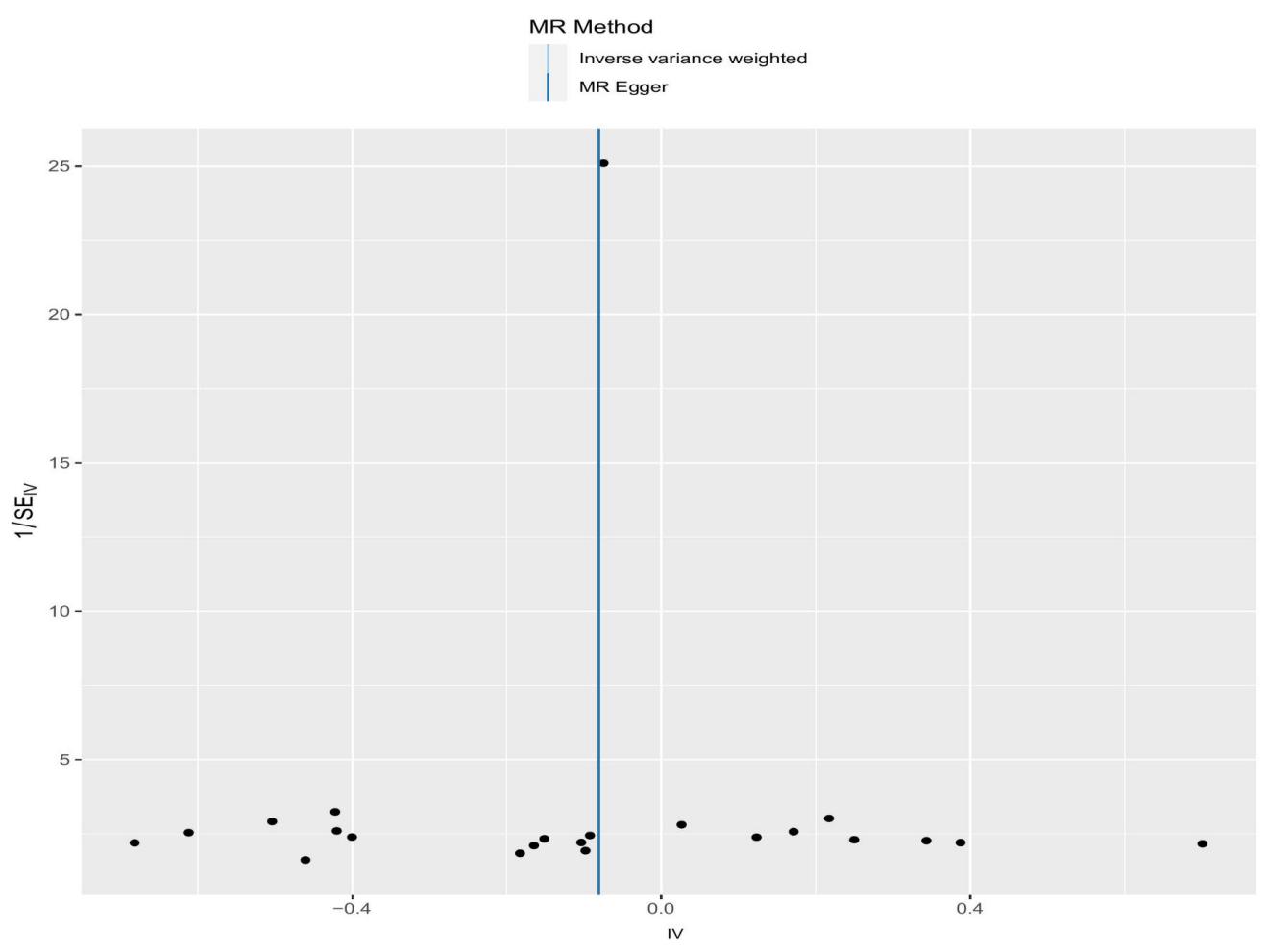


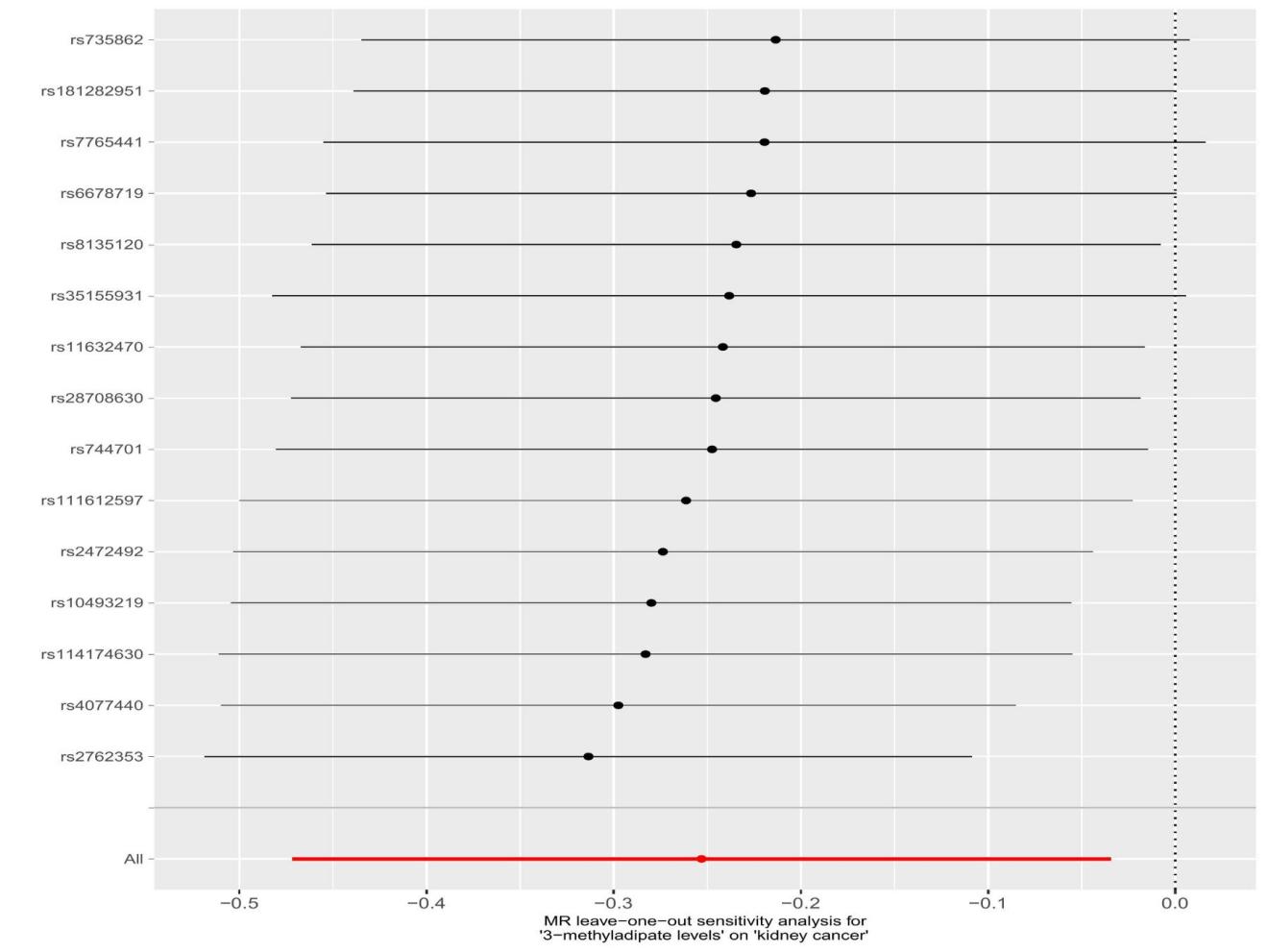


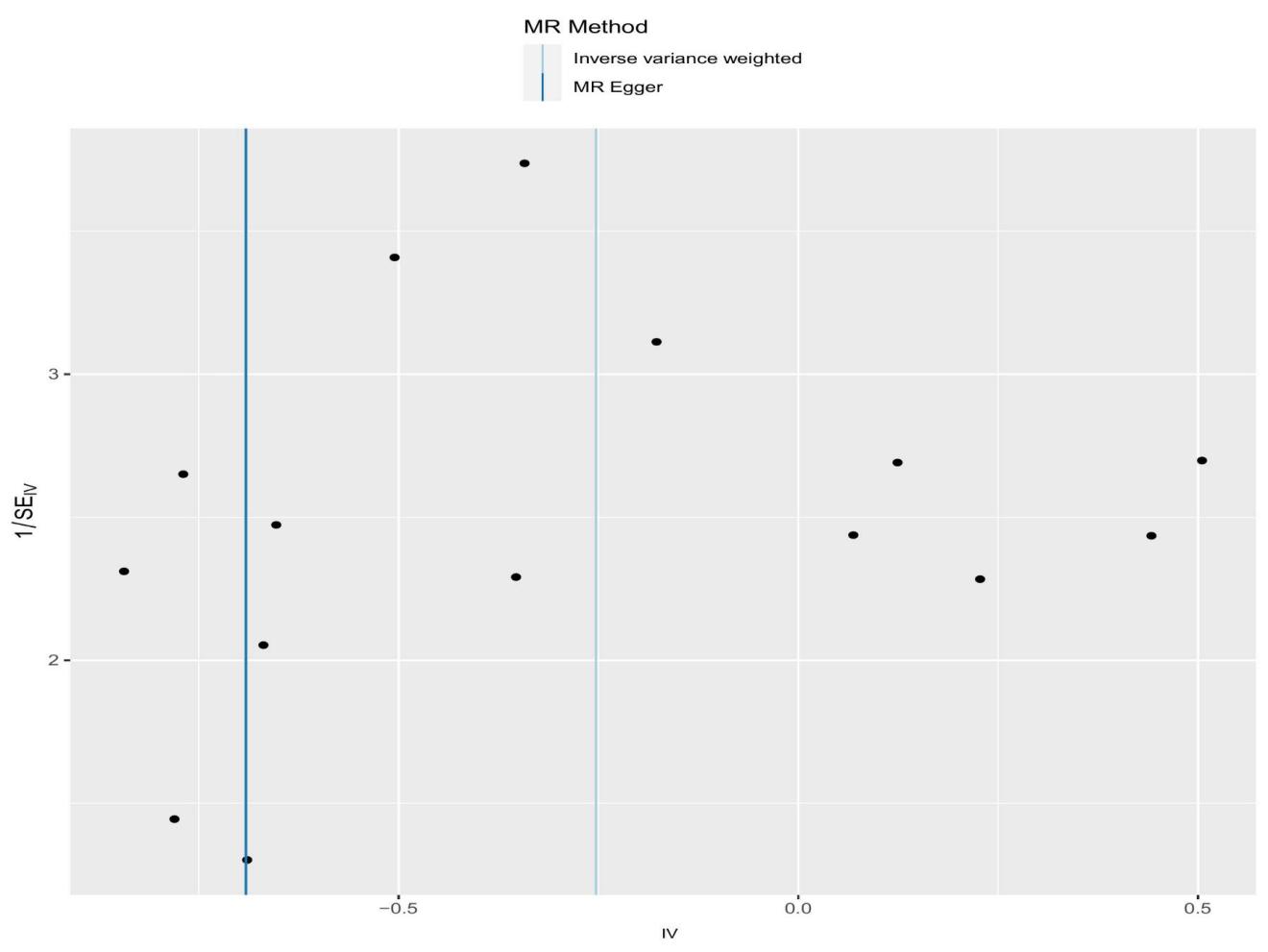


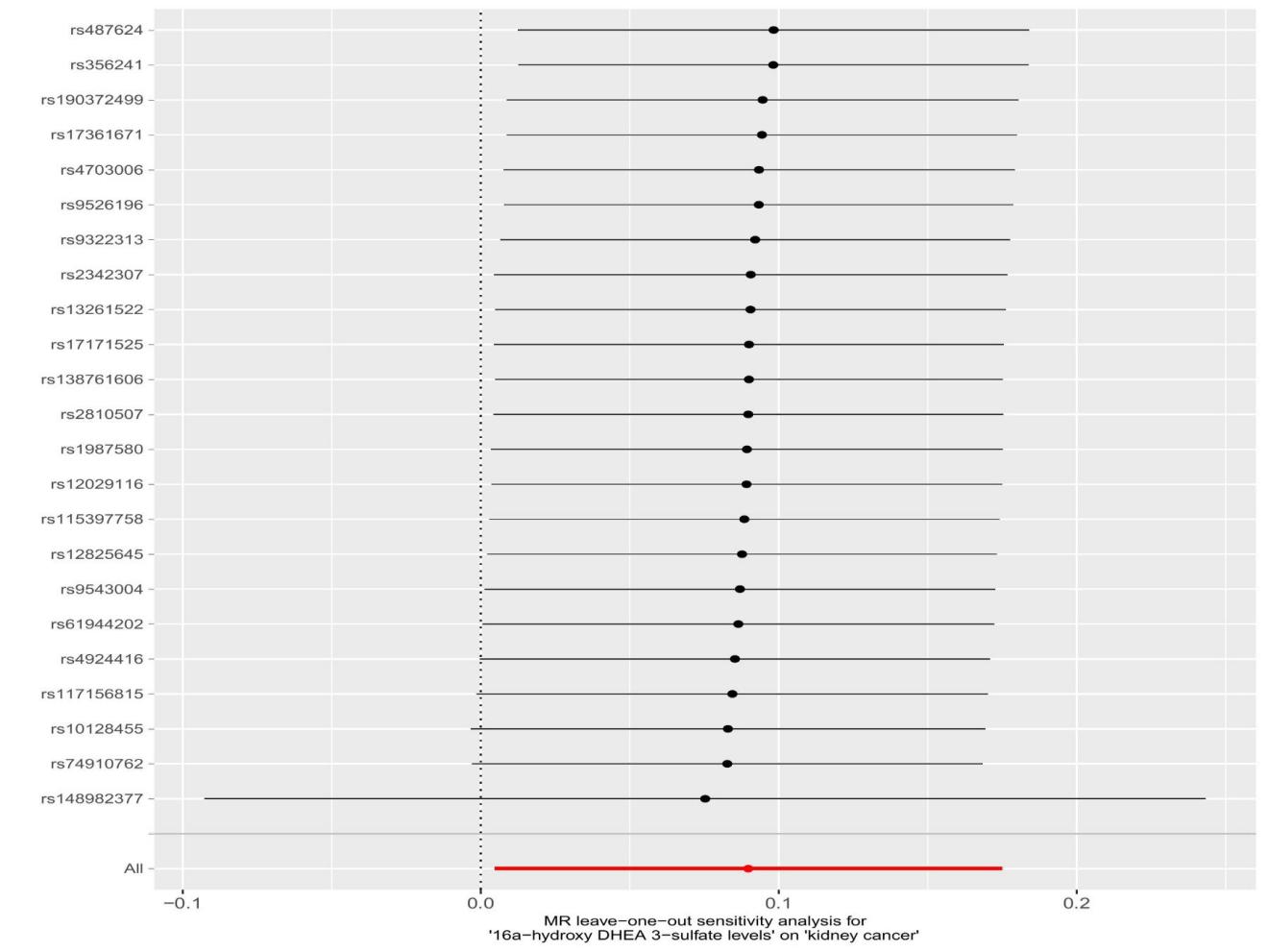


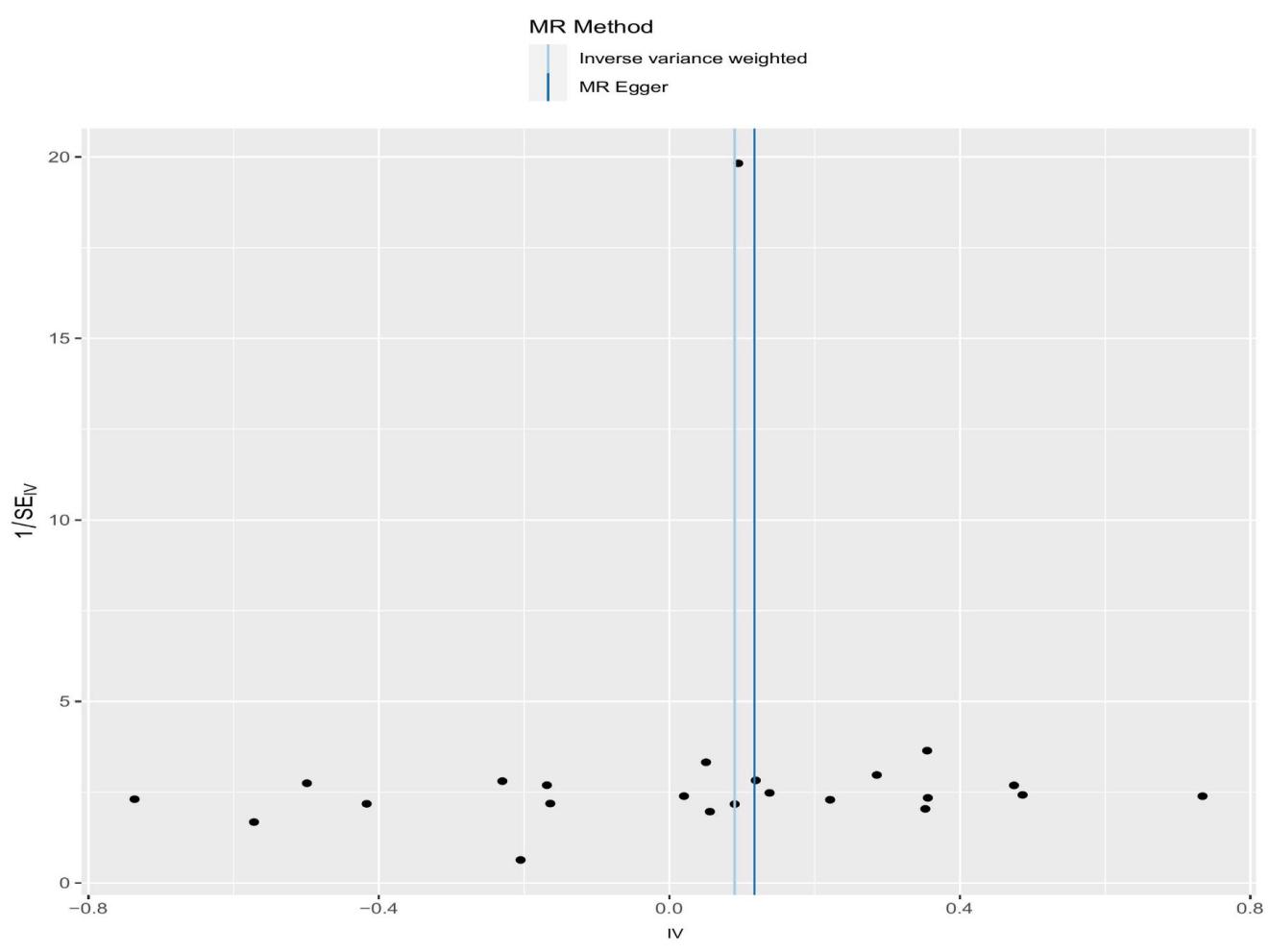


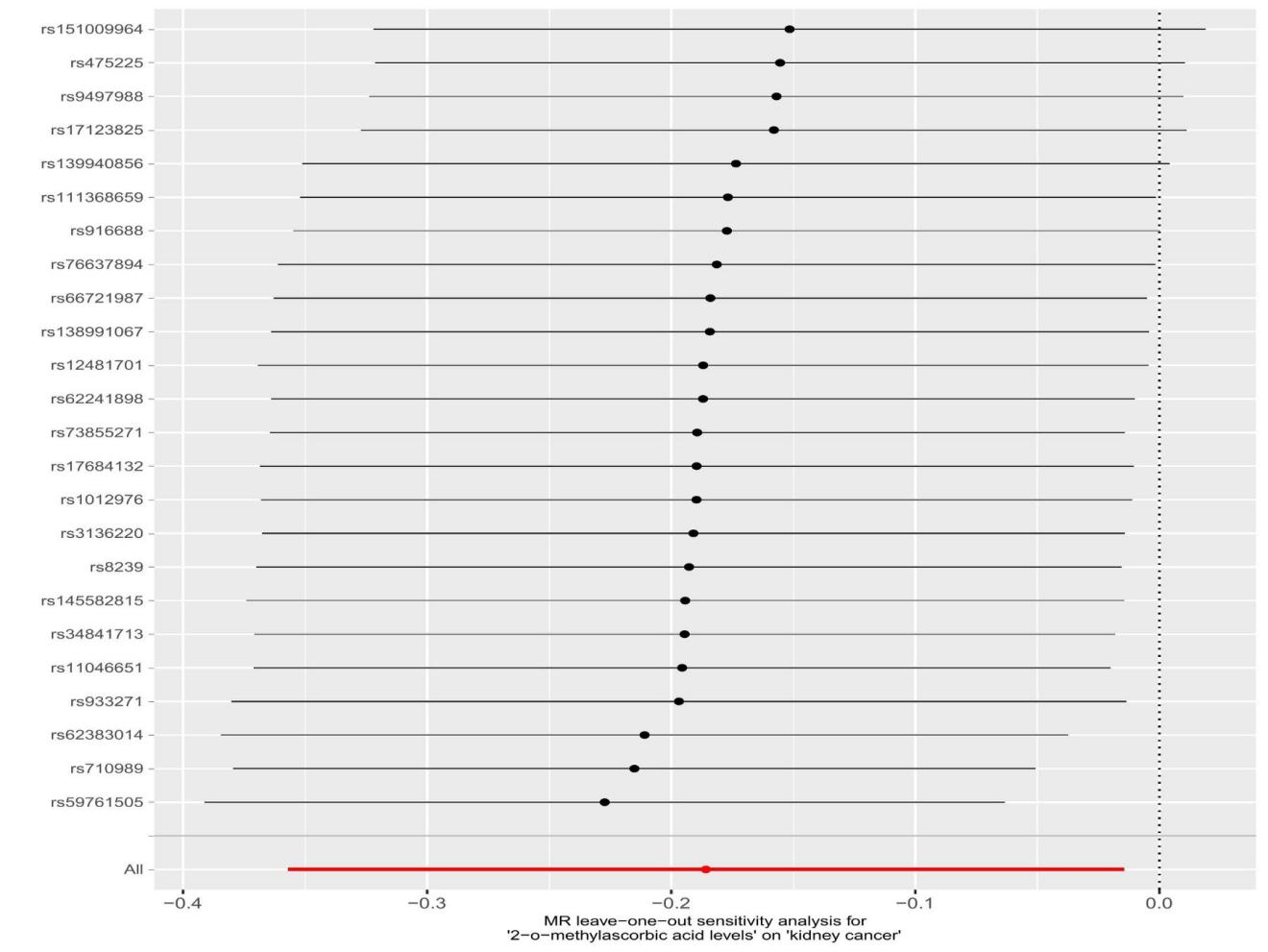


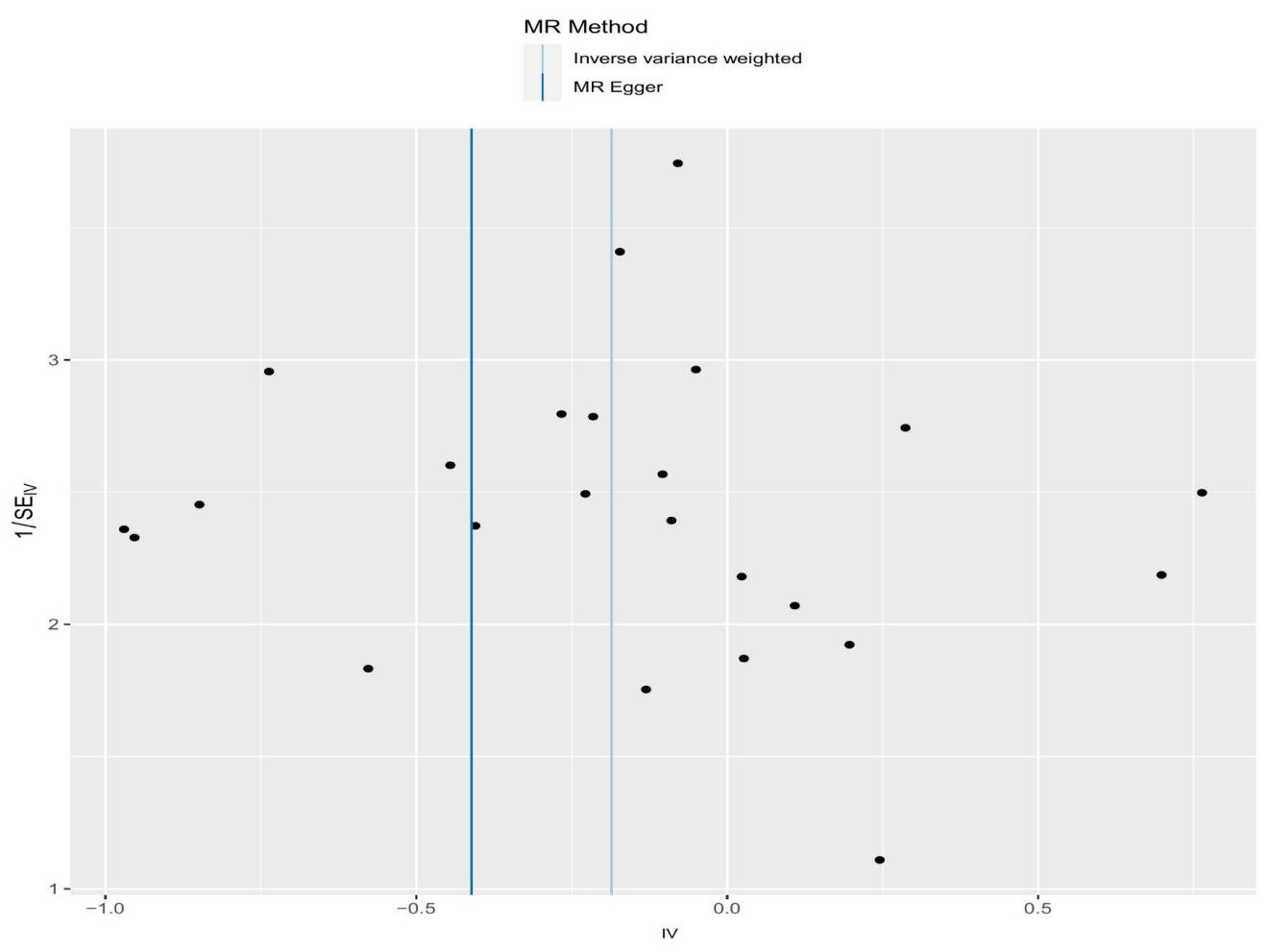


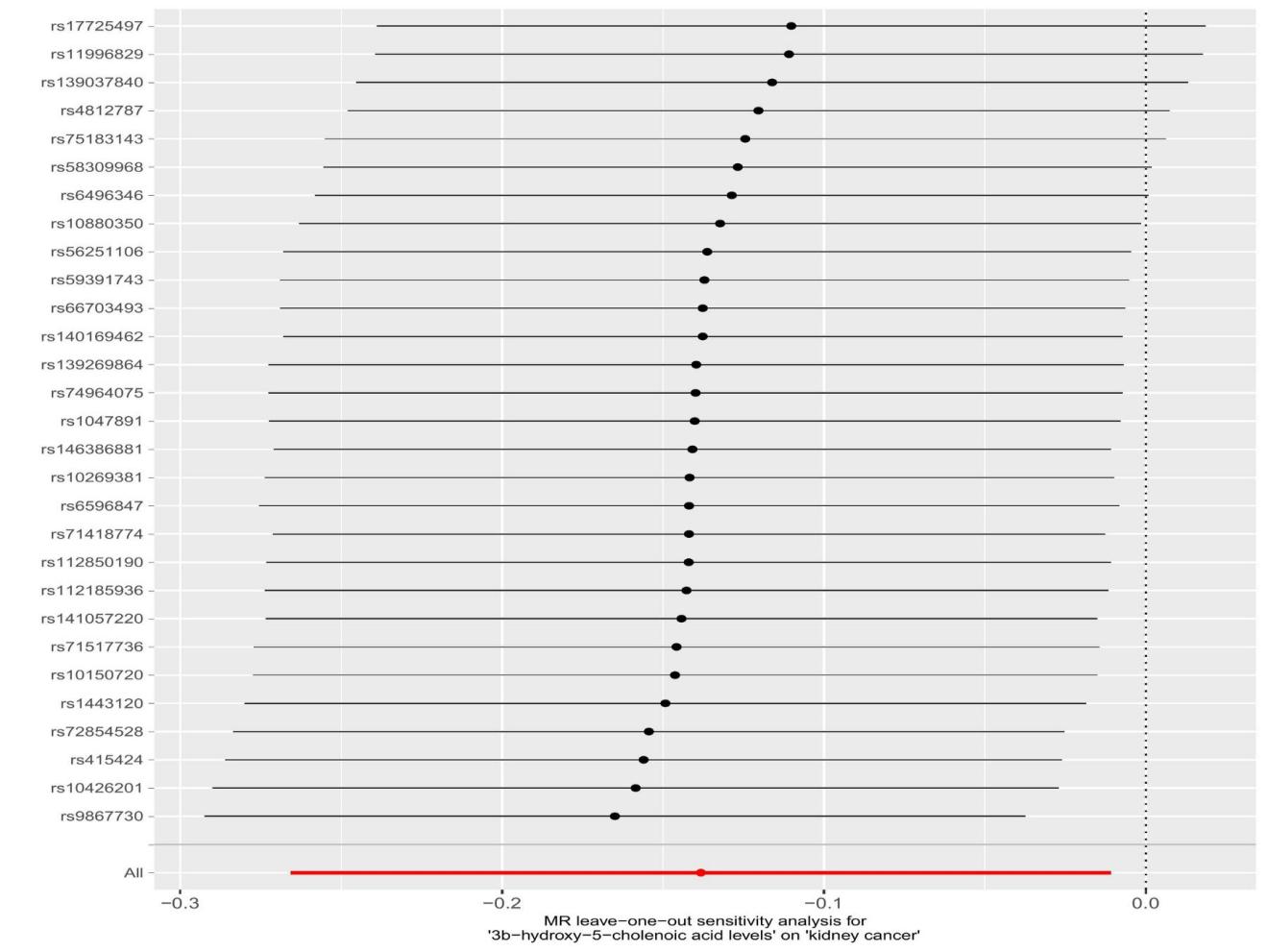


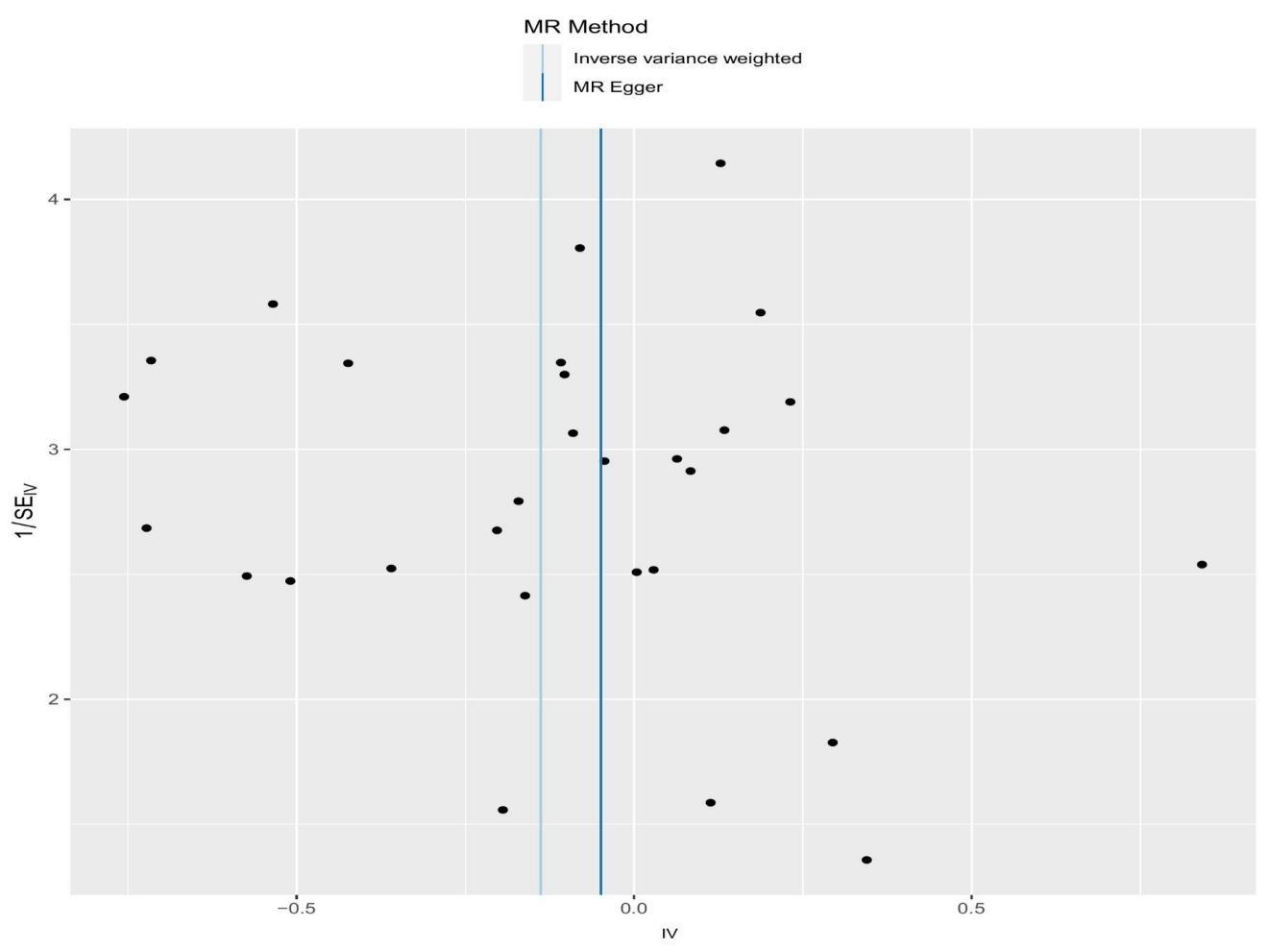


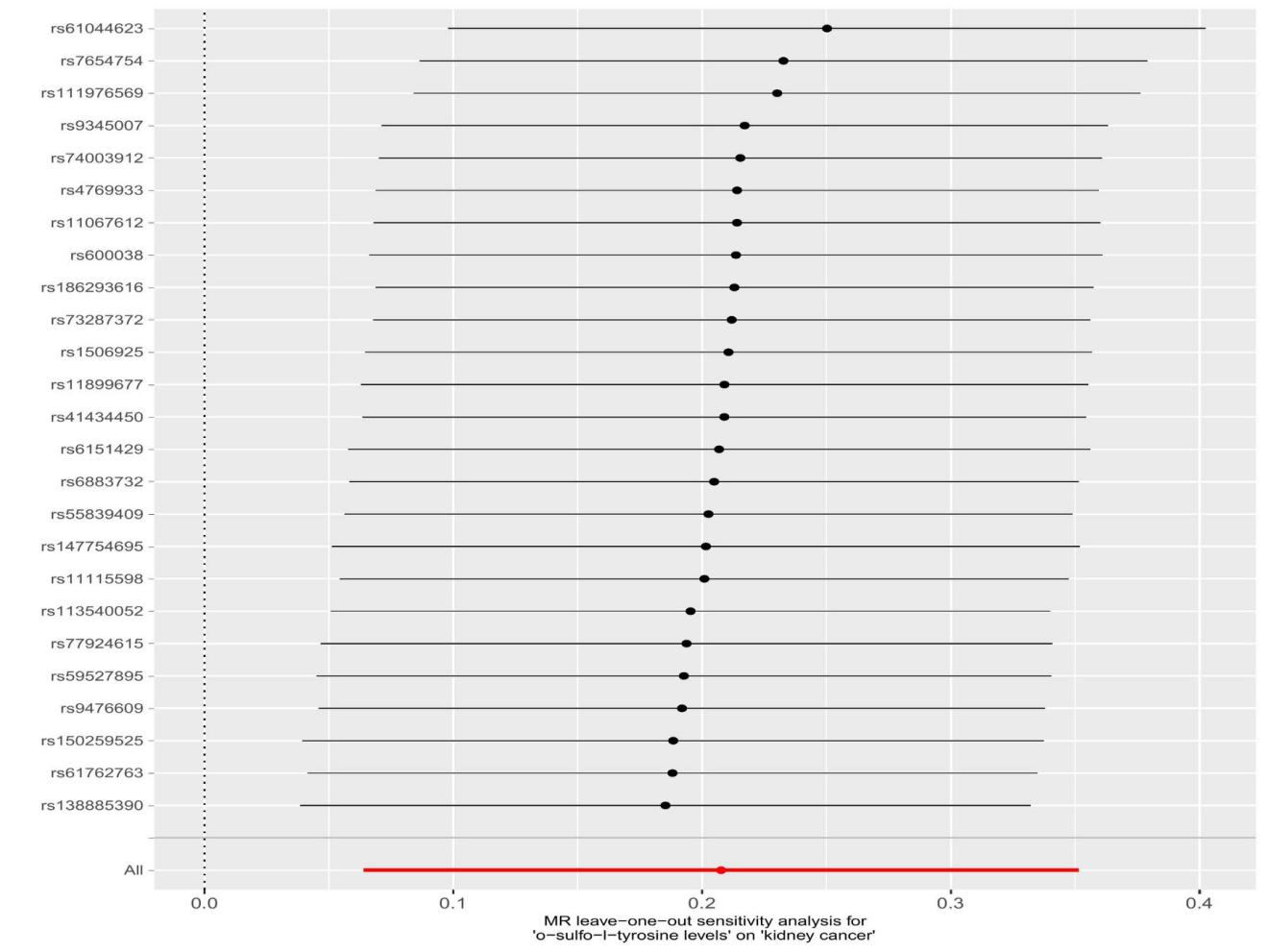


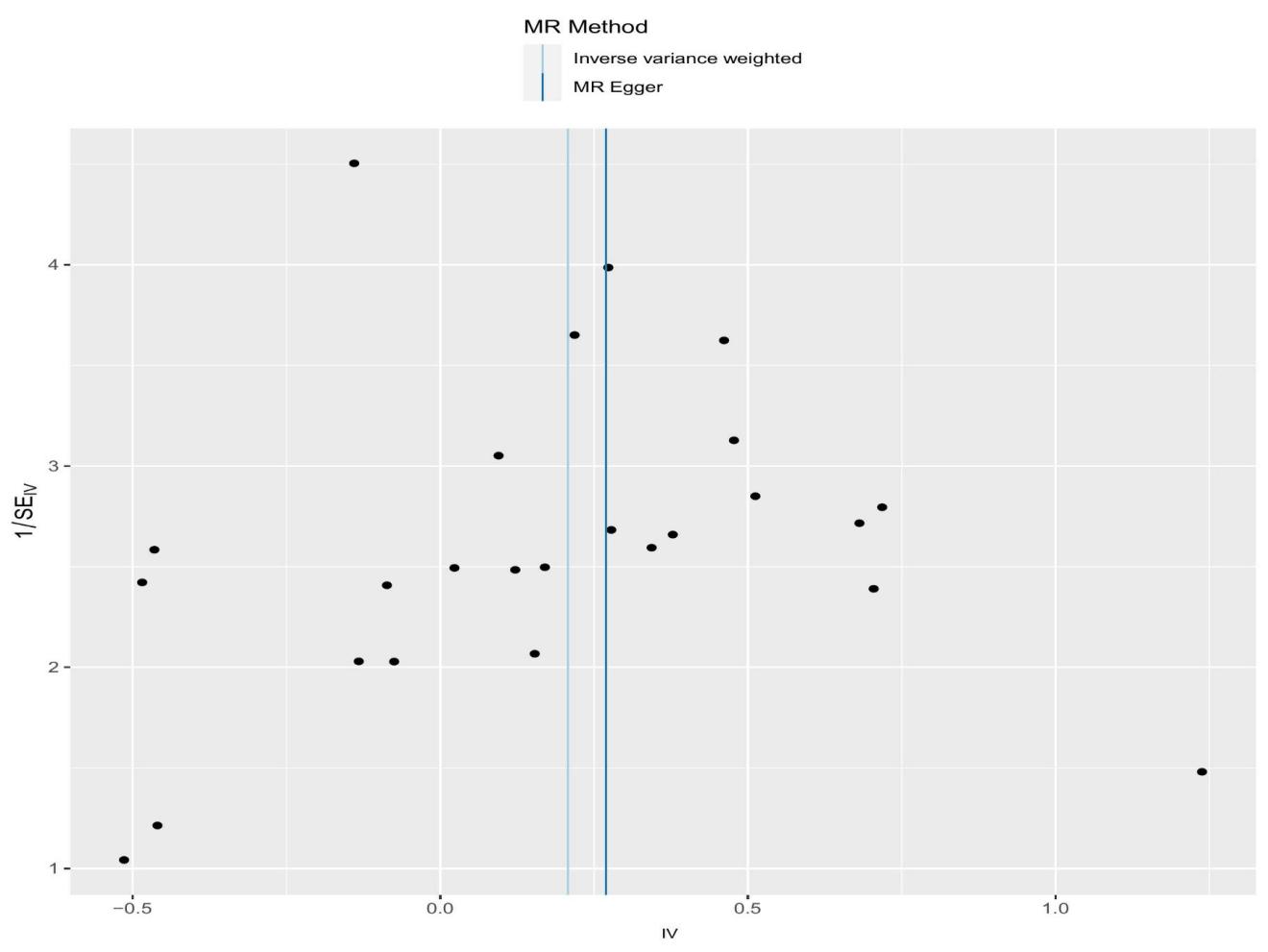


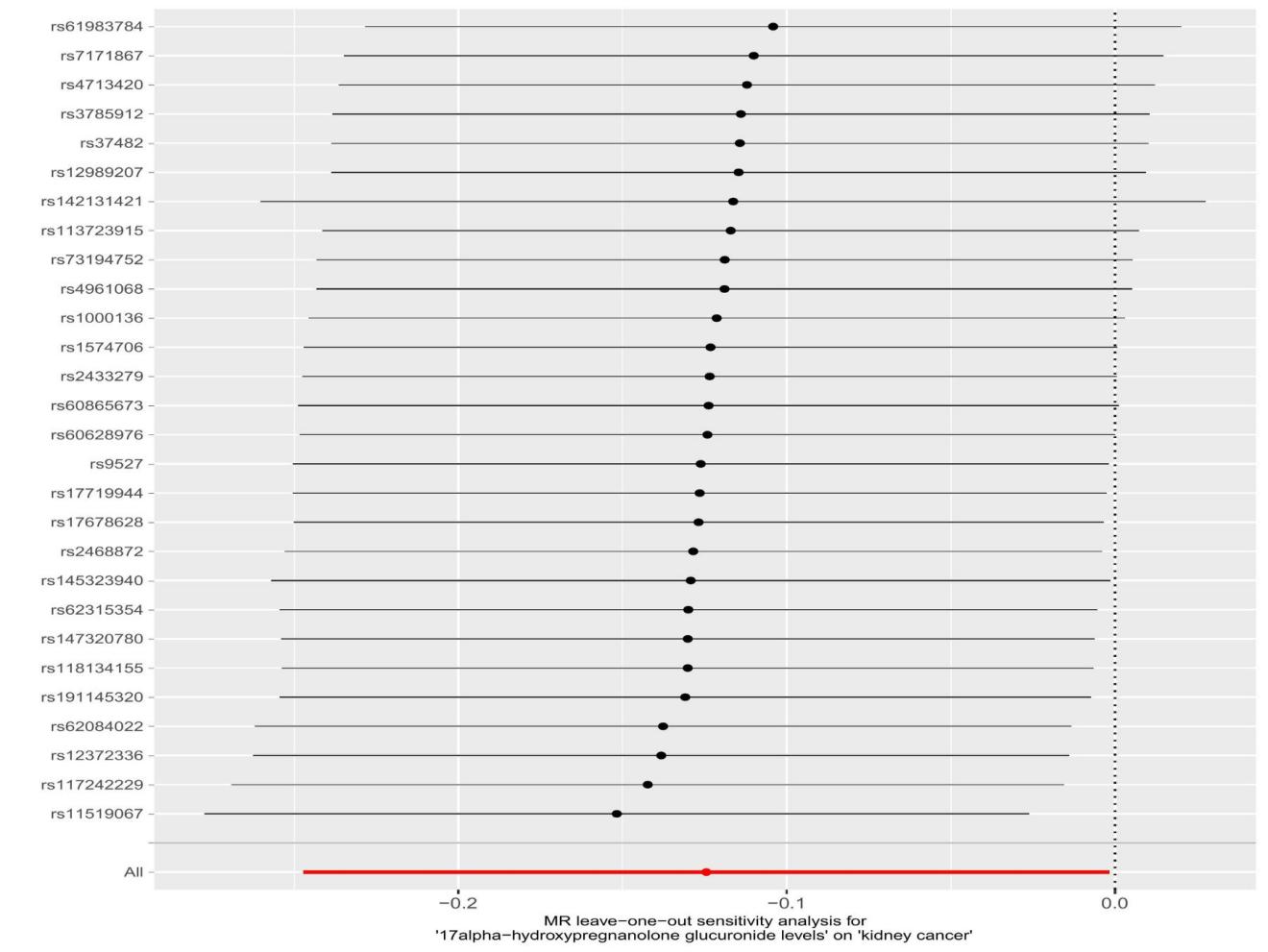


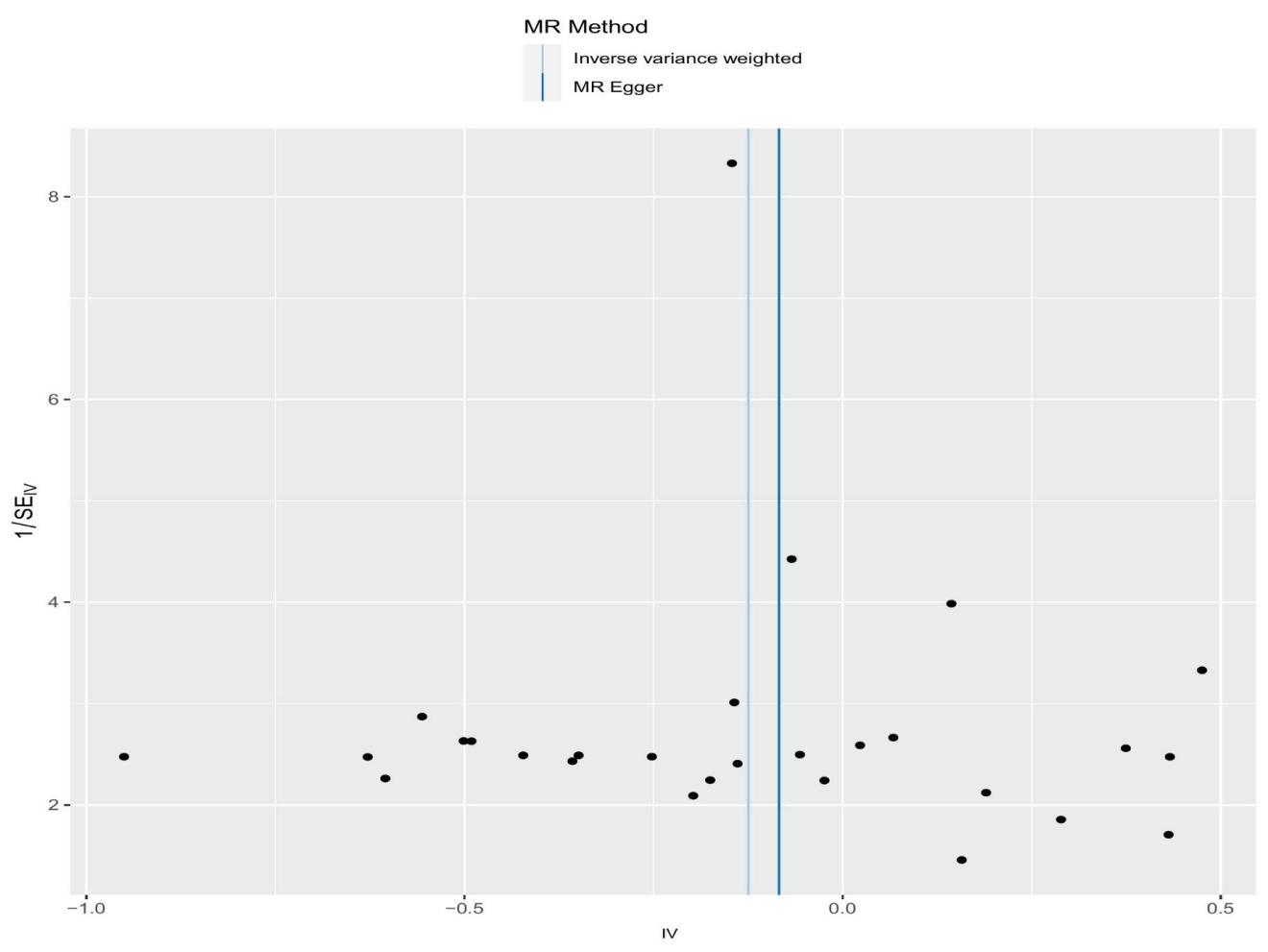


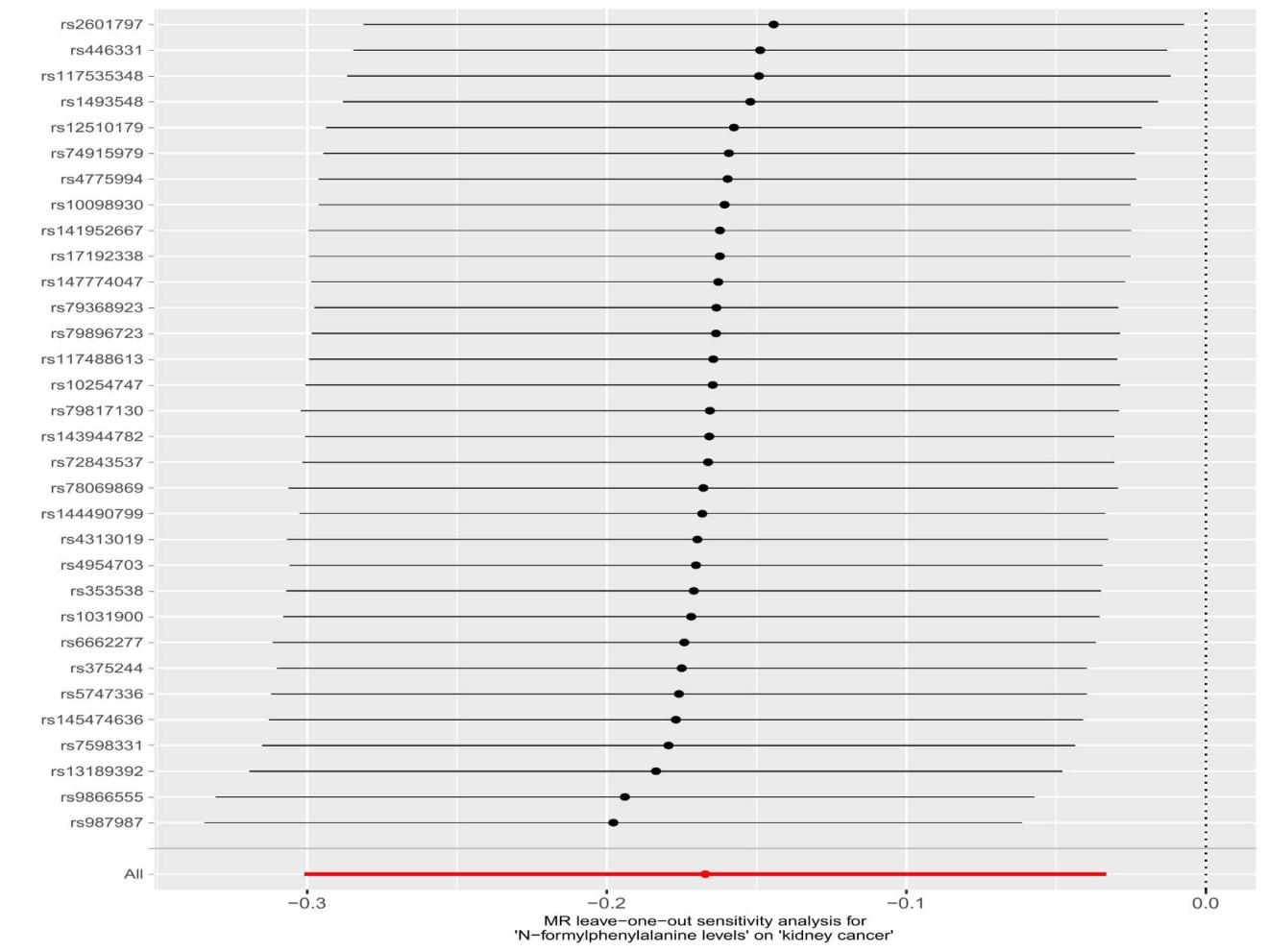


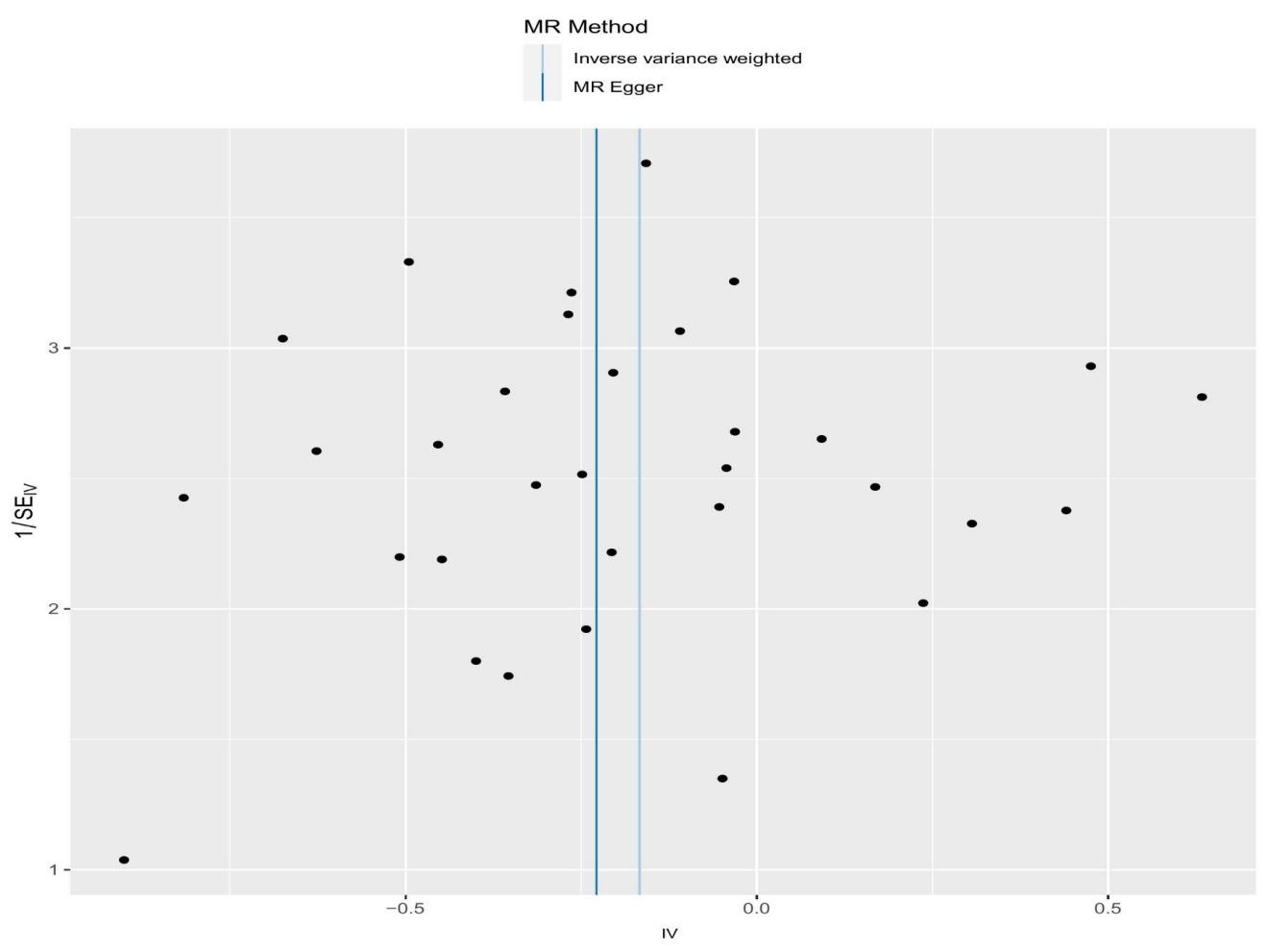


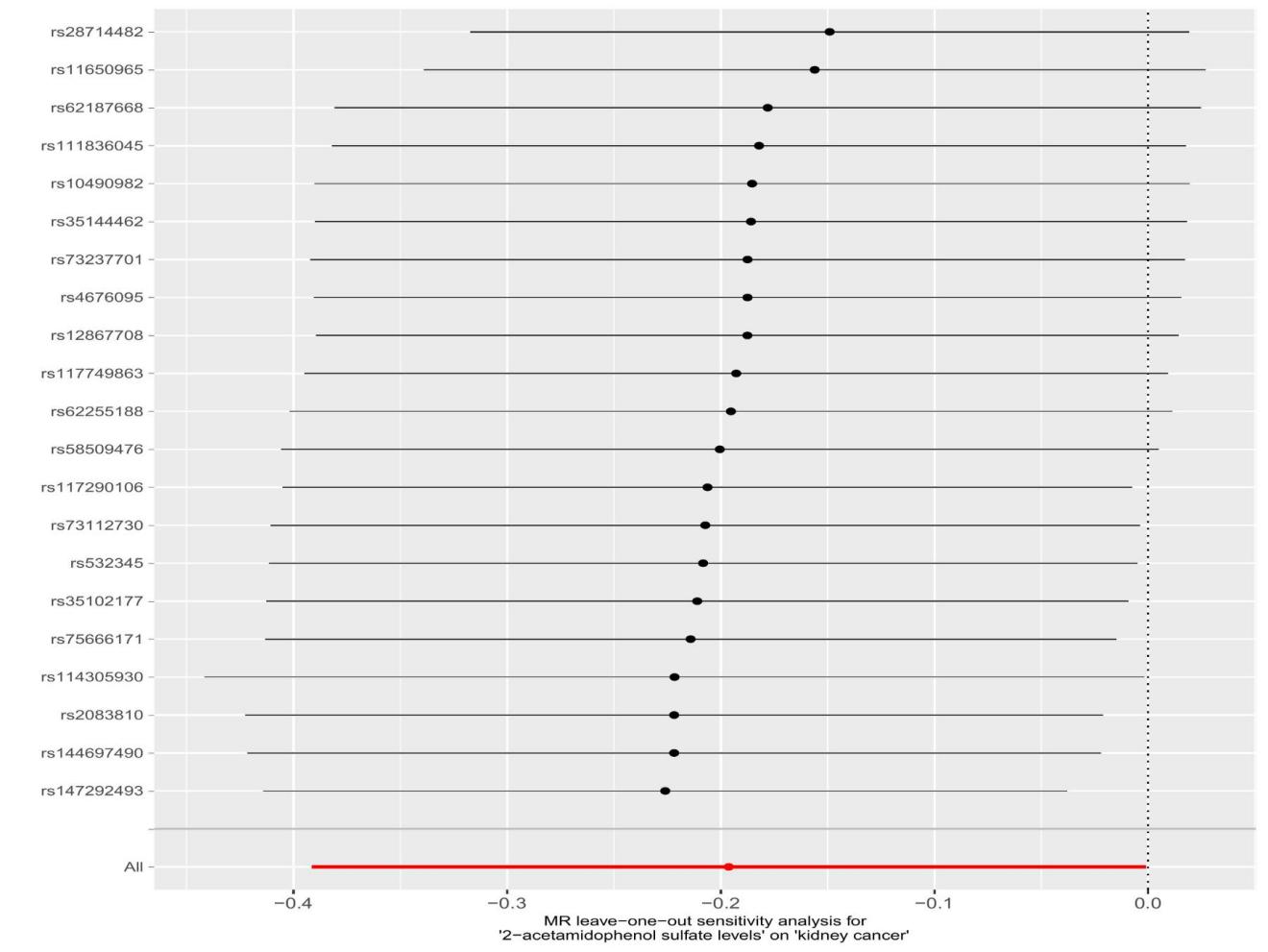


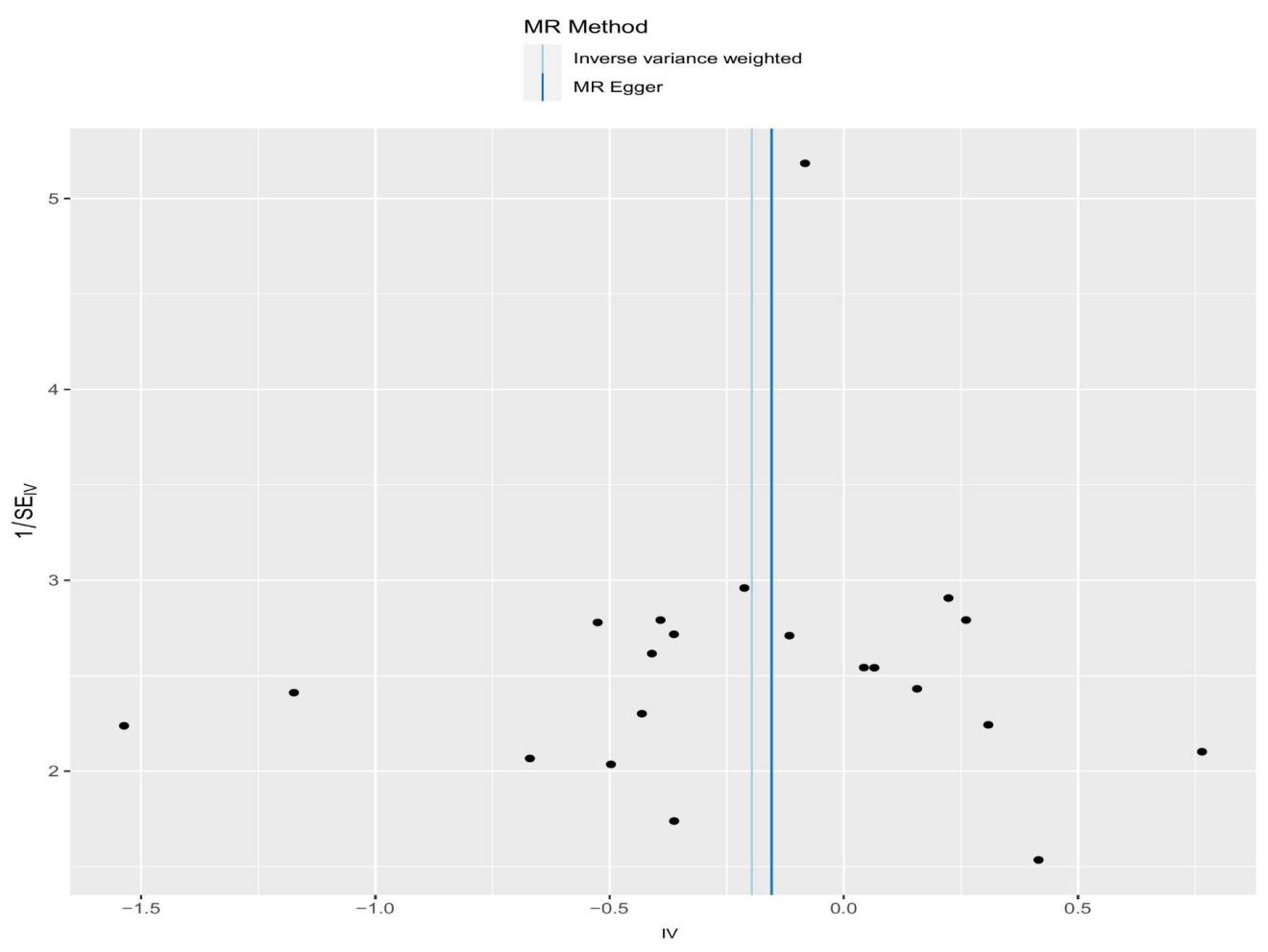


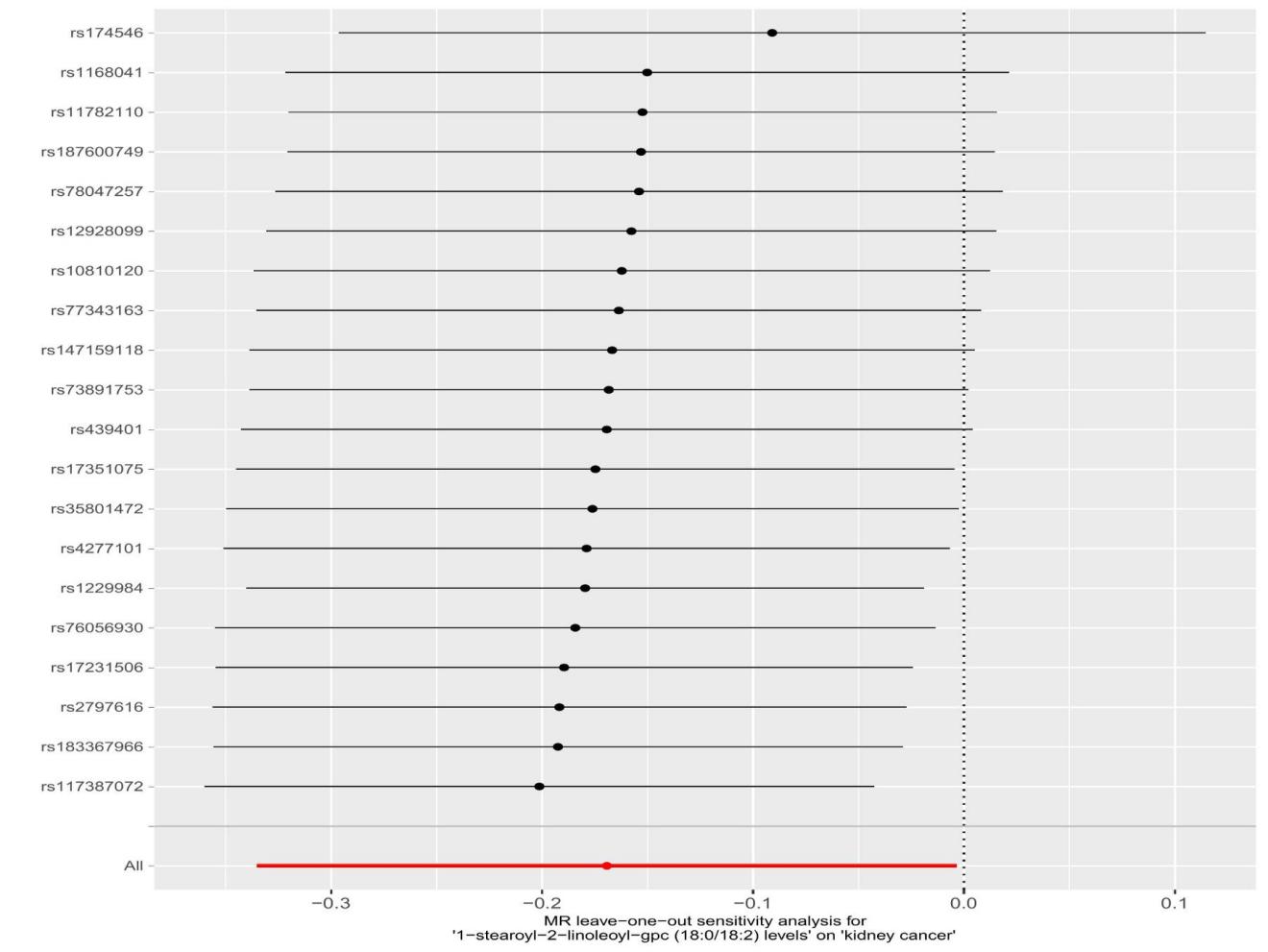


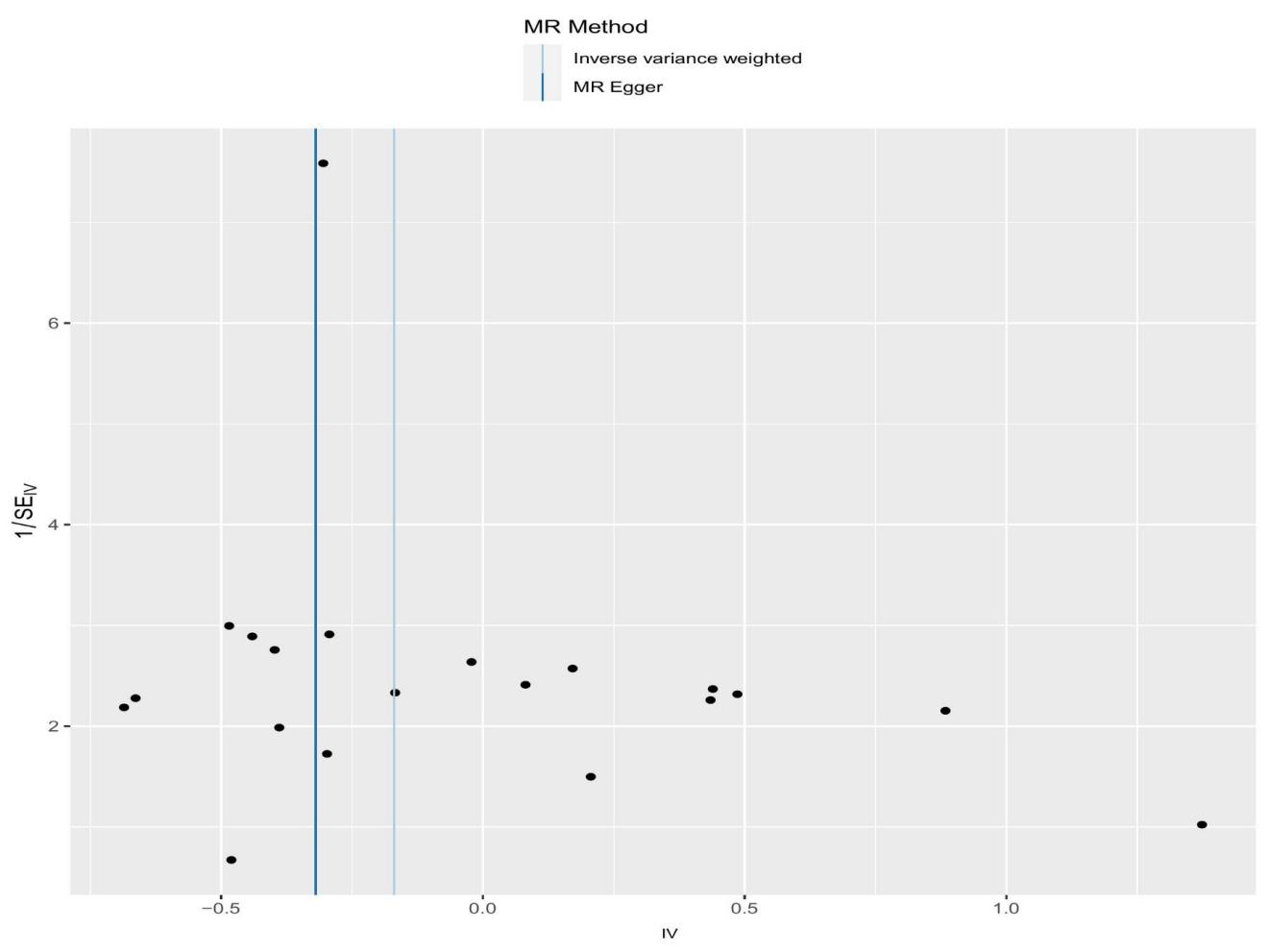


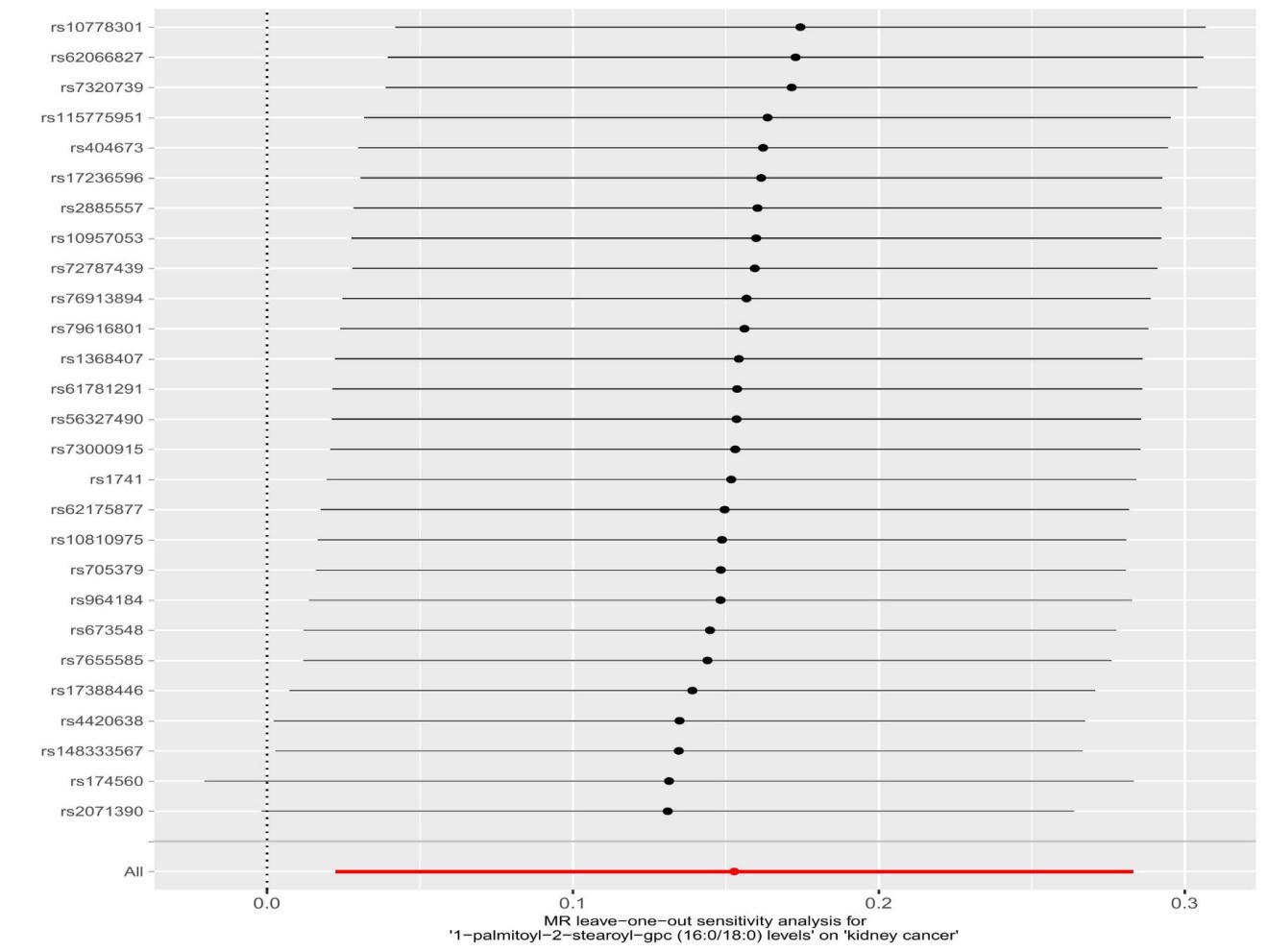


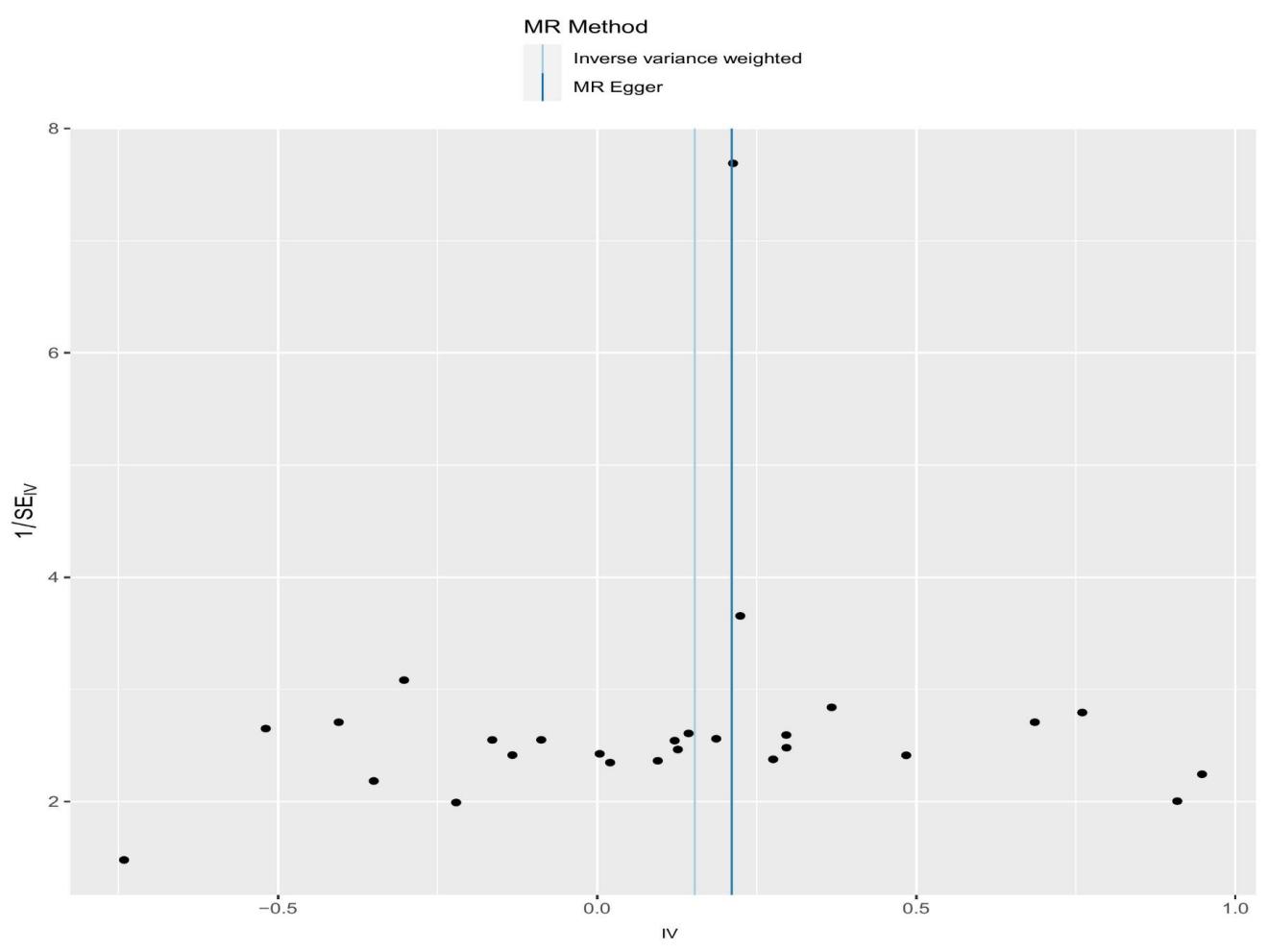


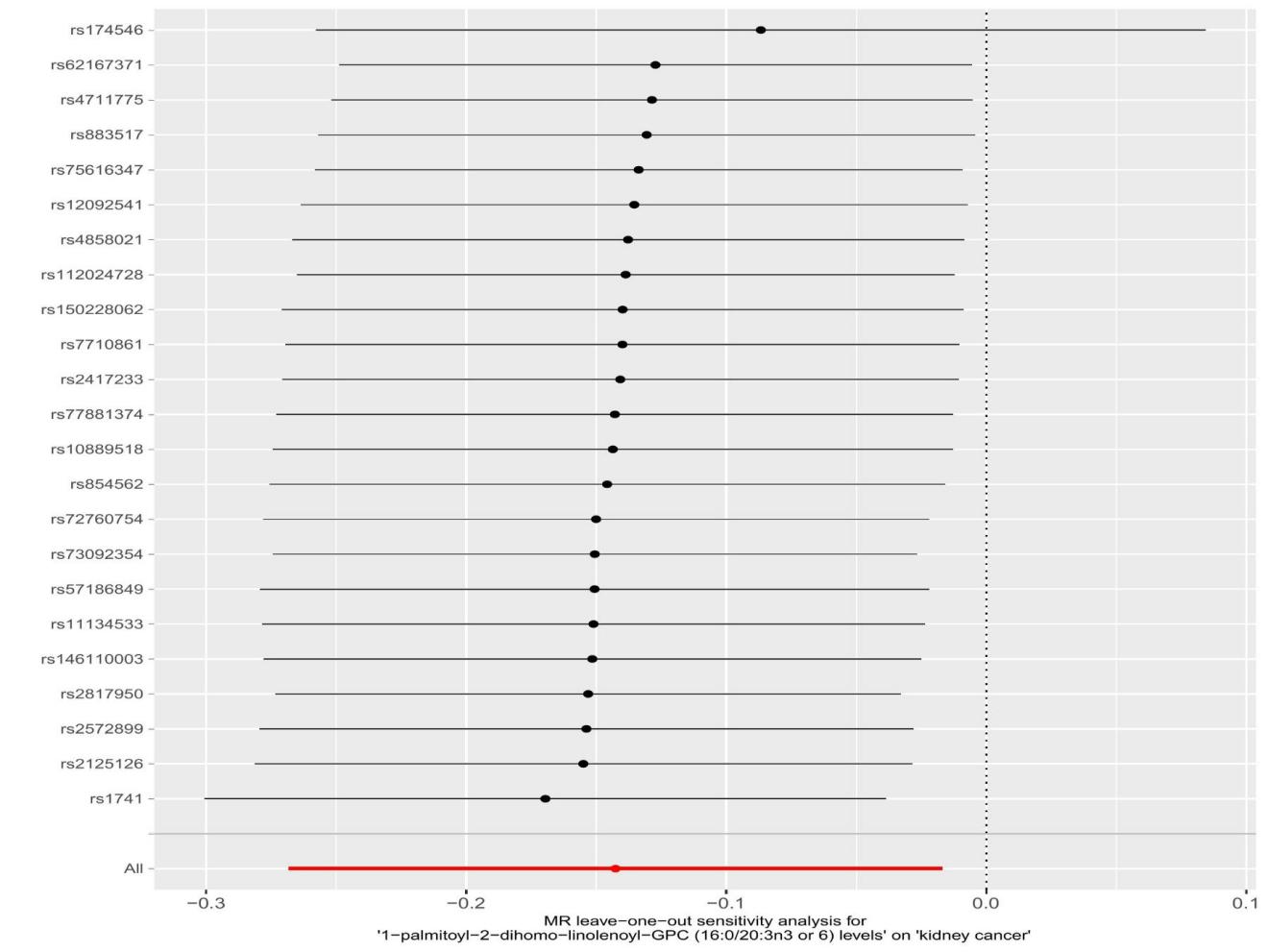


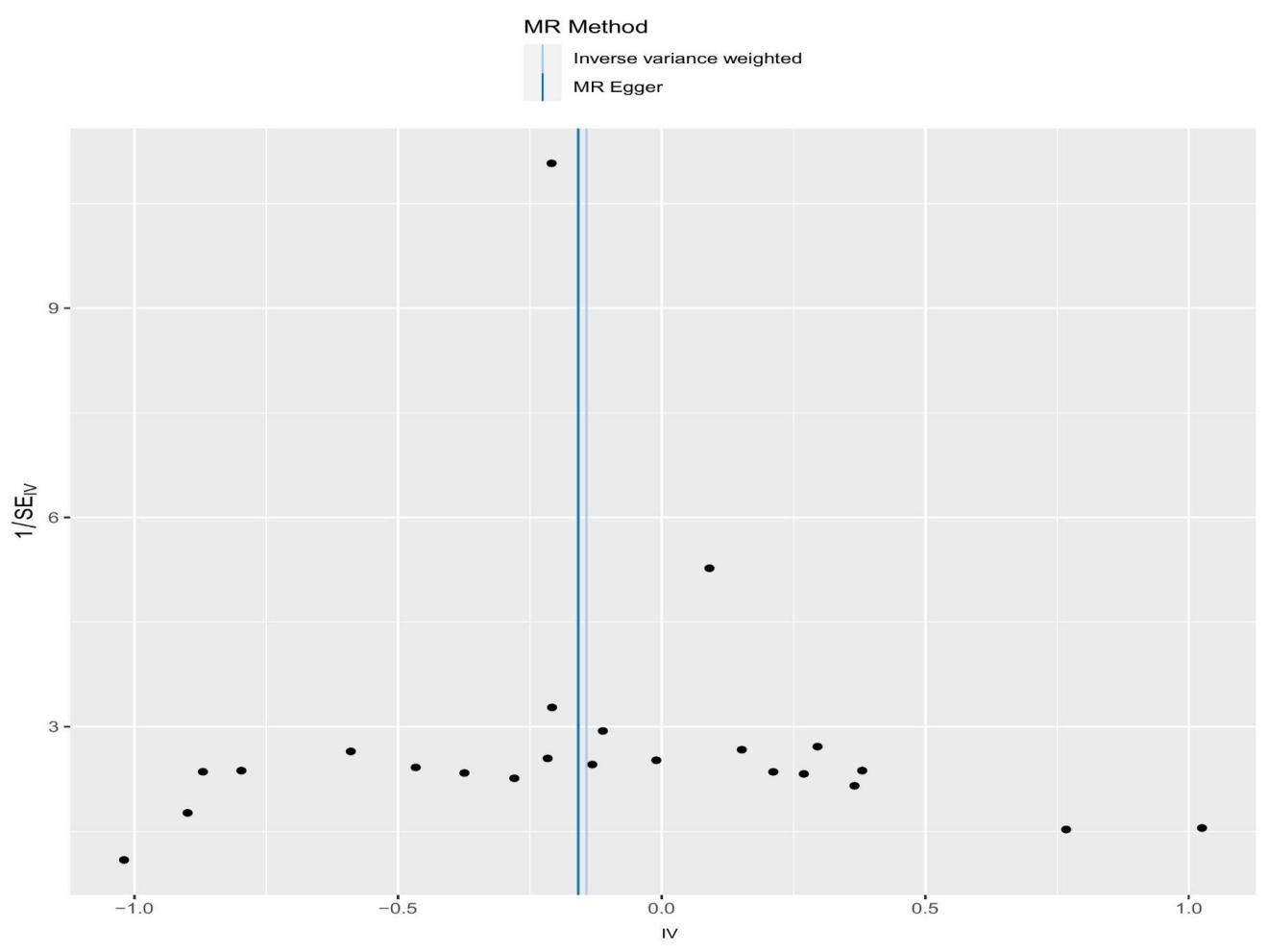


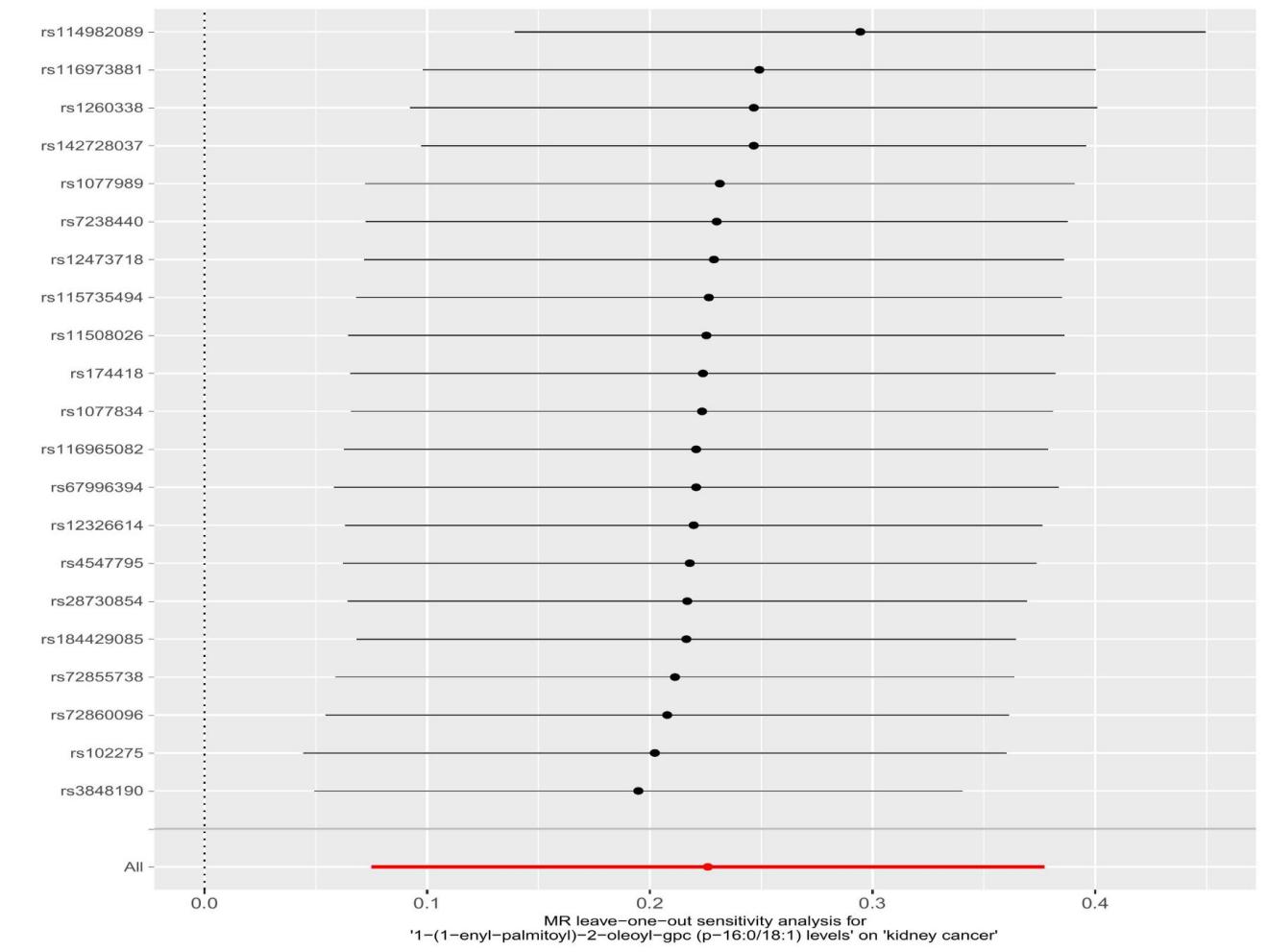


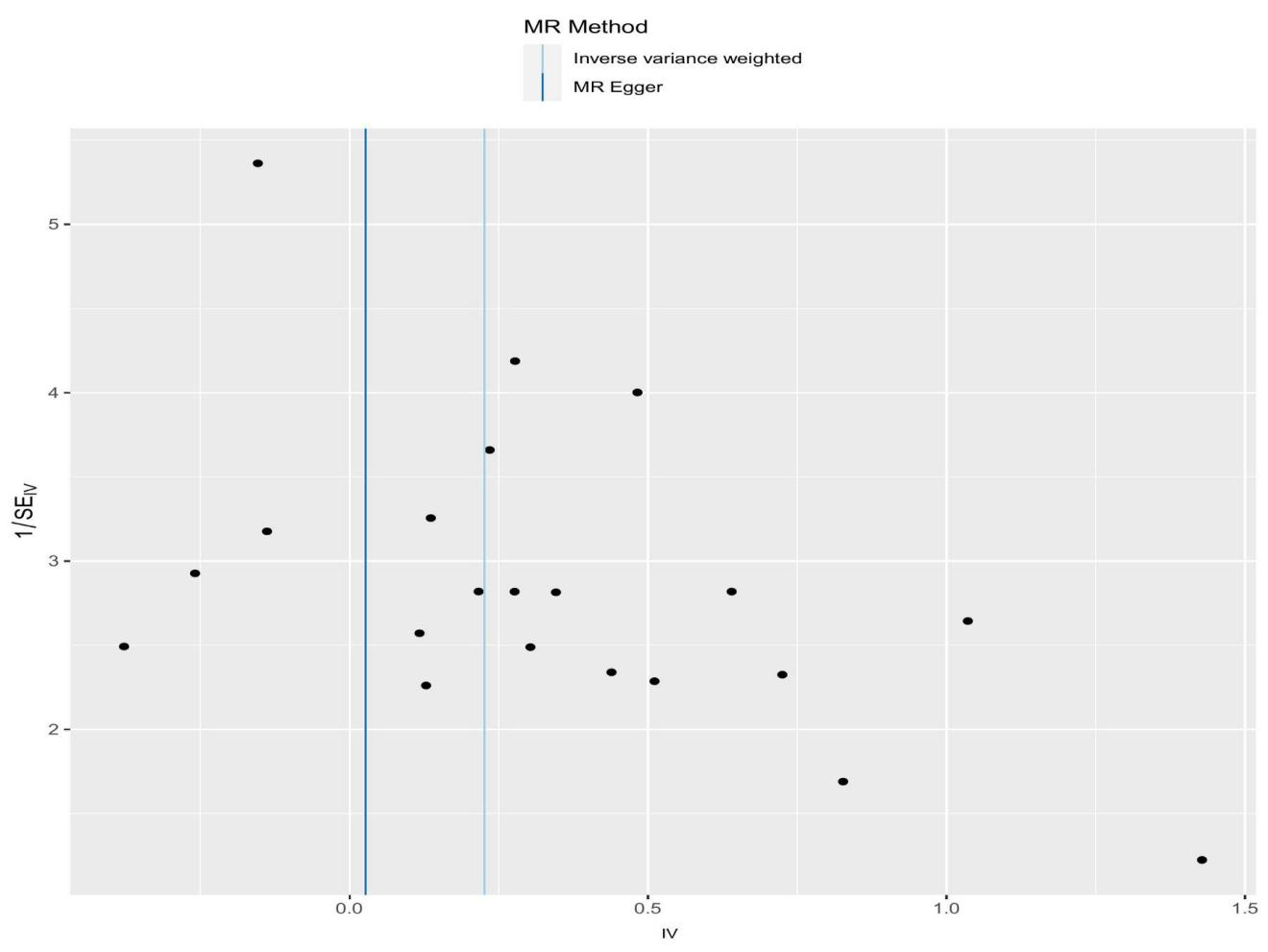


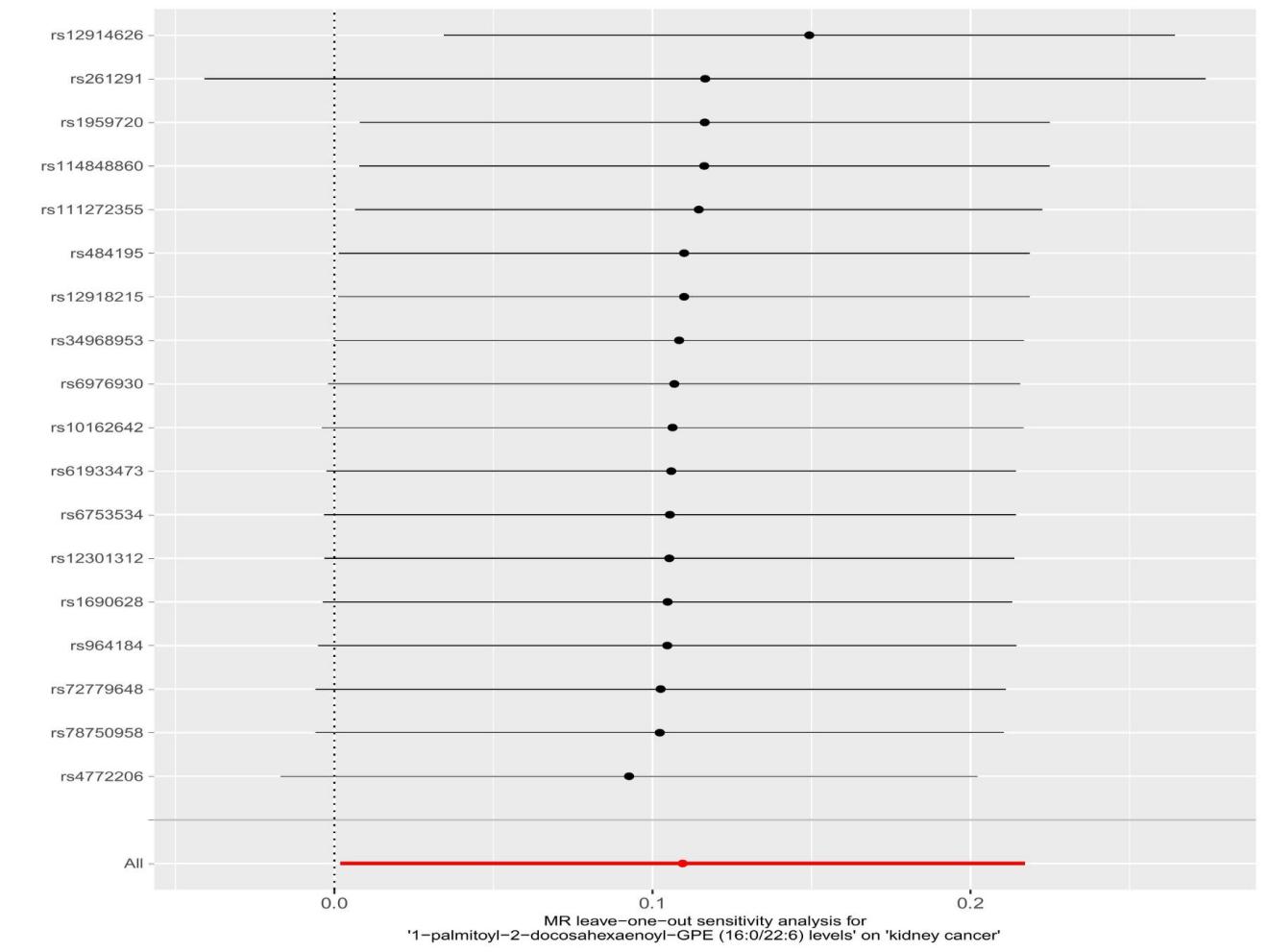


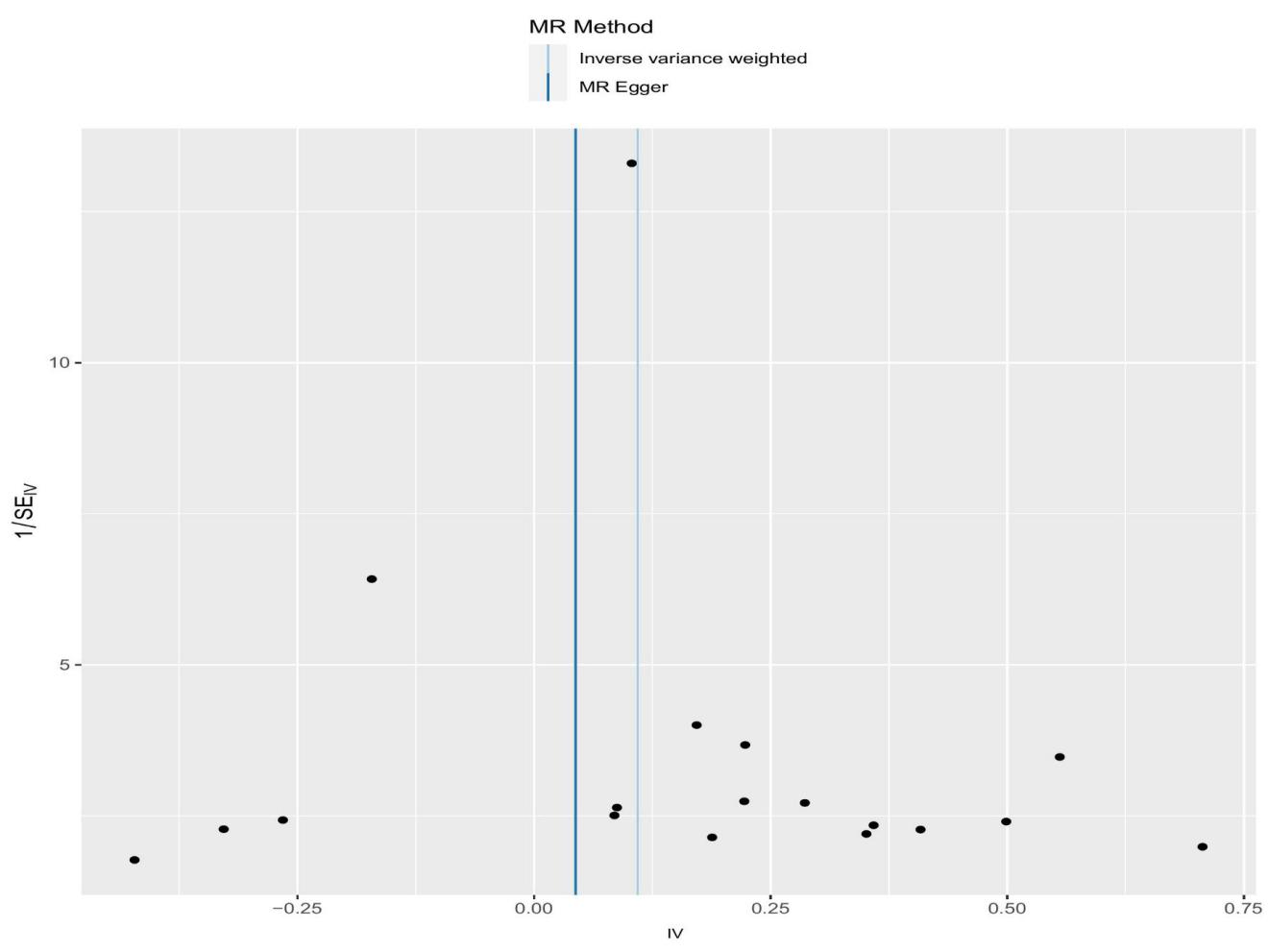


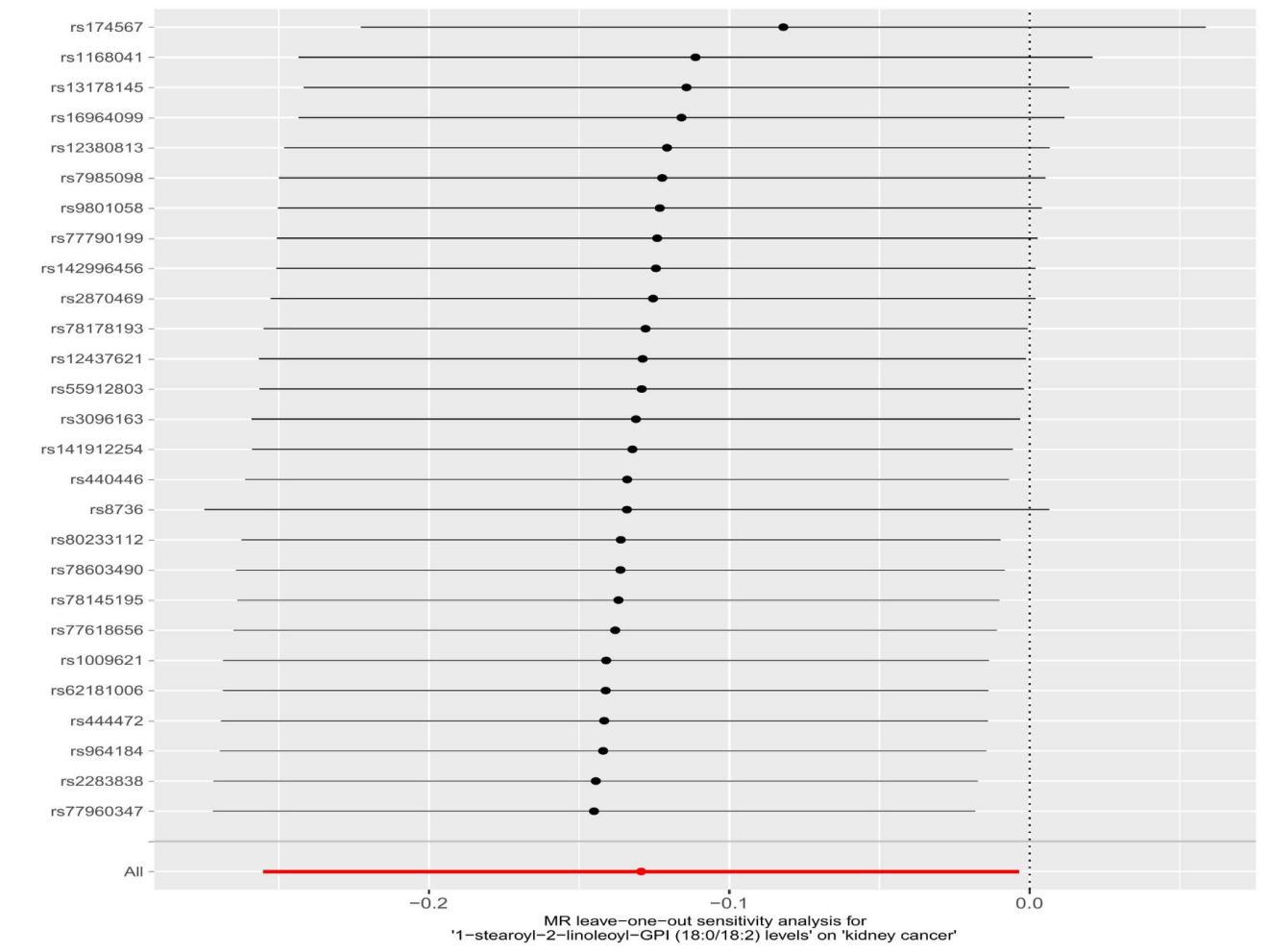


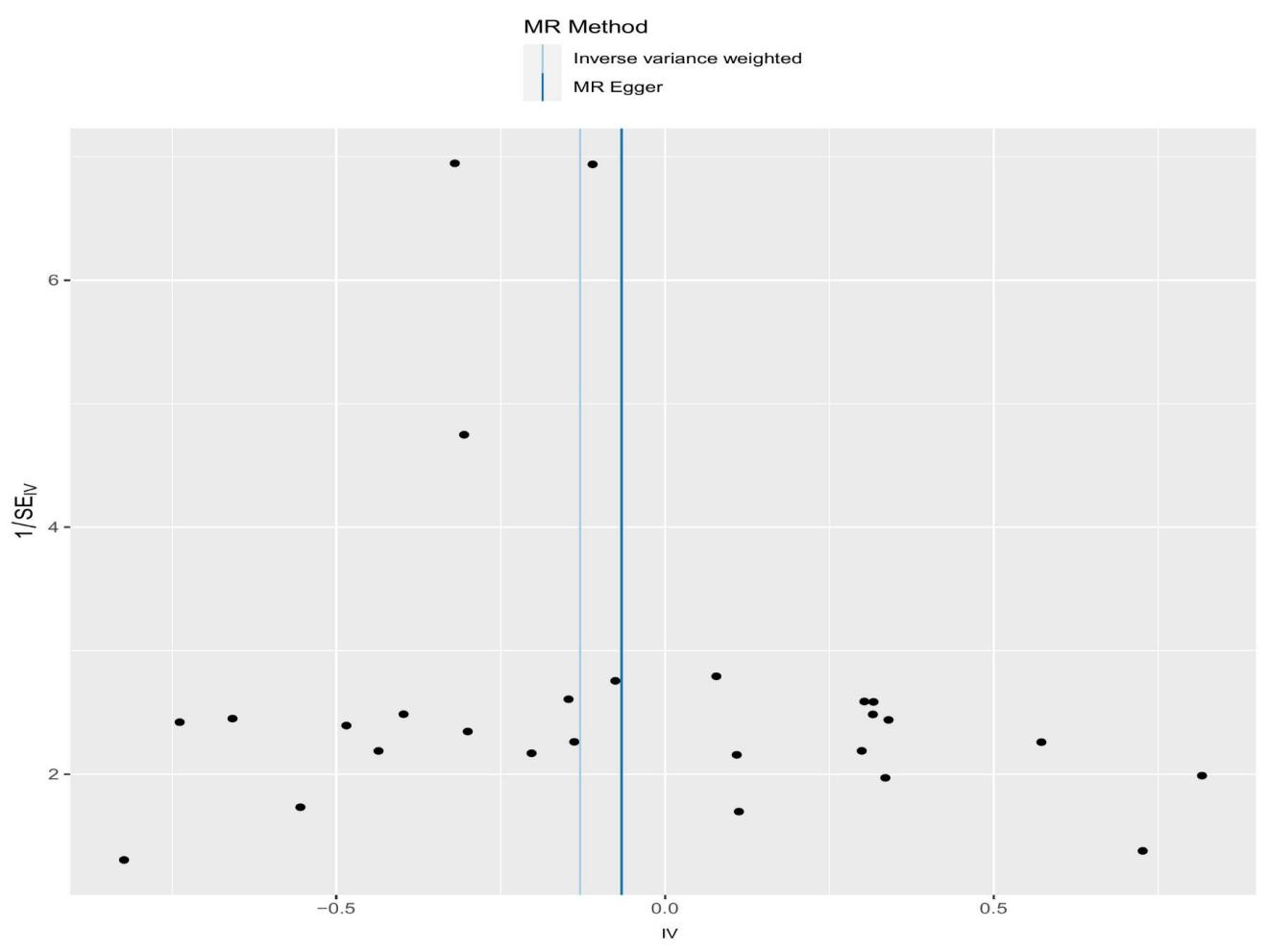


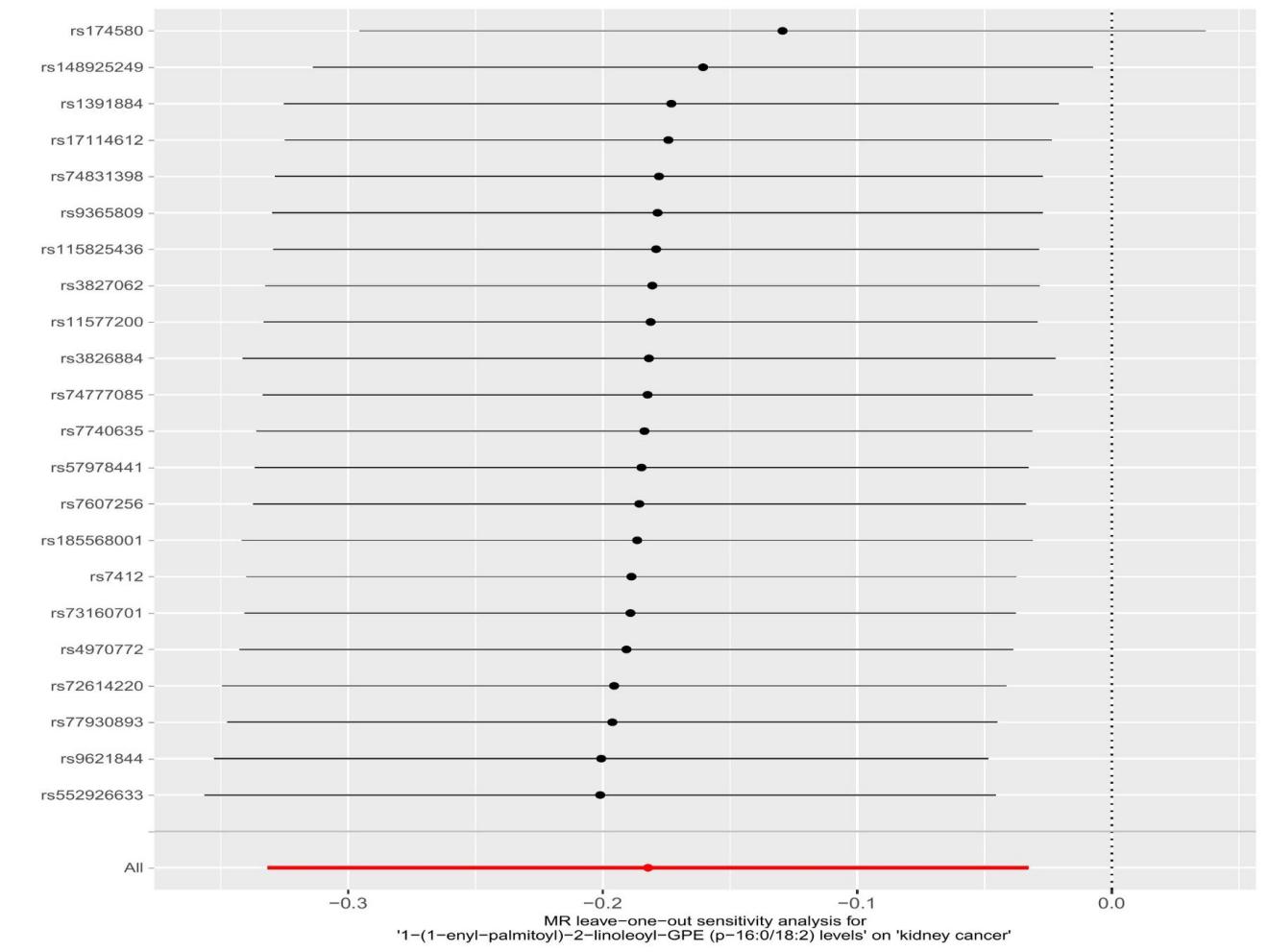


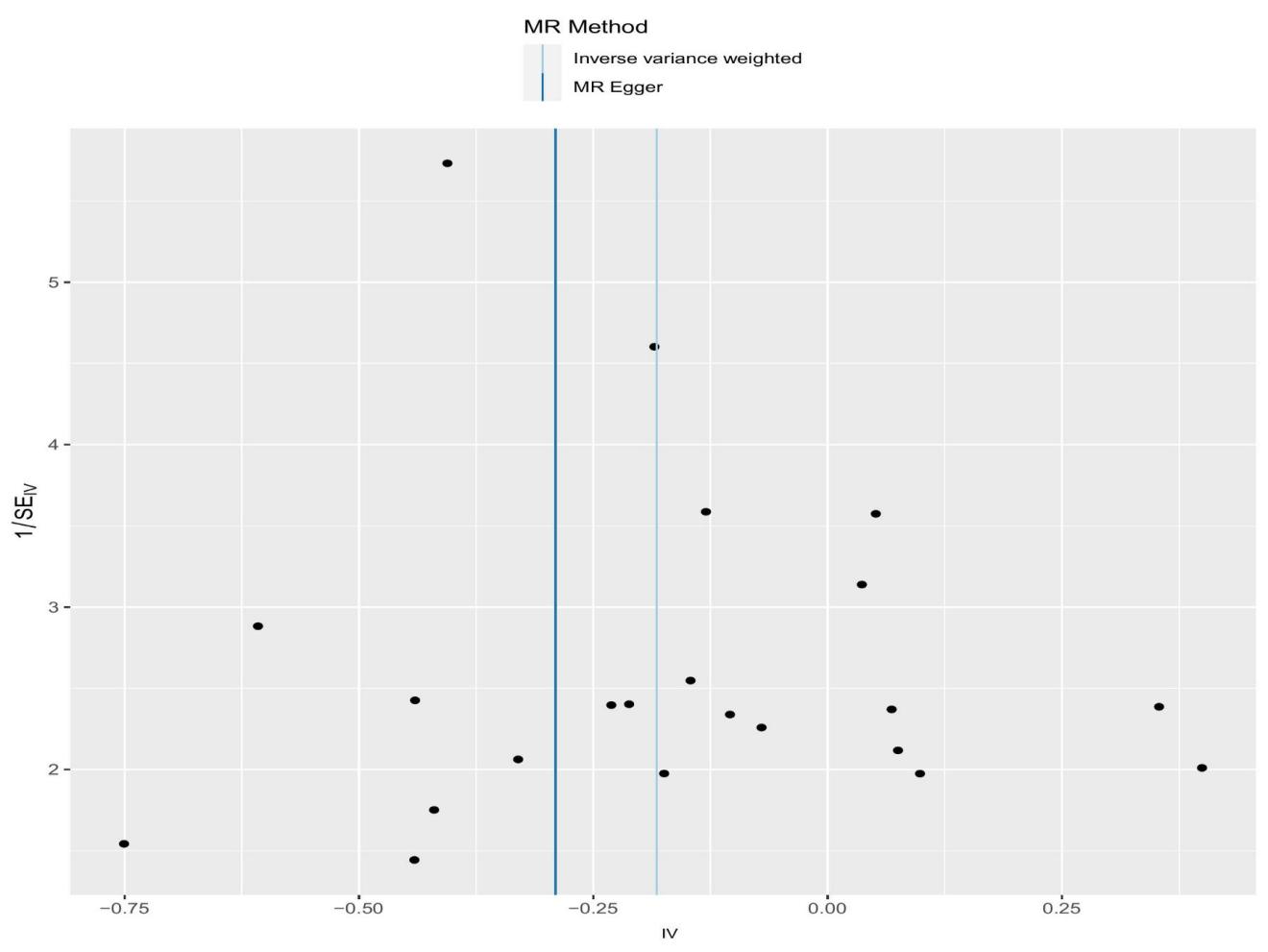


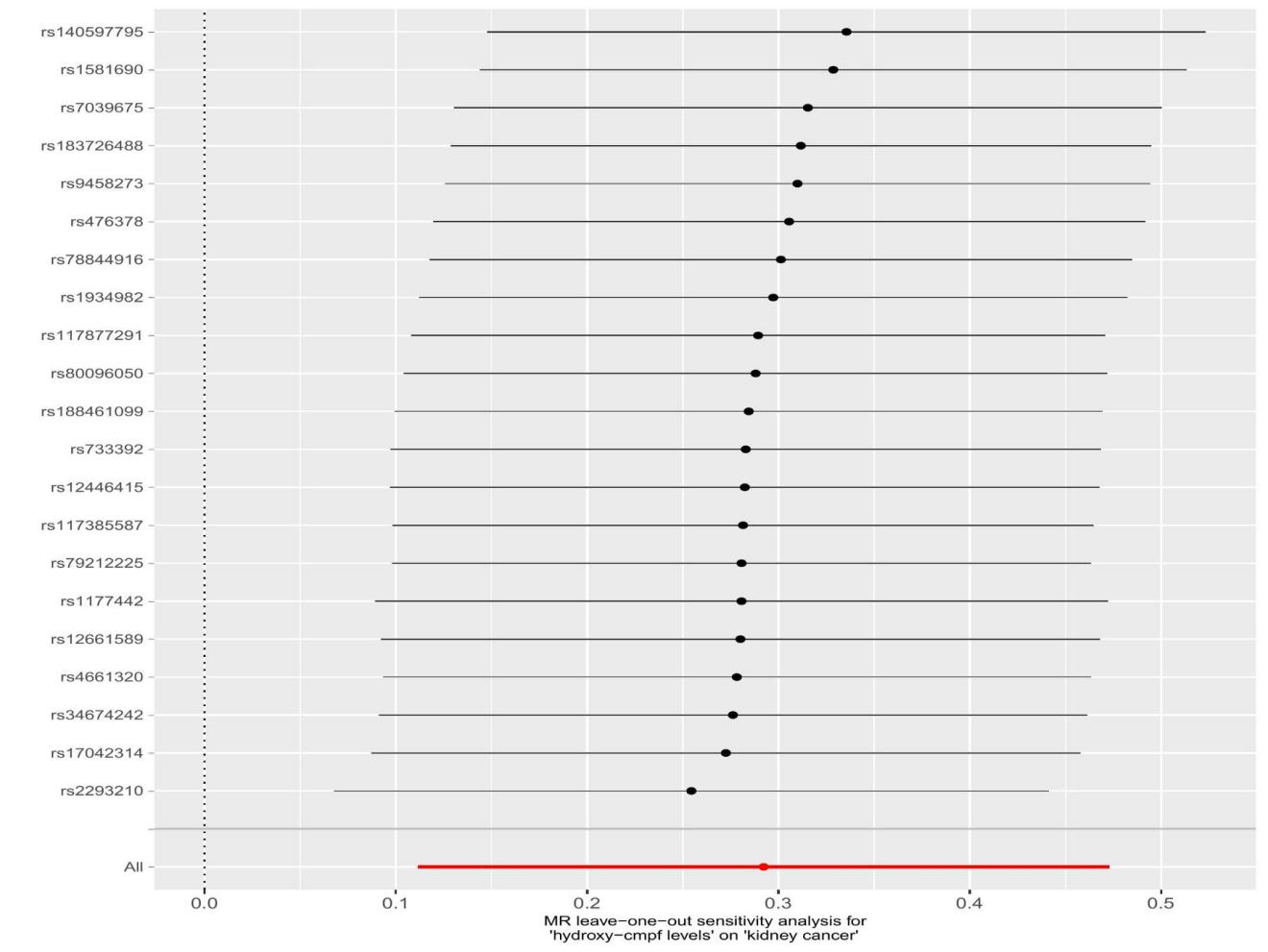


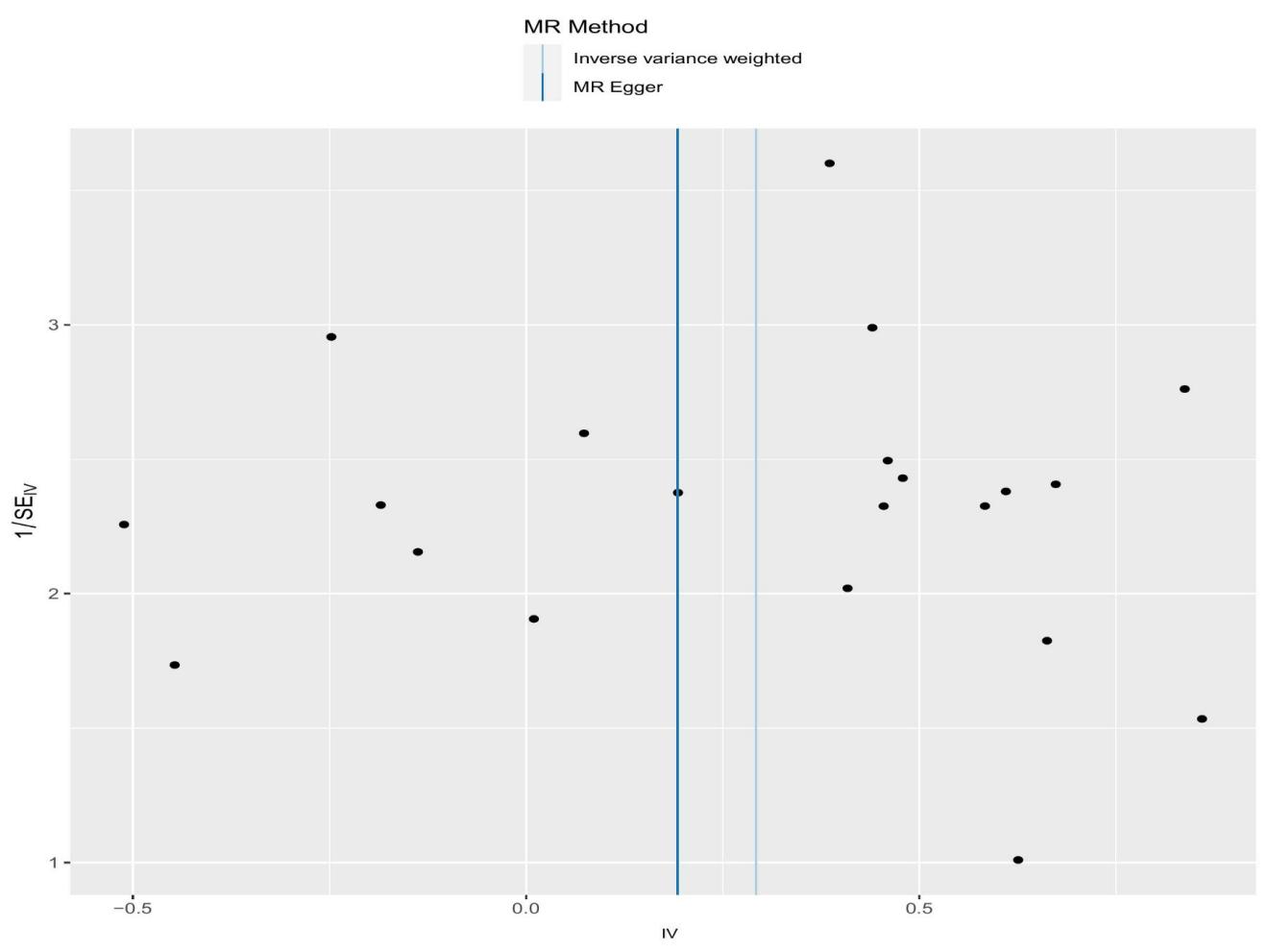


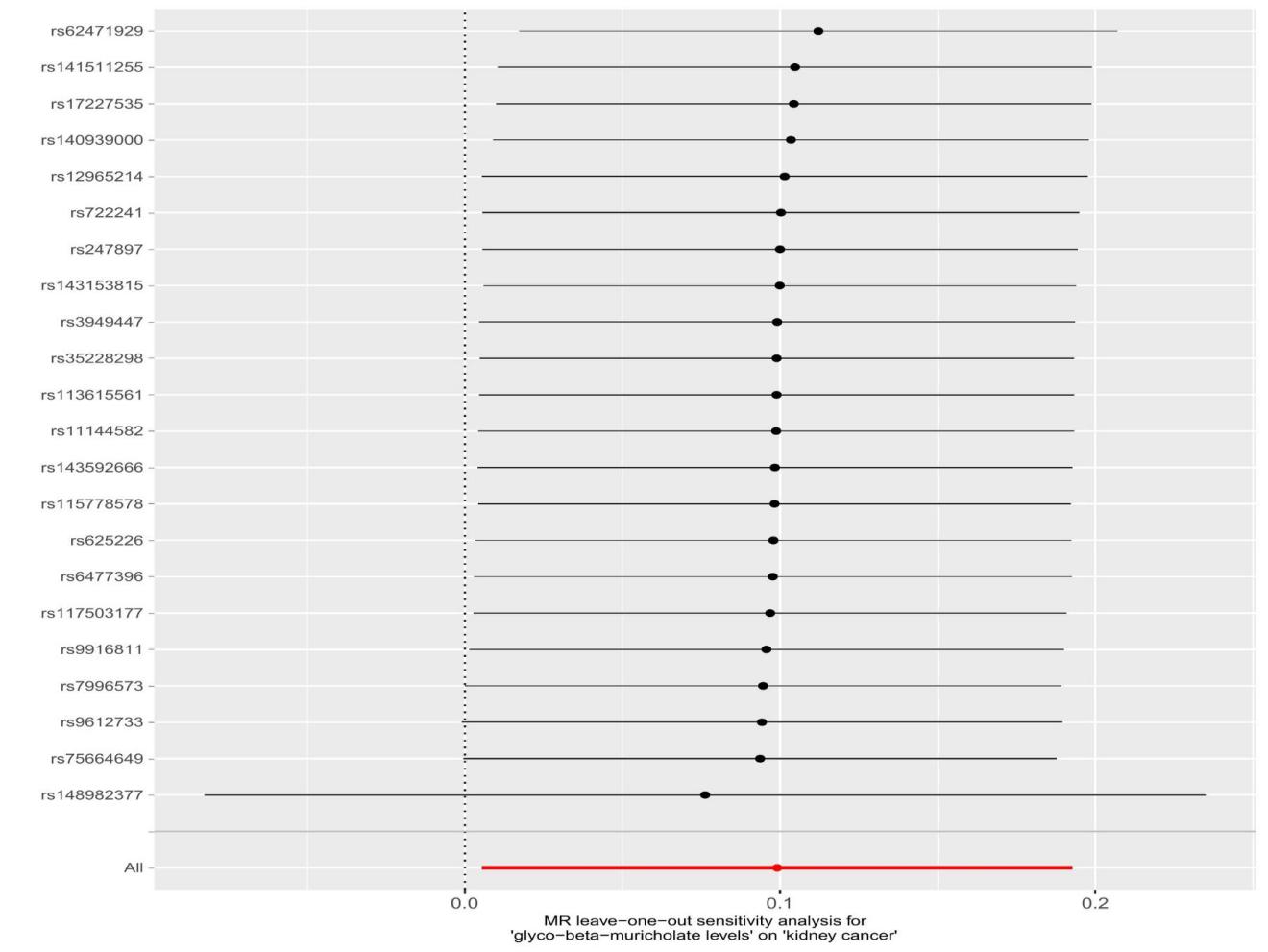


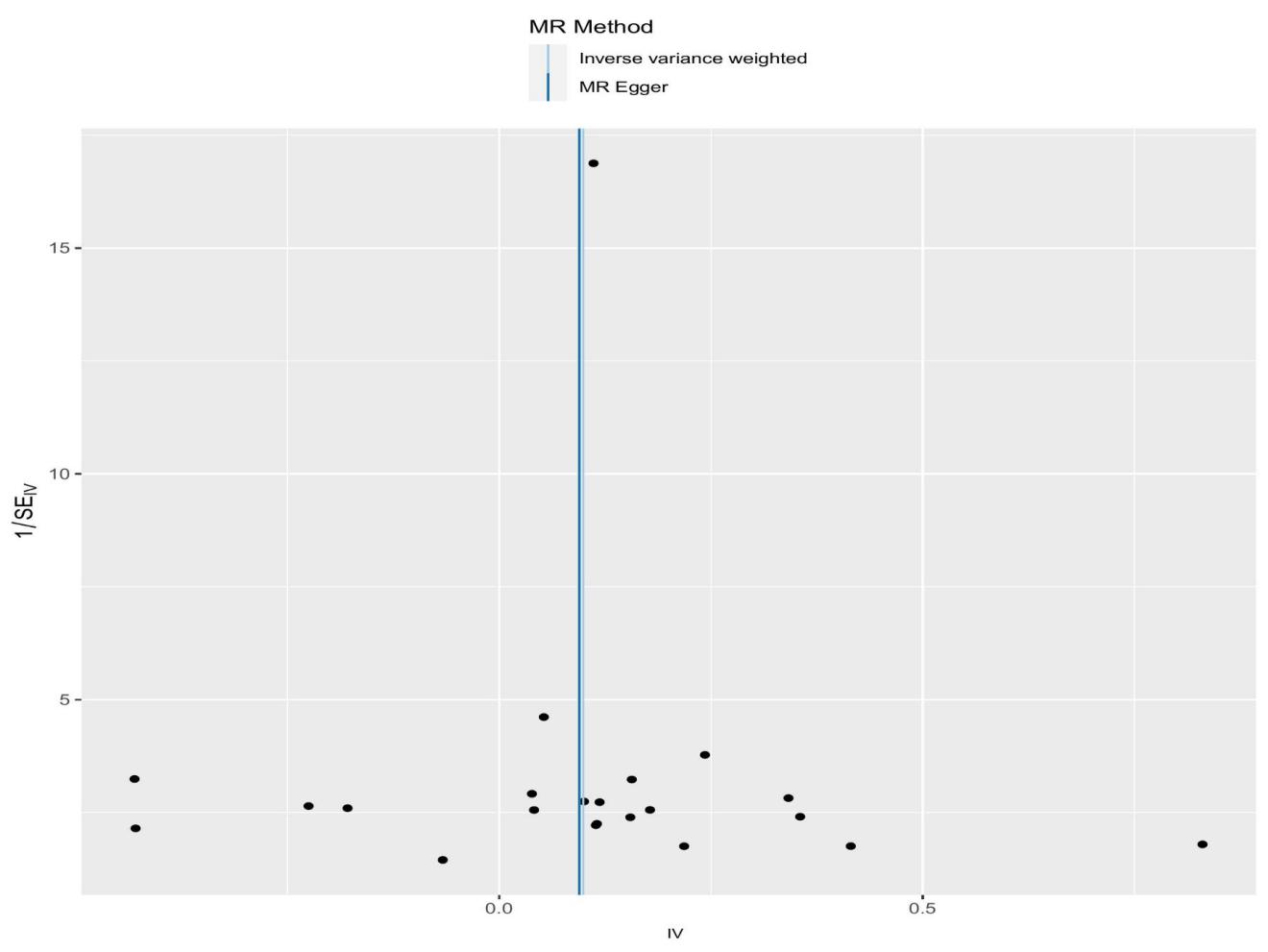


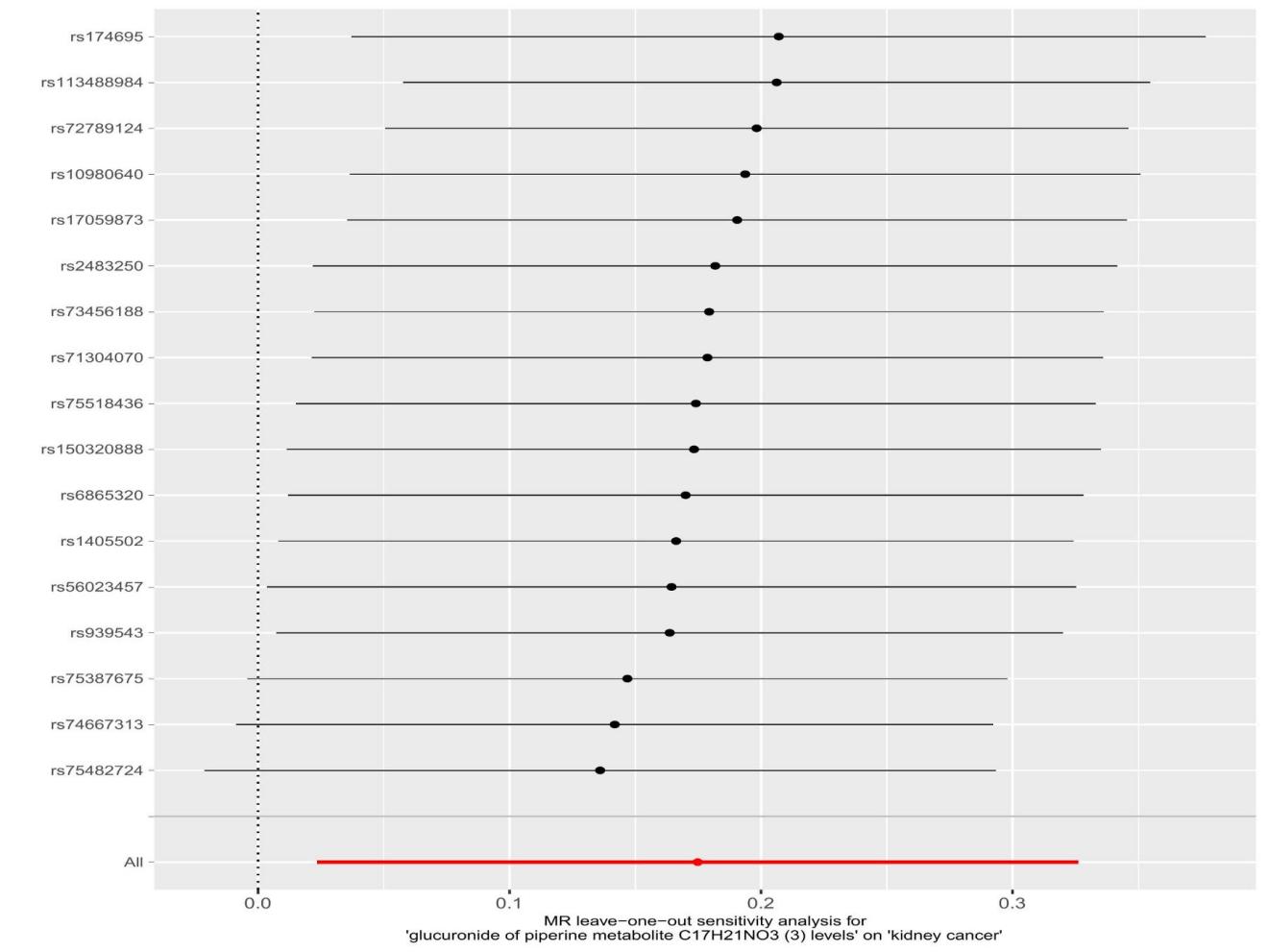


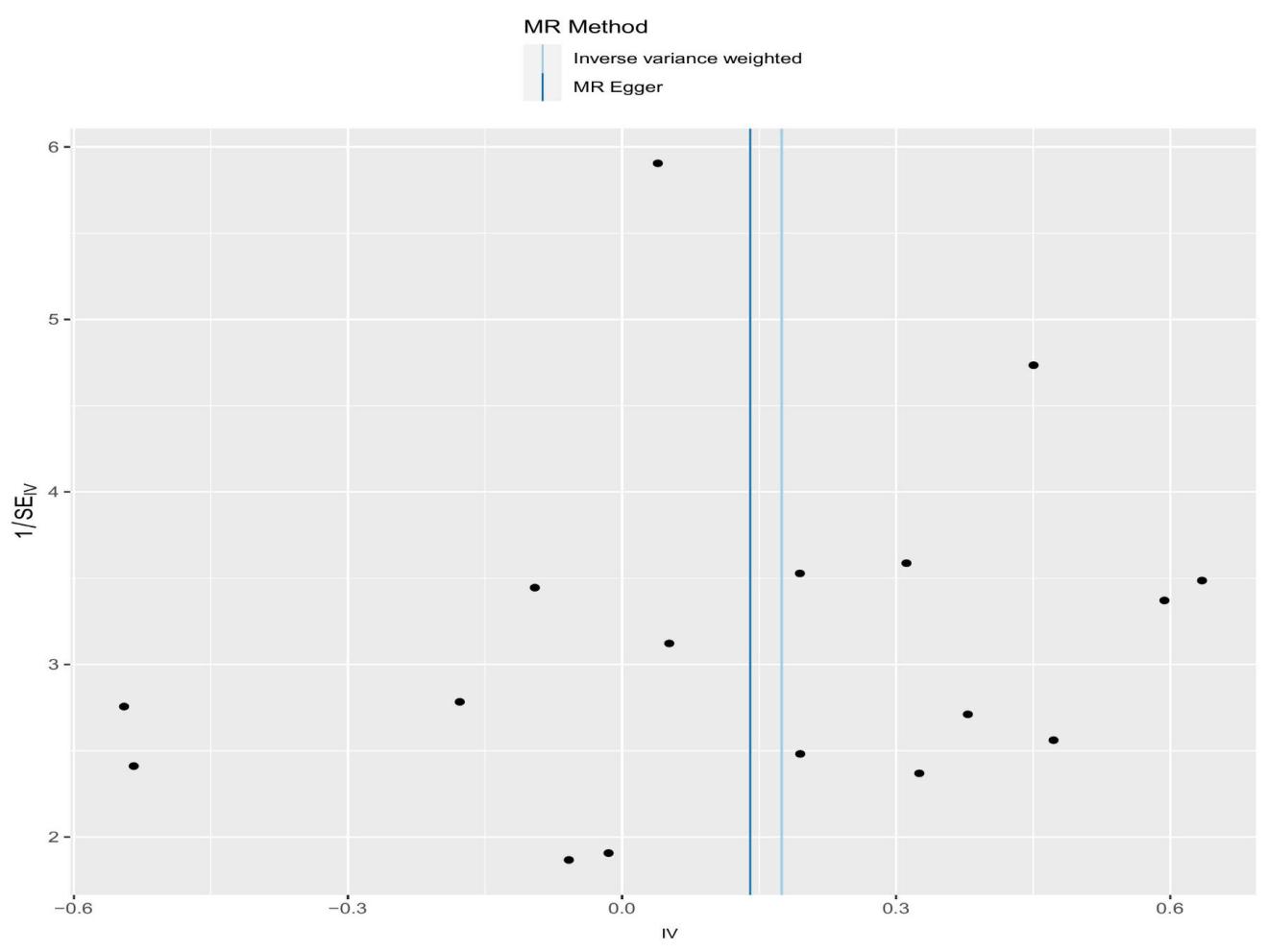


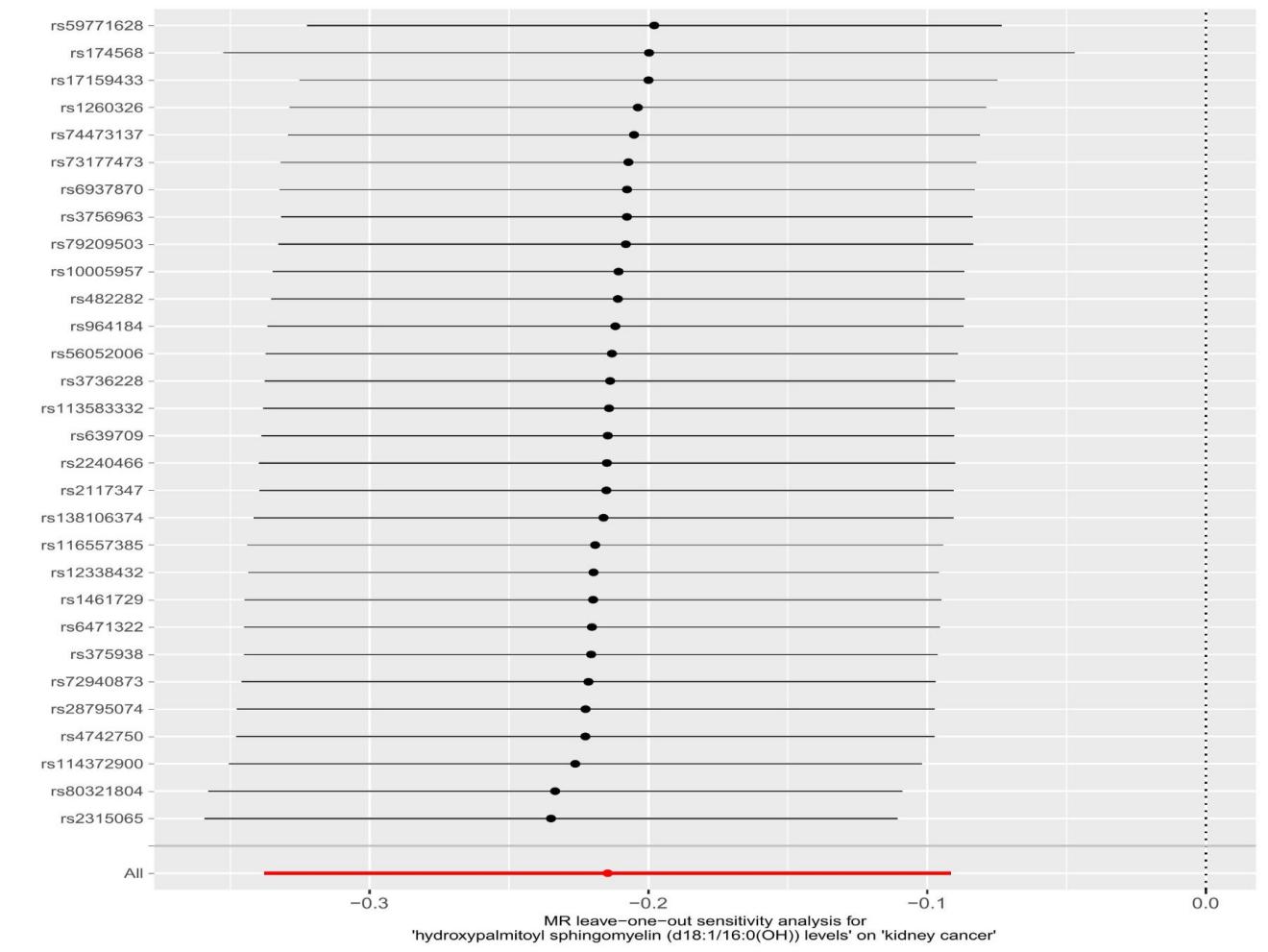


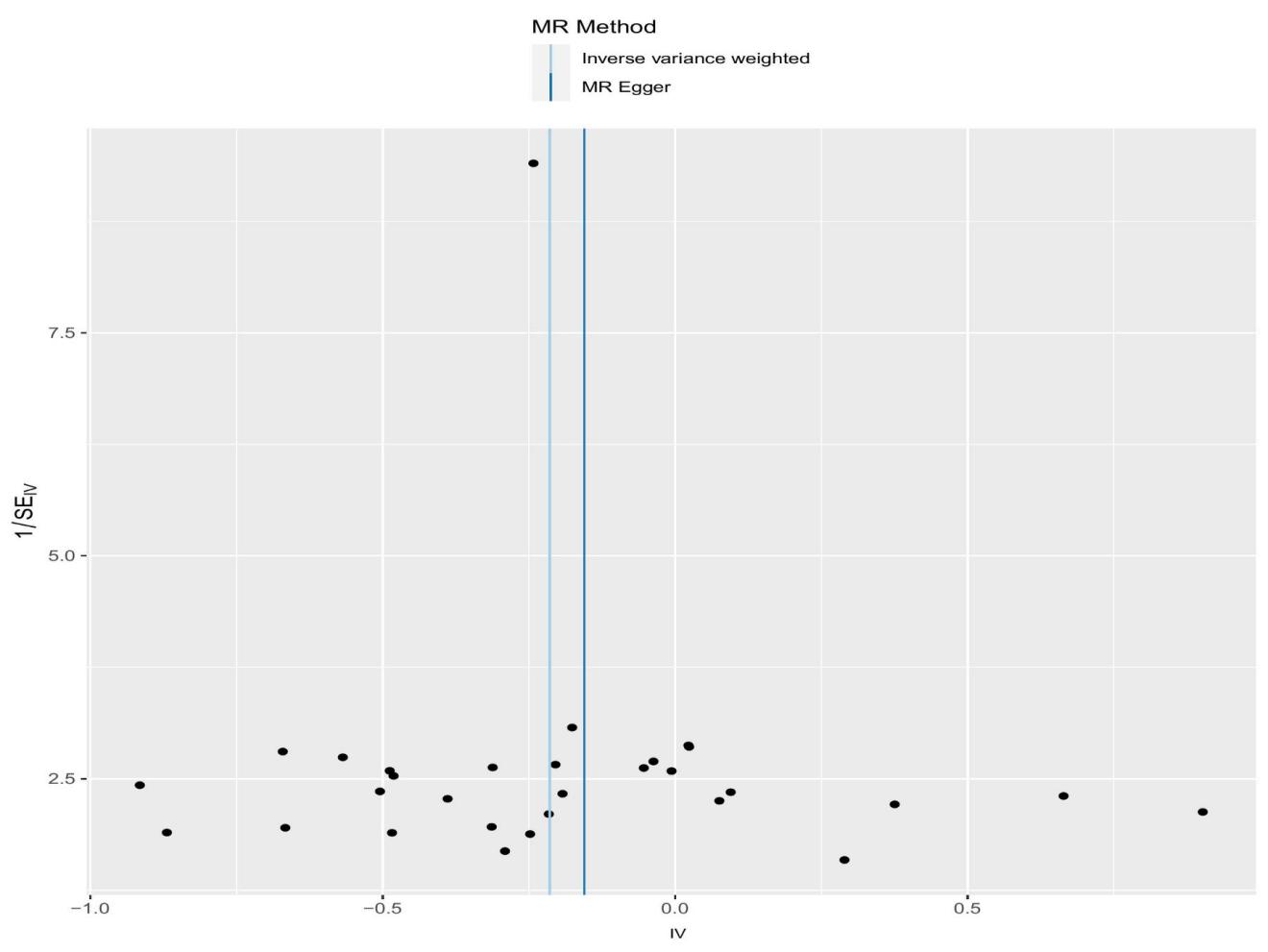


Prostate cancer

Testicular Carcinoma
